# *Exidia* and *Exidiopsis* (*Auriculariales*, *Basidiomycota*): a reconsideration

**DOI:** 10.3897/imafungus.17.189042

**Published:** 2026-07-29

**Authors:** Viacheslav Spirin, Vera Malysheva, Nathan Schoutteten, Anton Savchenko, Bernard Rivoire, Jan Olsson, Thomas Læssøe, Leif Ryvarden, Django Grootmyers, Matti Kulju, Josef Vlasák, María Victoria Vignale, Ilya Viner

**Affiliations:** 1 Finnish Museum of Natural History, University of Helsinki, PO Box 7, 00014 Helsinki, Finland Institute of Biological Sciences, University of Oslo Oslo Norway; 2 Komarov Botanical Institute, Prof. Popova str. 2, 197022 St. Petersburg, Russia Department of Ecology and Evolutionary Biology, University of Tennessee Knoxville United States of America; 3 Organismic Botany and Mycology, Institute of Plant Science and Microbiology, Ohnhorststraße 18, 22609 Hamburg, Germany Globe Institute, Department of Biology, University of Copenhagen Copenhagen Denmark; 4 Naturalis Biodiversity Center, Darwinweg 2, 2333 CR Leiden, Netherlands Instituto de Biotecnología Misiones (InBioMis), Facultad de Ciencias Exactas, Químicas y Naturales, Universidad Nacional de Misiones Misiones Argentina; 5 Société Linnéenne de Lyon, 33 rue Bossuet, 69006 Lyon, France Finnish Museum of Natural History, University of Helsinki Helsinki Finland; 6 Österplana Skattegården 2, 53384 Hällekis, Sweden Ecology and Genetics Research Unit, University of Oulu Oulu Finland; 7 Globe Institute, Department of Biology, University of Copenhagen, Universitetsparken 15, 2100 Copenhagen Ø, Denmark Naturalis Biodiversity Center Leiden Netherlands; 8 Institute of Biological Sciences, University of Oslo, P.O. Box 1045, Blindern, N-0316 Oslo, Norway Biology Centre, Academy of Sciences of the Czech Republic České Budějovice Czech Republic; 9 Department of Ecology and Evolutionary Biology, University of Tennessee, 1406 Circle Drive, Knoxville, Tennessee 37996, USA Komarov Botanical Institute St. Petersburg Russia; 10 Ecology and Genetics Research Unit, University of Oulu, P.O. Box 3000, FI-90014 Oulu, Finland Organismic Botany and Mycology, Institute of Plant Science and Microbiology Hamburg Germany; 11 Biology Centre, Academy of Sciences of the Czech Republic, Branišovská 31, CZ 37005, České Budějovice, Czech Republic Société Linnéenne de Lyon Lyon France; 12 Instituto de Biotecnología Misiones (InBioMis), Facultad de Ciencias Exactas, Químicas y Naturales, Universidad Nacional de Misiones, Ruta Nacional N12, km 7.5, Posadas, Misiones, CP3304, Argentina Unaffiliated Hällekis Sweden; 13 Instituto Misionero de Biodiversidad (IMiBio), Ruta Nacional N12, km 5, Puerto Iguazú, Misiones, CP3370, Argentina Instituto Misionero de Biodiversidad (IMiBio) Misiones Argentina

**Keywords:** Corticioid fungi, environmental sequences, heterobasidiomycetes, phylogeny, taxonomy

## Abstract

The taxonomy of the *Auriculariaceae* (*Auriculariales*, *Basidiomycota*) is revised based on morphological data and four- and three-marker datasets, with special emphasis on the *Exidia* clade. We argue in favor of retaining *Exidia* as a large genus, encompassing most species previously relegated to it, with an addition of four effused (*Exidiopsis*-like) species. Phylogenetic data allow us to justify two segregates from *Exidia* s. lato, namely the reinstated *Ulocolla* (*Exidia saccharina* complex) and a newly erected genus *Descidia*, encompassing *Exidia repanda* and *E. thuretiana*. At large, morphological data corroborate this splitting. *Exidiopsis effusa*, the generic type of *Exidiopsis*, is typified and redescribed; together with a newly described *E. perflua*, it constitutes the genus *Exidiopsis* s. str. The rest of *Exidiopsis* s. lato species studied by us are redistributed among the redefined genera *Adustochaete*, *Leiostroma*, *Proterochaete*, and *Ulocolla*, as well as a newly introduced genus, *Tegmenticium*. *Amphistereum* is placed among the synonyms of *Eichleriella* and the single representative of *Sclerotrema*, *Exidiopsis griseobrunnea*, is moved into *Ulocolla*. In total, 52 species are properly redescribed or introduced below, of them twenty are new to science, and twenty new combinations are proposed. Additionally, we detected two single-species lineages: *Pholiobasidion senex***gen. nov. et sp. nov**., from North America, which is recovered as the sister group to all other members of the *Auriculariaceae* sampled, and *Scrupulispora perparvula***gen. nov. et sp. nov**., from Europe.

## Introduction

*Exidia* Fr. is one of the oldest genera introduced among jelly fungi (heterobasidiomycetes). Having been separated from *Tremella* Pers. mainly via macroscopic features (orbicular basidiomata with clearly differentiated abhymenial and hymenial surfaces – Fries 1822), the genus originally encompassed not only genuine *Exidia* spp. but also many species currently assigned to *Auricularia* Bull. Brefeld (1888) made the first attempt to redefine *Exidia* on the basis of anatomical traits and stressed four-celled, longitudinally septate basidia and cylindrical basidiospores as the most important microscopic differentiating characters of the genus. In the same work, he introduced a new subgenus, *Exidiopsis*, for the single species with resupinate basidiomata, Exidia (Exidiopsis) effusa Bref. This subgenus was technically raised to the genus rank by Vuillemin (1890) and then redescribed by Möller (1895) for a wider set of species. However, most authors did not accept *Exidiopsis* (Bref.) Vuillemin as a genus and continued to use the generic epithet *Sebacina* Tul. & C. Tul. for nearly all effused fungi with longitudinally septate basidia (cf. Bresadola 1892, 1908; Bourdot and Galzin 1927; Neuhoff 1936a, 1936b). Based on meticulous microscopic studies, Luck-Allen (1960, 1963) and Wells (1959, 1961) proved that *Sebacina* s. lato represents a heterogeneous assemblage of highly divergent resupinate heterobasidiomycetes. As a result, *Sebacina* was restricted exclusively to the clampless species while taxa with clamped hyphae were rearranged in other genera – in particular, in the reinstated *Exidiopsis*.

Recent DNA studies showed that most of the described *Exidia* and *Exidiopsis* spp., including their type species, belong to the family *Auriculariaceae* Fr. ex Lindau (Weiß and Oberwinkler 2001; Wells et al. 2004; Malysheva and Spirin 2017). The *Auriculariaceae* were first discovered as a strongly supported clade of the *Auriculariales* by Weiß and Oberwinkler (2001, as ‘*Exidia / Auricularia* group’). While the family-level classification of the other lineages of the *Auriculariales* is yet to be reestablished, the *Auriculariaceae* showed up as a monophyletic group with strong or very strong support in all subsequent phylogenetic studies (Wells et al. 2004; Malysheva and Spirin 2017; Malysheva et al. 2018; Liu et al. 2022; Spirin et al. 2025). Alongside *Auricularia*, *Exidia* and *Exidiopsis* s. lato, this clade encompasses a number of other taxa, previously attributed to *Eichleriella* Bres. and *Heterochaete* Pat. (Malysheva and Spirin 2017; Alvarenga et al. 2019; Li et al. 2023), as well as the currently accepted poroid genera, such as *Aporpium* Bondartsev & Singer ex Singer, *Elmerina* Bres., and *Protodaedalea* Imazeki (Sotome et al. 2014; Malysheva et al. 2018). Wells et al. (2004) first re-introduced the family name *Auriculariaceae* for this group.

Weiß and Oberwinkler (2001) were the first to investigate this group using molecular data. They relied solely on nuclear ribosomal sequences and showed that *Auricularia* and *Exidia* were likely to be polyphyletic. However, the phylogenetic signal was too weak to provide robust support, and it was evident that nuclear ribosomal markers alone have limited value for generic reclassification within the family. Nevertheless, all subsequent attempts to revise the genus-level classification of the family relied solely on these markers. Several genera for corticioid and stereoid representatives of the *Auriculariaceae* were recently described or reinstated, although usually without a critical overview of the remaining taxa (Malysheva and Spirin 2017; Yuan et al. 2018; Alvarenga et al. 2019; Liu et al. 2022; Li et al. 2023; Dong et al. 2025a, 2025b). It has therefore become clear that an all-encompassing revision of the family based on additional genetic markers is needed.

The use of multigene datasets was so far sporadic in reconstructing the phylogeny of the family. Wu et al. (2021) claimed that *Auricularia* is monophyletic based on a four-marker (ITS + LSU + *RPB1 + RPB2*) phylogeny of the family. However, only seven other taxa outside *Auricularia* were included in the dataset; of them, only *Elmerina efibulata* (Y.C. Dai & Y.L. Wei) Y.C. Dai & L.W. Zhou had all four markers while six others were represented only by ITS and LSU regions. In our opinion, such a gappy approach undermined their conclusions and thus left the monophyly of *Auricularia* questionable. At the same time, a few recently published species-level studies of *Exidia* also referred to multigene datasets although the *RPB1* gene was abandoned and, in some cases, replaced by *TEF1* sequences (Wu et al. 2020; Ye et al. 2020; Tohtirjap et al. 2023; Gruhn et al. 2024). In all these studies, only *Exidia* species were included in the final datasets and phylogenetic trees; therefore, its monophyly, as well as phylogenetic relationships with other members of the *Auriculariaceae* were not properly investigated.

In the present study, we aimed at the extensive species sampling of the *Auriculariaceae* and completing a full four-marker (ITS + LSU + *RPB1 + RPB2*) dataset for the family. In total, 46 species from the family were included in the final four-marker dataset, of which fourteen are the currently accepted generic types or their closest relatives. All four markers were generated for the first time for eleven described species of *Exidia* s. lato and fifteen resupinate species traditionally assigned to *Exidiopsis*, *Heterochaete* and *Eichleriella* s. lato. Additionally, we inferred a three-marker (ITS + LSU *+ RPB2*) phylogeny of the *Auriculariaceae* with a broader set of taxa (49 species), as well as twelve species-level ITS-, ITS + LSU, or ITS + *TEF1*-based phylogenies for six accepted genera.

## Material and methods

### Morphological study

Collections and type specimens from herbaria H, GB, LE, S, O, C, GENT, OULU, LD, L, UPS, TAAM, PC, LY, K, BPI, FH, NY, PH, MPU, HBG, TNS, GJO, W, CWU, TUF, SLP, EA were studied. Herbarium acronyms are given according to Thiers (2025). Microscopic routine follows Spirin et al. (2025). In total, 10–20 context / subicular and subhymenial hyphae, 10–20 basidia, and 20–35 basidiospores were measured per each specimen examined. All measurements were made in Cotton Blue, with the use of oil immersion and phase contrast illumination (Leitz Diaplan microscope, ×1250). All dried specimens with gelatinous or partly gelatinized basidiomata were rehydrated in water for 1–10 hours before making microscopic slides. After preparing a slide, it was left for several hours to be properly mounted in CB for the subsequent examination. The following abbreviations are used in the taxonomic part of the paper: L – average basidiospore length, W – average basidiospore width, Q – average L/W ratio, n – number of measurements per specimens measured. In microscopic descriptions, the wall thickness of hyphae, hymenial cells and basidiospores is defined according to Spirin et al. (2024a). In the macroscopic descriptions of *Exidia* s. lato species, the overall thickness of basidiomata refers to their total extension from the attachment to the basidiome uppermost / lowermost point. In section, basidiomata of *Exidia* s. lato species rarely exceed 1–2 mm thick; however, their strongly folded or lobate basidiomata are a reason for much higher values of the basidiome thickness given in the species descriptions below. Therein, the term “probasidium” refers to a juvenile, not septate basidial cell (basidiole). Mature basidia of all taxa revised in the present study are longitudinally septate; therefore, this trait is not specified in the morphological descriptions. All specimens examined (except for the types) are listed in Suppl. material 1.

### DNA isolation and sequencing

Genomic DNA was extracted from herbarium specimens and axenic cultures using a CTAB–chloroform protocol (Kutuzova et al. 2017). We used both standard primers and primers specifically developed for this study to amplify the complete nuc rDNA ITS1–5.8S–ITS2 region (ITS) and the LSU, *RPB1*, *RPB2*, and *EF*-1α markers (Table 1). After amplification, PCR products were run on a 1.5% agarose gel stained with GelRed (Biotium, Fremont, California) and visualized under UV light. PCR products were then purified using thermosensitive alkaline phosphatase combined with Exonuclease I (Thermo Fisher Scientific, Vantaa, Finland). Sequencing was performed on an ABI 3730XL DNA Analyzer (Applied Biosystems) by Macrogen (Amsterdam, the Netherlands). Several ITS and LSU sequences of *Exidia* spp. were obtained at the Komarov Botanical Institute (St. Petersburg, Russia); for sequencing protocols, see Spirin et al. (2024b). ITS sequences of specimens from Belgium, Luxembourg, and the Netherlands were generated at Ghent University (Ghent, Belgium), following protocols described in Schoutteten et al. (2023).

**Table 1.** Primers used in this study.

**Primer name**	**Target DNA marker**	**Binding site**	**Direction**	**Reference**
JS1	28S	28S	fwd	Landvik (1996)
LR5	28S	28S	rev	Vilgalys and Hester (1990)
LR7	28S	28S	rev	Hopple and Vilgalys (1994)
ITS1	ITS, ITS1	18S	fwd	White et al. (1990)
ITS1F	ITS, ITS1	18S	fwd	Gardes and Bruns (1993)
ITS5	ITS, ITS1	18S	fwd	White et al. (1990)
ITS4	ITS, ITS2	28S	rev	White et al. (1990)
ITS2	ITS1	5.8S	rev	White et al. (1990)
LR22	ITS1, ITS2	28S	rev	https://sites.duke.edu/vilgalyslab/rdna_primers_for_fungi
58A1F	ITS2	5.8S	fwd	Martin and Rygiewicz (2005)
ITS3	ITS2	5.8S	fwd	White et al. (1990)
RPB1 Af	RPB1	Domain A	fwd	Stiller and Hall 1997
RPB1_Au_intf*	RPB1	Intron 2 (between domains A and B)	fwd	this study
RPB1_Au_intr**	RPB1	Intron 2 (between domains A and B)	rev	this study
RPB1-Cr	RPB1	Domain C	rev	Matheny et al. 2002
RPB1-Cr Hyme***	RPB1	Domain C	rev	this study
RPB1-Int2.1f****	RPB1	Intron 2 (between domains A and B)	fwd	Frøslev et al. 2005
RPB1-Int2.1fr****	RPB1	Intron 2 (between domains A and B)	rev	Frøslev et al. 2005
RPB2-f5F	RPB2	Domain 5	fwd	Liu et al. (1999)
RPB2-6F	RPB2	Domain 6	fwd	Matheny (2005)
RPB2-7R2	RPB2	Domain 7	rev	Matheny et al. 2007
1567R	TEF1-α	~1567 bp	rev	Rehner and Buckley (2005)
1567R-Xylo	TEF1-α	~1567 bp	rev	Spirin et al. (2026)
2212R	TEF1-α	~2212 bp	rev	Rehner and Buckley (2005)
983F	TEF1-α	~983 bp	fwd	Rehner and Buckley (2005)
983F-Xylo	TEF1-α	~983 bp	fwd	Spirin et al. (2026)

* Sequence: AACTTCGCCGACAAGGTCCG. Better performance than standard primers for the target group. ** Sequence: CGGACCTTGTCGGCGAAGTT. Better performance than standard primers for the target group. *** Sequence: CCDGCRATRTCRTTRTCMATRTA. Better performance than standard primers for the focal group; also performs well across different lineages of the unrelated order *Hymenochaetales*. **** Poor performance for the target group, both as PCR and sequencing primers.

Deciphering and assembling chromatograms followed Viner et al. (2021). All newly generated sequences of acceptable quality, including those not used in the phylogenetic analyses, were deposited in GenBank (Suppl. material 2). In the species descriptions below and Suppl. material 1, sequenced specimens are marked by an asterisk.

Additionally, the type specimens of four taxa, viz. *Exidia cinnamomescens* Raitv., E. saccharina f. carnea Raitv., *Exidiopsis griseobrunnea* K. Wells & Raitviir *s. str*. and E. griseobrunnea ssp. macrogyna K. Wells & Raitviir (all in TAAM), were sequenced. DNA was extracted using the CTAB method according to the protocol in S2 by Schenk et al. (2023), with the following modifications: CTAB buffer with 1% PVP-10, no beta-mercaptoethanol, no vortexing, lysis and precipitation steps extended overnight. The ITS region was amplified with the primer pair ITS1ngs (Tedersoo et al. 2015) / ITS4 (White et al. 1990) adapted for Nanopore sequencing: ONT_ITS1ngs TTTCTGTTGGTGCTGATATTGCTCCGTAGGTGAACCTGC and ONT_ITS4 ACTTGCCTGTCGCTCTATCTTCTCCTCCGCTTATTGATATGC, respectively. Phire II HS Thermo F Taq Polymerase was used; PCR program: 98 °C 30 s, 35×(98 °C 5 s, 54 °C 5 s, 72 °C 15 s), 72 °C 5 m. Nanopore library was prepared with a custom combination of Native Barcoding kits SQK-NBD114.24 (outer barcodes) and SQK-NBD114.96 (inner barcodes) and sequenced on PromethION flow cell (FLO-PRO114M). Demultiplexing was made with Dorado v1.3.1, and consensus building with NGSpeciesID v0.3.1. ITS sequences of these four type specimens are available in UNITE (Abarenkov et al. 2024).

### Taxon sampling for phylogenetic analyses

In addition to our newly produced data, we retrieved relevant sequences from GenBank (Benson et al. 2018) and UNITE (Kõljalg et al. 2013). We also used three publicly available genomes (Floudas et al. 2012; Looney et al. 2013; Nagy et al. 2016; Mesny et al. 2021) from the Fungal Genomics Resource (https://mycocosm.jgi.doe.gov) and GenBank, as of December 1^st^ 2025. The JGI genome of *Aporpium caryae* (Schwein.) Teixeira & D.P. Rogers was misidentified and belongs to *Elmerina phellinoides* Vlasák & Ryvarden; therefore, the latter name is used in the phylogenetic trees. Alignments were produced with MAFFT v7.429 (https://mafft.cbrc.jp/alignment/server/) using the L-INS-i strategy (Katoh et al. 2017) and were manually adjusted where the machine-derived alignment clearly violated the homology principle. The final alignments are summarized in Suppl. material 3.

To determine the appropriate generic placement of the taxa studied, we constructed a comprehensive family-level dataset using four DNA markers (ITS, LSU, *RPB1*, and *RPB2*). Independent analyses of these genetic markers were conducted to assess dataset congruence; resulting topologies were either similar or remained unresolved, depending on the marker (not shown). The outgroup taxa (*Pseudohydnum gelatinosum* (Scop.) P. Karst. and *Protoacia delicata* Spirin & Malysheva) were chosen among the residual *Auriculariales* (Spirin et al. 2023).

For several focal genera and species groups, we used reliably aligned ITS + LSU or ITS-only datasets to provide phylogenetic resolution. Additionally, this approach enabled us to make full use of available ITS data from public databases and provided better phylogenetic context (all datasets are listed in Suppl. material 3). The outgroups were selected based on the highest pairwise similarity of the focal clades to sequences in public databases. Several species-level trees (*Leiostroma* Fr., *Exidiopsis* s. str., *Exidia pithya* complex, and *Ulocolla saccharina* complex) were midpoint-rooted as all available outgroup sequences exhibited substantially greater divergence from the ingroup than the ingroup taxa showed among themselves. This resulted in disproportionately long outgroup branches in preliminary analyses. The full alignments with annotated excluded characters were deposited at TreeBASE (TB2:S32514).

We inferred phylogenetic trees with Maximum Likelihood (ML) and Bayesian Inference (BI). Nucleotide substitution models for BI were chosen with ModelTest-NG 0.2.0 (Darriba et al. 2020) based on the Bayesian information criterion (BIC). Altogether we set seven partitions for the family-level alignment (ITS, LSU, *RPB1* and *RPB2*). Exons of the protein-coding genes were further divided into 1–2 vs. 3 codon positions. All introns were discarded from further analyses except for a fragment of the long *RPB1* intron between conserved amino acid motifs A and B (as designated in Stiller and Hall 1997). We performed BI using MrBayes 3.2 (Ronquist et al. 2012). In these analyses, three parallel runs with four chains each and other default parameters were run for ten million generations. A burn-in of 25% was used in the final analyses. The average standard deviation of split frequencies had reached < 0.01 for all data sets. When depicting phylograms, the support for nodes is indicated when posterior probabilities are ≥ 0.90. For ML analyses, IQ-TREE 2.3.6 (Minh et al. 2020) was used. Bootstrapping was performed using the standard nonparametric bootstrap algorithm with the number of replicates set to 1000. Support for nodes is indicated with bootstrap values ≥ 50.

## Results

**Figure 1.**
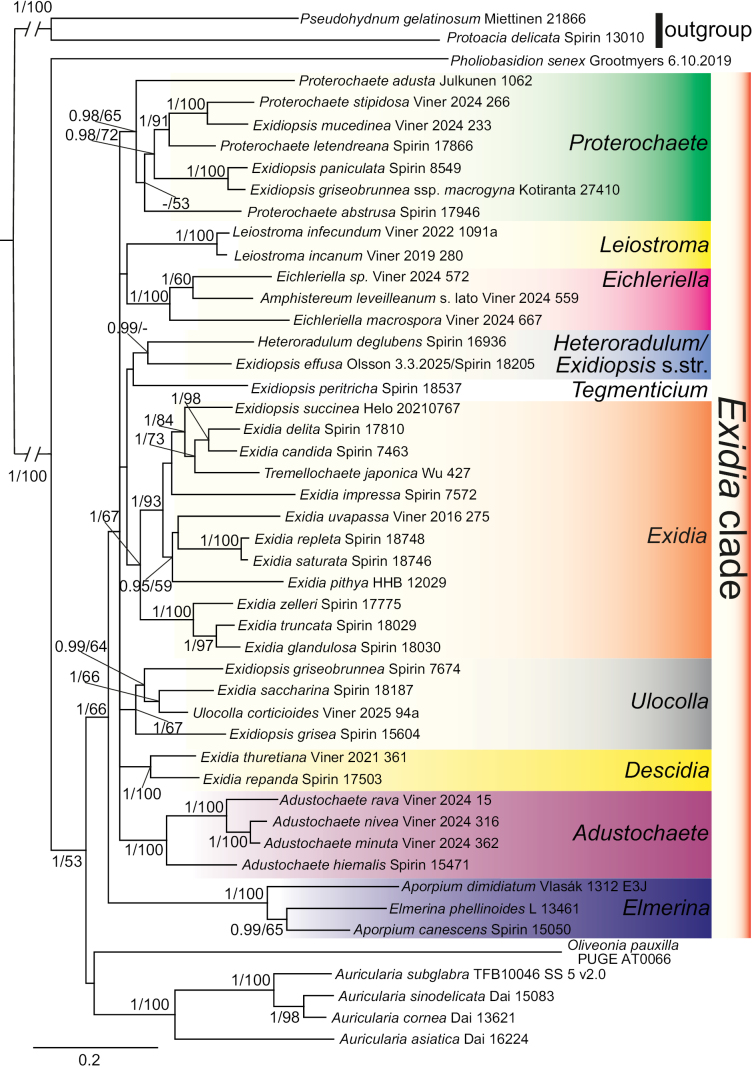
Phylogeny of the *Auriculariaceae* inferred from the ITS + LSU + *RPB1* + *RPB2* concatenated dataset using BI analysis. Bayesian posterior probabilities followed by ML bootstrap values are shown at nodes; branch lengths reflect estimated number of changes per site.

**Figure 2.**
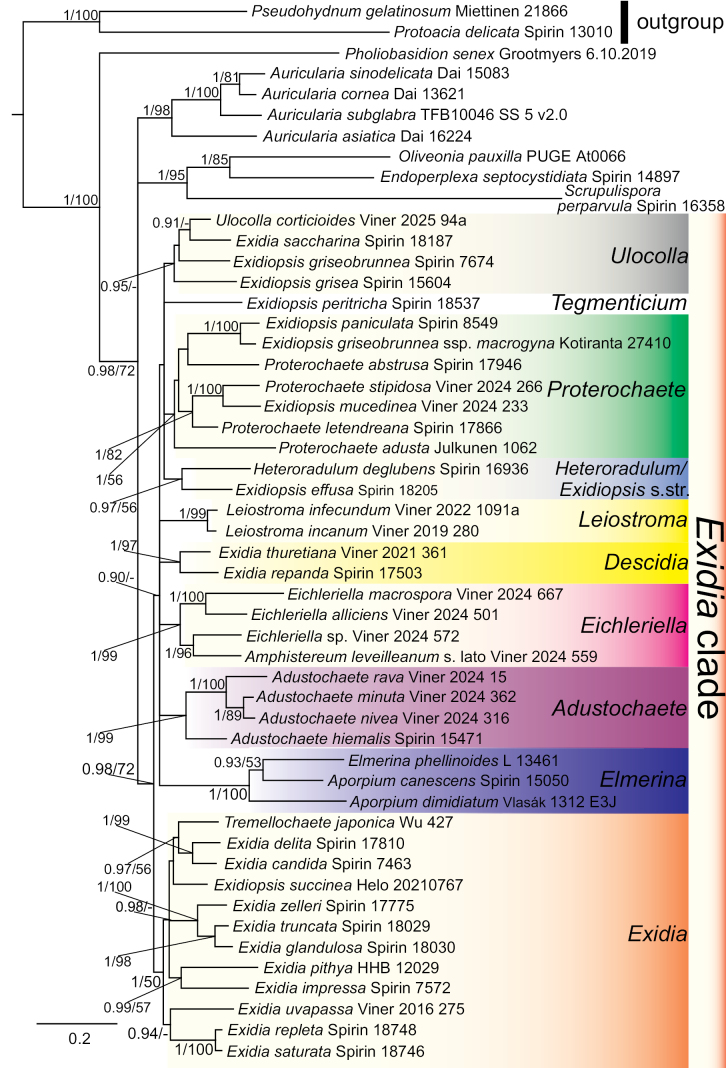
Phylogeny of the *Auriculariaceae* inferred from the ITS + LSU + *RPB2* concatenated dataset using BI analysis. Bayesian posterior probabilities followed by ML bootstrap values are shown at nodes; branch lengths reflect estimated number of changes per site.

In the resulting four-marker (ITS + LSU + *RPB1* + *RPB2*) phylogeny (Fig. 1), the *Auriculariaceae* are divided into four major lineages: (1) the single-species *Pholiobasidion* lineage (introduced as a new genus below), (2) the *Oliveonia* clade, represented by *Oliveonia pauxilla* (H.S. Jacks.) Donk s. lato, (3) the *Auricularia* clade, with four species, and (4) a large *Exidia* clade, containing the rest of the *Auriculariaceae*. The three-marker (ITS + LSU + *RPB2*) phylogeny with a slightly broader taxon sampling reveals the same overall structure of the family (Fig. 2); we discuss it in more detail below. Both the *Auricularia* and *Exidia* clades are strongly supported (Fig. 1) and therefore showed here to be monophyletic groups. However, the taxonomic interpretation of the phylogeny creates certain difficulties. The easiest option would be to recognize these four lineages as separate genera. This looks unproblematic for *Pholiobasidion* and *Auricularia*, both morphologically easily definable, and, to some extent, for *Oliveonia* Donk (see discussion on the three-marker dataset below). Although supported by the phylogenetic evidence, lumping all species of the *Exidia* clade into one genus seems to be hard to justify. Morphological diversity in the *Exidia* clade is so high that, if accepted as one genus, it becomes nearly indistinguishable from some genera outside the *Auriculariaceae* (cf. *Myxarium* and *Protohydnum* spp. with sessile basidia), as well as several taxa outside the *Auriculariales* (see under Excluded and insufficiently known species). In other words, the *Exidia* clade interpreted as one genus lacks any unique feature or a combination of traits which would allow its morphological recognition within the family and the order. We see this option undesirable and therefore opt for a more differentiated approach.

The four-marker dataset of the *Auriculariaceae* splits the *Exidia* clade into ten distinct lineages (Fig. 1). We briefly characterize these clades as they are represented in Fig. 1; at the present moment, the best taxonomic option which we advocate below is to accept them as separate genera.

**Figure 3.**
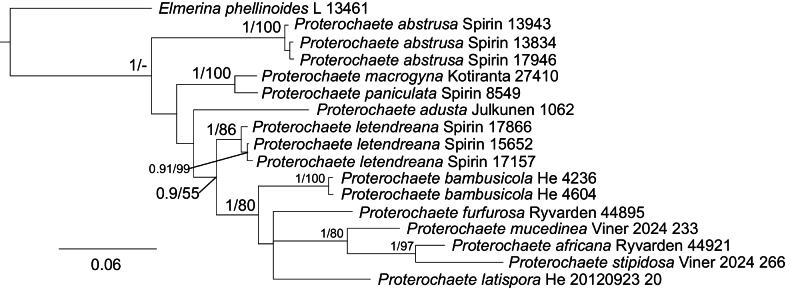
Phylogeny of *Proterochaete* inferred from the ITS + LSU concatenated dataset using BI analysis. Bayesian posterior probabilities followed by ML bootstrap values are shown at nodes; branch lengths reflect estimated number of changes per site.

**Figure 4.**
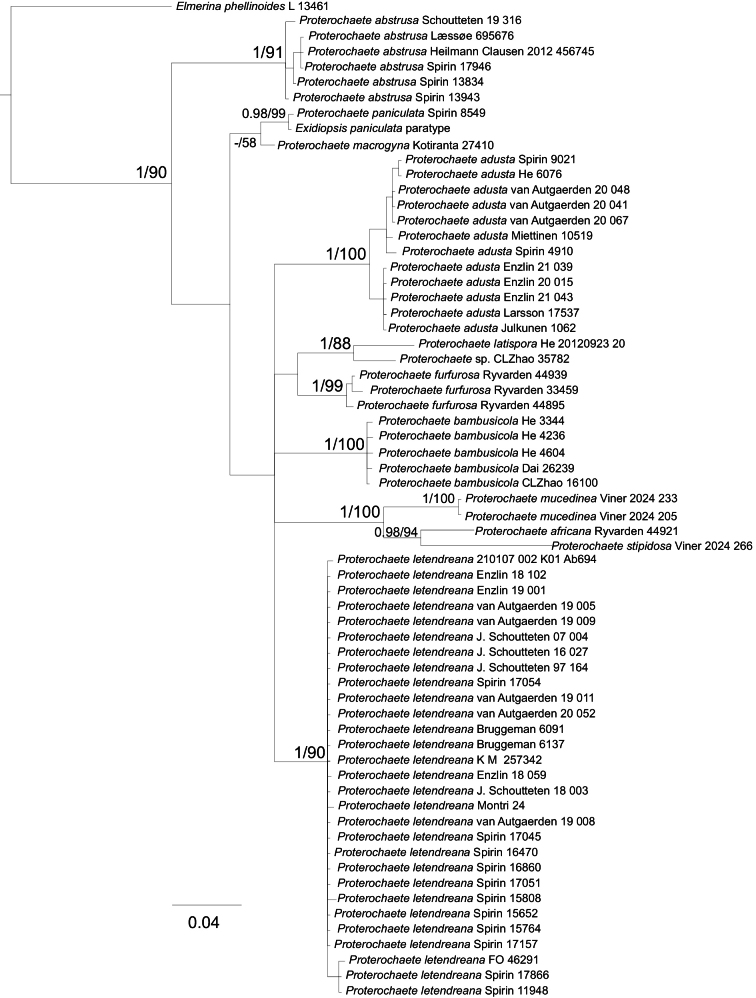
Phylogeny of *Proterochaete* inferred from the ITS dataset using BI analysis. Bayesian posterior probabilities followed by ML bootstrap values are shown at nodes; branch lengths reflect estimated number of changes per site.

**Figure 5.**
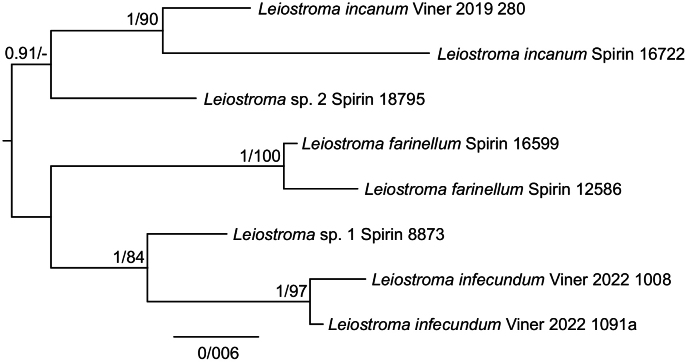
Phylogeny of *Leiostroma* inferred from the ITS + *TEF1* concatenated dataset using BI analysis. Bayesian posterior probabilities followed by ML bootstrap values are shown at nodes; branch lengths reflect estimated number of changes per site.

**Figure 6.**
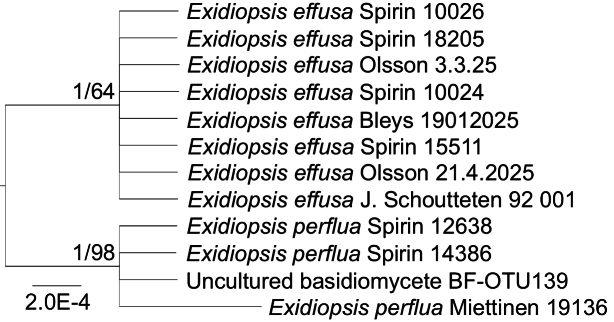
Phylogeny of *Exidiopsis* s. str. inferred from the ITS dataset using BI analysis. Bayesian posterior probabilities followed by ML bootstrap values are shown at nodes; branch lengths reflect estimated number of changes per site.

**Figure 7.**
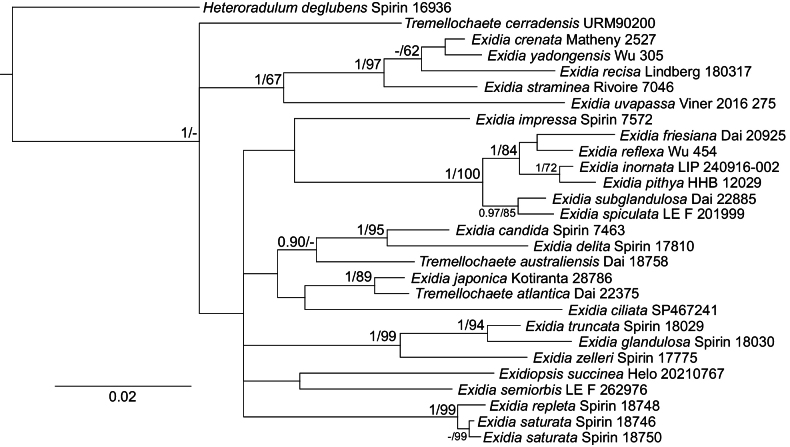
Phylogeny of the redefined *Exidia* inferred from the ITS + LSU concatenated dataset using BI analysis. Bayesian posterior probabilities followed by ML bootstrap values are shown at nodes; branch lengths reflect estimated number of changes per site.

**Figure 8.**
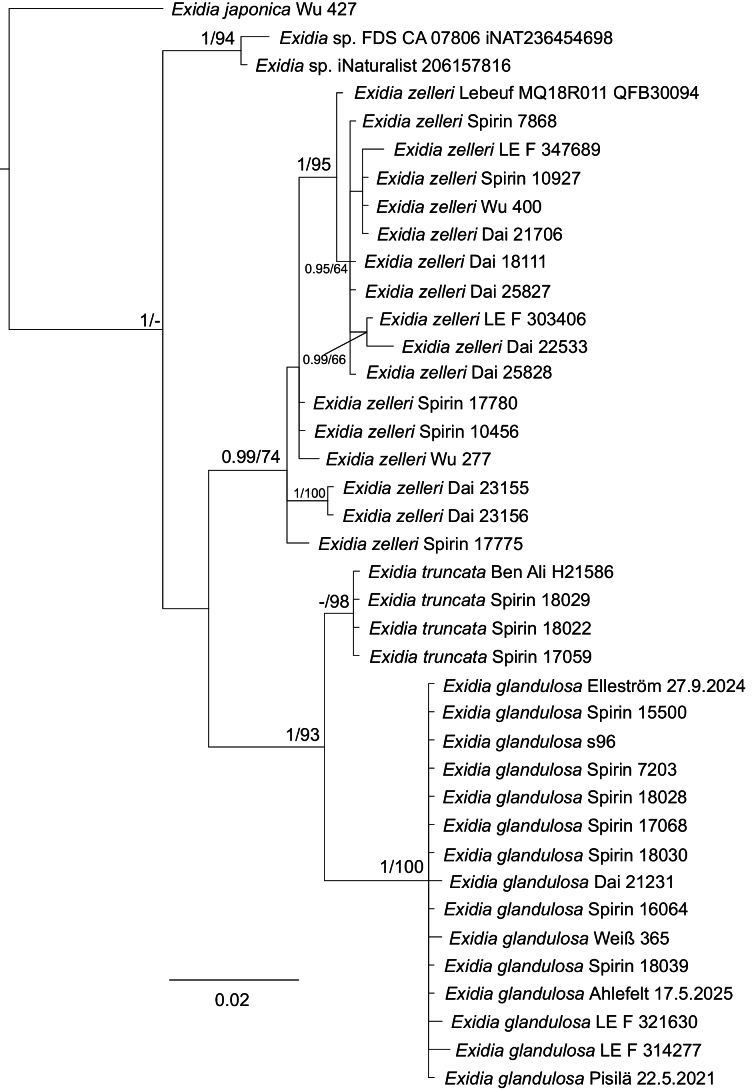
Phylogeny of the *Exidia glandulosa* complex inferred from the ITS dataset using BI analysis. Bayesian posterior probabilities followed by ML bootstrap values are shown at nodes; branch lengths reflect estimated number of changes per site.

**Figure 9.**
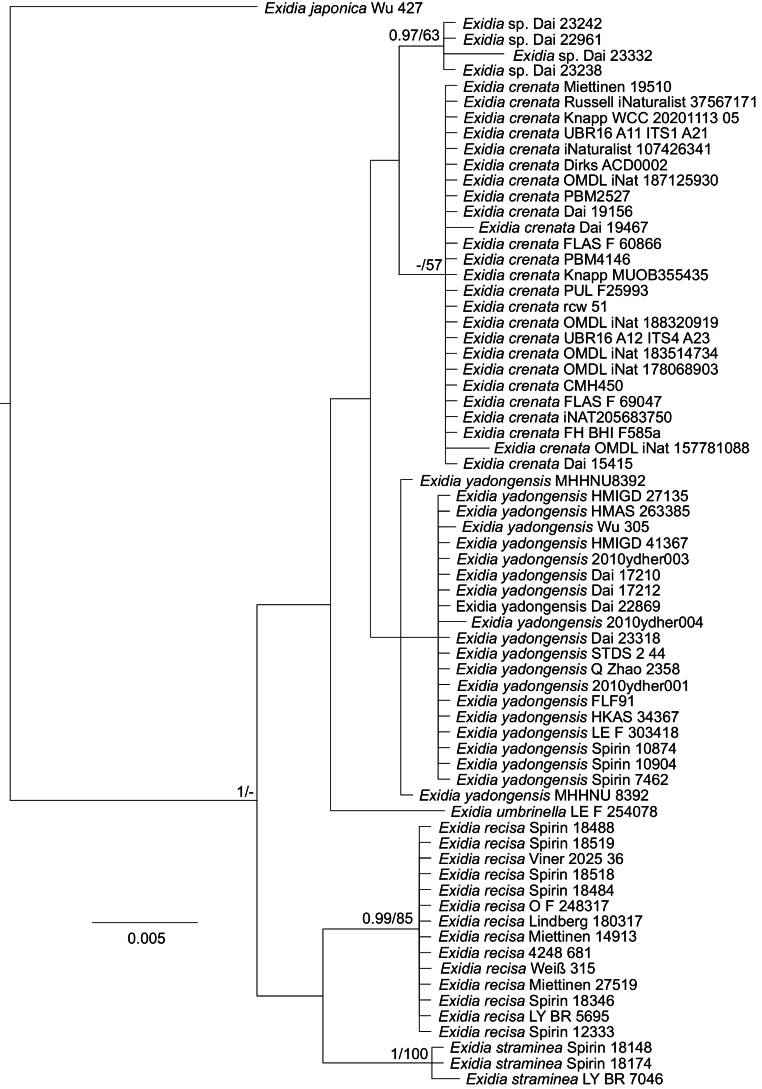
Phylogeny of the *Exidia recisa* complex inferred from the ITS dataset using BI analysis. Bayesian posterior probabilities followed by ML bootstrap values are shown at nodes; branch lengths reflect estimated number of changes per site.

**Figure 10.**
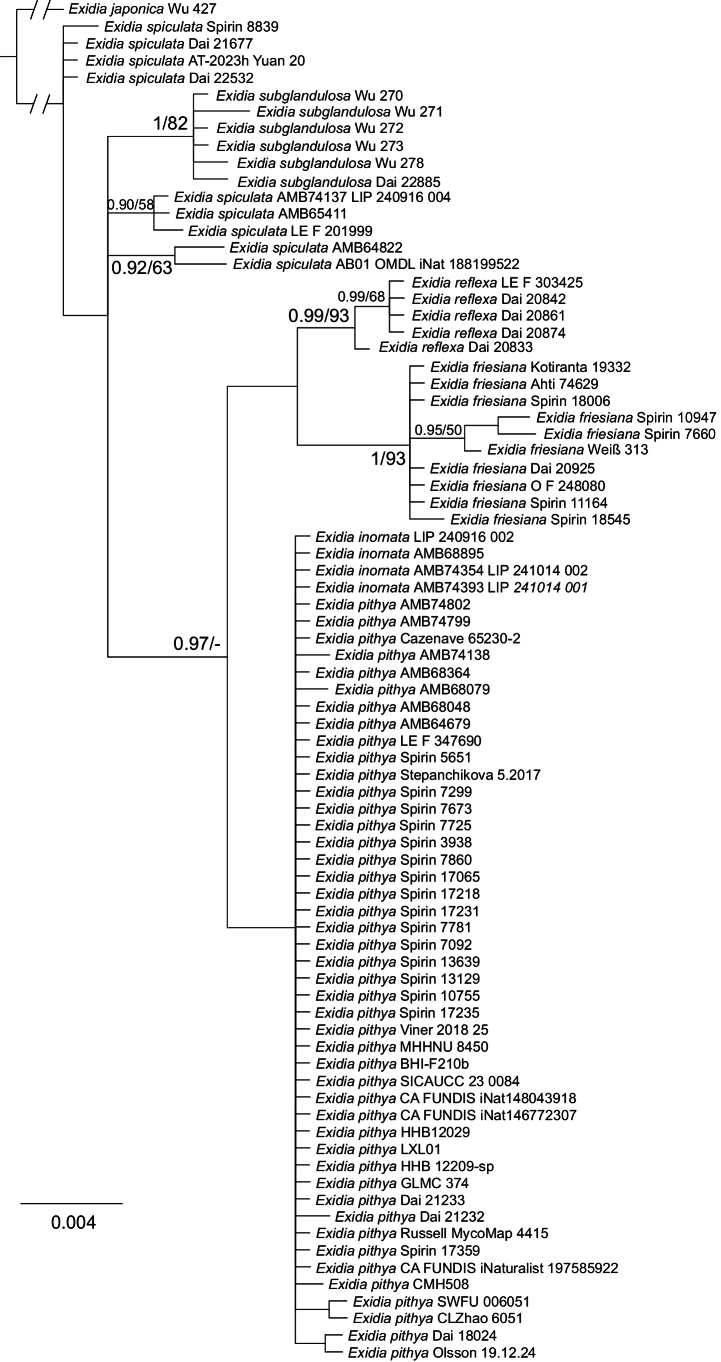
Phylogeny of the *Exidia pithya* complex inferred from the ITS dataset using BI analysis. Bayesian posterior probabilities followed by ML bootstrap values are shown at nodes; branch lengths reflect estimated number of changes per site.

**Figure 11.**
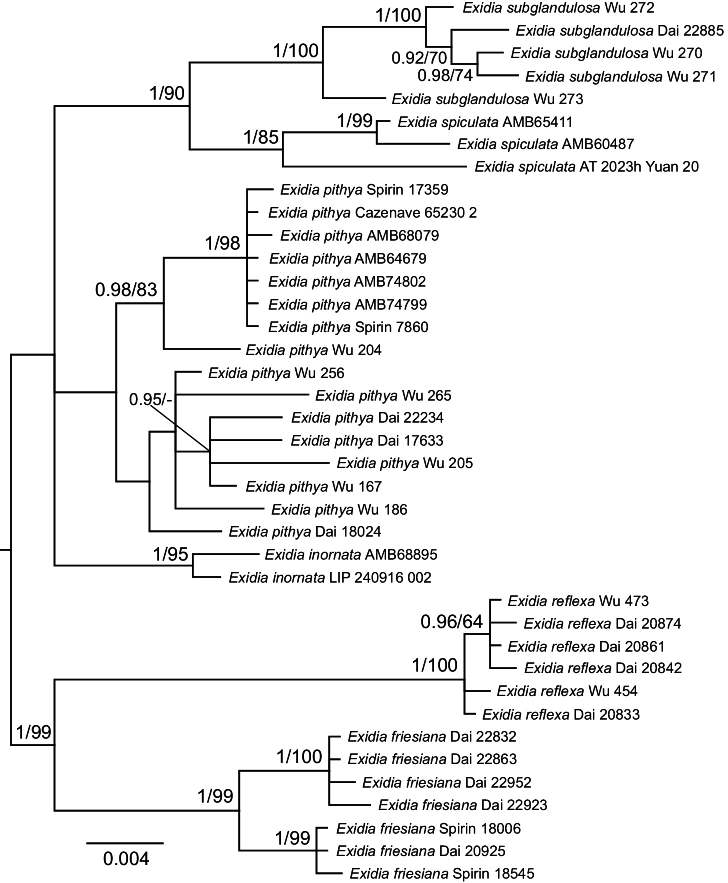
Phylogeny of the *Exidia pithya* complex inferred from the ITS + *TEF1* concatenated dataset using BI analysis. Bayesian posterior probabilities followed by ML bootstrap values are shown at nodes; branch lengths reflect estimated number of changes per site.

**Figure 12.**
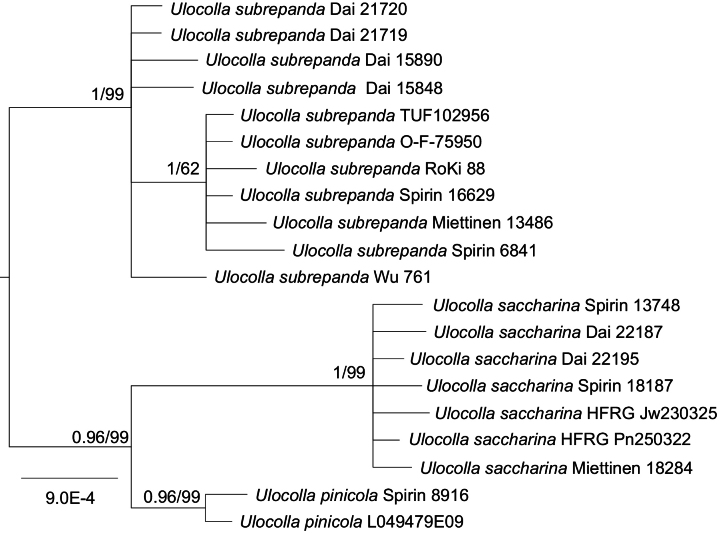
Phylogeny of the *Ulocolla saccharina* complex inferred from the ITS dataset using BI analysis. Bayesian posterior probabilities followed by ML bootstrap values are shown at nodes; branch lengths reflect estimated number of changes per site.

**Figure 13.**
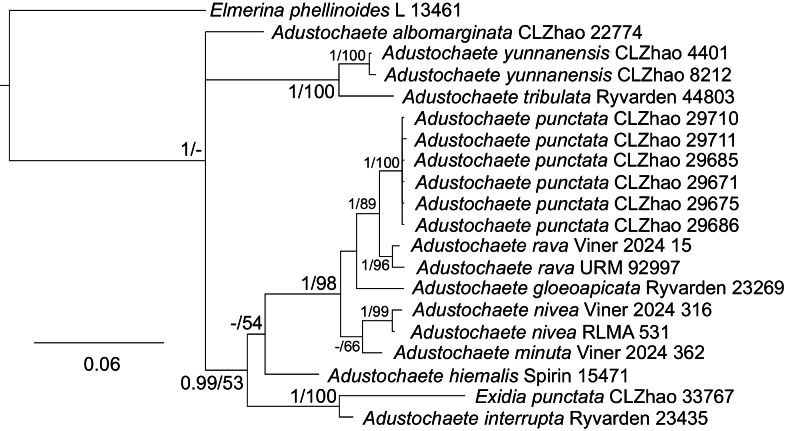
Phylogeny of *Adustochaete* inferred from the ITS + LSU concatenated dataset using BI analysis. Bayesian posterior probabilities followed by ML bootstrap values are shown at nodes; branch lengths reflect estimated number of changes per site.

**Figure 14.**
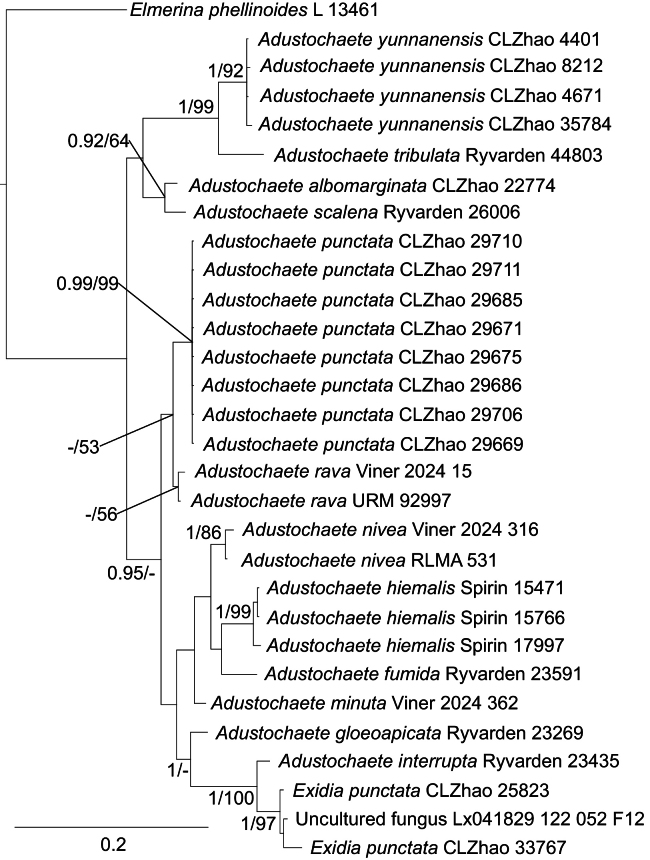
Phylogeny of *Adustochaete* inferred from the ITS dataset using BI analysis. Bayesian posterior probabilities followed by ML bootstrap values are shown at nodes; branch lengths reflect estimated number of changes per site.

1. *Proterochaete* lineage (pp = 0.98, bs = 65%). This group is represented by seven species in our four-marker phylogeny (Fig. 1); it gains a higher support (pp = 1) in the three-marker BI phylogeny (Fig. 2). Alongside with the generic type of *Proterochaete* Spirin & Malysheva, *Proterochaete adusta* (Burt) Spirin & Malysheva, it encompasses two species formerly assigned to *Exidiopsis*, viz. *Exidiopsis paniculata* K. Wells & Bandoni and *Exidiopsis mucedinea* (Pat.) K. Wells, as well as *Exidiopsis griseobrunnea* ssp. macrogyna K. Wells & Raitv. which we show not to be closely related to *Exidiopsis griseobrunnea* K. Wells & Raitv. *s. str*. (see below, under *Ulocolla / Sclerotrema* lineage). Furthermore, the recently described genus *Heterocorticium* S.H. He, T. Nie & Yue Li (Li et al. 2023) is a part of the redefined *Proterochaete* (Figs 3, 4). In total, eleven species are accepted in the genus, of which four are described as new, all but two being waxy or arid corticioid fungi with smooth (except for *P. adusta*) hymenium. Two species, *P. abstrusa* and *P. adusta*, have at least partly gelatinized basidiomata. Morphological redefinition of the genus is provided in the taxonomic section of the paper.
2. *Leiostroma* lineage (pp = 1, bs = 100%). It contains two newly described species, *L. infecundum* and *L. incanum*, both covered by the current concept of *Exidiopsis calcea* (Pers.) K. Wells (*sensu*Wells 1961). The latter species was placed by Liu et al. (2022) in their newly erected genus *Alloexidiopsis* L.W. Zhou & S.L. Liu. We argue below in favor of resurrecting an older generic name, *Leiostroma* Fr., for *E. calcea* sensu auct. and its closest relatives. As for the generic type of *Alloexidiopsis*, *Alloexidiopsis schistacea* L.W. Zhou & S.L. Liu, its identity and phylogenetic position deserve a closer look; at the moment, we have to leave this problem unsolved. In total, four accepted species are included in *Leiostroma* in the present study (Fig. 5, Suppl. material 4; see comments below). They all possess effused, arid, often easily crumbling basidiomata with white or gray, smooth (in one species spinulose) hymenial surface.
3. *Eichleriella / Amphistereum* lineage (pp = 1, bs = 100%). Two dimitic *Eichleriella* spp., *Eichleriella leveilleana* (Berk. & M.A. Curtis) Burt and *Eichleriella schrenkii* Burt, separated from the monomitic *Eichleriella* spp. in ITS – LSU phylogeny, were placed in the genus *Amphistereum* Spirin & Malysheva (Malysheva and Spirin 2017). Our current data show that *Amphistereum leveilleanum* (Berk. & M.A. Curtis) Spirin & Malysheva s. lato forms a strongly supported clade with other species of *Eichleriella* s. str. (Figs 1, 2). We therefore see merging *Amphistereum* with *Eichleriella* as a better taxonomic option than keeping them separate.
4. *Exidiopsis* s. str. / *Heteroradulum* lineage (pp = 1). In the four- and three-marker phylogenies (Figs 1, 2), *Exidiopsis effusa* (Bref.) Vuillemin (the generic type of *Exidiopsis*) and *Heteroradulum deglubens* (Berk. & Broome) Spirin & Malysheva (sibling species of *Heteroradulum kmetii* (Bres.) Spirin & Malysheva, the generic type of *Heteroradulum* Lloyd ex Spirin & Malysheva) cluster together but gain strong support only in BI analyses. We typify and redescribe *E. effusa* below and introduce another species in the genus, *E. perflua* (Fig. 6). As for the potential merging of *Exidiopsis* and *Heteroradulum* in one genus, we leave this question to future studies with a denser species sampling for this lineage than presented here.
5. *Exidiopsis peritricha* (*Tegmenticium*) lineage. Only one known species, *Exidiopsis peritricha* (Bourdot & Galzin) Sacc. & Trotter, belongs to this clade (Figs 1, 2, Suppl. material 5). Up to the present moment, it was treated as a synonym of *E. effusa*. We reinstate *E. peritricha* as a separate species and, owing to its isolated phylogenetic position, place it in a newly described genus, *Tegmenticium*.
6. *Exidia* s. str. lineage (pp = 1, bs = 67%). Our results confirm that most *Exidia* spp. previously assigned to this genus form a strongly supported lineage (Figs 1, 2, Suppl. material 4) and are therefore considered here as congeneric. Only *Exidia repanda* Fr., *Exidia thuretiana* (Lév.) Fr. and *Exidia saccharina* Fr. s. lato appear outside of *Exidia* s. str. (see below); their generic reclassification and morphological differences from the redefined *Exidia* are discussed in the taxonomic part of the paper. In the four-marker phylogeny, *Exidia* s. str. is covered by twelve species representing the main species complexes within the genus. The ITS + LSU phylogeny of the genus includes 26 species (Fig. 7). In the Taxonomy section, we clarify the identity of *Exidia glandulosa* (Bull.) Fr. which was misinterpreted as *Exidia truncata* Fr. in all earlier phylogenetic studies of the *Auriculariaceae*. *Exidia glandulosa*, the generic type of *Exidia*, forms a strongly supported subclade with the reinstated *E. truncata* and *Exidia zelleri* Lloyd (Figs 1, 2, 8, Suppl. material 5). The rest of species belong to another, much more species-rich subclade within the *Exidia* s. str. lineage. We specifically address the taxonomy of *Exidia pithya* Fr. and *Exidia recisa* (Ditm.) Fr. complexes in the corresponding ITS+ *TEF1*- and ITS-based phylogenies (Figs 9, 10, 11). Twenty-one *Exidia* species have adpressed-orbicular or pulvinate, normally brightly coloured, strongly gelatinous basidiomata and correspond well to the morphological definition of the genus originated from Brefeld (1888). Four *Exidia* species possess strictly resupinate, smooth basidiomata with adnate margins – characters aligned with the traditional concept of *Exidiopsis* (cf. Wells 1961). These species, *Exidiopsis succinea* K. Wells & Raitv. (= *Exidia plumbescens* below) and the newly described *Exidia delita*, *E. repleta*, and *E. saturata* do not form a joint clade within the *Exidia* lineage (Figs 1, 2, Suppl. material 4), thus pointing towards a potentially higher diversity among *Exidia* spp. with simplified, effused basidiomata yet to be discovered.
7. *Ulocolla / Sclerotrema* lineage (pp = 1, bs = 67%). This group is represented by four species in our multigene datasets, *Exidia saccharina* (the generic type of *Ulocolla* Bref.), *Exidiopsis griseobrunnea* (the generic type of *Sclerotrema* Spirin & Malysheva), *Exidiopsis grisea* (Bres.) Bourdot & Maire, and Sebacina calcea var. corticioides Malençon (reinstated below as an *Ulocolla* species) (Fig. 1, Suppl. material 5), and includes seven known species in total (Fig. 12, Suppl. material 5). Despite their quite divergent macroscopic habits, the members of the *Ulocolla* lineage share several important microscopic features discussed below. We therefore consider them as belonging to one genus, for which *Ulocolla* is the oldest available name.
8. *Exidia repanda* – *E. thuretiana* (*Descidia*) lineage (pp = 1, bs = 100%). The clade contains the two aforementioned species of *Exidia* s. lato which are phylogenetically not closely related to the rest of *Exidia* spp. A new genus, *Descidia*, is introduced below for *E. repanda* and *E. thuretiana*, and their differences from the similar-looking species of *Exidia* s. str. and *Ulocolla* are explained below.
9. *Adustochaete* lineage (pp = 1, bs = 100%). This group is represented by four species in our multigene datasets, including the generic type, *Adustochaete rava* R.L.M. Alvarenga & K.H. Larss. In total, thirteen species are assigned to the genus (Figs 13, 14). Of these, eight closely related species have spinulose hymenophore and constitute the core of the genus. They are more distantly related to three other species with smooth hymenophore (represented by *Adustochaete hiemalis* in Figs 1, 2, Suppl. material 5). Nevertheless, we refer these smooth species to *Adustochaete* R.L.M. Alvarenga & K.H. Larss . because of their great anatomical similarity to the spiny representatives of the genus and amend the original generic description accordingly.
10. *Elmerina / Aporpium* lineage (pp = 1, bs = 100%). As a monophyletic unit, this group has been recovered in several previous studies (Zhou and Dai 2013; Sotome et al. 2014; Malysheva et al. 2018). All but one species in this clade are poroid fungi. In our phylogeny, this group is represented by three species, *Elmerina phellinoides*, *Aporpium dimidiatum* A. David and *Aporpium canescens* (P. Karst.) Bondartsev & Singer. The generic division of this lineage has not been satisfactorily re-established yet and revising it is not directly connected to the tasks of the present study.

In general, equating the aforementioned eleven phylogenetic lineages of the *Exidia* clade to the taxonomic genera allows us to avoid two other, more extreme solutions. One of them, accepting the whole *Exidia* clade as one large genus, was already mentioned above. Another option would be to continue splitting the above-described lineages: for example, to divide *Exidia* into two genera, *Exidia* s. str. (limited to *E. glandulosa* complex) and *Tremellochaete* Raitv. (for the rest of species), or to separate the smooth *Adustochaete* spp. from the spinulose ones, or to retain *Proterochaete* only for the type species, *P. adusta*, and place others in *Heterocorticium* and two / three new genera, just to name a few. Although looking justified from both phylogenetic and, to some degree, morphological standpoints, this splitting scheme is profoundly dependent on overemphasizing one or two deliberately chosen morphological traits while ignoring others; the overemphasizing, which may largely be a result of our still very limited knowledge of the target group’s species diversity. Surely, the same could be said about the reclassification of the *Exidia* clade proposed above. However, considering that non-ribosomal markers are not yet available for the generic types of seven more existing genera from the *Exidia* clade (*Alloexidiopsis*, *Crystallodon* R.L.M. Alvarenga, *Heterochaete* s. str., *Hirneolina* (Pat.) Bres., *Metulochaete* R.L.M. Alvarenga, *Nodulochaete* J.H. Dong & C.L. Zhao, and *Punctochaete* J.H. Dong & C.L. Zhao) and they are therefore not represented in our family-level multigene phylogenies, we prefer not to multiply genera beyond necessity.

We additionally constructed a three-marker (ITS + LSU + *RPB2*) dataset encompassing 49 species of the *Auriculariaceae*, i.e. 46 species from the four-marker dataset plus *Eichleriella alliciens* (Berk. & Cooke) Burt, *Endoperplexa septocystidiata* (Hauerslev) P. Roberts, and *Scrupulispora perparvula* gen. nov. et sp. nov. The overall structure of the *Auriculariaceae* remains the same, showing four main splits, i.e. the single-species *Pholiobasidion* lineage, as well as *Auricularia*, *Exidia* and *Oliveonia* clades (Fig. 2). The *Oliveonia* clade is represented by *O. pauxilla* s. lato and *E. septocystidiata* clustering together with strong support (pp = 1, bs = 79%). Our yet unpublished data show that all residual *Endoperplexa* spp. are actually members of the *Oliveonia* clade and should be merged in one genus; we propose their reclassification in a separate paper. *Scrupulispora perparvula* clusters with *Oliveonia* – residual *Endoperplexa*, too, although it is on a long branch. This may be an indication that *Scrupulispora* is, in fact, a representative of its own lineage within the family and that its clustering with *Oliveonia* in our current phylogeny is a long branch attraction artifact. This question cannot be answered with our current taxon and genetic marker sampling. However, considering genetic distance of *Scrupulispora* from *Oliveonia* and other members of the *Auriculariaceae*, as well as its rather deviating morphology, we feel quite confident about introducing it as a new genus / species (see description below).

In general, the inner structure of the *Exidia* clade in the three-marker dataset mirrors that resulting from the four-marker analysis. There is, however, a significant drop in support values affecting four of ten lineages in the ML phylogeny (i.e. *Exidia* s. str., *Exidiopsis* s. str. */ Heteroradulum*, *Ulocolla / Sclerotrema*, and *Proterochaete*) (Fig. 2). In contrast, the *Proterochaete* lineage gains high support in the BI phylogeny (pp = 1).

Finally, thirteen species-level, ITS + LSU, ITS + *TEF1* or ITS-only phylogenies were reconstructed to assess species diversity in six genera of the *Auriculariaceae*. They included newly generated sequences, in addition to sequences obtained from GenBank and UNITE (including available unnamed environmental sequences).

1. *Proterochaete*. In total, eleven species are represented in the ITS + LSU phylogeny of *Proterochaete* (Fig. 3), including four species for which only ribosomal markers are available. These are *Heterocorticium bambusicola* S.H. He et al. and *Heterocorticium latisporum* S.H. He et al., combined below in *Proterochaete*, plus two new species from Africa, *P. africana* and *P. furfurosa*. The ITS dataset for *Proterochaete* encompasses 65 sequences of the eleven accepted species and one more, yet unnamed species from China (Fig. 4).
2. *Leiostroma*. In total, 22 sequences were included in the ITS phylogeny of *Leiostroma* (Suppl. material 4), thirteen newly obtained, and nine retrieved from GenBank. Only the newly described *L. squalidum* from Africa clearly shows up as a separate clade while three other species introduced below remain unresolved in the ITS-based tree. The main reason for this mixed picture is, in our opinion, the small differences in ITS region between *L. farinellum*, *L. incanum* and *L. infecundum*, combined with a rather high variation of ITS sequences of the first species. *Leiostroma incanum* and *L. infecundum* are included in the multigene phylogenies (Figs 1, 2) where they appear as separate species. Their genetic differences correspond to morphological and ecological data, as well as to their geographic distribution. Both species are well-supported in the combined ITS + *TEF1* phylogeny (Fig. 5). In the ITS region, *L. incanum* shows 5–6 bp difference from *L. farinellum* and 9–10 bp from *L. infecundum*; the difference of the latter species from *L. farinellum* is 5–8 bp. However, intraspecific variation of ITS sequences within *L. farinellum* approaches 9 bp. Eight sequences of *L. farinellum* from boreal and subalpine zones of Europe show 0–1 bp differences from each other, and they represent *L. farinellum* s. typi (see description and discussion below). Two specimens of *L. farinellum* from Europe (Slovenia and Sweden) were included in the ITS + *TEF1* phylogeny where they formed a strongly supported clade (Fig. 5). The only deviating European sequence so far available (GenBank OM104979) is from the Czech Republic; it differs from the rest of European *L. farinellum* sequences in 2 bp – either due to sequencing errors or intraspecific variation. No *TEF1* sequence from this collection is available. Two North American ITS sequences obtained by us show constant 6 bp difference from the European *L. farinellum*. One of them was included in the ITS + *TEF1* dataset where it showed up separate from *L. farinellum* (Fig. 5, as *Leiostroma* sp. 1). It is therefore possible that the North American collections belong to a sister species of *L. farinellum* distributed in North America; however, we failed to find any reliable morphological differences between the European and the North American material. Had they been shown to be a separate species, then the identity of *Sebacina monticola* Burt would need to be properly clarified. Four sequences identified as *E. calcea* by Liu et al. (2022) originating from China are not identical to each other; they show 5–8 bp vs. European and 3–8 bp vs. North American sequences of *L. farinellum*. Their identity deserves further clarification. One more putative *Leiostroma* species is represented by a single specimen from the Mediterranean (Fig. 5, Suppl. material 4 – as *Leiostroma* sp. 2).
3. *Exidiopsis* s. str. In the present study, we restrict *Exidiopsis* to two species: *E. effusa* and the newly described *E. perflua*. Differences in the ITS region between the two species are rather small, 5–6 bp only. They are, however, consistent with much larger differences (over 50 bp) in *RPB1* and *RPB2* regions and, to some extent, with morphology and geographic distribution. The ITS phylogeny of *Exidiopsis* s. str. (Fig. 6) includes twelve sequences (nine newly generated and three retrieved from GenBank) and is aimed mainly at presenting the distribution of the two species in Europe.
4. *Exidia* s. str. The ITS + LSU phylogeny of the genus includes 26 species (Fig. 7). Of these, twenty-three are accepted below as members of *Exidia*, including the generic type of *Tremellochaete*, *E. japonica*. Three other recently introduced *Tremellochaete* species, i.e. *T. atlantica*, *T. australiensis* and *T. cerradensis*, belong to *Exidia* in the current sense. Nevertheless, we are unwilling to combine them in *Exidia*, due to the existence of competing older names, all treated under excluded and insufficiently known taxa. Two other genuine *Exidia* species dealt with below, *E. umbrinella* Bres. and the newly described *E. tanzanica*, are represented by ITS sequences only; therefore, they were not included in the ITS + LSU phylogeny of the genus. *Exidia umbrinella* is a member of the *E. recisa* complex and its phylogenetic position is shown in the corresponding tree (see below). As for *E. tanzanica*, it is morphologically similar to other *Tremellochaete* species. Blasting its ITS sequence in GenBank gives *E. japonica* and *T. australiensis* as the closest hits (94–94.5% similarity), and we therefore assume that it belongs to *Exidia*. Four species-level phylogenies discussed in the following paragraphs are focused on selected species complexes within the redefined *Exidia*.
5. *Exidia glandulosa* complex. The ITS phylogeny (Fig. 8) indicates the presence of four species in this group, of which three, viz. the redefined *E. glandulosa* and *E. truncata*, as well as *E. zelleri*, are included in the four- and three-marker phylogenies (Figs 1, 2). The fourth, seemingly unnamed species is represented by two GenBank sequences ( PQ146076, PV455405), both obtained from specimens collected around San Francisco, California. ITS sequences of *E. zelleri* show considerable (up to 11 bp) intraspecific variation. They all, however, resolve in a strongly supported clade. Morphologically, all specimens of *E. zelleri* studied by us are highly uniform. Therefore, we argue here in favour of accepting them all as conspecific. Surely, the genetic variability of *E. zelleri* deserves a closer look in future studies.
6. *E. recisa* complex. The ITS phylogeny (Fig. 9) includes five accepted species, *E. crenata*, *E. recisa*, *E. straminea*, *E. umbrinella*, and *E. yadongensis*, plus three GenBank sequences which belong to two putative, yet-undescribed species from China. Two clades representing the sister species *E. crenata* and *E. yadongensis* receive moderate or no support in our phylogenies, evidently due to rather high variation in the ITS region in both species. However, their independent status was confirmed by Wu et al. (2020) with the use of additional (*RPB2*, *TEF1*) regions. For this reason, we accept *E. crenata* and *E. yadongensis* as separate species.
7. *E. pithya* complex. Our datasets include six morphologically definable species belonging to this group, i.e. the reinstated *E. friesiana* P. Karst., *E. pithya* (= *E. nigricans*) and *E. spiculata* Schwein. (= *E. inflata*), as well as the recently introduced *E. inornata* Cazenave & G. Gruhn, *E. reflexa* F. Wu et al. and *E. subglandulosa* F. Wu et al. (Ye et al. 2020; Gruhn et al. 2024). In the ITS-based phylogeny (Fig. 10), *E. friesiana*, *E. reflexa*, and *E. subglandulosa* are recovered as distinct lineages, while the rest form a large unresolved clade. The ITS + *TEF1* phylogeny shows all aforementioned species except for *E. pithya* to be phylogenetically supported (Fig. 11). It should be mentioned, however, that all accepted species possess significant intraspecific genetic variation and the use of additional markers would greatly supplement our species concepts in the *E. pithya* complex. Moreover, there is at least one case of conflict between ITS and *TEF1* sequences. The ITS sequence of the specimen Burdsall 12029 from the USA is identical to *E. pithya* sequences from Europe, while its *TEF1* sequence is identical to *E. friesiana*, not to *E. pithya* in the strict sense. We therefore discarded Burdsall 12029 from our ITS + *TEF1* dataset (see further discussion under *E. pithya*).
8. *Ulocolla*. The ITS-based phylogeny for *U. saccharina* complex encompasses sequences of three closely related species, *U. pinicola*, *U. saccharina*, and *U. subrepanda* (Fig. 12).
9. *Adustochaete*. The ITS + LSU phylogeny covers eleven species of the genus, including two species newly described below, *A. gloeoapicata* from Mexico and *A. tribulata* from Uganda (Fig. 13). The ITS phylogeny of *Adustochaete* includes two more new species from Africa, *A. fumida* and *A. scalena*, for which only ITS sequences are available (Fig. 14). Our data show that the recently described *Exidia punctata* L. Wang & C.L. Zhao from China is, in fact, a member of *Adustochaete* (Figs 13, 14); however, it cannot be transferred to the latter genus and is discussed under insufficiently known taxa below.

Finally, an ITS + LSU-based phylogeny of the whole *Exidia* clade was inferred (Suppl. material 5) with the same taxon sampling as the three-marker (ITS + LSU + *RPB2*) dataset and the addition of *Alloexidiopsis schistacea* (the generic type of *Alloexidiopsis*), two *Nodulochaete* species, *Punctochaete murina* (the generic type of *Punctochaete*), *Ulocolla orbiculata*, sp. nov., and two *Elmericium* spp., including the newly introduced *E. spinosum*. Only three genera out of eight accepted in this study (*Adustochaete*, *Eichleriella* and *Leiostroma*), as well as the *Elmerina* clade, are supported, while the representatives of other genera do not, in most cases, even cluster together. The ITS + LSU phylogeny is, therefore, of a very limited value for taxonomic conclusions and, in some cases, likely an artefact of long branch attraction. *Elmericium spinosum*, described below, forms a strongly supported clade with *E. alabastrinum* Spirin & Malysheva, the generic type of *Elmericium*; however, their phylogenetic position in the *Exidia* clade currently remains unclear.

### Taxonomy

#### Adustochaete

Taxon classificationAnimaliaAuricularialesAuriculariaceae

R.L.M. Alvarenga & K.H. Larss., Botany 97: 442, 2019

B0D6C74F-057B-5481-9C67-27677100FF9E

##### Description.

Basidiomata annual, effused, arid, nearly white to grayish or brownish. Hymenial surface smooth or spinulose. Hyphal structure monomitic; hyphae clamped, hyaline or brownish, spaced or partly agglutinated, basidia-bearing hyphae not differentiated. Crystals abundant, angular or sand-like. Cystidia as a rule present, clavate or tapering, in some species with brownish, cyanophilous contents (gloeocystidia). Hyphidia present, with a well-differentiated stem and richly branched, densely encrusted apical outgrowths. Basidia four-celled, ellipsoid-ovoid to nearly obconical, sessile, thin-walled or with a distinct wall, exposed or embedded, occasionally with a reduced stalk-like base. Basidiospores hyaline, thin-walled, narrowly to broadly cylindrical, curved. On dry or just fallen branches, more rarely on shrub stems of angiosperms.

##### Generic type.

*Adustochaete rava* R.L.M. Alvarenga & K.H. Larss.

*Adustochaete* was described to encompass two *Heterochaete*-like species, *A. rava* and *A. interrupta*. These species turned out to be phylogenetically distant from the generic type of *Heterochaete*, *H. andina*, as well as other *Heterochaete* s. lato species reclassified among *Eichleriella*, *Hirneolina*, *Heteroradulum*, and *Tremellochaete* (Alvarenga et al. 2019), and were therefore placed in their own genus. Subsequently, four more species were introduced in *Adustochaete*, viz. *A. nivea* from Brazil (Hyde et al. 2020), as well as *A. yunnanensis*, *A. albomarginata*, and *A. punctata* from China (Li and Zhao 2022; Dong et al. 2024). Here we add six more species to the genus and reintroduce *A. rava* and *A. nivea* based on more extensive material from Argentina (for the species-level phylogeny of the genus, see Figs 13, 14). See also remarks on *Exidia punctata* under Excluded and insufficiently known species below.

Although *Adustochaete* spp. form a strongly supported clade (Figs 1, 2) and are clearly separated from the rest of the genera in the *Exidia* clade in our phylogenies, the morphological definition of *Adustochaete* is not easy. Originally, the genus was defined as containing monomitic, cystidia-bearing species with grayish or brownish, spinulose or tuberculate hymenial surface and four-celled, ellipsoid-ovoid or nearly obconical basidia which possess thick sterigmata (Alvarenga et al. 2019). However, expanding *Adustochaete* with more species described makes it difficult to maintain the original concept of the genus. In particular, adding four completely or nearly smooth species (*A. fumida*, *A. gloeoapicata*, *A. hiemalis*, and *A. scalena*) blurs the borders between *Adustochaete* and some other genera containing the former *Exidiopsis* and *Heterochaete* spp.

Of them, the reinstated *Leiostroma* is strongly reminiscent of *Adustochaete*. Both genera include resupinate species with spinulose or smooth, grayish or brownish, easily crumbling, arid basidiomata and richly encrusted hyphae. Moreover, their hyphae and hymenial cells are sometimes brownish, evidently due to the color of the cytoplasm. The contents of these coloured cells clearly stain in CB (cyanophilous) in *Adustochaete* spp. while they normally stay unchanged or show only a weak cyanophilous reaction in *Leiostroma*. Another trait separating the two genera is the crystalline incrustation on hyphae. Species of both genera produce abundant, small, angular or sand-like (fine-grained) crystals; these crystals, however, mostly remain discrete in *Adustochaete* and often fuse together and produce continuous shields, especially on basal hyphae and apical branches of hyphidia, in *Leiostroma*. Furthermore, *Leiostroma* spp. have well-differentiated basidia-bearing hyphae which are as a rule wider than the surrounding subhymenial hyphae; no such differentiation was observed in the *Adustochaete* spp. studied by us.

Differences between *Adustochaete* and another similar-looking genus, *Proterochaete*, are more obvious. *Proterochaete* spp. possess tougher basidiomata than those of *Adustochaete*, which consist of densely arranged, partly glued hyphae. Along with angular crystals, hyphae and hymenial cells of many *Proterochaete* spp. also bear brownish, cyanophilous grains fusing in large amorphous bodies in mature and especially senescent basidiomata. See further remarks under *Adustochaete hiemalis*.

#### Adustochaete fumida

Taxon classificationAnimaliaAuricularialesAuriculariaceae

Spirin & Ryvarden sp. nov.

8216B8D6-441E-582F-8C4A-6246820A53C0

862464

Fig. 15A

##### Holotype.

Zimbabwe • Masvingo: Chiredzi, Tambuti Lodge, corticated angiosperm branch, 17 Feb 1986, Ryvarden 23591* (O, isotype – H).

**Figure 15.**
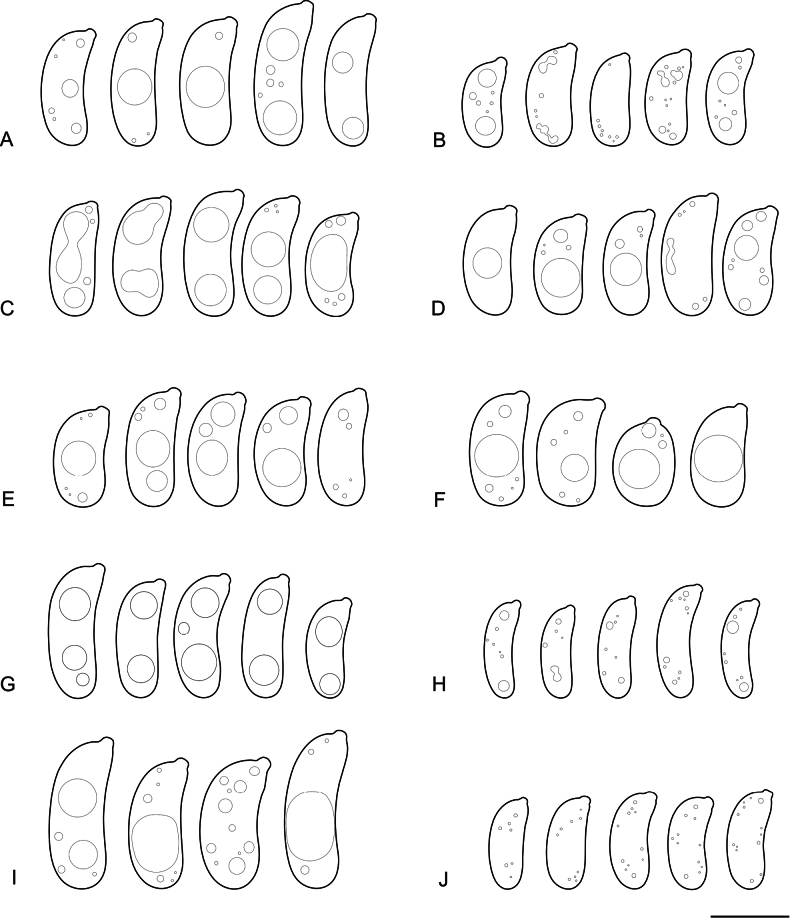
Basidiospores: **A***Adustochaete fumida* (holotype); **B***A. gloeoapicata* (holotype); **C***A. hiemalis* (holotype); **D***A. minuta* (Viner 2024/362); **E***A. nivea* (Viner 2024/316); **F***A. scalena* (holotype); **G***A. tribulata* (holotype); **H***Descidia repanda* (neotype); **I***D. thuretiana* (Spirin 17055); **J***Elmericium spinosum* (holotype). Scale bar: 10 μm.

##### Etymology.

Fumidus (Lat., adj.) – ash-gray.

##### Description.

Basidiomata annual, effused, up to 5 cm in the longest dimension, arid, rather soft, 0.1–0.2 mm thick. Hymenial surface smooth, cream-colored to grayish, occasionally cracking. Margin sharply delimited, concolourous with hymenial surface, adnate, up to 0.5 mm wide.

Hyphal structure monomitic, hyphae clamped; subicular hyphae hyaline or brownish, slightly to distinctly thick-walled, interwoven or subparallel, rather tightly arranged, 2–6 μm in diam., subhymenial hyphae predominantly hyaline, thin- to slightly thick-walled, ascending or interwoven, rather densely arranged and partly agglutinated, occasionally twisted, 2–4 μm in diam. Crystals abundant in all parts of basidiomata, encrusting hyphae, small, angular to sand-like. Cystidia very rare, thin-walled, single tubular-clavate or tapering and more or less distinctly swollen at the base, 20–23 × 5–8 μm, contents hyaline or grayish, CB+. Hyphidia hyaline, thin-walled, single or in groups of 3–4, with subfusiform or hyphoid stem, 18–42 × 2.5–5 μm, bearing several richly branched apical outgrowths, 0.5–1.2 μm in diam. at the very apex; apical branches encrusted by small, densely arranged crystals. Basidia four-celled, narrowly ellipsoid-ovoid to obconical, sessile, (15.5–) 18–25 (–26.5) × (8.9–) 9.0–12.0 (–12.5) μm (n = 25/1), thin-walled or with a distinct wall, usually embedded, occasionally with a reduced stalk-like base, up to 3 × 5 μm, sterigmata up to 30 × 3–4 μm. Basidiospores thin-walled, cylindrical, more or less clearly curved, (11.8–) 12.1–18.1 (–18.2) × (5.1–) 5.2–6.3 (–6.4) μm (n = 30/1), L = 15.96, W = 5.73, Q = 2.79.

##### Remarks.

This species is introduced here based on a single collection from Zimbabwe. *Adustochaete fumida* produces completely smooth basidiomata on thin branches of angiosperms. It differs from *A. scalena*, another smooth species from the same geographic area, in having lighter-coloured basidiomata and smaller basidia, as well as longer and distinctly narrower basidiospores. Microscopically, *A. fumida* is most similar to *A. hiemalis* from Europe. The latter species produces smaller, nearly white basidiomata on tiny plant remnants and its cystidia and basidia are larger than in *A. fumida*.

#### Adustochaete gloeoapicata

Taxon classificationAnimaliaAuricularialesAuriculariaceae

Spirin & Malysheva sp. nov.

6C109797-D84E-5511-A6E4-EF002C31A16D

862465

Fig. 15B

##### Holotype.

Mexico • Veracruz: Xalapa, Botanical Garden, dead corticated branches, 22 Sep 1985, Ryvarden 23269* (O, isotypes – H, LE).

##### Etymology.

Gloeoapicatus (Lat., adj.) – in reference to a resinous apical globule at the top of cystidia.

##### Description.

Basidiomata annual, effused, up to 10 cm in the longest dimension, arid, rather soft, 0.1–0.2 mm thick. Hymenial surface smooth, cream-colored to grayish, rarely irregularly cracking. Margin sharply delimited, first nearly white, then concolourous with hymenial surface, adnate, up to 0.5 mm wide.

Hyphal structure monomitic, hyphae clamped; subicular hyphae hyaline or brownish, thin- to slightly thick-walled, subparallel, partly agglutinated, 2–5 μm in diam., subhymenial hyphae hyaline or brownish, thin-walled, interwoven, rather densely arranged and partly agglutinated, 1.5–4 μm in diam. Crystals abundant in all parts of basidiomata, encrusting hyphae and hymenial cells, small, angular to sand-like. Cystidia (gloeocystidia) abundant, thin- to distinctly thick-walled, single or in groups of 3–4, more or less clearly tapering, often distinctly swollen at the base, 22–52 × 6–14 μm, contents brownish to grayish-brown, strongly CB+; some cystidia bearing resinous-brown, CB+ apical globule, up to 15 μm in diam. Hyphidia hyaline or more rarely brownish, thin- to slightly thick-walled, single or in groups of 3–4, with subfusiform or hyphoid stem, 17–34 × 3–5 μm, bearing several richly branched apical outgrowths, 1–1.5 μm in diam. at the very apex; apical branches encrusted by small, densely arranged crystals. Basidia four-celled, narrowly ellipsoid-ovoid to nearly obconical, sessile, (14–) 14.5–20 (–21) × (8.4–) 9.0–11.0 (–11.2) μm (n = 20/2), thin-walled, mostly embedded, occasionally with a reduced stalk-like base, up to 3 × 3 μm, sterigmata up to 20 × 3–3.5 μm. Basidiospores thin-walled, broadly cylindrical to cylindrical, slightly or moderately curved, (10.0–) 10.2–14.1 (–14.3) × (4.8–) 5.0–6.2 (–6.3) μm (n = 60/2), L = 11.68–12.63, W = 5.51–5.53, Q = 2.12–2.29.

##### Remarks.

*Adustochaete gloeoapicata* is described here as the single smooth species of the genus known to occur in North America. It has so far only been collected twice in the same locality in Veracruz, Mexico. *Adustochaete gloeoapicata* differs from other smooth species of the genus in having large, basally swollen gloeocystidia which sometimes bear an apical resinous globule. No such cystidia are known to us for any other representative of the *Auriculariales*.

#### Adustochaete hiemalis

Taxon classificationAnimaliaAuricularialesAuriculariaceae

Spirin sp. nov.

DFC42192-5F7E-5511-86E7-B465E65644F2

862466

Figs 15C, 16A, 17

##### Holotype.

Sweden • Västergötland: Mölndal, Snöhöjdsliden, 57.67614;11.98129, *Rubus fruticosus* s. lato (dead stem on the ground), 16 Dec 2024, Spirin 17997* (GB, isotype – H).

**Figure 16.**
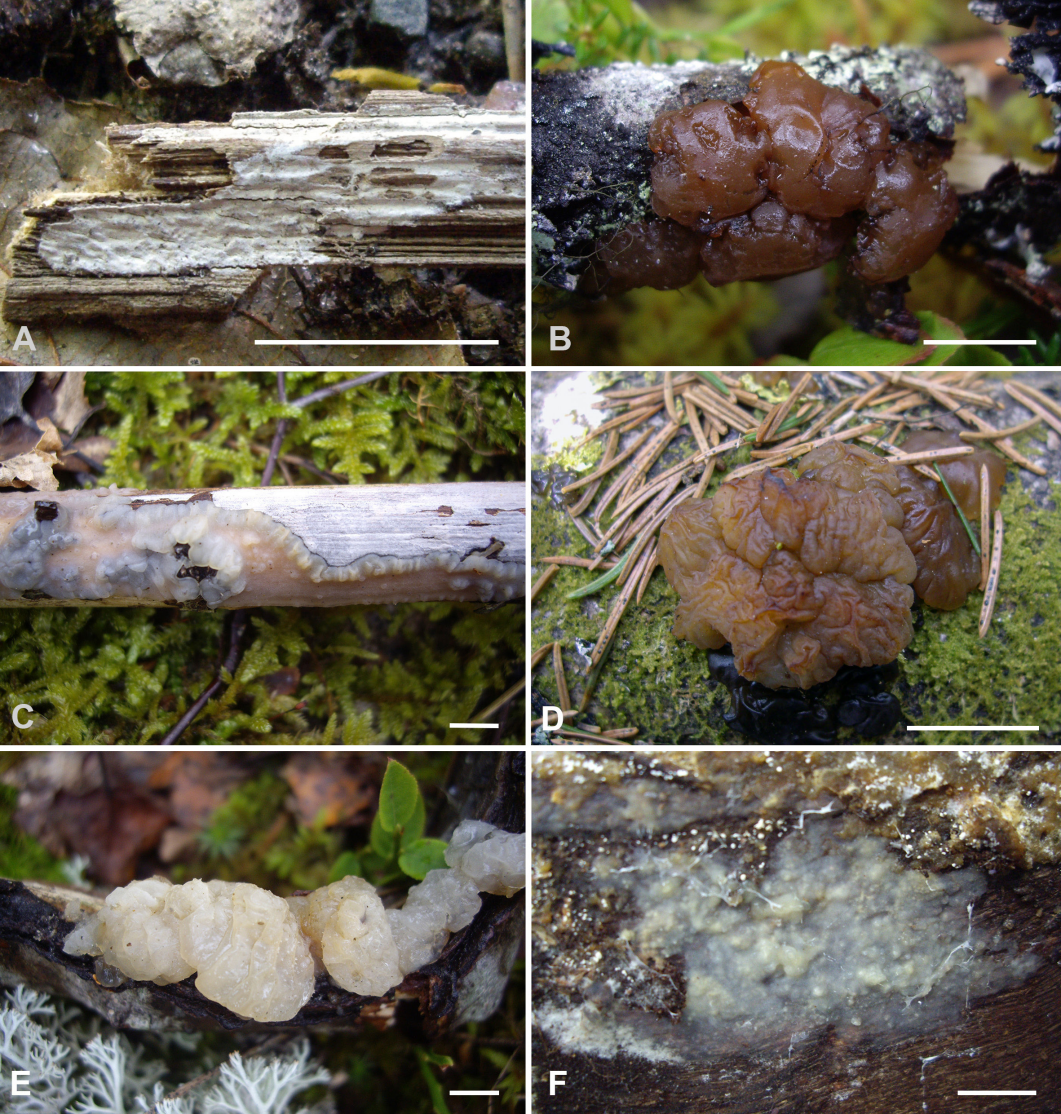
Basidiomata: **A***Adustochaete hiemalis* (holotype); **B***Descidia repanda* (Spirin 14193); **C***D. thuretiana* (Spirin 17055); **D**Exidia candida var. cartilaginea, sitting at the top of *E. pithya* (Spirin 11221); **E***E. candida* s. str. (Spirin 17471); **F***E. delita* (holotype). Scale bar: 1 cm.

##### Etymology.

Hiemalis (Lat., adj.) – wintry, hibernal.

##### Description.

Basidiomata annual, effused, patch-like, usually very small, a few mm in diam., rarely larger, up to 1 cm in the widest dimension, arid, pellicular and very soft, 0.05–0.2 mm thick, finally degrading to a white crumbling mass. Hymenial surface smooth, nearly white or cream-colored. Margin first pruinose, adnate, then rather sharply delimited and partly detaching, white, up to 0.5 mm wide.

**Figure 17.**
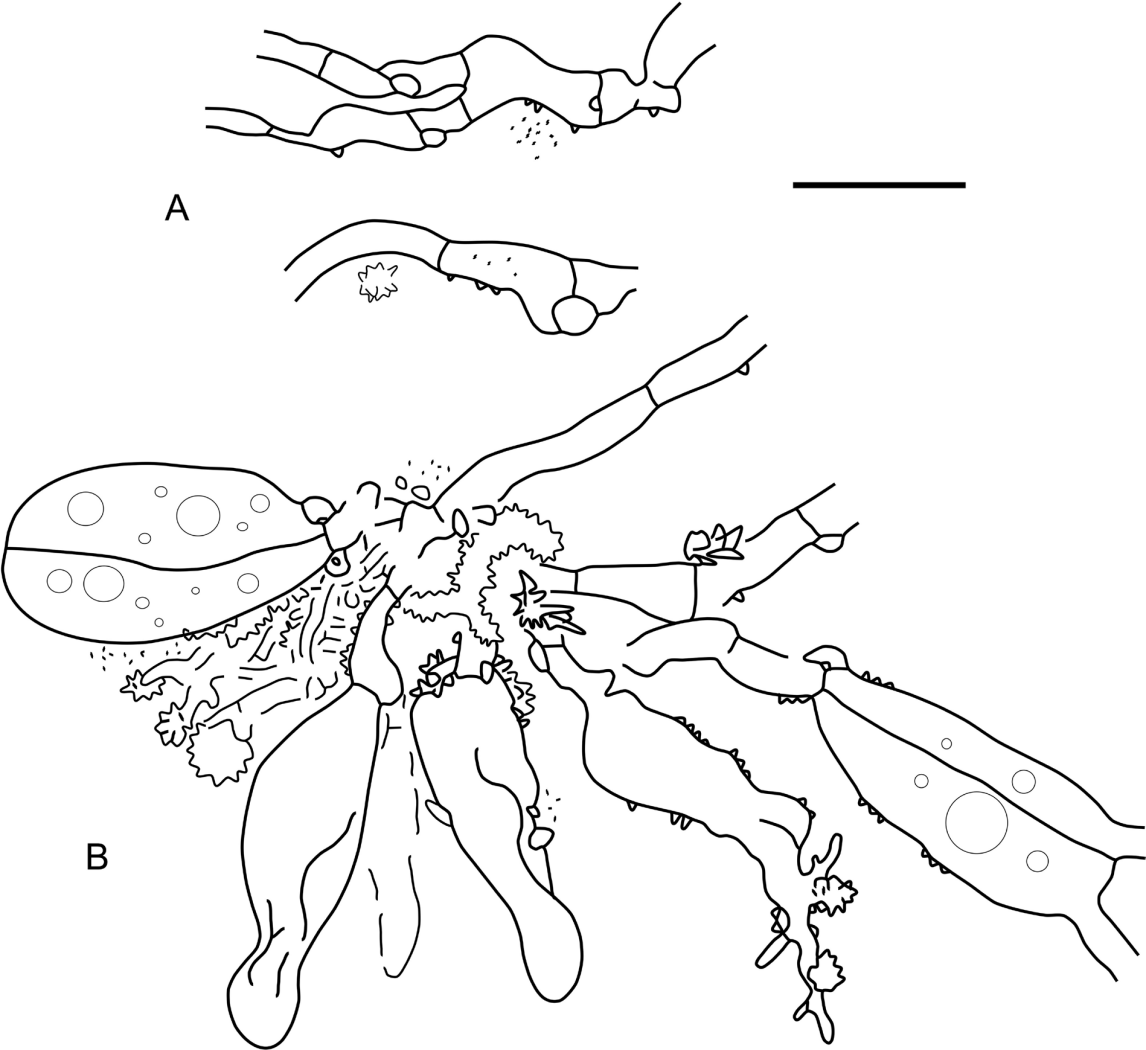
Microstructures of *Adustochaete hiemalis* (holotype): **A** subicular hyphae; **B** subhymenial hyphae and hymenial cells. Scale bar: 10 μm.

Hyphal structure monomitic, hyphae clamped; subicular hyphae hyaline, thin- to slightly thick-walled, loosely interwoven or subparallel, 1.5–4 μm in diam., frequently anastomosing, subhymenial hyphae hyaline, thin- to slightly thick-walled, ascending or interwoven, spaced, 1–4 μm in diam. Crystals abundant in all parts of basidiomata, often encrusting hyphae, small, angular to sand-like. Cystidia occasionally present, hyaline, thin-walled, single or in groups of 2–3, distinctly tapering or clavate, 18–57 × 5–12.5 μm, some with grayish-brown, distinctly CB+ contents. Hyphidia thin- to slightly thick-walled, often in groups of 2–4, with a cystidia-like, narrowly fusiform or hyphoid, hyaline or brownish stem, 20–40 × 2–8 μm, bearing several richly branched apical outgrowths, 1–2 μm in diam. at the very apex; apical branches encrusted by small, densely arranged crystals. Basidia four-celled, ellipsoid-ovoid to nearly obconical, sessile, (16–) 17–31 (–32) × (9.3–) 9.6–13.5 (–13.6) μm (n = 50/3), often with a distinct wall, embedded or exposed, rarely with a reduced stalk-like base, up to 4 × 3 μm, sterigmata up to 27 × 3–3.5 μm. Basidiospores thin-walled, broadly cylindrical to cylindrical, more or less clearly curved, (9.2–) 9.8–17.8 (–18.4) × (4.8–) 4.9–6.4 (–6.8) μm (n = 65/3), L = 12.90–15.00, W = 5.68–5.88, Q = 2.27–2.64.

##### Remarks.

*Adustochaete hiemalis* is the only representative of the genus known to occur in Europe. It produces minuscule, whitish, smooth basidiomata on thin dead stems of shrubs (*Lavandula*, *Rubus*) and lianas (*Clematis*) and, according to our observations, fruits from December to June. Morphologically, it is very similar to other smooth *Adustochaete* spp. known from tropical areas. One specimen of *A. hiemalis* was collected from a *Lavandula* stem which it occupied together with *Proterochaete letendreana*. The latter species occasionally produces nearly white, small basidiomata on shrub or grass stems which are superficially very similar to *A. hiemalis*. In these cases, the species can be recognized after careful morphological study. Basidiomata of *A. hiemalis* are softer than those of *P. letendreana*, and they consist of rather loosely arranged, spaced (not agglutinated) hyphae. Moreover, *A. hiemalis* possesses cystidia and its basidia are larger than in *P. letendreana*. Basidiospores of both species are nearly identical and quite variable in size. See further remarks under *P. letendreana*.

*Leiostroma farinellum* rarely occurs on thin angiosperm branches and can be mistaken for *A. hiemalis*, too. It can be separated from the latter species due to grayish basidiomata, the lack of cystidia, smaller basidia and more extensive, sometimes solid crystalline encrustation on hyphae and hymenial cells.

#### Adustochaete minuta

Taxon classificationAnimaliaAuricularialesAuriculariaceae

(Pat.) Spirin & Viner comb. nov.

9725B022-BF90-5AFA-8723-674317E7B365

862467

Fig. 15D

= Heterochaete minuta Pat., Bulletin de la Société Mycologique de France 9: 139, 1893. Holotype. Ecuador. Pichincha: Quito, Pululahua, twigs on the ground, Feb 1892, Lagerheim (FH).

##### Description.

Basidiomata annual, effused, up to 5 cm in the longest dimension, arid, rather soft, 0.05–0.1 mm thick. Hymenial surface spinulose, grayish, spines irregularly or evenly distributed, sharp-pointed, 70–150 × 30–60 μm, 6–8 (9) per mm. Margin floccose, clearly delimited, first white, then grayish, adnate, up to 0.5 mm wide.

Hyphal structure monomitic, hyphae clamped; subicular hyphae hyaline, slightly to distinctly thick-walled, interwoven or subparallel, rather loosely arranged, 1.5–4 μm in diam., subhymenial hyphae hyaline, thin-walled, interwoven, 1.5–3 μm in diam., hyphae of hymenial spines hyaline or brownish, thin- to slightly thick-walled, ascending, in older parts agglutinated and cemented by amorphous brown substance, CB (+) to CB+, 1.5–3 μm in diam. Crystals abundant in all parts of basidiomata, encrusting hyphae and hymenial cells, small, angular to sand-like. Cystidia rare, hyaline, thin-walled, more or less clearly tapering, often sinuous, more rarely clavate, 17–30 × 4.5–9 μm. Hyphidia hyaline, thin- to slightly thick-walled, with a hyphoid or subfusiform-clavate stem, 15–34 × 2–6 μm, bearing several richly branched apical outgrowths, 1–1.5 μm in diam. at the very apex; apical branches covered by continuous crystalline encrustation. Basidia four-celled, ellipsoid-ovoid to nearly obconical, sessile, (14–) 14.5–20 (–21.5) × (9.0–) 9.2–12.4 (–13.3) μm (n = 50/4), with a distinct wall, mostly exposed, rarely with a reduced stalk-like base, up to 4 × 3 μm, sterigmata up to 20 × 3–3.5 μm. Basidiospores thin-walled, broadly cylindrical to cylindrical, more rarely narrowly cylindrical, often clearly curved, (10.3–) 10.7–18.1 (–18.2) × (5.0–) 5.1–6.3 (–6.7) μm (n = 120/4), L = 13.18–15.28, W = 5.62–5.86, Q = 2.27–2.73.

##### Remarks.

This species was originally described as *Heterochaete minuta* by Patouillard and Lagerheim (1893) from Pululahua, Ecuador. It was subsequently redescribed by Bodman (1952), and her reintroduction of *H. minuta* fits well with three specimens from Argentina and Mexico studied by us. The only discordant feature mentioned in the protologue is the longer spines, measured as 200–450 μm long, i.e. clearly longer than in all our collections. Currently, it is impossible to determine whether *H. minuta* s. typi represents a similar species not conspecific with our material or the type material is a more developed specimen than those studied by us. This question can be answered only after getting sequenced material from the type locality. Morphologically and phylogenetically, *A. minuta* is very close to *A. nivea* (see below). It differs from the latter species in having smaller cystidia, as well as longer and slightly narrower basidiospores.

#### Adustochaete nivea

Taxon classificationAnimaliaAuricularialesAuriculariaceae

R.L.M. Alvarenga in Hyde et al., Fungal Diversity 100: 255, 2020

D99900F8-0113-563E-9B0C-8A4F7609E136

Fig. 15E

##### Holotype.

Brazil • Pernambuco: Recife, Federal University of Pernambuco, 2018, Alvarenga 531 (URM 93408*).

##### Description.

Basidiomata annual, effused, up to 5 cm in the longest dimension, arid, rather soft, 0.05–0.1 mm thick. Hymenial surface spinulose, cream-colored to grayish, spines rather regularly distributed, sharp-pointed, 100–200 × 35–60 μm, 5–7 per mm. Margin pruinose, white to cream-coloured, adnate, up to 0.5 mm wide.

Hyphal structure monomitic, hyphae clamped; subicular hyphae hyaline, slightly to distinctly thick-walled, variably inflated, interwoven or subparallel, loosely arranged, 1.5–4.5 μm in diam., subhymenial hyphae hyaline, thin- to distinctly thick-walled, interwoven, rather loosely arranged, 1–3 μm in diam., hyphae of hymenial spines hyaline, thin- to slightly thick-walled, ascending, 1.5–3 μm in diam. Crystals abundant in all parts of basidiomata, encrusting hyphae and hymenial cells, small, angular to sand-like. Cystidia occasionally present, hyaline or grayish-brownish, thin- to distinctly thick-walled, more or less clearly tapering, often sinuous, 21–40 × 5.5–10 μm. Hyphidia hyaline, thin- to slightly thick-walled, with hyphoid, occasionally branched stem, 20–44 × 3.5–5.5 μm, bearing several richly branched apical outgrowths, 1–1.5 μm in diam. at the very apex; apical branches richly encrusted by small, densely arranged crystals. Basidia four-celled, ellipsoid-ovoid to nearly obconical, sessile, (14–) 14.5–18.5 (–19) × (9.1–) 9.2–12.0 (–12.2) μm (n = 30/2), thin-walled, embedded or exposed, sterigmata up to 30 × 3–3.5 μm. Basidiospores thin-walled, broadly cylindrical to cylindrical, clearly curved, (10.2–) 10.3–15.2 (–15.9) × (5.0–) 5.1–6.8 (–7.2) μm (n = 64/2), L = 12.01–13.12, W = 5.93–6.02, Q = 2.03–2.18.

##### Remarks.

Here we reintroduce *A. nivea* based on our specimens from Argentina and Mexico. The sequence of the Argentinian collection is identical to that of the holotype from Brazil (Hyde et al. 2020). However, the protologue describes *A. nivea* as an acystidiate species with shorter and narrower basidiospores. More sequenced specimens of *A. nivea* are needed to understand its intraspecific variation. As redescribed here, *A. nivea* is a close relative of *A. minuta*. Their morphological differences are mentioned under the latter species.

#### Adustochaete rava

Taxon classificationAnimaliaAuricularialesAuriculariaceae

R.L.M. Alvarenga & K.H. Larss., Botany 97: 442, 2019

2B1EF7B3-79C4-5918-96BD-15512E2C4CAA

##### Holotype.

Brazil • Rondônia: Porto Velho, São Francisco Farm, deciduous wood, 15 Mar 2012, Larsson 15526* (URM 92997, isotype – H, studied).

##### Description.

Basidiomata annual, effused, up to 5 cm in the longest dimension, waxy-arid, rather soft, 0.05–0.2 mm thick. Hymenial surface spinulose, grayish to gray or pale grayish-brown, in older parts with small dark-brown spots, spines rather regularly distributed, sharp-pointed, 75–200 × 35–40 μm, 7–8 per mm. Margin first arachnoid to fimbriate, adnate, then sharply delimited and somewhat elevated, white, up to 1 mm wide.

Hyphal structure monomitic, hyphae clamped; subicular hyphae hyaline or brownish, thin- to slightly thick-walled, interwoven or subparallel, loosely arranged, in older parts agglutinated, 1.5–4 μm in diam., subhymenial hyphae hyaline or rarely brownish, thin-walled or with a distinct wall, ascending or interwoven, rather densely arranged and partly agglutinated, 1.5–3 μm in diam., hyphae of hymenial spines hyaline or more often brownish, slightly thick-walled, ascending or interwoven, 1.5–3 μm in diam., occasionally cemented by brownish amorphous substance. Crystals abundant in all parts of basidiomata, encrusting hyphae and hymenial cells, small, angular to sand-like. Cystidia abundant to rather rare, hyaline, thin- to slightly thick-walled, clavate or more or less clearly tapering, often sinuous, 21–52 × 4–8 μm. Hyphidia hyaline or brownish, thin- to slightly thick-walled, with hyphoid or narrowly clavate, occasionally branched stem, 20–40 × 2–5 μm, bearing several richly branched apical outgrowths, 1–1.5 μm in diam. at the very apex; apical branches densely encrusted by small, densely arranged crystals. Basidia four-celled, ellipsoid-ovoid to nearly obconical, sessile, (9.5–) 11–16 (–16.5) × (7.2–) 7.3–10.5 (–11.2) μm (n = 30/2), thin-walled or with a distinct wall, exposed or embedded, occasionally with a reduced stalk-like base, up to 4 × 4 μm, sterigmata up to 18 × 3–4 μm. Basidiospores thin-walled, narrowly cylindrical to cylindrical, clearly curved, (10.1–) 10.2–13.7 (–14.2) × (3.7–) 3.8–5.1 (–5.2) μm (n = 60/2), L = 11.83–12.01, W = 4.13–4.55, Q = 2.62–2.91.

##### Remarks.

*Adustochaete rava* was described based on the single collection from Rondônia, Brazil (Alvarenga et al. 2019). Here we supplement its description and report it from Argentina. Morphologically, *A. rava* can be easily distinguished from other neotropical *Adustochaete* spp. due to brownish tints of its basidiomata, as well as narrow basidiospores. The species was illustrated in Alvarenga et al. (2019). Phylogenetically, *A. rava* is closely related to two other spinulose *Adustochaete* species from the neotropics, *A. minuta* and *A. nivea* (Figs 2, 13, 14).

#### Adustochaete scalena

Taxon classificationAnimaliaAuricularialesAuriculariaceae

Spirin & Ryvarden sp. nov.

4BA78139-DC63-5873-BCC8-8C1A04604FBF

862468

Fig. 15F

##### Holotype.

Zimbabwe • Mashonaland East: Binga Swamp Forest, dry partly corticated angiosperm branch, 14 Jan 1989, Ryvarden 26006* (O, isotype – H).

##### Etymology.

Scalenus (Lat., adj.) – scalene, unequilateral; in reference to a variable spore shape.

##### Description.

Basidiomata annual, effused, up to 3 cm in the longest dimension, arid, rather soft, 0.1–0.2 mm thick. Hymenial surface smooth or indistinctly tuberculate, grayish-ochraceous to brownish-gray, sometimes irregularly cracking. Margin floccose, nearly white, adnate, up to 0.3 mm wide.

Hyphal structure monomitic, hyphae clamped; subicular hyphae hyaline, thin-walled or with a distinct wall, subparallel or interwoven, anastomosing, 2–5 μm in diam., subhymenial hyphae hyaline, thin-walled, predominantly interwoven, rather loosely arranged, 1.5–4 μm in diam. Crystals abundant in all parts of basidiomata, encrusting hyphae and hymenial cells, small, angular to sand-like. Cystidia (gloeocystidia) rather rare, thin-walled, single, more or less clearly tapering, often somewhat swollen at the base, 43–63 × 7.5–11.5 μm, contents brownish to grayish-brown, CB+. Hyphidia hyaline or brownish to grayish-brown, thin-walled, single or in groups of 2–3, with subfusiform or hyphoid stem, 20–54 × 3.5–9 μm, bearing several richly branched apical outgrowths, 1–2 μm in diam. at the very apex; apical branches encrusted by small, densely arranged crystals. Basidia four-celled, narrowly obconical, sessile, (19–) 23.5–30 (–30.5) × (10.2–) 11.2–14.2 (–14.3) μm (n = 20/1), thin-walled, mostly exposed, occasionally with a reduced stalk-like base, up to 5 × 3 μm, sterigmata up to 45 × 4–5 μm. Basidiospores thin-walled, broadly cylindrical, slightly or moderately curved, or narrowly ellipsoid, (11.1–) 11.2–15.8 (–16.2) × (6.2–) 6.3–9.1 (–9.2) μm (n = 30/1), L = 13.80, W = 7.90, Q = 1.75.

##### Remarks.

*Adustochaete scalena* is described here based on a single collection from Zimbabwe. It differs from *A. fumida*, another smooth *Adustochaete* species from the same area, in having darker basidiomata, larger and more abundant cystidia, as well as longer basidia and wider basidiospores. The basidiospores of *A. scalena* are quite diverse in shape, varying from curved-cylindrical to nearly ellipsoid. Superficially, the basidiomata of *A. scalena* are reminiscent of senescent, withered individuals of *Proterochaete furfurosa*. Their differences are listed under the latter species.

#### Adustochaete tribulata

Taxon classificationAnimaliaAuricularialesAuriculariaceae

Spirin & Ryvarden sp. nov.

DB577AA4-1729-5482-8615-92B3C91BE1DD

862469

Fig. 15G

##### Holotype.

Uganda • Kabarole: Kibale National Park, Makere University Field Station, dead, partly corticated branches, 20 Apr 2002, Ryvarden 44803* (O, isotype – H).

##### Etymology.

Tribulatus (Lat., adj.) – sharpened.

##### Description.

Basidiomata annual, effused, gregarious, up to 2 cm in the longest dimension, arid, rather soft, 0.05–0.1 mm thick. Hymenial surface spinulose, cream-colored to grayish, spines rather regularly distributed, sharp-pointed, 60–200 × 40–50 μm, 8–10 per mm. Margin pruinose, white to cream-colored, adnate, up to 0.5 mm wide.

Hyphal structure monomitic, hyphae clamped; subicular hyphae hyaline, slightly thick-walled, subparallel, tightly agglutinated, not encrusted, 2–4 μm in diam., subhymenial hyphae hyaline, thin- to slightly thick-walled, interwoven, rather loosely arranged, 2–3 μm in diam., occasionally inflated up to 5 μm in diam., hyphae of hymenial spines hyaline, slightly thick-walled, ascending, CB (+), some irregularly inflated, 1.5–3.5 μm in diam. Crystals abundant in all parts of basidiomata, encrusting hyphae and hymenial cells, small, angular or sand-like. Cystidia occasionally present, hyaline or grayish-brownish, thin- to slightly thick-walled, clavate to broadly clavate or somewhat tapering, sometimes sinuous, predominantly embedded, 15–30 × 6–12 μm. Hyphidia abundant, dominating in hymenial layer, hyaline, slightly or distinctly thick-walled, CB (+), originating from subtend subhymenial hyphae, frequently dichotomously branched, 1–1.5 μm in diam. at the very apex, richly encrusted by small, densely arranged crystals. Basidia four-celled, ellipsoid-ovoid to nearly obconical, sessile, (12.5–) 13–17 (–17.5) × (9.3–) 9.7–12.1 (–12.2) μm (n = 23/1), thin-walled or with a distinct wall, embedded or exposed, sterigmata up to 15 × 3–3.5 μm. Basidiospores thin-walled, narrowly cylindrical to cylindrical, clearly curved, (11.4–) 12.1–17.0 (–17.2) × (4.8–) 5.0–5.8 (–6.1) μm (n = 30/1), L = 14.85, W = 5.30, Q = 2.82.

##### Remarks.

*Adustochaete tribulata* is macroscopically similar to two other whitish *Adustochaete* species described above, viz. *A. minuta* and *A. nivea*. It differs from them in having firm, abundant, richly branched and encrusted hyphidia dominating in the hymenial layer. *Adustochaete tribulata* is the single spinulose *Adustochaete* species so far detected in Africa. It is currently known only from the type locality in Uganda.

#### Descidia

Taxon classificationAnimaliaAuricularialesAuriculariaceae

Spirin gen. nov.

721BFD3F-AB4B-5682-905B-34280079853F

862470

##### Etymology.

From ‘descisco’ (Lat., verb) – to secede, to desert.

##### Description.

Basidiomata annual, gelatinous, resupinate, with elevated margin, or adpressed-orbicular, turbinate to nearly cerebriform, hyaline, white or ivory-yellowish to light orange-brown. Abhymenial surface smooth; hymenial surface almost smooth to indistinctly or clearly folded, spine-like projections absent. Crystals accidental. Hyphal structure monomitic; hyphae clamped, as a rule not encrusted. Cystidia absent. Hyphidia present, usually forming a continuous layer. Basidia four-celled, longitudinally septate, ellipsoid-ovoid. Basidiospores hyaline, thin-walled, cylindrical or allantoid, often distinctly curved. On dead wood of angiosperms.

##### Generic type.

*Tremella thuretiana* Lév.

The genus is introduced here to accommodate two *Exidia* s. lato species, *E. repanda* and *E. thuretiana*, which cluster together outside of the main *Exidia* clade (Figs 1, 2). These species differ from almost all genuine *Exidia* species in having a rather loose epihymenial layer which is composed of smooth (i.e. non-encrusted) apical branches of hyphidia. Moreover, *E. repanda* and *E. thuretiana* do not produce spine-like projections on the hymenial surface, which are characteristic of the great majority of *Exidia* s. str. species. See further remarks under *Exidia*. The exidioid representatives of *Ulocolla* (i.e. *Exidia saccharina* and related species) often lack these projections, too. However, the epihymenial layer in these species consists of variably thick-walled and often inflated, pigmented (brownish) hyphidia. Moreover, jelly-like species of *Ulocolla* occur exclusively on coniferous hosts while the two *Descidia* spp. are restricted to angiosperm wood.

#### Descidia repanda

Taxon classificationAnimaliaAuricularialesAuriculariaceae

(Fr.) Spirin comb. nov.

8D87440F-C067-5EB4-943F-E8015ABD5954

862471

Figs 15H, 16B

≡ Exidia repanda Fr., Systema Mycologicum 2: 225, 1822. Neotype (selected by Aoki and Tubaki 1986: 881). Sweden. Uppland: Lena, Ängeby, Rönningen, Betula sp., 11 Nov 1935, Bruun & Lundell (Lundell & Nannfeldt, Fungi Exsiccati Suecici #266) (UPS F-010989, studied).
= Exidia qinghaiensis S.R. Wang & Thorn, Mycoscience 62: 213, 2021. Holotype. China. Qinghai: Menyuan, Xianmi, Betula sp., 19 Oct 2004, Xiaoqing (HMAS 156328*).

##### Description.

Basidiomata annual, first cushion-shaped, adpressed-orbicular or turbinate, up to 3 cm in diam., narrowly or broadly attached, later fusing together and forming compound cerebriform basidiomata, up to 20 cm in the longest dimension and 2 cm thick, gelatinous. Hymenial surface smooth to indistinctly or clearly folded, amber-yellow to reddish or reddish-brown, in dry condition normally darkening to dirty-brown, sometimes with a lilac hue. Abhymenial surface smooth, concolourous with hymenial surface. Margin free, concolourous with hymenial surface.

Hyphal structure monomitic, hyphae clamped; context hyphae hyaline, thin- to slightly thick-walled, interwoven or subparallel, spaced, anastomosing, 0.5–6.5 μm in diam., subhymenial hyphae hyaline or yellowish, thin- to slightly thick-walled, predominantly interwoven, 1–4 μm in diam., basidia-bearing hyphae 2–4 μm in diam. Hyphidia with a hyaline or yellowish, often slightly thick-walled, hyphoid stem, 2–5 μm in diam., and hyaline apical branches, 0.5–1.2 μm in diam., in mature basidiomata cemented by yellowish or reddish amorphous substance and forming a continuous layer up to 35 μm thick. Probasidia predominantly tapering, 9–20 × 4–6 μm. Basidia four-celled, ellipsoid-ovoid, (9–) 9.5–16 (–16.5) × (7.6–) 7.8–9.7 (–10.3) μm (n = 40/4), sterigmata up to 15 × 1.5–2 μm. Basidiospores narrowly cylindrical to allantoid, usually distinctly curved, (9.2–) 10.1–15.1 (–15.4) × (2.7–) 2.8–4.1 (–4.2) μm (n = 150/5), L = 11.87–13.11, W = 3.15–3.75, Q = 3.19–4.19.

##### Remarks.

*Descidia repanda* is widely distributed in the temperate and boreal areas of Eurasia. It occurs exclusively on dead, hanging or just fallen, corticated branches of *Betula* spp. No verified records of *D. repanda* from North America are known to us. Macroscopically, *D. repanda* is most similar to *Exidia candida*. Their differences are discussed under the latter species. This species was first introduced by Fries (1822) as *Exidia repanda*. No original specimens of *E. repanda* exist and the protologue implies that Fries’ species was originally a mixture of *E. repanda* in the present sense and *E. candida*. In particular, *Alnus* as a host tree and minute, papillose projections on the hymenial surface point to the latter species. Nevertheless, *E. repanda* was neotypified with a specimen growing on *Betula* (Aoki and Tubaki 1986). We studied the neotype and concluded that it is conspecific with *E. repanda* as it was interpreted in North Europe by Neuhoff (1935), Raitviir (1970), Torkelsen (1972), and Hansen and Knudsen (1997). Specimens and sequences of *E. repanda* by Wu et al. (2020) belong to *Exidia straminea* instead (see below).

#### Descidia thuretiana

Taxon classificationAnimaliaAuricularialesAuriculariaceae

(Lév.) Spirin comb. nov.

47ECF90A-050B-535F-B804-9428734DAA86

862472

Figs 15I, 16C, 18

≡ Tremella thuretiana Lév., Annals des Sciences Naturelles, Botanique 3 (9): 127, 1848. Lectotype (selected here, MBT 10031428). France. Seine-et-Marne: Bussy-Saint-Martin, Rentilly, Carpinus betulus, [no collecting date], Léveillé (PC, studied).
= Exidia thuretiana (Lév.) Fr., Hymenomycetes Europaei: 694, 1874.
= Heterochaete europaea Höhn., Annales Mycologici 1: 393, 1903. Holotype. Bosnia and Herzegovina. Central Bosnia: Jajce, Fagus sylvatica, 14 Apr 1901, Höhnel (FH00304798, studied).
= *Sebacina plumbea* Bres. & Torrend, Brotéria, sér. Bot. 11: 87, 1913. Holotype. Portugal. [no locality and collecting date indicated], Torrend (S F20661, studied).

##### Description.

Basidiomata annual, at first cushion-shaped or adpressed-orbicular, up to 2 cm in diam., broadly attached, later partly or completely fusing together and forming compound effused basidiomata, up to 30 cm in the longest dimension and 1.5 cm thick, gelatinous. Hymenial surface smooth or covered by shallow folds, pure white to cream-coloured, sometimes with a bluish hue, in older basidiomata with brownish tints, especially at the middle, in dry condition darkening to grayish- or reddish-brown. Abhymenial surface smooth, concolorous with hymenial surface. Margin sharply delimited, often somewhat elevated and partly detaching, occasionally radially furrowed or indistinctly lobate.

**Figure 18.**
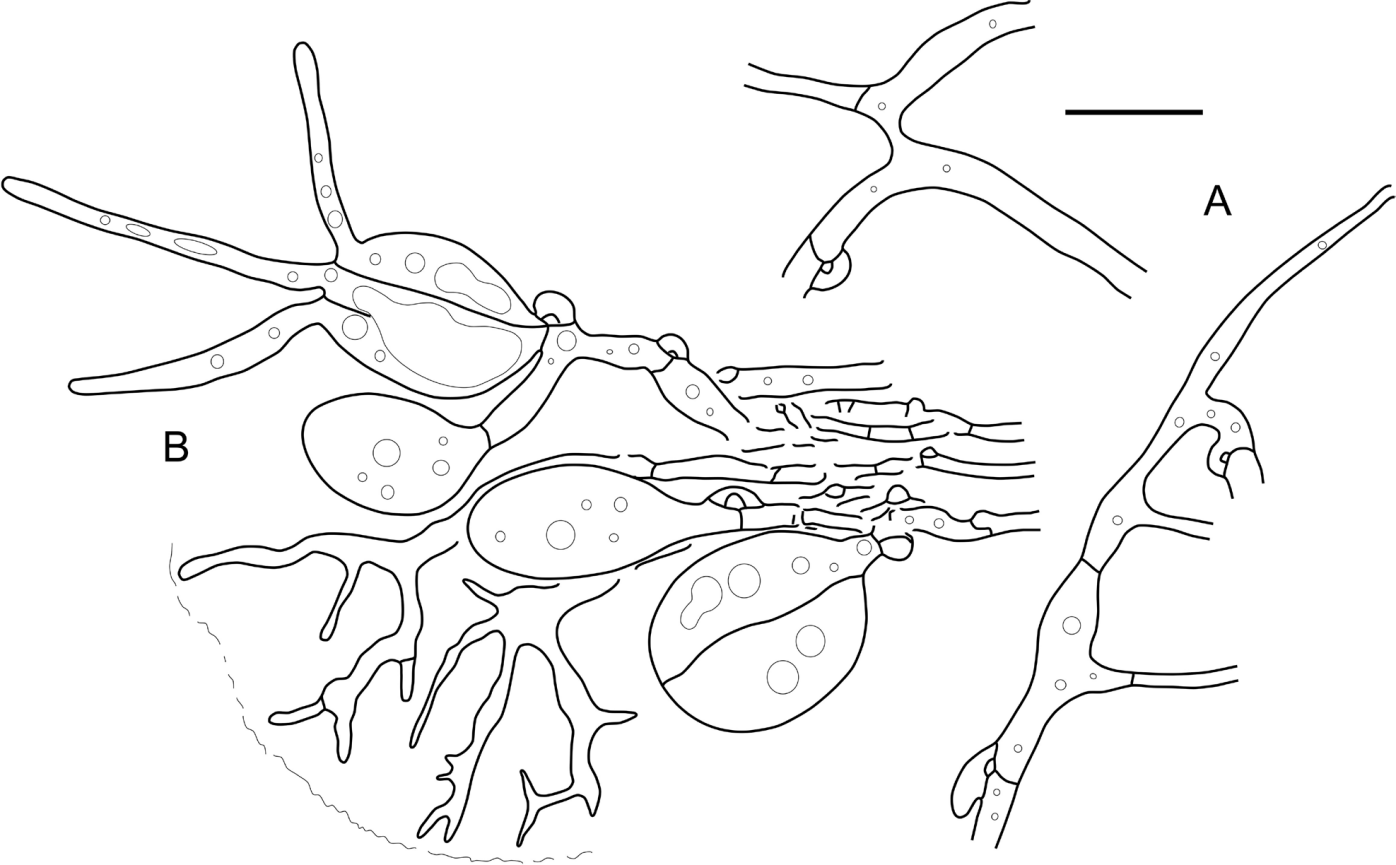
Microstructures of *Descidia thuretiana* (Spirin 17055): **A** context hyphae; **B** subhymenial hyphae and hymenial cells. Scale bar: 10 μm.

Hyphal structure monomitic, hyphae clamped; context hyphae hyaline, thin-walled or with a distinct wall, interwoven, anastomosing, 0.5–5 μm in diam., subhymenial hyphae hyaline, thin-walled or with a distinct wall, ascending or interwoven, 0.5–3 μm in diam., basidia-bearing hyphae 2–6 μm in diam. Hyphidia with a hyaline, hyphoid or subfusiform stem, 1.5–9 μm in diam., and hyaline apical branches, 0.5–2 μm in diam., forming rather loose continuous layer up to 30 μm thick. Probasidia clavate or tapering, 15–30 × 5–9.5 μm. Basidia four-celled, embedded or exposed, ellipsoid-ovoid to almost obconical, more rarely subglobose, (16–) 17–28 (–29) × (9.9–) 10.6–19.1 (–19.2) μm (n = 60/6), occasionally with a distinct stalk-like base, up to 7 × 3.5 μm, sterigmata up to 70 × 2.5–3 μm. Basidiospores cylindrical to broadly cylindrical, distinctly curved, (11.8–) 12.1–20.2 (–20.9) × (5.1–) 5.2–8.1 (–8.2) μm (n = 264/9), L = 14.87–18.43, W = 5.79–7.03, Q = 2.26–2.88.

##### Remarks.

Pale basidiomata, often showing a strong tendency to effused growth, as well as large basidiospores make *Descidia thuretiana* easily recognizable. Microscopically, it is similar to two effused *Exidia* species, *E. delita* and *E. plumbescens*, described below. From them, *D. thuretiana* can be differentiated by its larger basidia and basidiospores. Moreover, the basidiome margin of *D. thuretiana* is always elevated and often (at least partly) detached from the substrate. In the two aforementioned *Exidia* spp., the margin is constantly adnate and gradually thinning. *Descidia thuretiana* is widely distributed in nemoral and hemiboreal zones of Europe. It occurs on a wide range of angiosperm trees and shrubs (and rarely also on thick grass stems) in both natural and human-induced habitats. Another member of *Descidia*, *D. repanda*, differs from *D. thuretiana* in having smaller basidia, as well as shorter and distinctly narrower basidiospores. The basidiomata of *D. repanda* are more distinctly colored, and it occurs exclusively on *Betula* branches, mostly in areas with a continental climate.

The species was first described by Léveillé (1848) as *Tremella thuretiana*. According to the protologue, the original collections of *T. thuretiana* were made from branches of *Fagus sylvatica*. We could not trace them in herbarium PC. Therefore, we select the only authentic specimen (with Léveillé’s own handwriting) collected in the type locality as a lectotype of *T. thuretiana*. This specimen is in relatively good condition, fertile, and is undoubtedly conspecific with other *D. thuretiana* collections studied by us. Due to its macroscopic variability, *D. thuretiana* was subsequently described at least twice, as *Heterochaete europaea* and *Sebacina plumbea* (see synonymy above). Both taxa refer to *D. thuretiana* in its effused condition.

Some authors have argued that *Tremella albida* is, in fact, the oldest available name for *D. thuretiana* (Brefeld 1888; Neuhoff 1936a; Donk 1966). After studying the protologue of *T. albida* (Hudson 1778), as well as the species’ reintroduction in the sanctioning work (Fries 1822), we find it hard to believe that *T. albida* is conspecific with *D. thuretiana*. Hudson’s description is difficult to interpret unambiguously, and his reference to ‘*Echinus gelatinosus crystallinus*’ by Haller (1768) (currently *Pseudohydnum gelatinosum*) makes it even more difficult to understand. As for Fries’ redescription of *T. albida*, it is almost equally ambiguous, although some elements (undulate basidiomata, yellowish colouration) suggest that Fries most likely dealt with small, discoloured forms of *Tremella mesenterica* Retz. No authentic specimens of *T. albida* are extant, however, and our suggestion is merely hypothetical.

#### Eichleriella

Taxon classificationAnimaliaAuricularialesAuriculariaceae

Bres., Annales Mycologici 1: 115, 1903

2AE58E1C-CB55-53E3-8DB1-E52D86582621

= Amphistereum Spirin & Malysheva, Fungal Biology 121: 696, 2017. Generic type. Eichleriella schrenkii Burt.

##### Note.

For a description, see Malysheva and Spirin (2017).

##### Generic type

**(*lectotype*)**. *Eichleriella incarnata* Bres. (selected by Burt 1915: 743).

In our earlier treatment of *Eichleriella* and related genera (Malysheva and Spirin 2017), the genus *Amphistereum* was introduced to encompass two stereoid, dimitic species, *A. leveilleanum* and *A. schrenkii*. In the ITS – LSU-based phylogeny, they clustered with *Eichleriella* s. str., although without statistical support, and so were placed in a separate genus. Our current data (Figs 1, 2) show that *A. leveilleanum* is part of a strongly supported *Eichleriella* clade. *Amphistereum* is therefore considered a later synonym of *Eichleriella*.

#### Eichleriella macrochaeta

Taxon classificationAnimaliaAuricularialesAuriculariaceae

(Bres. & Torrend) Spirin & Malysheva comb. nov.

79A72E15-8B5F-5D67-B382-25279669DD77

862473

≡ Heterochaete macrochaeta Bres. & Torrend, Brotéria, sér. Bot. 11: 86, 1913. Holotype. Portugal. Lisbon: Sintra, Quercus suber, [no collection date indicated], Torrend 236 (S F21050, studied).
= Sebacina shearii Burt, Annals of the Missouri Botanical Garden 2: 758, 1915. Holotype. USA. District Columbia: Washington, Dept. of Agriculture Grounds, Berberis vulgaris, 25 Oct 1902, Shear 1238 (BPI 719719 – isotype, studied).
= Eichleriella shearii (Burt) Spirin & Malysheva, Fungal Biology 121: 708, 2017.

##### Note.

Bresadola and Torrend (Torrend 1913) introduced *Heterochaete macrochaeta* based on a single collection from Portugal. Wells (1961) suggested that *H. macrochaeta* could be conspecific with *Sebacina strigosa* Bourdot & Galzin described from France (Bourdot and Galzin 1909). After studying type specimens of both species, we concluded that they belong to two different species. *Heterochaete macrochaeta* is definitely the same species as *Eichleriella shearii* (Burt) Spirin & Malysheva which is widely distributed in America and also occurring in Europe (see Malysheva and Spirin 2017). Since *H. macrochaeta* predates *H. shearii*, a new combination in *Eichleriella* is proposed here. Our data confirm the presence of *E. macrochaeta* in the southern part of Africa. As for *S. strigosa*, it was made the generic type of *Fibulosebacea* by Wells and Raitviir (1987). Despite its high morphological similarity to some *Exidiopsis* s. lato species, our data suggest that *S. strigosa* does not belong to *Auriculariaceae* or even *Auriculariales*. Its proper taxonomic placement will be clarified on another occasion.

#### Elmericium

Taxon classificationAnimaliaAuricularialesAuriculariaceae

Spirin & Malysheva, MycoKeys 120: 348, 2025

11D617F4-7091-5C85-885B-D5BA3E19BE23

##### Note.

For a description, see Spirin et al. (2025).

##### Generic type.

*Elmericium alabastrinum* Spirin & Malysheva.

This genus was recently introduced as monotypic, with the East Asian *E. alabastrinum* as the sole species (Spirin et al. 2025). Here we expand it with the newly described *E. spinosum* from Africa (see Suppl. material 5).

#### Elmericium spinosum

Taxon classificationAnimaliaAuricularialesAuriculariaceae

Spirin & Viner sp. nov.

B7EA6988-0750-5530-958D-8C6DAD3583DC

862474

Fig. 15J

##### Holotype.

Cameroon • Campo Prov.: Akok Lowland Rainforest Reserve, corticated angiosperm branch on the ground, 2–15 Dec 1991, Ryvarden 30580* (O, isotype – H).

##### Etymology.

Spinosus (Lat., adj.) – spinose, bearing spines.

##### Description.

Basidiomata annual, effused, up to 10 cm in the longest dimension, opaque, gelatinous, 0.1–0.2 mm thick. Hymenial surface smooth or spinulose, gray to brownish-gray, spines irregularly distributed, sharp or blunt, up to 100 × 100 μm, 6–8 per mm. Margin gradually thinning, concolourous with hymenial surface, adnate, up to 1 mm wide.

Hyphal structure dimitic (skeletals present in subiculum only), hyphae clamped; subicular generative hyphae hyaline, slightly to distinctly thick-walled, CB (+) to CB+, subparallel, tightly agglutinated, 1.5–3.5 μm in diam., skeletal hyphae rather rare, hyaline, distinctly thick-walled, CB (+), 1–2 μm in diam., subhymenial hyphae hyaline, thin-walled or with a distinct wall, interwoven, rather densely arranged and partly agglutinated, 1–3 μm in diam., hyphae of hymenial spines hyaline, slightly or distinctly thick-walled, CB (+) to CB+, ascending or interwoven, 2–4.5 μm in diam. Crystals abundant in all parts of basidiomata, angular, often in large groups (up to 25 μm in widest dimension). Cystidia absent. Hyphidia hyaline, thin-walled, richly branched, 1.5–2 μm in diam. (apical branches), partly covering basidial cells. Basidia four-celled, ellipsoid-ovoid to broadly ellipsoid, sessile, (12–) 13–15.5 (–16) × (8.1–) 8.2–11.2 (–11.8) μm (n = 20/1), with a distinct wall or slightly thick-walled, exposed, often glued in groups of up to four, occasionally with a reduced stalk-like base, up to 5 × 5 μm, sterigmata up to 11 × 3–3.5 μm. Basidiospores thin-walled, narrowly cylindrical to cylindrical, clearly curved, (8.6–) 9.1–11.3 (–12.2) × (3.7–) 3.9–4.7 (–4.8) μm (n = 30/1), L = 10.32, W = 4.19, Q = 2.47.

##### Remarks.

Despite their phylogenetic affinity, *E. spinosum* and *E. alabastrinum* (the generic type of *Elmericium*) show quite significant morphological differences. Macroscopically, *E. spinosum* can be differentiated from *E. alabastrinum* by its spinulose hymenophore (vs. completely smooth in the latter species). The most striking anatomical feature of *E. spinosum* is the presence of subicular skeletal hyphae strongly reminiscent of narrow, occasionally branched, solid skeletals of the poroid *Aporpium hexagonoides* A. David & Jaq. from the *Elmerina* clade. In turn, basidiomata of *E. alabastrinum* are completely monomitic. Moreover, *E. alabastrinum* possesses cystidia, and its basidia and basidiospores are clearly larger than those of *E. spinosum*. Basidia of *E. alabastrinum* have a long and thick basal stalk. This basal basidial segment, if present, is strongly reduced in *E. spinosum*.

#### Exidia

Taxon classificationAnimaliaAuricularialesAuriculariaceae

Fr., Systema Mycologicum 2: 220, 1822

CEF68008-F562-5253-91D8-6AF5525DF2B4

= Tremellochaete Raitv., Eesti NSV Teaduste Akadeemia Toimetised 13: 29, 1964. Generic type. Exidia japonica Lloyd.

##### Description.

Basidiomata annual, gelatinous, adpressed-orbicular, turbinate or cerebriform, more rarely resupinate and then with adnate, gradually thinning margin, reddish-brown to almost black, more rarely lighter-coloured. Abhymenial surface smooth, dotted, papillose or hirsute; hymenial surface smooth to indistinctly or clearly folded, spine-like projections present in most species. Crystals usually abundant; dark-colored granular incrustation as a rule present on hyphidia and hyphae (apparently lacking in five pale-coloured species). Hyphal structure monomitic; hyphae clamped, in many species encrusted. Cystidia present only in two resupinate species. Hyphidia present, often forming a continuous layer (epihymenial membrane). Basidia four-celled, longitudinally septate, ellipsoid-ovoid or broadly clavate. Basidiospores hyaline, thin-walled, broadly cylindrical to allantoid, usually distinctly curved. On dead wood of angiosperms, more rarely on conifers.

##### Generic type.

*Tremella glandulosa* Bull.

Here we reintroduce *Exidia* for most species previously ascribed to this genus, at least those distributed in Europe. Two other genera encompassing *Exidia*-like fungi are *Descidia* and *Ulocolla*. From them, *Exidia* s. str. can be distinguished due to two quite distinctive features. First, the majority of *Exidia* spp. treated below possess spine-like or (in some subtropical / tropical species formerly treated under *Tremellochaete*) wart-like projections on the hymenial surface. However, these seem to be constantly lacking in a few species of *Exidia* s. str. Some individuals of the normally papillose species can be devoid of them, too. Additionally, *Ulocolla saccharina* may sometimes develop clearly visible and rather regularly arranged hymenial projections. In these cases, the genuine *Exidia* species can be separated from the other exidioid taxa by the permanent presence of the characteristic granular encrustations on hyphidia and, in many cases, also on subhymenial and context hyphae (Figs 21, 22). These discrete deposits are as a rule coloured reddish, brownish or nearly black, and their presence causes the overall basidiome colouration. The incrustation of this type is clearly different from the amorphous yellowish or brownish substance cementing an epihymenial layer in mature basidiomata of *Descidia repanda* and the exidioid *Ulocolla* spp. The only two exceptions known to us are *Exidia candida* and *E. semiorbis*. In these species, no signs of granular encrustation on hyphidia or hyphae have been detected. Moreover, the epihymenial membrane in *E. candida* var. *candida* (= *E. villosa*) is very loose and it is eventually absent in *E. semiorbis*. These deviating features make it difficult to differentiate these two species from similar *Descidia* spp. However, most specimens of *E. candida* and all known specimens of *E. semiorbis* bear spine-like projections on their hymenial surface. The basidiospores of *E. candida* are cylindrical to broadly cylindrical and shorter than in *Descidia* spp. These two traits help in differentiating *E. candida* from the superficially similar *D. repanda* and *D. thuretiana*. The basidiospores of *E. semiorbis* are very similar to those of *D. repanda*. However, mature basidiomata of *E. semiorbis* are orbicular-cupulate and have a warted abhymenial surface, while they are turbinate or pulvinate and smooth on the outside in *D. repanda*.

The inclusion of four completely resupinate, smooth species in *Exidia*, i.e. *Sebacina plumbescens* (= *Exidiopsis succinea*) and the newly described *Exidia delita*, *E. repleta*, and *E. saturata*, poses another problem – namely, it obfuscates the limits of *Exidia* vs. the redefined *Exidiopsis* (*E. effusa* and *E. perflua*), *Proterochaete* (*P. abstrusa*), *Tegmenticium* (*T. peritrichum*) and *Ulocolla* (*U. grisea*). The four effused *Exidia* species can be differentiated from the representatives of the aforementioned genera by their thicker, clearly gelatinous basidiomata and, as a rule, larger basidia. In *E. delita* and *E. plumbescens*, the basidiomata usually produce numerous embedded mineral grains easily detectable under magnification. These inclusions are very rare or completely lacking in the similar-looking species of *Exidiopsis*, *Proterochaete*, *Tegmenticium*, or *Ulocolla*. In turn, *E. repleta* and *E. saturata* can be differentiated from the representatives of the four mentioned genera in having thicker epihymenial layer and distinctly inflated hyphae.

The identity of the generic type, *E. glandulosa*, is still a subject of ongoing controversy. We address this issue in remarks to *E. glandulosa*, *E. pithya* and *E. truncata*. In total, 26 *Exidia* species are described or commented on below and several insufficiently known taxa belonging to the redefined *Exidia* are addressed in the corresponding section of the paper.

#### Exidia candida

Taxon classificationAnimaliaAuricularialesAuriculariaceae

Lloyd, Mycological Writings 5: 620, 1916

5B6B20FB-3DF1-59CE-98D3-6951030F03D2

Fig. 16D, E

= Exidia villosa Neuhoff, Pilze Mitteleuropas 2: 22, 1935. Neotype (designated by Spirin et al. 2018: 5). Norway. Oslo: Bygdøy, Dronningberget, Tilia cordata, 3 May 2016, Spirin 10012* (O, studied).
= Exidia cartilaginea S. Lundell & Neuhoff, Pilze Mitteleuropas 2: 19, 1935. Lectotype (selected by Spirin et al. 2018: 7). Sweden. Uppland: Bondkyrka, Vårdsätra, Betula sp., 18 Oct 1932, Lundell (Fungi Exsiccati Suecici #263) (UPS F-010986, studied).

##### Lectotype

**(selected by Spirin et al. 2018: 5)**. USA • Washington: Clallam Co., Sequim, [no collecting date], Grant (BPI 701884, studied) (Fig. 16D, E).

##### Description.

Basidiomata annual, at first cushion-shaped or adpressed-orbicular, up to 5 cm in diam., broadly attached, later partly fusing together and forming compound cerebriform basidiomata, up to 20 cm in the longest dimension and 2 cm thick, gelatinous. Hymenial surface uneven or folded, occasionally bearing regularly arranged spine-like projections up to 0.3 mm long, white to cream-coloured or pale ochraceous-brownish, sometimes darker, reddish-brown, in dry condition darkening to brown or brownish-black. Abhymenial surface smooth, concolourous with hymenial surface. Margin sharply delimited, elevated, concolourous with hymenial surface, or adnate, fimbriate, whitish and gradually thinning.

Hyphal structure monomitic, hyphae clamped; context hyphae hyaline, thin-walled or with a distinct wall, interwoven, anastomosing, 0.5–5 μm in diam., subhymenial hyphae hyaline or yellowish, thin-walled or with a distinct wall, predominantly interwoven, 1.5–4 μm in diam., basidia-bearing hyphae 2–4 μm in diam., often showing a zigzag-like branching pattern. Hyphidia with a hyaline or yellowish, hyphoid or narrowly clavate stem, 2.5–6 μm in diam., and hyaline or yellowish apical branches, 1–2 μm in diam., forming a continuous layer up to 40 μm thick. Probasidia clavate or tapering, 10–22 × 4–7 μm. Basidia four-celled, predominantly embedded, ellipsoid-ovoid, occasionally distinctly tapering to the base, (12–) 12.5–18 (–21) × (7.8–) 7.9–11.3 (–11.4) μm (n = 30/3), sometimes obliquely septate, sterigmata up to 30 × 2–2.5 μm. Basidiospores narrowly to broadly cylindrical, distinctly curved, (8.7–) 8.9–15.2 (–15.6) × (3.1–) 3.2–5.2 (–5.3) μm (n = 240/8), L = 10.23–12.40, W = 3.97–4.55, Q = 2.35–3.12.

##### Remarks.

*Exidia candida* was described from the US North-West (Lloyd 1916) and recently reinstated by Spirin et al. (2018). Here we redescribe it based on a larger set of specimens from the northern hemisphere. The species is macroscopically very variable. Its darker-colored forms with a well-developed epihymenial membrane were assigned to var. cartilaginea (Spirin et al. 2018). This variety occurs mainly in the northern areas of Eurasia. The southern, “typical” variety (= *E. villosa*) is lighter-coloured, softer and it often (but not always!) bears sharp outgrowths on the hymenial surface. This feature, as well as its shorter and wider basidiospores help to separate *E. candida* from the superficially similar *D. repanda*. Phylogenetically, *E. candida* is more closely related to the effused *Exidia* spp. (*E. delita*, *E. japonica*, *E. plumbescens*) than to other members of the genus with reflexed basidiomata (Figs 1, 2).

#### Exidia ciliata

Taxon classificationAnimaliaAuricularialesAuriculariaceae

(Möller) Spirin comb. nov.

11FF59A7-D2D3-54C8-882A-0EE06A2AB55A

862475

≡ Exidiopsis ciliata Möller, Botanische Mittheilungen aus den Tropen 8: 91, 1895. Lectotype (selected by Alvarenga et al. 2019: 449). Brazil, Santa Catarina, Blumenau, on bark, 1891, Möller 10 (91) (HBG).
= Tremellochaete ciliata (Möller) Spirin & Alvarenga, Botany 97: 449, 2019.
For a description and further synonymy, see Alvarenga et al. (2019).

#### Exidia crenata

Taxon classificationAnimaliaAuricularialesAuriculariaceae

(Schwein.) Fr., Systema Mycologicum 2: 226, 1822

18E0648F-CE6D-5AE7-B64A-C7FC9855AAAD

Fig. 19A

≡ Tremella crenata Schwein., Schriften der Naturforschenden Gesellschaft zu Leipzig 1: 115, 1822. Holotype. USA. North Carolina, [no collecting date indicated], Schweinitz 1141 (PH).
= Tremella corrugata Schwein., Transactions of the American Mycological Society 4: 185, 1832. Holotype. USA. Pennsylvania: Bethlehem, [no collecting date indicated], Schweinitz 1113 (PH00077946, studied).

##### Description.

Basidiomata annual, cupulate-orbicular, up to 2 cm in diam., narrowly attached, later partly fusing together but remaining separable, up to 0.5 cm thick, gelatinous. Hymenial surface uneven or folded, rarely bearing scattered granular projections up to 0.1 mm long, at first ochraceous- to reddish-brown, later dark brown, in dry condition darkening to nearly black. Abhymenial surface rough, covered by tightly arranged dots, reddish-brown or dark brown. Margin free, concolourous with hymenial surface, first entire, even or indistinctly lobate, in older basidiomata usually distinctly incised (crenate).

**Figure 19.**
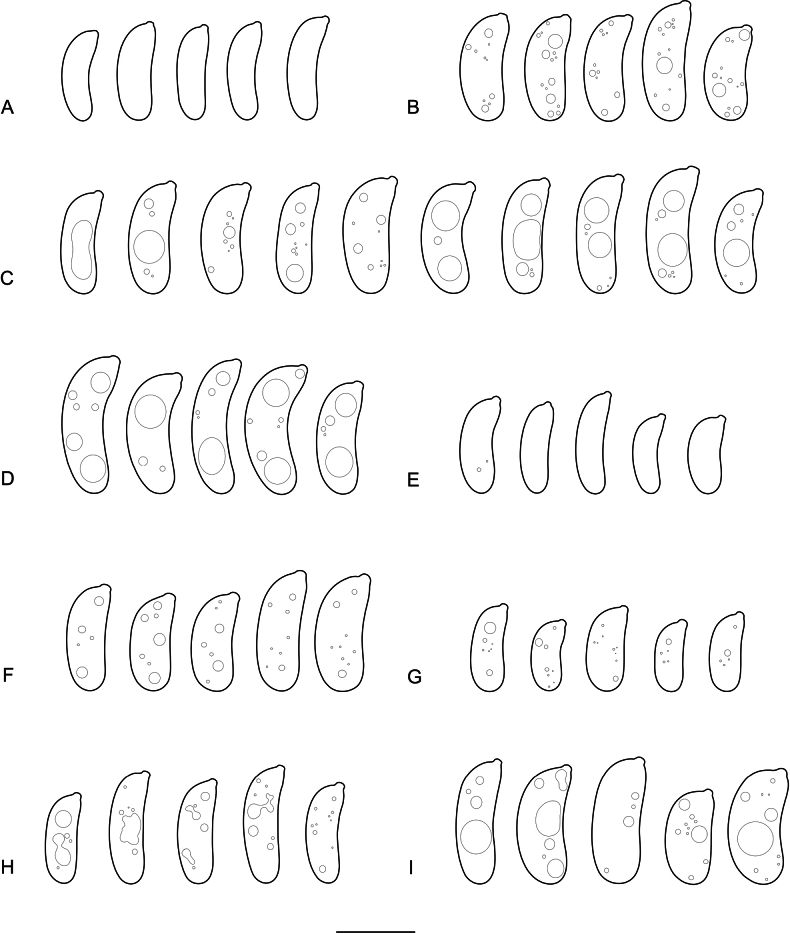
Basidiospores: **A***Exidia crenata* (holotype of *Tremella corrugata*); **B***E. delita* (holotype); **C***E. friesiana* (five left – Spirin 7660, five right – Spirin 18006); **D***E. glandulosa* (Spirin 18030); **E***E. impressa* (neotype); **F***E. inornata* (isotype); **G***E. pithya* (neotype); **H***E. pithya* (J. Olsson 19.12.2024); **I***E. plumbescens* (three left – Spirin 5836, two right – T. Helo 20210767). Scale bar: 10 μm.

Hyphal structure monomitic, hyphae clamped; context hyphae hyaline or yellowish, occasionally encrusted, thin- to slightly thick-walled, interwoven, anastomosing, 1–5 μm in diam., subhymenial hyphae hyaline or yellowish, occasionally encrusted, thin- to slightly thick-walled, predominantly ascending, 1.5–4 μm in diam., basidia-bearing hyphae with a distinct wall, 2–3 μm in diam. Hyphidia with a hyaline or yellowish-brownish, often encrusted, hyphoid or more rarely subfusiform stem, 2–12 μm in diam., and hyaline or yellowish apical branches, 0.8–2 μm in diam., covered by yellowish-brownish gelatinous matrix and forming a continuous layer up to 25 μm thick. Probasidia distinctly tapering or more rarely clavate, 11–15 × 5–7 μm. Basidia four-celled, ellipsoid-ovoid, (10–) 10.5–15 (–15.5) × (7.2–) 7.3–10.6 (–11.4) μm (n = 30/2), sterigmata up to 20 × 1.5–2 μm. Basidiospores cylindrical to allantoid, slightly or distinctly curved, (9.3–) 10.2–14.2 (–14.3) × (3.1–) 3.2–4.6 (–4.8) μm (n = 90/3), L = 11.30–11.73, W = 3.71–3.90, Q = 2.92–3.20.

##### Remarks.

This species was first described as *Tremella crenata* (Schweinitz 1822) and the same year moved to *Exidia* (Fries 1822). Coker (1920) studied the type material of both *T. crenata* and *T. corrugata* and placed them as synonyms of the European *E. recisa* (as *E. gelatinosa*). Since then, *E. crenata* was almost totally forgotten. Recently, Wu et al. (2020) reinstated *E. crenata* as a species on its own and provided a color photo, as well as a brief comparison of it with the newly described *E. yadongensis* from East Asia. We affirm their observations. *Exidia crenata* is phylogenetically closely related to *E. yadongensis*, and they belong to the *E. recisa* complex (Fig. 9). They can hardly be separated from each other under the microscope. Macroscopically, the scalloped (crenate) margin of mature *E. crenata* individuals is quite characteristic; in *E. yadongensis*, the margin remains more or less entire. *Exidia crenata* occurs exclusively in the eastern part of North America where it inhabits dead branches of *Quercus* spp. In turn, *E. yadongensis* is distributed in East Asia on various deciduous hosts.

#### Exidia delita

Taxon classificationAnimaliaAuricularialesAuriculariaceae

Spirin & Schoutteten sp. nov.

9F38A79D-E575-5655-ACED-4153828124C7

862476

Figs 16F, 19B

##### Holotype.

France • Aveyron: Combret, Bétirac, 43.86348; 2.71597, *Populus nigra* (fallen decorticated log), 6 Nov 2024, Spirin 17810* (H, isotype – PC).

##### Etymology.

Delitus (Lat., part. pf.) – plastered, spread.

##### Description.

Basidiomata annual, effused, up to 7 cm in the widest dimension, gelatinous, opalescent, up to 0.3 mm thick. Hymenial surface indistinctly tuberculate and whitish in fresh condition, smooth and dirty-grayish after drying; mineral inclusions occasionally present, white, mostly small and visible only under lens. Margin rather clearly delimited, adnate, gradually thinning, concolourous with hymenial surface.

Hyphal structure monomitic, hyphae clamped; subicular hyphae hyaline, with variably thickened, gelatinized walls, mostly subparallel, hardly distinguishable, 2–3.5 μm in diam., subhymenial hyphae hyaline or yellowish, thin-walled, easily collapsing, predominantly interwoven, 1.5–3 μm in diam., occasionally covered by small, densely arranged, acicular crystals, basidia-bearing hyphae thin-walled, 2.5–6 μm in diam., occasionally showing a zigzag-like branching pattern. Crystals single, angular, scattered or forming large agglomerations, up to 20 μm in widest dimension. Hyphidia richly branched, with a poorly differentiated, hyphoid stem, 2–4 μm in diam., and hyaline apical branches, 1–1.5 μm in diam., occasionally forming a continuous, rather loose layer up to 20 μm thick. Probasidia ellipsoid-ovoid to broadly clavate, 12–17 × 6–11 μm. Basidia four-celled, often obliquely septate, ellipsoid-ovoid to almost obconical, sessile, (14–) 14.5–20 (–21) × (9.7–) 10.1–12.4 (–12.9) μm (n = 20/1), thin-walled, exposed or rarely embedded, occasionally with a tapering, stalk-like base, up to 12 × 2–7 μm, sterigmata up to 35 × 2.5–3.5 μm. Basidiospores thin-walled, cylindrical, more rarely broadly cylindrical, often distinctly curved, (11.3–) 11.9–17.2 (–17.5) × (4.5–) 4.7–6.4 (–6.5) μm (n = 62/2), L = 13.44–14.77, W = 5.22–5.87, Q = 2.53–2.58.

##### Remarks.

*Exidia delita* is a look-alike of *E. plumbescens*, reintroduced below. It differs from the latter species due to its thinner basidiomata, smaller basidia and hyphidia with poorly differentiated, slenderer stems. The basidiospores of *E. delita* are on average shorter than in most specimens of *E. plumbescens* studied by us; however, their dimensions overlap. Although these species look very much the same and are congeneric, they are not closely related phylogenetically (Figs 1, 2). *Exidia delita* is so far known only from two localities, in France and the Netherlands.

#### Exidia friesiana

Taxon classificationAnimaliaAuricularialesAuriculariaceae

P. Karst. in von Thümen, Mycotheca Universalis 12: 1111, 1878

AC0ADB04-B0D9-5CE4-9B7E-70203F5BFE79

Figs 19C, 20A

##### Lectotype

**(selected here, MBT 10031432)**. Finland • Etelä-Häme: Hämeenlinna, Tammela, Mustiala, *Picea abies*, March.1878 *Karsten* (H, studied).

##### Description.

Basidiomata annual, at first button-shaped or adpressed-orbicular, up to 5 cm in diam., broadly attached, later fusing together but remaining partly separable and forming compound effused basidiomata, up to 20 cm in the longest dimension, up to 0.5 cm thick, gelatinous. Hymenial surface variably folded, very rarely bearing scattered granular projections up to 0.1 mm long, brownish- to bluish-black, in dry condition completely black, shining. Abhymenial surface smooth, concolourous with hymenial surface. Margin free, concolourous with hymenial surface, entire, even or indistinctly lobate.

**Figure 20.**
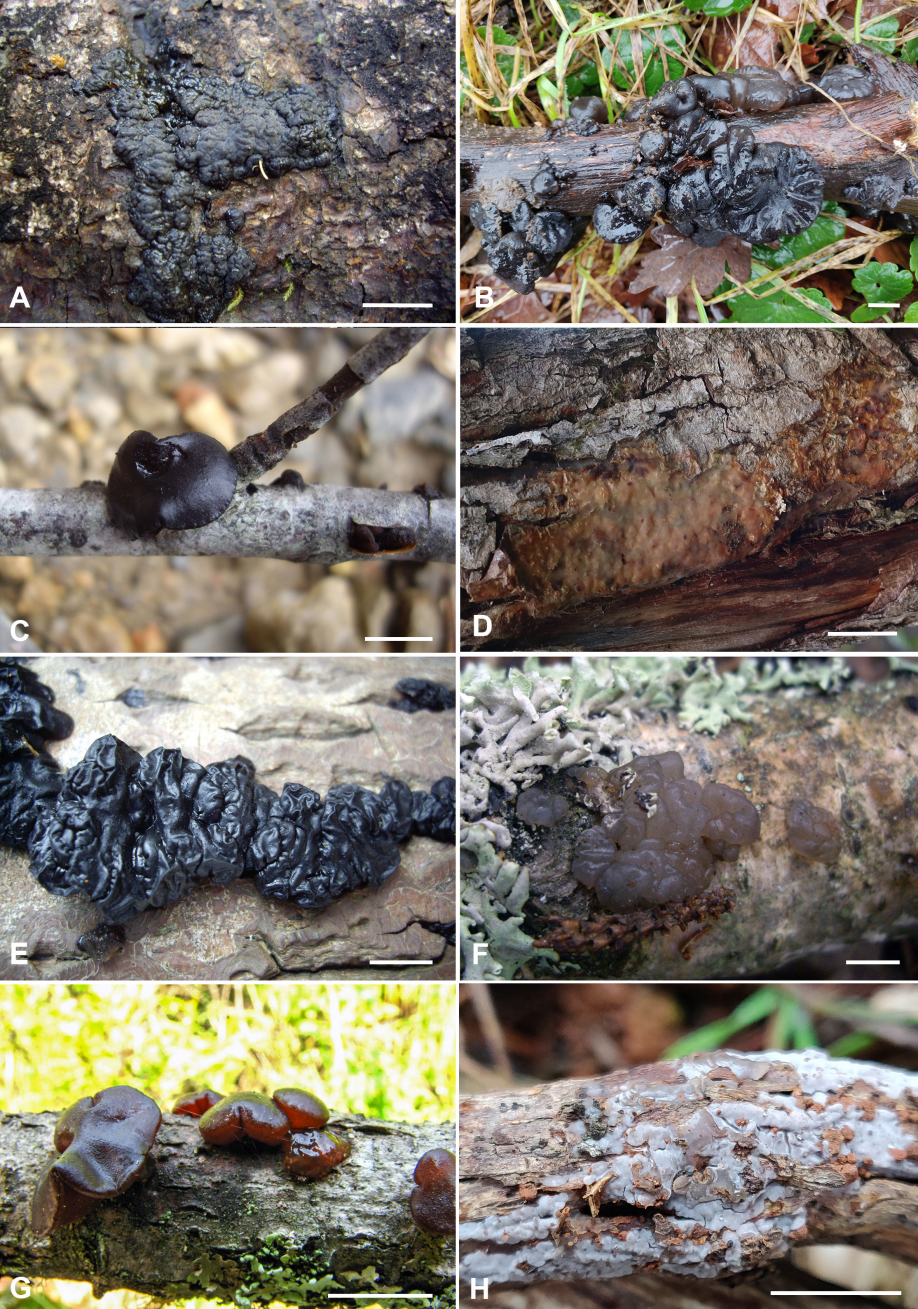
Basidiomata: **A***Exidia friesiana* (Spirin 11067); **B***E. glandulosa* (Spirin 18030); **C***E. impressa* (neotype); **D***Exidia plumbescens* (Kulju 68); **E***Exidia pithya*, dark form (Spirin 17065); **F***Exidia pithya*, pale form (Spirin 11953); **G***E. recisa* (LE 231765); **H***E. saturata* (holotype). Scale bar: 1 cm.

Hyphal structure monomitic, hyphae clamped; context hyphae hyaline to grayish, thin-walled or with a distinct wall, predominantly subparallel, anastomosing, 1–6 μm in diam., subhymenial hyphae hyaline to grayish, thin-walled or with a distinct wall, ascending, 1–4 μm in diam., basidia-bearing hyphae 2–5 μm in diam. Hyphidia with a slightly thick-walled, grayish or brownish, hyphoid or nodulose stem, 2–11 (16) μm in diam., and grayish or brownish apical branches, 1–2 μm in diam., forming rather loose continuous layer up to 60 μm thick. Probasidia clavate or tapering, 12–17 × 6–8 μm. Basidia thin- to slightly thick-walled, four-celled, ellipsoid-ovoid, (12.5–) 13.5–19 (–20) × (9.1–) 10.0–14.4 (–15.2) μm (n = 54/4), sterigmata up to 20 × 2–2.5 μm. Basidiospores narrowly to broadly cylindrical, distinctly curved, (10.2–) 10.7–18.8 (–19.2) × (4.0–) 4.1–6.5 (–6.6) μm (n = 171/6), L = 12.61–15.89, W = 4.39–5.43, Q = 2.41–3.16.

##### Remarks.

*Exidia friesiana* is widely distributed in the northern hemisphere and occurs exclusively on recently fallen, often still corticated wood remnants of *Abies* and *Picea* spp. In the Mediterranean, it was also found on *Pinus halepensis*. Before the present study, *E. friesiana* was treated under the misapplied name *E. pithya* (see remarks on the latter species). Still, the two species are closely related (Figs 9, 10) and morphologically quite similar. Macroscopically, *E. friesiana* can be separated from *E. pithya* by its consistent lack of spike-like hymenial projections, which are typically present in the latter species. Anatomically, *E. friesiana* is easily recognizable even if sterile because of its regularly ascending subhymenial hyphae. In *E. pithya*, subhymenial hyphae are arranged in a much more random manner, predominantly ascending in one part of the basidiome and mostly interwoven in another. It seems that the hyphal arrangement follows the hymenophore configuration, being nearly smooth or faintly radially plicate in *E. friesiana* and strongly and irregularly folded in mature individuals of *E. pithya*. Additionally, the basidiospores of *E. friesiana* are wider and normally longer than in *E. pithya*; however, specimens of both species are often sterile or contain only a few turgid spores. See further notes under *E. pithya*.

#### Exidia glandulosa

Taxon classificationAnimaliaAuricularialesAuriculariaceae

(Bull.) Fr., Systema Mycologicum 2: 224, 1822

80B1606A-17C0-55CD-A5E9-A86E27BFBD62

Figs 19D, 20B, 21

≡ Tremella glandulosa Bull., Herbier de la France 9: pl. 420, fig. 1, 1789. Lectotype (iconotype) (selected here, MBT 10031433). Fig. 1, pl. 420 in Bulliard, Herbier de la France 9, 1789. Epitype (designated here, MBT 10031434). Sweden. Västergötland: Askim, Årekärr, Alnus glutinosa, 15 Mar 1999, E. Larsson 3-99* (GB-0150310, studied).
= Exidia strigosa (P. Karst.) P. Karst., Bidrag till Kännedom av Finlands Natur och Folk 48: 451, 1889. Holotype. Finland. Varsinais-Suomi: Turku, Ruissalo, Corylus avellana, 2 Jun 1869, Karsten 2141 (H6061342, studied).
= Exidia grambergii Neuhoff, Zeitschrift für Pilzkunde 5: 187, 1926.

##### Description.

Basidiomata annual, at first button-shaped or adpressed-orbicular, up to 5 cm in diam., narrowly attached and occasionally with a reduced stalk-like base up to 2 cm long, later turbinate or nearly cupulate, up to 10 cm in diam., sometimes in large groups and partly fusing together, up to 3–4 cm thick, gelatinous. Hymenial surface uneven or variably folded, covered by rather regularly arranged spike-like black projections up to 0.25 mm long, dark gray to brownish-gray, later brownish-black, in dry condition completely black. Abhymenial surface felty or strigose, covered by densely arranged blackish hairs up to 0.2 mm long, grayish-brown to nearly black. Margin free, concolourous with hymenial surface, entire, even or lobate.

Hyphal structure monomitic, hyphae clamped; context hyphae hyaline or grayish, thin- to distinctly thick-walled, predominantly interwoven, anastomosing, 1–7 μm in diam., subhymenial hyphae hyaline to grayish, thin-walled or with a distinct wall, predominantly ascending, 1–3 μm in diam., basidia-bearing hyphae 2–4 μm in diam. Hyphidia with a hyaline or grayish-brownish hyphoid stem, 2–9 μm in diam., and hyaline or grayish-brownish apical branches, 0.5–1 μm in diam., forming a continuous layer up to 100 μm thick. Probasidia clavate to broadly clavate, more rarely tapering, 12–21 × 5–10 μm. Basidia four-celled, ellipsoid-ovoid, thin- or occasionally thick-walled (wall up to 1–1.5 μm thick), (14–) 14.5–24 (–25) × (9.6–) 9.7–13.7 (–13.8) μm (n = 85/8), sterigmata up to 50 × 1.5–2.5 μm. Basidiospores narrowly cylindrical to cylindrical, moderately or strongly curved, (10.3–) 11.0–17.9 (–19.1) × (3.9–) 4.0–6.4 (–6.8) μm (n = 330/10), L = 13.53–15.87, W = 4.56–5.47, Q = 2.48–3.31.

**Figure 21.**
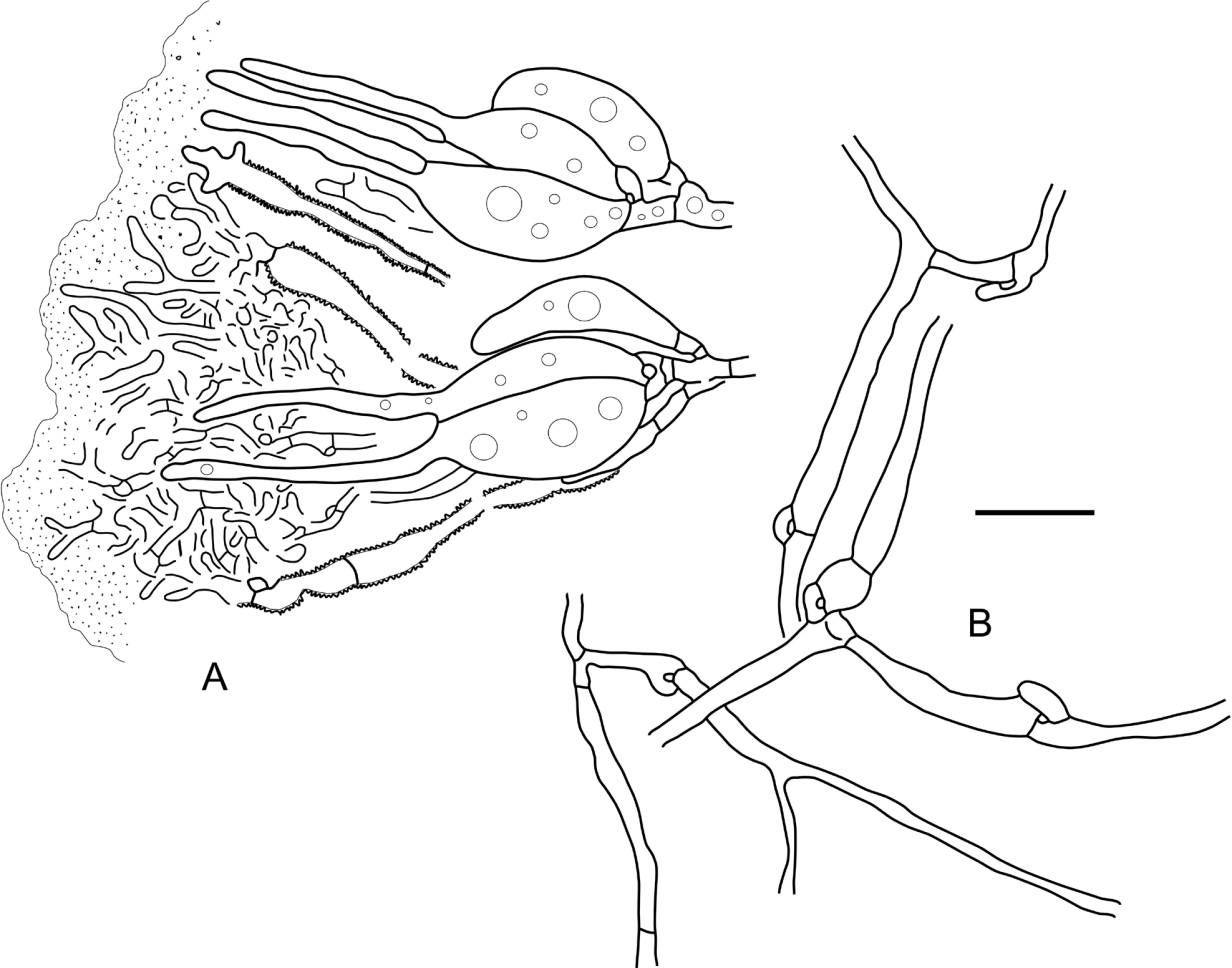
Microstructures of *Exidia glandulosa* (Spirin 18030): **A** subhymenial hyphae and hymenial cells; **B** context hyphae. Scale bar: 10 μm.

##### Remarks.

As redefined here, *E. glandulosa* is morphologically and phylogenetically the closest to *E. truncata*. They both produce adpressed-orbicular or turbinate, nearly black basidiomata with a distinctly spinulose hymenial surface. These species also share similar ecological preferences, being collected and sometimes occurring side by side in the same habitats (nemoral or hemiboreal forests with *Quercus robur*), as a rule on hanging or just fallen branches. *Exidia glandulosa* differs from *E. truncata* in having a hairy abhymenial surface (scabrose or minutely punctate in *E. truncata*) and on average shorter basidiospores.

Up to the present moment, the identity of *E. glandulosa* has remained controversial. Neuhoff (1936b) made the first attempt to sort out names of the dark-colored *Exidia* species in Europe. He coined the name *E. glandulosa* (based on Buillard’s *T. glandulosa*) and applied it to the most common black *Exidia* species with prostrate basidiomata and smooth abhymenial surface. At the same time, he named a similar species with orbicular or turbinate basidiomata and uneven upper surface *E. truncata*. Neuhoff (ibid.) referred to descriptions of *E. glandulosa* and *E. truncata* in Systema Mycologicum (Fries 1822) and stated that they leave no doubt about Fries’ concept of these species. Moreover, he mentioned that the authentic specimens of *E. glandulosa* in Fries’ herbarium in Uppsala corroborate his concept. Neuhoff’s viewpoint on the identity of *E. glandulosa* and *E. truncata* was accepted by most subsequent authors (Pilát 1957; Raitviir 1970; Torkelsen 1972; Wojewoda 1981; Hansen and Knudsen 1997). In recent DNA studies, *E. glandulosa* and *E. truncata* sensu Neuhoff were adopted by Weiß and Oberwinkler (2001), Wu et al. (2020) and Ye et al. (2020).

Donk (1966) proposed an alternative interpretation of the two species. He pointed to the ambiguity of Fries’ descriptions of *E. glandulosa* and stated that some elements of the text in Systema Mycologicum definitely refer to *E. truncata*. Therefore, it is highly likely that Fries himself could not distinguish between the two species. The only certain element in his introduction of *E. glandulosa* (Fries 1822) is a reference to Bulliard’s illustration of *Tremella glandulosa* (Bulliard 1789). It clearly depicts *E. truncata* as interpreted by Neuhoff (1936b), and, in Donk’s opinion, also as understood by Fries himself. As a consequence, Donk (1966) synonymized *E. truncata* with *E. glandulosa* and formally introduced a new name, *E. plana*, for *E. glandulosa* sensu Neuhoff. Roberts (2009) accepted this synonymy but proposed another name, *E. nigricans*, for *E. plana*.

We agree with Donk’s opinion that Fries’ concept of *E. glandulosa* was quite obscure and that the single clear element in his description is the link to Bulliard’s illustration of *T. glandulosa*. We could not trace any specimens in UPS collected or received by Fries prior to the publication of his description of *E. glandulosa* in 1822. Hence, it is uncertain which specimens in Fries’ herbarium in Uppsala Neuhoff referred to. However, there are two specimens with Fries’ handwritings in Lund (herbarium LD) which he studied before publishing Systema Mycologicum. One specimen labelled as *Tremella arborea* Huds. (LD 1473064) is identical with *E. glandulosa* in the sense of Donk and Roberts and the second one (‘*Tremella spiculata* Pers.’, LD 1473124) belongs to *E. nigricans*. Both *T. arborea* and *T. spiculata* were listed by Fries (1822) as synonyms of *E. glandulosa*, and this confirms Donk’s assertion that *E. glandulosa* sensu Fries was a mixture of (at least) two species.

*Exidia glandulosa* has never been typified. The existence of the two authentic specimens by Fries, as well as his reference to Bulliard’s illustration of *T. glandulosa*, confront us with a difficult choice, because either of the specimens or the illustration can be used for typification. One option would be to select the specimen LD 1473124 as a lectotype of *T. glandulosa* and thus support Neuhoff’s view on the identity of this species. This would allow for the retention of *E. glandulosa* as it has been accepted in recent DNA studies. However, this selection would be in the explicit conflict with the original species concept presented by Bulliard (1789), as well as, to some degree, with Fries’ opinion on its identity. From his description and synonymy in Systema, we still can conclude that his *E. glandulosa* mainly referred to a species with a strigose abhymenial surface. We therefore support Donk’s conclusions on the identity of *E. glandulosa* but exclude *E. truncata* from its synonyms. The latter species is redescribed and discussed below. We designate Bulliard’s original illustration of *T. glandulosa* as its lectotype and supplement it with an epitype collected on alder (the only host tree mentioned by Bulliard in the protologue of *T. glandulosa*).

No type material is extant for *E. grambergii*. The species description (Neuhoff 1926) certainly refers to *E. glandulosa* as redefined here. We place *E. grambergii* in the synonyms of *E. glandulosa*.

#### Exidia impressa

Taxon classificationAnimaliaAuricularialesAuriculariaceae

(Pers.) Fr., Systema Mycologicum 2: 226, 1822

6E45EB46-4940-5BB8-9512-A0C84DB571A0

Figs 19E, 20C, 22

≡ Tremella impressa Pers., Mycologia Europaea 1: 102, 1822. Neotype (selected here, MBT 10031435). France. Aveyron: Millau, Le Monna, Laburnum sp. (dead hanging branch), 23 Feb 2025, Spirin 18143* (H).

##### Description.

Basidiomata annual, cupulate-orbicular, up to 3 cm in diam., narrowly attached, up to 0.5 cm thick, gelatinous. Hymenial surface even or indistinctly folded, rarely bearing spine-like outgrowths up to 0.3 mm long, dark reddish-brown to brownish-black, in dry condition completely black. Abhymenial surface rough, covered by tightly arranged dots, brownish-black. Margin free, concolourous with hymenial surface, entire, even or indistinctly lobate.

Hyphal structure monomitic, hyphae clamped; context hyphae hyaline or yellowish, with a variably thickened, gelatinized wall, interwoven, anastomosing, occasionally encrusted, 1.5–7 μm in diam., sometimes inflated and then reaching up to 15 μm in diam., subhymenial hyphae hyaline or yellowish, thin-walled or with a distinct wall, predominantly ascending, 1.5–3 μm in diam., basidia-bearing hyphae thin-walled or with a distinct wall, 2–5 μm in diam., often short-celled. Hyphidia of two types: a) hymenial hyphidia, with a hyaline, narrowly clavate or nearly hyphoid stem, 3–5 μm in diam., and predominantly hyaline, usually rather short, dendroid or coralloid apical branches, 1–2 μm in diam.; b) deeply rooted hyphidia, with a hyaline or yellowish-brownish, often encrusted hyphoid stem, 3–8 μm in diam., and yellowish or brownish, usually rather long apical branches, 1–1.5 μm in diam., embedded in yellowish-brownish gelatinous matrix and forming a continuous layer up to 100 μm thick. Probasidia ellipsoid-ovoid, moderately tapering, or clavate, 10–17 × 3–7 μm. Basidia four-celled, narrowly ellipsoid-ovoid or broadly clavate, often obliquely septate, (13.5–) 15–20 (–21) × (5.1–) 5.4–7.3 (–7.4) μm (n = 20/1), sterigmata up to 50 × 1–2 μm. Basidiospores cylindrical to allantoid, slightly or distinctly curved, (8.8–) 9.0–12.7 (–13.1) × (3.0–) 3.1–4.1 (–4.2) μm (n = 30/1), L = 11.01, W = 3.52, Q = 3.16.

**Figure 22.**
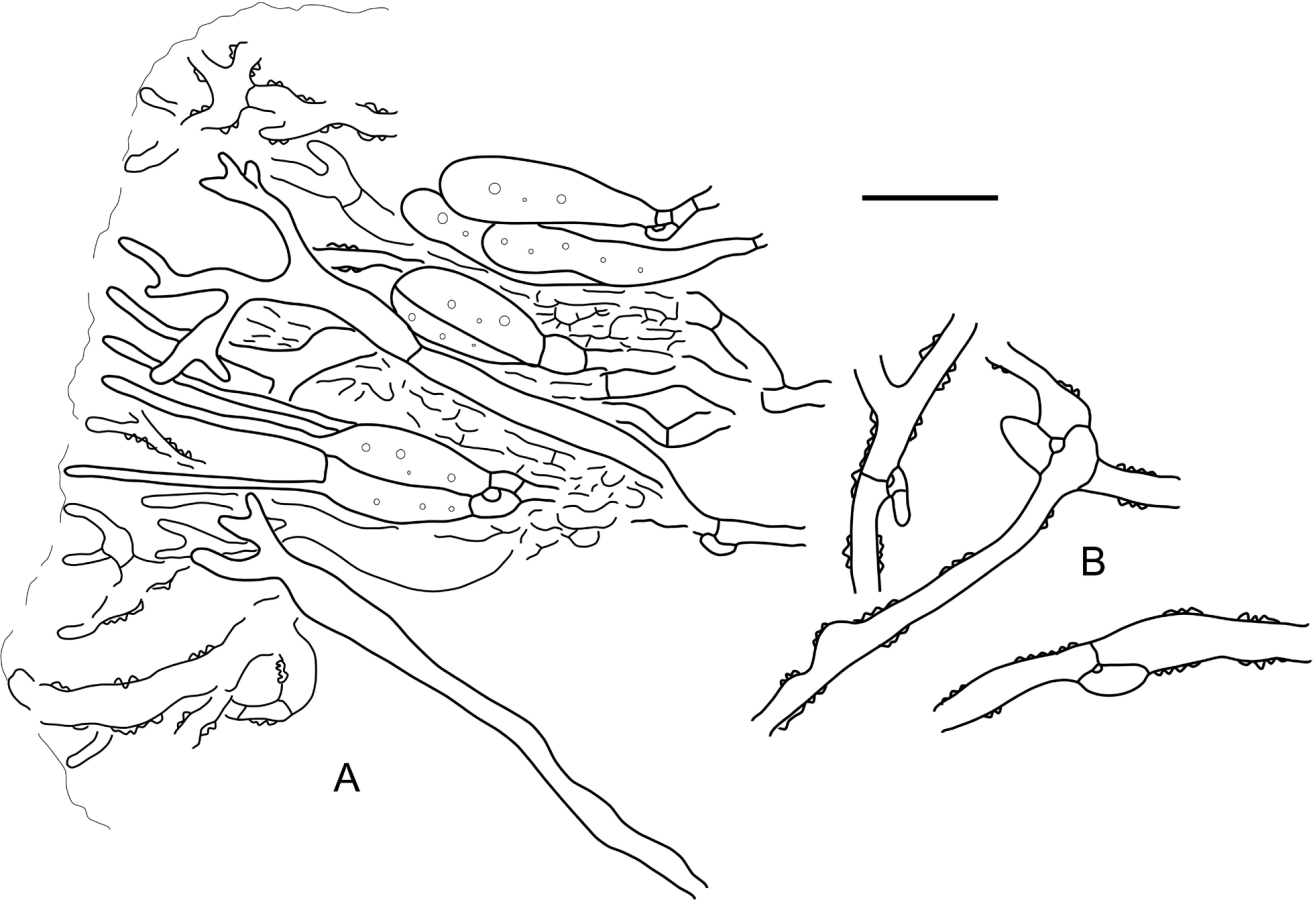
Microstructures of *Exidia impressa* (neotype): **A** subhymenial hyphae and hymenial cells; **B** context hyphae. Scale bar: 10 μm.

##### Remarks.

This species was described by Persoon (1822) as *Tremella impressa*. The protologue referred to another, provisional name, *T. rufescens* Ehrenb. Fries (1822) sanctioned *T. impressa* and moved it to *Exidia* although he had never studied it and simply repeated Persoon’s description. Since then, the identity of *E. impressa* has remained obscure. Bourdot and Galzin (1927) accepted it as a good species. However, their description seemingly refers to another species, *E. straminea* (see below). Neuhoff (1936a) placed *E. impressa* in the synonyms of *E. truncata* (= *E. glandulosa* in the present study), and this synonymy was later accepted by Donk (1966).

In our opinion, Neuhoff’s species concept cannot be accepted because it contradicts some important elements mentioned in the protologue of *T. impressa*. First, Persoon (1822: 102) stated that his species becomes dark reddish-brown in dry condition (“sicca … rufo fusca fit”). Further, he specified that it is completely smooth after being rehydrated, and its margin is faintly plicate (“humectata tota laevis … marginem parum plicata”, ibid.). All these features clearly rule out *E. glandulosa* and *E. truncata*: neither of them show reddish tints, nor is the surface of their basidiomata smooth. Moreover, the basidiome edge of *E. glandulosa* is clearly folded and sometimes lobate. In *E. truncata*, as redescribed below, it remains entire and more or less even. The type material of *E. impressa* is not extant, and we therefore designate a recent, sequenced collection from France as a neotype of *T. impressa*. In all important aspects, it agrees with the descriptions of Persoon and Fries.

Morphologically, *E. impressa* is strongly reminiscent of species in the *E. recisa* complex. Due to its dark, opaque basidiomata, it can be easily separated from two other similar species in Europe, *E. recisa* and *E. straminea*. These two species have lighter-colored, semitranslucent basidiomata when fresh. Some individuals of *E. impressa* possess distinct, sharp projections on the hymenial surface. In contrast, the hymenial surface is completely smooth or only indistinctly warted in *E. recisa* and *E. straminea*. However, the presence of these outgrowths is inconsistent. In a collection of *E. impressa* containing multiple basidiomata, some of them may be completely devoid of the hymenial projections. Moreover, hyphidia of *E. impressa* are of two types – of subhymenial and tramal origin, and they are more or less clearly differentiated from each other. In *E. recisa* and especially in *E. straminea*, all kinds of transitions can be found between hymenial and deeply rooted hyphidia. In Asia, *E. impressa* can be confused with *E. yadongensis* and *E. zelleri*. All three species have dark, cupulate-orbicular basidiomata and similar host preferences, and they may occur in the same habitats. *Exidia zelleri* can be distinguished from *E. impressa* and *E. yadongensis* via its large basidiospores and (if sterile) much bigger basidia. Differences between *E. impressa* and *E. yadongensis* are discussed under the latter species.

We studied and sequenced three collections of *E. impressa*. They are morphologically very uniform but their ITS sequences show 5–6 bp difference from each other. All these positions are in the ITS2 region. We preliminarily accept them as a sign of the infraspecific geographic variation. A much denser sampling from different parts of temperate Eurasia is necessary to prove otherwise.

#### Exidia inornata

Taxon classificationAnimaliaAuricularialesAuriculariaceae

Cazenave & G. Gruhn, Bulletin de la Société Mycologique de France 140: 219, 2024

74193FDE-0917-586C-BE24-88E91EB2F2A8

Fig. 19F

##### Holotype.

France • Hautes-Pyrénées: Séméac, Bois de Labarthe, *Quercus* sp., 26 Feb 2023, Cazenave (LIP GG-240916-002*, isotype – H, studied).

##### Description.

Basidiomata annual, button-shaped or adpressed-orbicular, up to 4 cm in diam., broadly attached, often in groups and partly fusing together, up to 1.5 cm thick, gelatinous. Hymenial surface variably folded, occasionally minutely warted, dark brownish-gray to brownish-black, in dry condition completely black. Abhymenial surface smooth, concolourous with hymenial surface. Margin free, concolourous with hymenial surface, entire, even or indistinctly lobate.

Hyphal structure monomitic, hyphae clamped; context hyphae hyaline or very pale grayish, thin- to slightly thick-walled, interwoven, anastomosing, occasionally encrusted, 1–6 μm in diam., subhymenial hyphae hyaline or grayish-brown, thin- to slightly thick-walled, ascending or interwoven, 1.5–4 μm in diam., some hyphae short-celled and inflated, up to 6–7 μm in diam., basidia-bearing hyphae thin- to slightly thick-walled, 2–5 μm in diam., occasionally irregularly inflated and then up to 6–8 μm in diam. Hyphidia with a grayish or brownish, hyphoid or nodulose stem, 2–9 μm in diam., and hyaline or brownish-gray apical branches, 1–2 μm in diam., forming a continuous layer up to 25 μm thick. Probasidia broadly clavate, more rarely tapering, 14–22 × 7.5–10.5 μm. Basidia four-celled, ellipsoid-ovoid to subglobose, (14–) 14.5–19.5 (–20) × (10.2–) 10.3–13.5 (–13.9) μm (n = 20/1), sterigmata up to 20 × 2.5–3 μm. Basidiospores cylindrical to broadly cylindrical, slightly or distinctly curved, (10.0–) 10.1–15.8 (–16.2) × (4.5–) 4.8–6.2 (–6.3) μm (n = 30/1), L = 13.15, W = 5.47, Q = 2.41.

##### Remarks.

*Exidia inornata* was recently described as a sister species of *E. nigricans* (= *E. pithya* in the present study) (Gruhn et al. 2024). Morphologically, it differs from the latter species due to the lack of projections on the hymenial surface and in having wider basidiospores. This species is so far known only from the southern part of France. Collections of *E. friesiana* from deciduous hosts mentioned by Bourdot and Galzin (1927) may actually belong to *E. inornata* instead. See also notes on *E. benieri* under insufficiently known species.

#### Exidia japonica

Taxon classificationAnimaliaAuricularialesAuriculariaceae

Lloyd, Mycological Writings 5: 599, 1916

678F2BF7-95D6-526F-AA68-FF691015BE10

= Tremellochaete japonica (Lloyd) Raitv., Eesti NSV Teaduste Akadeemia Toimetised 13: 30, 1964.
Exidia japonica is the generic type of Tremellochaete (Raitviir 1964; Malysheva and Spirin 2017). According to our phylogenetic analyses (Figs 1, 2), Tremellochaete must be treated as a part of the redefined Exidia. Thus, the original binomial is the correct name for this species.

##### Holotype.

Japan. Sendai, 3 Nov 1915, Yasuda 356 (TNS 203268, studied).

#### Exidia pithya

Taxon classificationAnimaliaAuricularialesAuriculariaceae

Fr., Systema Mycologicum 2: 226, 1822

B80B37C8-3F71-5AA1-ADB9-F4EF99E00BF6

Figs 19G, 19H, 20E, 20F

= Tremella nigricans With., A Botanical Arrangement of All the Vegetables, Naturally Growing in Great Britain 2: 732, 1776.
= Exidia nigricans (With.) P. Roberts, Mycotaxon 109: 220, 2009.
= Exidia plana Donk, Persoonia 4: 228, 1966.

##### Neotype

**(designated here, MBT 10031436)**. Sweden • Bohuslän: Hönö, Ersdalsvägen, *Pinus sylvestris* (fallen corticated log), 12 Aug 2024 Spirin 17359* (GB) (Figs 19G, 19H, 20E, 20F).

##### Description.

Basidiomata annual, at first cushion- or button-shaped, then adpressed-orbicular or more rarely nearly cerebriform, up to 5 cm in diam., broadly attached, in large groups, finally often fusing together and reaching 30 cm in the longest dimension, up to 1.5 cm thick, gelatinous. Hymenial surface uneven or variably folded, as a rule covered by rather regularly arranged spike-like black projections up to 0.2 mm long, at first gray to brownish-gray, later brownish-black, in older basidiomata sometimes fading to yellowish-brown, in dry condition completely black. Abhymenial surface smooth or near so, concolourous with or slightly paler than hymenial surface. Margin free, concolourous with hymenial surface, entire, even or lobate.

Hyphal structure monomitic, hyphae clamped; context hyphae hyaline to grayish, occasionally encrusted by brownish deposits, thin- to slightly thick-walled, interwoven or subparallel, anastomosing, 0.5–6 μm in diam., subhymenial hyphae hyaline to grayish or brownish, thin-walled or with a distinct wall, ascending or interwoven, 1–3.5 μm in diam., occasionally irregularly inflated (up to 7.5 μm in diam.), basidia-bearing hyphae 2–5.5 μm in diam. Hyphidia with a brownish, occasionally irregularly inflated hyphoid stem, 2–10.5 μm in diam., and hyaline or brownish apical branches, 0.5–1 μm in diam., forming a continuous layer up to 50 μm thick. Probasidia bullet-shaped or broadly clavate, 10.5–23 × 4–10 μm. Basidia four-celled, ellipsoid-ovoid, (10–) 11.5–20.5 (–21.5) × (7.9–) 8.1–11.8 (–12.1) μm (n = 160/14), sterigmata up to 25 × 1–2.5 μm. Basidiospores narrowly to broadly cylindrical, more rarely allantoid, slightly or moderately curved, (8.1–) 8.3–16.7 (–19.7) × (3.1–) 3.2–5.2 (–5.3) μm (n = 560/19), L = 9.55–14.43, W = 3.59–4.61, Q = 2.49–3.49.

##### Remarks.

As redefined here, *E. pithya* is morphologically most similar to *E. friesiana*. Both species produce black or nearly black, adpressed-orbicular basidiomata with smooth abhymenial surface, soon fusing together and making up compound, irregularly shaped fructifications. The main macroscopic differences between the two species are a more distinctly folded hymenial surface of *E. pithya* compared to *E. friesiana* and the presence of sharp or blunt hymenial projections in the first species. Microscopically, *E. pithya* differs from *E. friesiana* in having more irregularly arranged subhymenial hyphae (often partly ascending and partly interwoven in the same fruitbody) and narrower and as a rule shorter basidiospores. Additionally, *E. pithya* occurs on both angiosperms and conifers, while *E. friesiana* is restricted to freshly fallen logs and branches of *Abies* and *Picea* spp. (as well as *Pinus* in the Mediterranean).

Up to now, *E. pithya* was believed to be a conifer-dwelling species which was differentiated from the similar *E. glandulosa* sensu Neuhoff (= *E. nigricans* after Roberts 2009) by its host preference. The latter species was described as growing exclusively on deciduous trees (Raitviir 1970; Hansen and Knudsen 1997). However, some authors noted that the so-called *E. glandulosa* can also be found on conifers (Neuhoff 1936a; Pilát 1957; Torkelsen 1972; Wojewoda 1981), and that its only reliable trait versus *E. pithya* is the presence of hymenial outgrowths. This concept, as well as the application of the name *E. pithya* to the species without these projections came from Neuhoff (1936a). In our opinion, this viewpoint cannot be maintained because it explicitly contradicts Fries’ description of *E. pithya*. In his sanctioning treatment, Fries (1822: 226) added the following remark to *E. pithya*: “Ad truncos et ramos emortuos pineos, etiam mihi minute papillifera”. The reference to minutely papillose hymenophore clearly excludes *E. pithya* in the sense of Neuhoff (= *E. friesiana* in the present study). Moreover, no verified records of the latter species on *Pinus* in temperate or boreal Europe are known to us. On the contrary, *E. glandulosa* sensu Neuhoff (= *E. nigricans*) occurs in the south-western part of Sweden on both angiosperms and conifers, including pine. We therefore designate one of the sequenced Swedish specimens collected on *Pinus* as a neotype of *E. pithya*. *Tremella nigricans* (= *E. nigricans*) is an older name for the species reinstated here as *E. pithya*. However, it has never been sanctioned. Thence, *E. pithya* has a priority over *T. nigricans*.

*Exidia pithya* is widely distributed in the northern hemisphere. Its differences from *E. friesiana* are listed above. However, the boundaries between the two species need further examination. The specimen Burdsall 12029, morphologically matching *Exidia pithya* and the only North American sample sequenced for more than one marker, shows a conflicting genetic pattern. Its ITS sequence is identical to the European *E. pithya* whereas its *TEF1* is the same as in *E. friesiana*. When two unlinked markers fully correspond to organisms from two different species, this usually indicates a very recent introgression event. Other processes, such as incomplete lineage sorting, are less likely – if these markers had been inherited from the ancestral population prior to the species split, enough time should have passed for at least some sequence divergence to accumulate. Denser sampling from North America and additional unlinked markers, ideally supported by mating tests, are needed to clarify this issue.

Another widely distributed species similar to *E. pithya* is *E. spiculata*. Like *E. friesiana*, it is totally devoid of the hymenial projections which are characteristic of *E. pithya*. However, in some collections of *E. pithya* they can be very sparse or completely lacking, although the number of these deviating specimens is relatively low. See further remarks under *E. spiculata*.

#### Exidia plumbescens

Taxon classificationAnimaliaAuricularialesAuriculariaceae

(Burt) Spirin comb. nov.

3342BF43-8EC0-54E6-B4A4-5B2FCCF320AD

862477

Figs 19I, 20D

≡ Sebacina plumbescens Burt, Annals of the Missouri Botanical Garden 3: 241, 1916 (nomen novum for S. plumbea Burt).
= Sebacina plumbea Burt, Annals of the Missouri Botanical Garden 2: 765, 1915 (nom. illeg., non S. plumbea Bres. & Torrend). Holotype. USA. Washington: Klickitat Co., Bingen, Populus trichocarpa (fallen decorticated log), 18 Nov 1902, Suksdorf 862 (H – isotype, studied).
= *Sebacina burtii* Trotter, Sylloge Fungorum 23: 573, 1925.
= Exidiopsis plumbescens (Burt) K. Wells, Lloydia 20: 53, 1957.
= Exidiopsis succinea K. Wells & Raitv., Transactions of the British Mycological Society 89: 341, 1987. Holotype. Russia. Krasnoyarsk Reg.: Yenisseysk Dist., Yartsevo, Salix fragilis, 10 Aug 1958, Parmasto (TAAM 7008*, studied).

##### Description.

Basidiomata annual, effused, up to 10 cm in the widest dimension, gelatinous, opalescent, 0.5–1.5 mm thick. Hymenial surface smooth or indistinctly tuberculate, grayish to pale ochraceous in fresh condition, sometimes with reddish hues, corneous and amber-yellow to reddish-brown in herbarium; mineral inclusions abundant, white, first small and visible only under lens, in senescent basidiomata prominent and easily detectable. Margin rather clearly delimited, adnate, gradually thinning, concolourous with hymenial surface.

Hyphal structure monomitic, hyphae clamped; subicular hyphae hyaline, thin-walled or with variably thickened, gelatinized walls, tightly interwoven or subparallel, anastomosing, 1.5–4 μm in diam., subhymenial hyphae hyaline, thin-walled, predominantly ascending, tightly arranged, 1.5–3.5 μm in diam., occasionally inflated up to 6 μm in diam., basidia-bearing hyphae well-differentiated, 3–5 μm in diam., occasionally showing a zigzag-like branching pattern. Crystals present as large, rosette-like agglomerations, up to 25 μm in diam. Hyphidia richly branched, with a hyphoid or subfusiform stem, 15–35 × 5–7.5 μm, and hyaline apical branches, 1–1.5 μm in diam., partly covering basidia. Probasidia broadly clavate to subglobose, 10–18 × 6–10.5 μm. Basidia four-celled, ellipsoid-ovoid, more rarely subglobose, sessile, occasionally slightly tapering to the base, (14–) 15–22 (–23) × (10.0–) 10.3–17.4 (–18.0) μm (n = 70/7), thin- or slightly thick-walled, exposed or embedded, in older hymenium often surrounded by gelatinous substance, sterigmata up to 35 × 2.5–3 μm. Basidiospores thin-walled, narrowly to broadly cylindrical, often distinctly curved, (11.7–) 12.1–19.4 (–20.2) × (4.3–) 4.5–7.8 (–8.0) μm (n = 240/8), L = 14.11–16.17, W = 5.19–6.62, Q = 2.21–3.13.

##### Remarks.

This species was first described as *Sebacina plumbea* based on a single specimen from the US North-West (Burt 1915). Burt’s name turned out to be a younger homonym of *S. plumbea* described by Bresadola and Torrend two years earlier (see above, under synonyms of *D. thuretiana*). Consequently, it was validated by Burt (1916) as *S. plumbescens*. Trotter (Saccardo and Trotter 1925) also reintroduced *S. plumbea* under a new name, *Sebacina burtii*. However, it must be rejected as a homotypic synonym of *S. plumbescens*. This species was accepted by Wells (1957) as a member of *Exidiopsis* and combined therein. A few years later, Wells (1961) included *E. plumbescens* in his broadly interpreted *E. grisea* and then resurrected it as a good species again (Wells and Wong 1985). We studied type material of both *S. plumbescens* and *Exidiopsis succinea* described from Siberia (Wells and Raitviir 1987) and concluded that they are conspecific. DNA data place *S. plumbescens* in the redefined *Exidia* (Figs 1, 2).

*Exidia plumbescens* is widely distributed in the northern hemisphere although it is not common. In boreal forests, it occurs on dead branches and stems of *Salix* spp., more rarely on *Populus* spp. In more southern areas, it may inhabit other angiosperm trees (*Fagus* and *Ulmus*). Wells and Wong (1985) gave a long list of substrates for *Exidiopsis plumbescens* from the North American Pacific coast. It is, however, not clear to us whether all these records refer to *Exidia plumbescens* sensu typi.

*Exidia plumbescens* is one of four entirely effused *Exidia* s. str. species dealt with in this paper. Of them, *E. delita*, differs from *E. plumbescens* in having thinner basidiomata, narrower hyphidia and on average smaller basidia and basidiospores (see above). Two other European species, *E. repleta* and *E. saturata*, differ from *E. plumbescens* in having thicker basidiomata covered by easily removable, whitish-grayish deposit. Both mentioned species possess a distinct epihymenial layer and they have cystidia. Moreover, hyphae of *E. plumbescens* are more or less evenly outlined while they are often clearly inflated in *E. repleta* and *E. saturata*.

*Exidia plumbescens* can also be confused with effused forms of *Descidia thuretiana*. The easiest way to distinguish between them is to check the basidiome margin: it is always adnate, gradually thinning in *E. plumbescens* and elevated, often partly detaching from the substrate in *D. thuretiana*. Spirin et al. (2025) showed that earlier records of *Exidiopsis livescens* from North Europe belong to *Exidiopsis succinea* (*Exidia plumbescens* here). Previous reports and descriptions of *Exidiopsis griseobrunnea* from Norway (Torkelsen 1972; Hansen and Knudsen 1997) also refer to *E. plumbescens*.

#### Exidia recisa

Taxon classificationAnimaliaAuricularialesAuriculariaceae

(Ditm.) Fr., Systema Mycologicum 2: 223, 1822

28404581-2D8E-5024-941E-0753E4EDBC18

Figs 20G, 23A, 23B

≡ Tremella recisa Ditm. in Sturm, Deutschlands Flora, Abt. 3, 1 (1): 27, 1817. Lectotype (iconotype) (selected here, MBT 10031437). Table 13 (Tremella recisa) in Sturm, Deutschlands Flora, Abtheilung 3, 1 (1), 1817. Epitype (selected here, MBT 10031438). Finland. Etelä-Häme: Muurame, Niittyaho, Salix fragilis, 18 Mar 2017, Laaka-Lindberg 1-180317* (H).
= Exidia brunneola P. Karst., Bidrag till Kännedom av Finlands Natur och Folk 48: 450, 1889. Holotype. Finland. Etelä-Häme: Tammela, Mustiala, Populus nigra, 2 Mar 1870, Karsten 2138 (H6049699, studied).

##### Description.

Basidiomata annual, cupulate-orbicular, up to 3 cm in diam., rarely up to 5 cm in diam., narrowly attached, later partly fusing together but remaining separable, up to 1 cm thick, gelatinous. Hymenial surface even or variably folded, rarely bearing scattered granular projections up to 0.1 mm long, at first ochraceous- to reddish-brown, later dark brown, in dry condition darkening to nearly black. Abhymenial surface rough, covered by tightly arranged dots, reddish-brown or dark brown. Margin free, concolourous with hymenial surface, entire, even or indistinctly lobate, in older basidiomata sometimes indistinctly incised.

**Figure 23.**
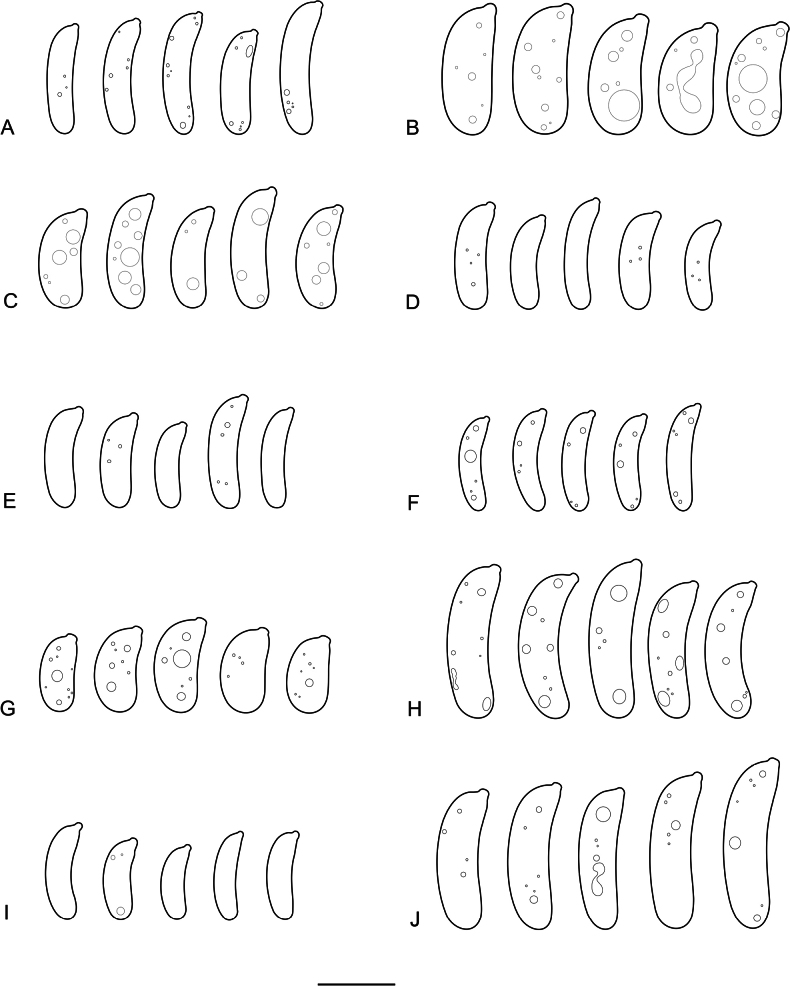
Basidiospores: **A***Exidia recisa* (epitype); **B***E. repleta* (holotype); **C***E. saturata* (holotype); **D***E. semiorbis* (holotype); **E***E. spiculata* (isotype of *E. inflata*); **F***E. straminea* (epitype); **G***E. tanzanica* (holotype); **H***E. truncata* (neotype); **I***E. umbrinella* (lectotype); **J***E. zelleri* (Spirin 17780). Scale bar: 10 μm.

Hyphal structure monomitic, hyphae clamped; context hyphae hyaline or yellowish-brownish, occasionally encrusted, thin- to slightly thick-walled, interwoven or subparallel, anastomosing, 1–6.5 μm in diam., subhymenial hyphae hyaline or yellowish-brownish, occasionally encrusted, thin- to slightly thick-walled, predominantly interwoven, 1–4 μm in diam., basidia-bearing hyphae with a distinct wall, 1.5–4 μm in diam. Hyphidia with a hyaline or brownish, often encrusted, hyphoid or nodulose stem, 2–12 μm in diam., and hyaline apical branches, 0.5–1.2 μm in diam., covered by yellowish-brown gelatinous matrix and forming a continuous layer up to 80 μm thick. Probasidia distinctly tapering or more rarely clavate, 10.5–25 × 5–9 μm. Basidia four-celled, ellipsoid-ovoid, often somewhat tapering to the base, sometimes obliquely septate, (12–) 12.5–18 (–19) × (6.2–) 6.3–10.8 (–11.5) μm (n = 60/6), sterigmata up to 35 × 1.5–2.5 μm. Basidiospores narrowly cylindrical to allantoid, distinctly curved, (9.2–) 11.0–17.9 (–19.2) × (2.8–) 2.9–4.2 (–4.3) μm (n = 240/8), L = 13.14–15.82, W = 3.15–3.83, Q = 3.15–4.48.

##### Remarks.

*Exidia recisa* is a common species in Europe. It mostly inhabits branches and thin stems of *Salix* spp. in various habitats but rarely occurs on hanging or just fallen twigs of other angiosperms (*Padus*, *Populus*, *Tilia*, and *Ulmus*). The occurrence on *Salix* and on average longer basidiospores help to differentiate it from other European representatives of the *E. recisa* complex, i.e. *E. impressa*, *E. straminea* and *E. umbrinella*. However, specimens of *E. recisa* from the other host trees, especially those with shorter basidiospores, can be easily mistaken for *E. impressa* and *E. straminea*. In these cases, additional morphological characters (basidiome color, the presence of hymenial projections, as well as the shape of hyphidia) should be used for species identification. The eastern distribution limits of *E. recisa* are unknown. According to our data, the easternmost record of this species is in Khakassia, Central Siberia.

The species was first introduced by Ditmar (in Sturm 1817) as *Tremella recisa* and then moved to *Exidia* by Fries (1822). No authentic material of *T. recisa* is known to us. We therefore select the original illustration by Ditmar as the lectotype of this species and supplement it with a newly selected and sequenced epitype. *Exidia brunneola* was described from Finland based on a single collection from a poplar tree (Karsten 1889) which is not native to the country. However, its morphological traits do not separate it from other *E. recisa* specimens studied by us. We therefore relegate *E. brunneola* to the synonyms of *E. recisa*.

#### Exidia reflexa

Taxon classificationAnimaliaAuricularialesAuriculariaceae

F. Wu, L.F. Fan & S.Y. Ye, Mycological Progress 19: 862, 2020

74A3210F-4601-5E89-9FC6-2A16E17CB340

##### Holotype.

China • Jilin: Antu, Changbai, *Tilia* sp., 21 Sep 2019, Dai 20861* (BJFC).

*Exidia reflexa* is a member of the *E. pithya* complex (Figs 10, 11) distributed in temperate East Asia. It differs from other similar species of the *E. pithya* group in having the smallest basidiospores, 9–10 × 2–2.5 μm. *Exidia reflexa* was described from China (Ye et al. 2020) and is reported here from Far East Russia.

#### Exidia repleta

Taxon classificationAnimaliaAuricularialesAuriculariaceae

Spirin & Viner sp. nov.

1937603E-4290-500E-B2AD-1FCC82A486F8

864366

Fig. 23B

##### Holotype.

France • Var: Toulon, Mont Faron, Plateau des Jésuits, 43.1437; 5.9248, *Quercus coccifera* (dead hanging branch), 23 Jan 2026, Spirin 18748* (H, isotype – PC).

##### Etymology.

Repletus (Lat., adj.) – replete, filled; in reference to water-soaked basidiomata.

##### Description.

Basidiomata annual, effused, first appearing as small, gregarious frustulae, 0.5–1.5 mm in diam., then partly fusing together (but remaining discernible) and forming compound basidiomata, up to 3 cm in the longest dimension, gelatinous, opaque, 0.1–0.2 mm thick. Hymenial surface smooth or indistinctly tuberculate, entirely covered by grayish-white to watery-gray, easily removable pruina, occasionally with grayish-brown flecks, in older individuals partly disappearing and showing a darker, brownish inner layer. Margin clearly delimited, adnate, gradually thinning, concolourous with hymenial surface.

Hyphal structure monomitic, hyphae clamped; subicular hyphae hyaline, slightly thick-walled, mostly interwoven, anastomosing, 1.5–5 μm in diam., occasionally strongly inflated (up to 8–10 μm in diam.), subhymenial hyphae hyaline or yellowish-brownish, thin- to slightly thick-walled, predominantly ascending, 1.5–5 μm in diam., occasionally inflated up to 6–9 μm in diam., basidia-bearing hyphae well-differentiated, 2–6 μm in diam., often showing a zigzag-like branching pattern. Crystals accidental, angular, small. Cystidia occasionally present, hyaline or brownish, thin- or slightly thick-walled, narrowly clavate or gradually tapering to the apex, rarely constricted in the middle, 23–44 × 6–9 μm, sometimes with one or two inner septa. Hyphidia richly branched, with a hyphoid or nodulose stem, 20–60 × 2–8 μm, and hyaline or grayish-brownish apical branches, 1–1.5 μm in diam., covering basidia and forming a continuous layer up 40 μm thick. Probasidia clavate or somewhat tapering, 15–28 × 6–9 μm. Basidia four-celled, ellipsoid-ovoid, more rarely broadly ellipsoid, sessile, (13–) 14–21 (–22.5) × (9.3–) 9.6–15.9 (–16.2) μm (n = 25/2), thin-walled or with a distinct wall, embedded, occasionally with a tapering, stalk-like base, up to 8 × 3 μm, sterigmata up to 35 × 2–3.5 μm. Basidiospores thin-walled, broadly cylindrical to cylindrical, often slightly curved, (11.0–) 11.2–15.1 (–15.3) × (5.0–) 5.1–6.2 (–6.3) μm (n = 60/2), L = 12.72–13.00, W = 5.62–6.29, Q = 2.08–2.27.

##### Remarks.

*Exidia repleta* is introduced here based on three collections from the southern part of France, all from dead, hanging branches of *Quercus coccifera*. It is a resupinate species closely related to *E. saturata* described below. The species show small (3–5 bp) differences in the ITS region but their *RPB2* sequences are much more divergent (up to 25 bp difference). Combined with clear morphological differences, we feel confident in introducing them as two separate species. Macroscopically, *E. repleta* differs from *E. saturata* in having smaller basidiomata and grayish-white (without bluish or violet hues) hymenial surface. In the microscope, the two species are distinguishable primarily due to their spore shape and size.

*Tegmenticium peritrichum* redescribed below shows a certain morphological similarity to *E. repleta*, mainly due to its gelatinous basidiomata covered by grayish removable pruina, as well as predominantly broadly cylindrical basidiospores. However, it can be separated from *E. repleta* (as well as *E. saturata*) due to its corticioid basidiomata and more evenly outlined, thin-walled subicular and subhymenial hyphae. Moreover, basidia-bearing hyphae of *T. peritrichum* are mostly poorly differentiated while they are easily detectable in the two *Exidia* spp. The epihymenial layer is rather loose and rarely exceeds 20 μm thick in *T. peritrichum*; in *E. repleta* and *E. saturata*, it is quite dense and at least 30–40 μm thick. Differences of *E. repleta* from *E. delita* and *E. plumbescens* are mentioned under the latter species.

#### Exidia saturata

Taxon classificationAnimaliaAuricularialesAuriculariaceae

Spirin & Viner sp. nov.

12A0AC9A-5D3B-5562-8DC5-1D0BAE8D0E38

864367

Figs 20H, 23C

##### Holotype.

France • Var: Toulon, Mont Faron, Plateau des Jésuits, 43.1437; 5.9248, *Rosmarinus officinalis* (dead stem), 23 Jan 2026, Spirin 18746* (H, isotype – PC).

##### Etymology.

Saturatus (Lat., adj.) – saturated; in reference to hygroscopic basidiomata.

##### Description.

Basidiomata annual, effused, first small, gregarious, adpressed-orbicular, 0.5–1.5 mm in diam., then partly or completely fusing together and forming compound basidiomata, up to 15 cm in the longest dimension, gelatinous, opaque, 0.2–0.5 mm thick. Hymenial surface smooth or tuberculate, entirely covered by bluish- or violet-gray, easily removable pruina, often with brown flecks, in older basidiomata partly disappearing and showing a darker, vinaceous-brownish inner layer. Margin clearly delimited, adnate, often somewhat elevated and lobate, concolourous with hymenial surface.

Hyphal structure monomitic, hyphae clamped; subicular hyphae hyaline or brownish, thin- to slightly thick-walled, mostly interwoven, anastomosing, 1–5 μm in diam., occasionally strongly inflated (up to 6–8 μm in diam.), subhymenial hyphae hyaline or brownish, thin-walled or with a distinct wall, predominantly ascending, 1.5–3 μm in diam., occasionally inflated up to 4–5 μm in diam., basidia-bearing hyphae well-differentiated, 2–3 μm in diam., often showing a zigzag-like branching pattern. Crystals accidental, angular, small. Cystidia occasionally present, hyaline or grayish-brownish, thin- or slightly thick-walled, narrowly clavate or gradually tapering to the apex, occasionally constricted, 18–52 × 5–11 μm. Hyphidia richly branched, with a hyphoid or nodulose stem, 20–50 × 2.5–7 μm, and hyaline or brownish apical branches, 0.8–1.5 μm in diam., covering basidia and forming a continuous layer up 60 μm thick. Probasidia broadly clavate or somewhat tapering, 13–19 × 5–9 μm. Basidia four-celled, ellipsoid-ovoid or broadly ellipsoid, sessile, (13–) 13.5–17 (–17.5) × (10.1–) 10.2–13.3 (–13.5) μm (n = 20/2), thin-walled, sometimes obliquely septate, embedded, occasionally with a tapering, stalk-like base, up to 5 × 2.5 μm, sterigmata up to 30 × 2–4 μm. Basidiospores thin-walled, narrowly to broadly cylindrical, often distinctly curved, (9.2–) 9.8–14.9 (–15.2) × (3.9–) 4.0–5.1 (–5.2) μm (n = 60/2), L = 12.11–12.67, W = 4.53–4.71, Q = 2.59–2.82.

##### Remarks.

*Exidia saturata* is described here as a sister species of *E. repleta*. It differs from the latter species primarily due to the presence of bluish or violet hues in the basidiome color and in having narrower basidiospores. At large part, the originally adpressed-orbicular basidiomata of *E. saturata* completely fuse together and form rather extensive compound crust-like fructifications, while they mainly remain discernible and normally quite small in *E. repleta*.

*Exidia saturata* and *E. repleta* occur in the same localities in the Mediterranean. Two of our specimens of *E. saturata* were collected from dead stems of *Rosmarinus*. The GenBank sequence AJ427409 labelled as *Sebacina umbrina* D.P. Rogers belongs to *E. saturata*; its source is a specimen collected from *Cistus monspelliensis* in France. In turn, *E. repleta* has been so far found only on *Quercus*.

#### Exidia semiorbis

Taxon classificationAnimaliaAuricularialesAuriculariaceae

Malysheva sp. nov.

8F81AAE3-FB83-5413-93A6-228EF96FB766

862478

Figs 23D, 24A

##### Holotype.

Russia • Primorie Reg.: Shkotovo Dist., Ussuri Nat. Res., Peishula, 43.64769; 132.59026, *Salix* sp. (fallen log), 16 Aug 2011, Malysheva (LE F-262976*).

##### Etymology.

Semiorbis (Lat., noun) – semicircle.

##### Description.

Basidiomata annual, button-shaped or cupulate-orbicular, up to 1 cm in diam., narrowly attached, up to 0.5 cm thick, gelatinous. Hymenial surface even, smooth or rarely bearing scattered granular projections up to 0.1 mm long, pinkish- to reddish-brown, in dry condition dark-brown. Abhymenial surface smooth or covered by scattered minute dots, reddish-brown. Margin free, concolorous with hymenial surface, entire, evenly rounded or indistinctly lobate.

Hyphal structure monomitic, hyphae clamped; context hyphae hyaline, thin-walled or with a distinct wall, interwoven, anastomosing, 2–5 μm in diam., subhymenial hyphae hyaline, thin-walled or with a distinct wall, ascending or interwoven, 2–4 (5) μm in diam., basidia-bearing hyphae 2–5 μm in diam. Hyphidia scattered, with a hyaline, hyphoid or tortuous stem, 2.5–5 μm in diam., and hyaline apical branches, 0.5–2 μm in diam. Probasidia tapering, 15–30 × 5–9.5 μm. Basidia four-celled, broadly clavate to ellipsoid-ovoid, more rarely subglobose, (10–) 11.5–15 (–16) × (6.1–) 6.6–8.2 (–9.0) μm (n = 20/2), sterigmata up to 40 × 2.5–3 μm. Basidiospores allantoid to narrowly cylindrical, distinctly curved, (10.2–) 11.0–15.6 (–16.9) × (2.7–) 2.8–4.2 (–4.3) μm (n = 38/2), L = 12.19–13.95, W = 3.11–3.54, Q = 3.92–3.99.

**Figure 24.**
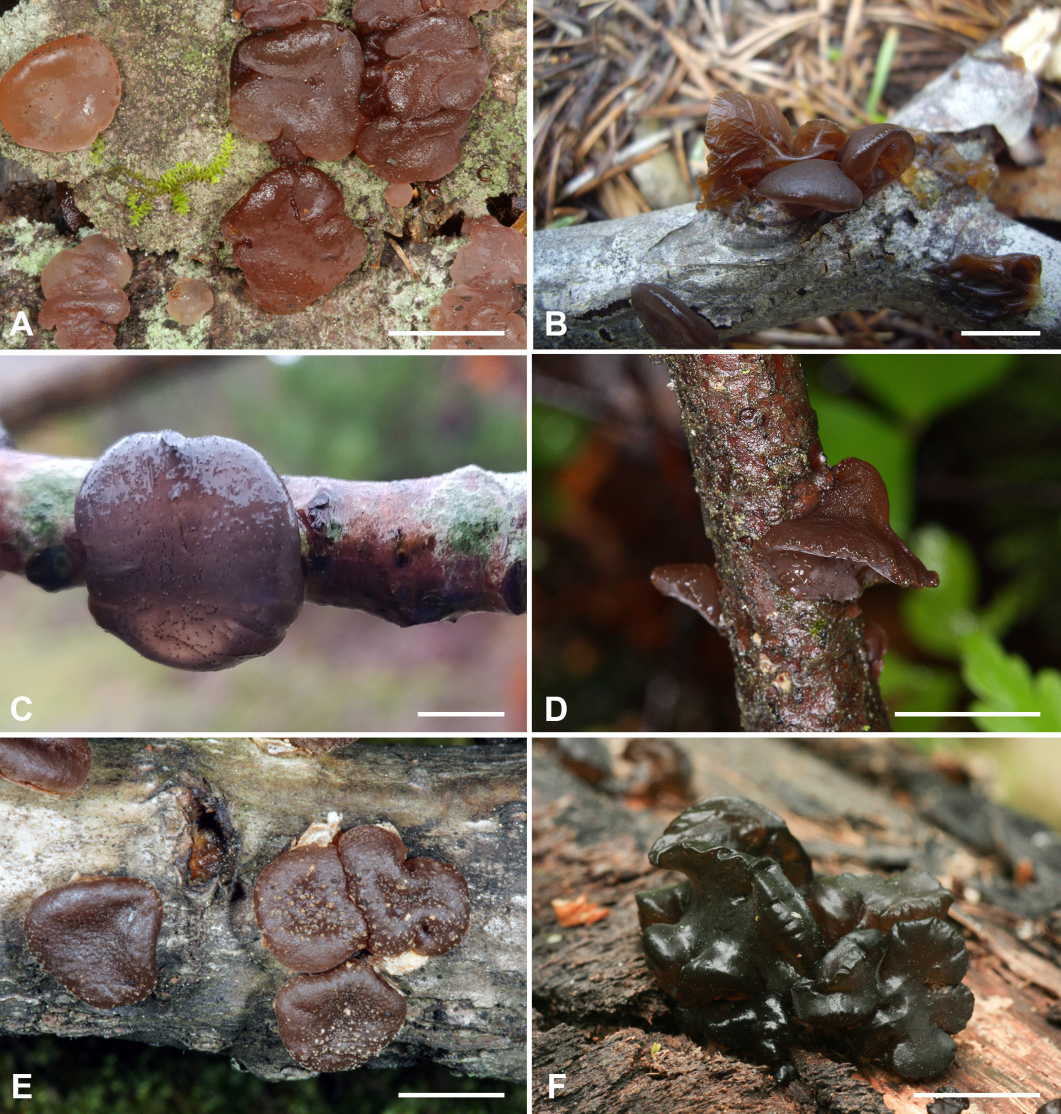
Basidiomata: **A***Exidia semiorbis* (LE F-262977); **B***E. straminea* (epitype); **C***E. truncata* (neotype); **D***E. umbrinella* (LE F-254078); **E***E. uvapassa* (LE 262970); **F***E. zelleri* (LE F-34768). Scale bar: 1 cm.

##### Remarks.

*Exidia semiorbis* is introduced here as a new species from Far East Russia where it inhabits fallen branches of deciduous trees. Morphologically, it is most similar to the East Asian *E. uvapassa*. *Exidia semiorbis* differs from the latter species in having lighter-colored basidiomata and in lacking an epihymenial membrane. Moreover, *E. uvapassa* has much larger basidia than *E. semiorbis*, reaching 25 μm or even more. Phylogenetically, these species are not closely related (Fig. 7). Differences between *E. semiorbis* and the superficially similar *D. repanda* are discussed in the remarks on the genus *Exidia* above.

#### Exidia spiculata

Taxon classificationAnimaliaAuricularialesAuriculariaceae

Schwein., Transactions of the American Mycological Society 4: 185, 1832

83068EC9-0403-5A79-AB35-144CE43C688B

Fig. 23E

= Exidia glandulosa f. populi Neuhoff, Arkiv för Botanik 28A (1): 13. Holotype. Sweden. Uppland: Uppsala, Lassby backar, Populus tremula, 10 Apr 1884, Romell 4388 (S F207857, studied).
= Exidia inflata Cazenave & G. Gruhn, Bulletin de la Société Mycologique de France 140: 214, 2024. Holotype. France. Hautes-Pyrénées: Séméac, Bois de Labarthe, Populus tremula, 26 Feb 2022, Cazenave (LIP GG-240916-001*, isotype – H, studied).

##### Holotype.

USA. Pennsylvania: Bethlehem, *Platanus* sp. (cut surface of log), [no collecting date indicated], Schweinitz 1101 (PH00062438, studied) (Fig. 23E).

##### Description.

Basidiomata annual, at first cushion- or button-shaped, then adpressed-orbicular, up to 2 cm in diam., broadly attached, often in groups, finally fusing together and forming compound effused basidiomata but rarely reaching 4 cm in the longest dimension, up to 0.5 cm thick, gelatinous. Hymenial surface uneven or variably folded, brownish-gray to brownish-black, rarely with indistinct warts normally associated with embedded white mineral inclusions, in older basidiomata sometimes fading to yellowish-brown, in dry condition nearly black. Abhymenial surface smooth or rarely minutely dotted, concolorous with hymenial surface. Margin free, concolourous with hymenial surface, entire, even.

Hyphal structure monomitic, hyphae clamped; context hyphae hyaline or grayish, thin- to slightly thick-walled, interwoven, anastomosing, occasionally encrusted, 0.5–5 μm in diam., sometimes inflated up to 7.5 μm in diam., subhymenial hyphae hyaline or grayish, thin- to slightly thick-walled, predominantly ascending, 1–4 μm in diam., basidia-bearing hyphae thin- to slightly thick-walled, 2–5 μm in diam. Hyphidia with a grayish or brownish, hyphoid or nodulose stem, 2.5–7 μm in diam., and hyaline or brownish-gray apical branches, 0.8–1.5 μm in diam., forming a continuous layer up to 60 μm thick and sometimes covered by masses of angular crystals. Probasidia tapering or clavate, 11–18 × 4–8 μm. Basidia four-celled, ellipsoid-ovoid, (9.5–) 10–14 (–15.5) × (7.1–) 7.3–10.0 (–10.2) μm (n = 50/4), sterigmata up to 15 × 1–2 μm. Basidiospores narrowly cylindrical to allantoid, slightly or distinctly curved, (9.8–) 10.2–15.6 (–15.7) × (2.8–) 2.9–4.2 (–4.3) μm (n = 173/6), L = 11.91–13.60, W = 3.44–3.77, Q = 3.41–3.74, normally aguttulate.

##### Remarks.

*Exidia spiculata* is one of the nigrescent *Exidia* spp. belonging to the *E. pithya* complex (Figs 10, 11). It seems that it is widely distributed in the cold temperate and boreal ecosystems of Eurasia but overlooked. *Exidia spiculata* inhabits dead branches or just fallen, corticated logs of many deciduous trees but with a certain preference for *Salicaceae*. Morphologically and phylogenetically, it is very close to the East Asian *E. subglandulosa* which, however, has wider basidiospores (see below). The superficially similar *E. pithya* has on average wider basidiospores than *E. spiculata*. They are more allantoid and normally aguttulate in the latter species. In *E. pithya*, the spores are always filled with numerous and easily detectable oil droplets (Fig. 19). Moreover, *E. spiculata* never produces spine-like projections on the hymenial surface, while these outgrowths are typically present in *E. pithya*.

*Exidia spiculata* was first described by Schweinitz (1832) from Bethlehem, Pennsylvania. Coker (1920) studied the type material of *E. spiculata* and concluded that it was of no value. On the contrary, Burt (1921) found the type of *E. spiculata* to be identifiable. He was able to find basidiospores and associate the type specimen with a few other collections from the US North-East. Burt stressed another macroscopic feature of *E. spiculata* which was missing in the original description, i.e. the presence of white mineral grains on the hymenial surface, easily detectable without magnification. Both Coker and Burt were misled by Schweinitz’s remark in the protologue that *E. spiculata* has a papillose surface (‘papillis frequentibus in superficie’ – Schweinitz, ibid.). They thought that this detail referred to papillae on the hymenial surface which they could not find in the type specimen. In fact, this part of Schweinitz’s description refers to the abhymenial surface which is, according to our observations, covered by small, densely arranged papillate projections, ca. 50 × 50 μm. The hymenial surface in the type collection is folded but devoid of any other kind of outgrowths.

Neuhoff (1936a) placed *E. spiculata* in the synonyms of *E. glandulosa* sensu auct. (= *E. pithya* in the present study). The same year, he described E. glandulosa f. populi from Sweden (Neuhoff 1936b). In the description of this form, Neuhoff emphasized its minutely dotted abhymenial surface (‘subtus punctato-scabra’), i.e. the same feature mentioned by Schweinitz for his *E. spiculata*, as well as its folded hymenial surface lacking any other projections (‘disco plano, dein subcostato’). The type specimen of E. glandulosa f. populi perfectly matches other specimens of *E. spiculata* studied by us. We should note, however, that we were able to observe the papillose abhymenial surface only in a few specimens of *E. spiculata* (including the holotype) while it is apparently smooth in most collections studied by us. This trait is, therefore, of minor value to distinguish between *E. spiculata* and *E. pithya*. As for easily visible mineral grains, we could find them only in senescent, coalescent individuals of *E. spiculata*. They were both embedded and exposed. Nevertheless, if present, they give an additional clue for recognizing *E. spiculata* vs. *E. pithya* because old specimens of both species are often sterile. Basidiomata of *Exidia pithya* may also contain mineral concretions. However, they are small, visible only under lens, and as a rule embedded.

#### Exidia straminea

Taxon classificationAnimaliaAuricularialesAuriculariaceae

Berk., Hooker’s Journal of Botany 3: 19, 1851

37245CC1-7525-5648-B0C9-B757B32E537E

Figs 23F, 24B

##### Lectotype

**(iconotype) (selected here, MBT 10031440)**. Table I, fig. 4 in Hooker’s Journal of Botany 3, 1851.

Epitype (selected here, MBT 10031441). France. Aveyron: Millau, Le Monna, *Amelanchier ovalis* (dead hanging branch), 23 Feb 2025, Spirin 18148* (H).

##### Description.

Basidiomata annual, cupulate-orbicular, up to 3 cm in diam., narrowly attached, later partly fusing together but remaining separable, up to 1 cm thick, gelatinous, older basidiomata sometimes nearly cerebriform. Hymenial surface even or variably folded, sometimes bearing scattered granular projections up to 0.1 mm long, at first amber-yellow or ochraceous-brownish, later bright reddish-brown, in dry condition darkening to dark brown or brownish-black. Abhymenial surface rough, covered by tightly arranged dots, yellowish-ochraceous to reddish-brown. Margin free, concolourous with hymenial surface, entire, even or indistinctly lobate.

Hyphal structure monomitic, hyphae clamped; context hyphae hyaline, occasionally bearing scattered colorless encrustation, thin- to slightly thick-walled, interwoven, anastomosing, 1–6 μm in diam., subhymenial hyphae hyaline or brownish, occasionally sparsely encrusted, thin-walled or with a distinct wall, ascending or interwoven, 1.5–4 μm in diam., sometimes producing large bubble-like segments up to 15 μm in diam., basidia-bearing hyphae thin-walled or with a distinct wall, 2–5 μm in diam. Hyphidia with a hyaline or brownish, occasionally encrusted hyphoid stem, 1.5–8 μm in diam., and hyaline apical branches, 1–1.5 μm in diam., embedded in brownish gelatinous matrix and forming a continuous layer up to 40 μm thick. Probasidia distinctly tapering or clavate, 9–24 × 4–7 μm. Basidia four-celled, ellipsoid-ovoid, more rarely broadly clavate, sometimes obliquely septate, (10.5–) 11–18 (–18.5) × (5.8–) 6.2–11.0 (–11.1) μm (n = 80/8), sterigmata up to 40 × 1–1.5 μm. Basidiospores narrowly cylindrical to allantoid, distinctly curved, occasionally two-or three-celled, (9.2–) 9.3–15.7 (–15.8) × (2.8–) 2.9–4.1 (–4.3) μm (n = 260/9), L = 11.32–13.86, W = 3.18–3.72, Q = 3.35–4.13.

##### Remarks.

*Exidia straminea* is a look-alike of *E. recisa* distributed in the southern part of Europe. It inhabits dead branches and thin stems of various shrubs but seemingly never occurs on *Salix* spp. Morphologically, it can be separated from *E. recisa* in having amber-yellow or light-brown basidiomata without any tints of red, as well as by its shorter basidiospores. Another similar species occurring in the same geographic area on deciduous hosts is *E. impressa*. It has much darker basidiomata than *E. straminea* and occasionally possesses distinctive sharp projections on the hymenial surface. Microscopically, it can be separated from *E. straminea* in having two types of hyphidia. The basidiospores of both species are nearly identical.

*Exidia straminea* was described from southern France (Berkeley 1851). We could not trace the type specimen of this species. The only extant authentic material of *E. straminea* is a microscopic drawing published by Berkeley. We therefore select it as a lectotype (iconotype) of *E. straminea*. Unfortunately, it provides no worthwhile information for species recognition. The “2–3-septate moniliform threads” depicted by Berkeley may equally be bubble-like segments of subhymenial hyphae or immature basidia. Fortunately, the macroscopic part of the protologue is rather clear and it evidently refers to a pale-colored specimen of *E. straminea* as it is reintroduced above. We designate an epitype to maintain our understanding of this species. The description of *E. impressa* by Bourdot and Galzin (1927) mostly refers to *E. straminea*. This species was misidentified by Wu et al. (2020) as *E. repanda*.

#### Exidia subglandulosa

Taxon classificationAnimaliaAuricularialesAuriculariaceae

F. Wu, L.F. Fan & S.Y. Ye, Mycological Progress 19: 862, 2020

BAA9DFFF-538D-521A-8725-8E974D8E7D2F

##### Holotype.

China • Qinghai: Zeku, Maixiu, *Salix cheilophila*, 12 Jul 2019, Wu 270* (BJFC).

*Exidia subglandulosa* is closely related to *E. spiculata* (Figs 10, 11). The species are very similar morphologically, and the only reliable difference between them seems to be the basidiospore width. The spores are on average wider in *E. subglandulosa* (see description in Ye et al. 2020) than in *E. spiculata*. *Exidia subglandulosa* is so far known only from China.

#### Exidia tanzanica

Taxon classificationAnimaliaAuricularialesAuriculariaceae

Spirin & Ryvarden sp. nov.

5FDAC009-473C-5930-AECA-0DFC70462126

862479

Fig. 23G

##### Holotype.

Tanzania • Arusha Prov.: Arusha Nat. Park, Mt. Meru (crater side), dead wood, 24 Jun 1994, Ryvarden 34395* (O, isotype – H).

##### Etymology.

Tanzanicus (Lat., adj.) – Tanzanian.

##### Description.

Basidiomata annual, effused, up to 7 cm in the widest dimension, gelatinous, semitranslucent, 0.5–1.5 mm thick. Hymenial surface dark brownish-gray to brownish-black in fresh condition, corneous and nearly black in dry specimens, covered by densely arranged, grayish, blunt projections, 100 × 80 μm, 4–10 per mm. Margin sharply delimited, detaching, concolorous with hymenial surface, always more or less clearly lobate.

Hyphal structure monomitic, hyphae clamped; subicular hyphae hyaline, thin-walled or with variably thickened, gelatinized walls, interwoven, 1.5–4 (5) μm in diam., subhymenial hyphae hyaline or grayish, thin-walled or with a distinct, gelatinized wall, ascending or interwoven, agglutinated, 1.5–3 μm in diam., basidia-bearing hyphae 2–5 μm in diam. Crystals accidentally present, angular, small. Hyphidia richly branched, with a hyphoid or subfusiform stem, 2–6 μm in diam., and grayish or brownish-black apical branches, 1–2 μm in diam., forming a continuous layer up to 30 μm thick. Probasidia tapering to narrowly clavate, 13–29 × 6.5–8.5 μm. Basidia four-celled, narrowly ellipsoid-ovoid, sessile, more or less clearly tapering to the base, (16–) 17.5–23.5 (–24) × (9.6–) 10.2–13.5 (–13.8) μm (n = 20/1), slightly thick-walled, embedded, sometimes obliquely septate, sterigmata up to 10 × 2.5–3 μm. Basidiospores thin-walled, broadly cylindrical to cylindrical, often distinctly curved, rarely almost narrowly ovoid, (9.1–) 9.2–12.2 (–12.6) × (4.2–) 4.4–6.1 (–6.2) μm (n = 20/1), L = 10.97, W = 5.32, Q = 2.08.

##### Remarks.

*Exidia tanzanica* is introduced here as a close relative of *E. japonica* and other species recently assigned to the genus *Tremellochaete* (e.g., *T. atlantica* and *T. australiensis*). Due to its nearly black basidiomata covered by densely arranged, obtuse hymenial projections, *E. tanzanica* is strongly reminiscent of *E. japonica*. However, the latter species differs from *E. tanzanica* in having narrower basidiospores (4–5 μm wide) and smaller basidia (up to 15 μm long). See also remarks on *Exidia purpureocinerea* (under Excluded taxa).

#### Exidia truncata

Taxon classificationAnimaliaAuricularialesAuriculariaceae

Fr., Systema Mycologicum 2: 224, 1822

ED5154B2-7440-5AAD-95BF-C8B17C67324E

Figs 23H, 24C

##### Neotype

**(designated here, MBT 10031443)**. Sweden • Västergötland: Göteborg, Guldheden, *Quercus robur* (dead hanging branch), 28 Dec 2024, Spirin 18029* (GB, duplicate – H).

##### Description.

Basidiomata annual, button-shaped or adpressed-orbicular, narrowly attached, rarely with a reduced stalk-like base up to 0.5 cm long, later turbinate or nearly cupulate, up to 5 cm in diam., up to 1.5 cm thick, gelatinous. Hymenial surface uneven or indistinctly folded, covered by rather regularly arranged spine-like black projections up to 0.2 mm long, dark gray to brownish-gray, later brownish-black, in dry condition completely black. Abhymenial surface rough, covered by densely arranged blackish dots or sharp outgrowths up to 0.1 mm long, grayish-brown. Margin free, concolourous with hymenial surface, entire, even or indistinctly lobate.

Hyphal structure monomitic, hyphae clamped; context hyphae hyaline or rarely grayish, thin- to distinctly thick-walled, predominantly interwoven, anastomosing, 0.5–6 μm in diam., subhymenial hyphae hyaline to grayish or brownish, thin-walled or with a distinct wall, ascending or interwoven, 1.5–4 μm in diam., basidia-bearing hyphae 1.5–5 μm in diam. Hyphidia with a hyaline or grayish-brownish hyphoid stem, 1.5–8 μm in diam., and hyaline or pale grayish apical branches, 0.5–1 μm in diam., forming a continuous layer up to 50 μm thick. Probasidia tapering to subfusiform, 14–22 × 5–10.5 μm. Basidia four-celled, ellipsoid-ovoid, thin-walled, (14.5–) 15–22 (–23) × (9.1–) 9.3–13.3 (–13.7) μm (n = 30/3), sterigmata up to 50 × 1.5–2 μm. Basidiospores narrowly cylindrical to allantoid, distinctly to strongly curved (lunate), (13.0–) 13.7–20.1 (–20.4) × (4.3–) 4.4–6.2 (–6.3) μm (n = 90/3), L = 17.12–18.03, W = 4.89–5.06, Q = 3.44–3.57.

##### Remarks.

*Exidia truncata* was described by Fries (1822). However, its identity remained controversial, mainly because no original specimens or illustrations were extant. Neuhoff (1936b) applied the name *E. truncata* to an *Exidia* with adpressed-orbicular or turbinate basidiomata occurring mainly on *Quercus* branches in temperate Europe. Our data show that Neuhoff’s concept of *E. truncata* covers two similar and closely related species. The first species has a hairy abhymenial surface, while it is minutely dotted or scabrose (covered by tiny, densely arranged sharp warts) in the second one. Moreover, the second species has longer basidiospores than the first one. The ongoing convention, including recent DNA studies, attributed the epithet *E. truncata* to the first species (Weiß and Oberwinkler 2001; Ye et al. 2020; Gruhn et al. 2024). However, this attribution is at odds with Fries’ concept of the species. He described *E. truncata* as ‘subtus punctato-scabra’ (Fries 1822: 224) while all forms with a hairy abhymenial surface were addressed by him to *E. glandulosa* (ibid., ‘subtus cinerea subtomentosa’). We therefore reintroduce the name *E. truncata* for the second of the two above-mentioned species; the first species is redescribed and typified above as *E. glandulosa*.

*Exidia truncata* seems to be rather common in the south-western part of Sweden. The GenBank sequence KU973844 from Tunisia belongs to this species, too. It means that the species is most likely widely distributed and just overlooked. Fries observed *E. truncata* growing in the winter, and this fits our observations. The basidiomata of *E. truncata* start developing in December and completely disappear by the end of April. In contrast, the similar *E. glandulosa* can be collected throughout the year, although it sporulates mainly in winter or early spring. All our records of *E. truncata* came from hanging or recently fallen branches of *Quercus robur*. Fries (1822) stated that he collected *E. truncata* from *Tilia* twigs. It is so far uncertain whether he misidentified the host tree or whether *E. truncata* can inhabit host trees other than *Quercus*. The third, equally possible option is that Fries’ concepts of both *E. glandulosa* and *E. truncata* were intertwining – a suggestion made by Donk (1966) and discussed above under *E. glandulosa*. Whichever the case is, it does not influence the neotype selection guided by the most reliable feature distinguishing the two species, namely the character of the abhymenial surface.

#### Exidia umbrinella

Taxon classificationAnimaliaAuricularialesAuriculariaceae

Bres., Fungi Tridentini 2 (14): 98, 1900

1539721A-B639-5FE9-AD52-9AB78F82FFE3

Figs 23I, 24D

##### Lectotype

**(selected here, MBT 10031444)**. Italy • Trentino-Alto Adge: Trento, Pellizzano, Val di Sole, *Abies alba*, 1888 *Bresadola* (S F20648, studied).

##### Description.

Basidiomata annual, button-like to cupulate-orbicular, up to 2 cm in diam., narrowly attached, up to 0.5 cm thick, gelatinous. Hymenial surface even or folded, rarely bearing scattered granular projections up to 0.1 mm long, at first ochraceous- to reddish-brown, later dark brown, in dry condition darkening to nearly black. Abhymenial surface rough, covered by tightly arranged dots, reddish-brown or dark brown. Margin free, concolourous with hymenial surface, entire, even or indistinctly lobate.

Hyphal structure monomitic, hyphae clamped; context hyphae hyaline or yellowish, thin-walled or with variably thickened, gelatinized walls, interwoven or subparallel, anastomosing, 1–5 μm in diam., subhymenial hyphae hyaline or yellowish-brownish, thin-walled or with a distinct wall, predominantly ascending, 1–3 μm in diam., basidia-bearing hyphae thin-walled or with a distinct wall, 2–4 μm in diam. Hyphidia with a hyaline or brownish, often sinuous hyphoid stem, 3–5 μm in diam., and hyaline or brownish apical branches, 1–2 μm in diam., embedded in yellowish-brownish gelatinous matrix and forming a continuous layer up to 20 μm thick. Probasidia tapering or clavate, 9–13.5 × 4.5–5.5 μm. Basidia four-celled, narrowly ellipsoid-ovoid, (9.5–) 10–13.5 (–14) × (6.2–) 6.7–9.8 (–10.8) μm (n = 42/2), sterigmata up to 30 × 1–2 μm. Basidiospores narrowly cylindrical to allantoid, distinctly curved, (8.7–) 9.0–13.2 (–13.8) × (2.7–) 2.8–3.8 (–4.0) μm (n = 52/2), L = 11.29–12.00, W = 3.10–3.33, Q = 3.41–3.90.

##### Remarks.

*Exidia umbrinella* is a member of the *E. recisa* complex (Fig. 9). It is the only species in this group known so far to occur on coniferous hosts. The substrate, as well as rather short basidiospores, provide sufficient grounds to separate *E. umbrinella* from the otherwise very similar *E. recisa*. This species was described by Bresadola (1900) based on a rather large set of specimens from Italy and Slovakia. *Abies* spp. and *Larix decidua* were mentioned as its substrates. Here we select the best-preserved, fertile specimen of *E. umbrinella* from Stockholm as a lectotype of this species. Bourdot and Galzin (1927) reported *E. umbrinella* from France. Neuhoff (1935) published records of *E. umbrinella* from Central Europe which seem to belong to this species, except for his records on *Pinus* in Germany most likely referring to *Ulocolla saccharina* (see below). Here we report *E. umbrinella* from the Caucasus.

#### Exidia uvapassa

Taxon classificationAnimaliaAuricularialesAuriculariaceae

Lloyd, Mycological Writings 5: 774, 1918

EFC09F50-4C8A-5728-A2D9-16FCEE2E09F2

Fig. 24E

##### Lectotype

**(selected by Stevenson and Cash 1936: 32)**. Japan • Kobe, [no collecting date], J.E.A. Lewis (Lloyd’s herbarium #33506) (BPI).

For a description, see Malysheva (2012).

#### Exidia yadongensis

Taxon classificationAnimaliaAuricularialesAuriculariaceae

F. Wu, Qi Zhao, Zhu L. Yang & Y.C. Dai, Mycosystema 39: 1210, 2020

8B83B0A1-E1C1-58C1-92B1-710D0CE88856

##### Holotype.

China • Hubei: Shennongjia, *Ulmus* sp., 16 Oct 2016, Dai 17268* (BJFC).

For a description, see Wu et al. (2020). This species was introduced as a member of the *E. recisa* complex closely related to the North American *E. crenata*. Here we report it from the Russian Far East. See further remarks under *E. crenata* above. *Exidia impressa*, occurring in the same geographic area and on the same set of hosts, can be separated from *E. yadongensis* by its darker basidiomata with occasional sharp hymenial projections and the presence of two types of hyphidia.

#### Exidia zelleri

Taxon classificationAnimaliaAuricularialesAuriculariaceae

Lloyd, Mycological Writings 6: 931, 1920

E67E65E8-20B4-58B3-A927-40859FFCA319

Figs 23J, 24F

= Exidia glandulosa f. lobata Neuhoff, Arkiv för Botanik 28A (1): 13.
= Exidia glandulosa f. nannfeldtii Neuhoff, Arkiv för Botanik 28A (1): 14.

##### Holotype.

USA. Oregon: Benton Co., Corvallis, [*Acer* sp.], 11 Oct 1919, Zeller 1775 (NY00738335 – isotype, studied) (Figs 23J, 24F).

##### Description.

Basidiomata annual, at first adpressed-orbicular, narrowly attached, then turbinate or nearly cupulate, up to 1 cm in diam., up to 0.5 cm thick, sometimes partly fusing together and forming compound effused basidiomata, up to 3 cm in the longest dimension, gelatinous. Hymenial surface uneven or indistinctly folded, rarely minutely warted, brownish-black, in dry condition completely black. Abhymenial surface smooth, grayish-brown to brownish-black. Margin free, concolourous with hymenial surface, entire, even or lobate.

Hyphal structure monomitic, hyphae clamped; context hyphae hyaline or brownish, predominantly thin-walled, interwoven, anastomosing, 1–5 μm in diam., subhymenial hyphae hyaline to brownish, thin-walled or with a distinct wall, ascending or interwoven, 1–4 μm in diam., basidia-bearing hyphae 2–6 μm in diam. Hyphidia with a hyaline or brownish hyphoid stem, 2–11 μm in diam., and hyaline or brownish apical branches, 0.5–2 μm in diam., forming a continuous layer up to 100 μm thick. Probasidia broadly clavate to tapering or subfusiform, 15–29 × 6–11.5 μm. Basidia four-celled, ellipsoid-ovoid to narrowly ovoid, thin-walled, (14–) 14.5–26 (–28.5) × (8.9–) 9.0–15.1 (–15.3) μm (n = 71/5), sterigmata up to 50 × 1.5–2.5 μm. Basidiospores narrowly cylindrical or cylindrical to allantoid, slightly to distinctly curved, (13.0–) 13.2–21.9 (–22.2) × (4.1–) 4.2–7.0 (–7.2) μm (n = 140/5), L = 16.49–18.68, W = 4.81–6.08, Q = 2.92–3.55.

##### Remarks.

*Exidia zelleri* is a member of the *E. glandulosa* complex (Figs 1, 2, 8). Macroscopically, it is very different from the two other species of this group, *E. glandulosa* and *E. truncata*, due to its rather small basidiomata with smooth hymenial and abhymenial surfaces (Fig. 24). However, its large basidia and basidiospores are strongly reminiscent of those of the two aforementioned species. *Exidia zelleri* is widely distributed in the northern hemisphere but rarely collected, most likely due to its quite insignificant fructifications. It occurs predominantly on dead wood of *Salicaceae* and rarely also on other host trees (*Acer* and *Alnus*). Specimens of *E. zelleri* are often sterile. They can be nonetheless identified as belonging to this species due to the nearly smooth hymenial surface without spine-like projections, as well as the large basidia.

*Exidia zelleri* was described by Lloyd (1920) from Oregon, USA. The host species was identified by the original collector as *Sambucus*. However, the later re-examination by H.C. Klett (herbarium note in NY from August 26^th^, 1963) showed that the host tree was actually *Acer* sp. The isotype of *E. zelleri* in NY is still in a good condition, with rather numerous, turgid, allantoid or narrowly cylindrical spores, ca. 14–18 × 4–5.5 μm. These spore dimensions, in combination with the lack of hymenial projections, definitely preclude any other nigrescent *Exidia* species dealt with in this paper. Neuhoff (1936b) introduced two forms of *E. glandulosa* sensu auct. from northern Sweden, f. *lobata* and f. *nannfeldtii*. Based on his descriptions, they are both considered here as belonging to *E. zelleri*. Torkelsen (1972) reported f. *lobata* from Norway. The basidial and basidiospore measurements given by her certainly point to *E. zelleri*.

#### Exidiopsis

Taxon classificationAnimaliaAuricularialesAuriculariaceae

(Bref.) Vuillemin, Bulletin des Séances de la Société des Sciences de Nancy 2 (3): 30, 1890

C03165B8-9CC3-5F50-B162-8683E17C1C90

≡ Exidia subgen. Exidiopsis Bref., Untersuchungen aus dem Gesammtgebiete der Mykologie 7: 94, 1888.

##### Description.

Basidiomata annual, effused, waxy-gelatinous, watery-grayish, often with pink or lilac hues. Hymenial surface smooth or spinulose. Hyphal structure monomitic; hyphae clamped, hyaline, gelatinized and often agglutinated, basidia-bearing hyphae usually well-differentiated. Crystals rare or usually absent. Cystidia absent. Hyphidia present, not encrusted, at least partly covering basidial cells. Basidia four-celled, ellipsoid-ovoid, sessile, thin-walled, usually embedded, often with a distinct stalk-like base. Basidiospores thin-walled, cylindrical or allantoid, distinctly curved. On dead wood of angiosperms.

##### Generic type.

*Exidia effusa* Bref.

Our data show that the generic type of *Exidiopsis*, *E. effusa*, is not closely related to any other previously described *Exidiopsis* species. Instead, it is sister to *Heteroradulum* (Figs 1, 2; see Results) and, probably, *Heterochaete* s. typi (Malysheva and Spirin 2017); however, their phylogenetic relationships must be investigated further, with a much larger taxon sampling than used in the present study. In the current scope, the genus contains two species, *E. effusa* and *E. perflua*, introduced below. These two species differ from other species previously assigned to *Exidiopsis* in having pale and thin, waxy-gelatinous basidiomata, usually with a pink or lilac hue, ellipsoid-ovoid basidia with a clearly tapering base, long and curved basidiospores, as well as in lacking cystidia and distinctive encrustation.

The taxonomic history of the genus is complicated. *Exidiopsis* was first introduced by Brefeld (1888) as a subgenus of *Exidia* for a single species, *Exidia effusa*. However, the species name was given in his text as ‘*Exidiopsis effusa*’, and this naming became the source of subsequent controversy. Donk (1958) correctly cited Brefeld’s binomial as *Exidia effusa* and considered it as validly published. However, he changed his mind eight years later (Donk 1966) and cited Brefeld’s species as *Exidiopsis effusa* Bref. ‘n. v. p.’ (= not validly published). The reasons for this later interpretation were not provided. Most likely, Donk switched to a more literal interpretation of Brefeld’s description and considered *Exidiopsis effusa* as a new species described by Brefeld without introducing the appropriate genus for it, i.e. as invalid. This viewpoint was accepted by Index Fungorum and MycoBank (as of December 1^st^, 2025), both listing Brefeld’s binomial as the invalidly published *Exidiopsis effusa*. However, Brefeld’s explanations in the protologue of *Exidiopsis effusa* clearly show that he found no grounds to raise *Exidiopsis* to the genus rank and even argued against this option (Brefeld 1888: 94–95). It is therefore evident that Brefeld ascribed his new species to *Exidia* and merely followed the fashion of the time in using subgenera along with generic names in the species descriptions. Hence, *Exidia effusa*.

Vuillemin (1890: 30) was the first author who used the name *Exidiopsis* at the genus level. He introduced a new species under the name *Exidiopsis quercina* and compared it with ‘*Exidiopsis effusa* Bref.’ By using the latter binomial, Vuillemin in fact created a new combination, *Exidiopsis effusa* (Bref.) Vuillemin, as was already noted by Donk (1958). Donk (ibid.) further stated that the new species *E. quercina* and the new combination *E. effusa* were not validly published by Vuillemin because he ‘did not mention separately’ the generic name, i.e. *Exidiopsis*, at its new rank. However, this is not necessary under the current version of the Code: the indication ‘*Exidiopsis effusa* Bref.’ in Vuillemin’s description constitutes an indirect reference not only to *Exidia effusa* Bref. (Art. 38.14) but also to *Exidiopsis* as a validly published subgenus of *Exidia* (Art. 41.3). In other words, Vuillemin simultaneously combined *Exidia effusa* in *Exidiopsis* and raised the latter name to genus rank, thus fulfilling the requirements of Art. 41.2. Therefore, the generic name must be cited as *Exidiopsis* (Bref.) Vuillemin, while *Exidiopsis* (Bref.) Möller is merely its later isonym.

#### Exidiopsis effusa

Taxon classificationAnimaliaAuricularialesAuriculariaceae

(Bref.) Vuillemin, Bulletin des Séances de la Société des Sciences de Nancy 2 (3): 30, 1890

26560DEF-ADDF-5E4D-A51E-9F4652B44F41

**Figure 25.**
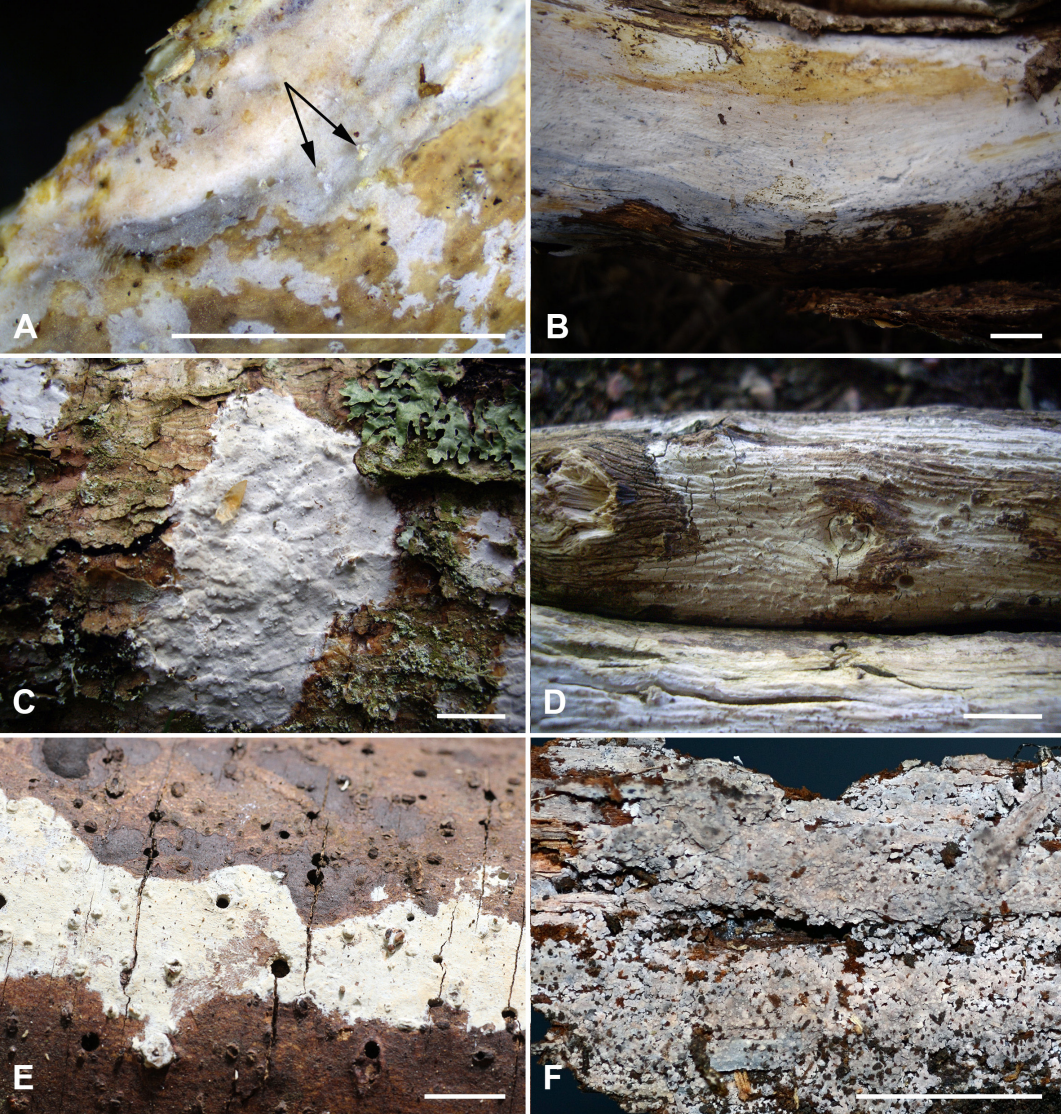
Basidiomata: **A***Exidiopsis effusa* (epitype; black arrows point to the spine-like hymenial outgrowths); **B***E. perflua* (Spirin 12638); **C***Leiostroma farinellum* (Spirin 12597); **D***L. incanum* (holotype); **E***L. infecundum* (holotype); **F***Pholiobasidion senex* (holotype). Scale bar: 1 cm.

**Figure 26.**
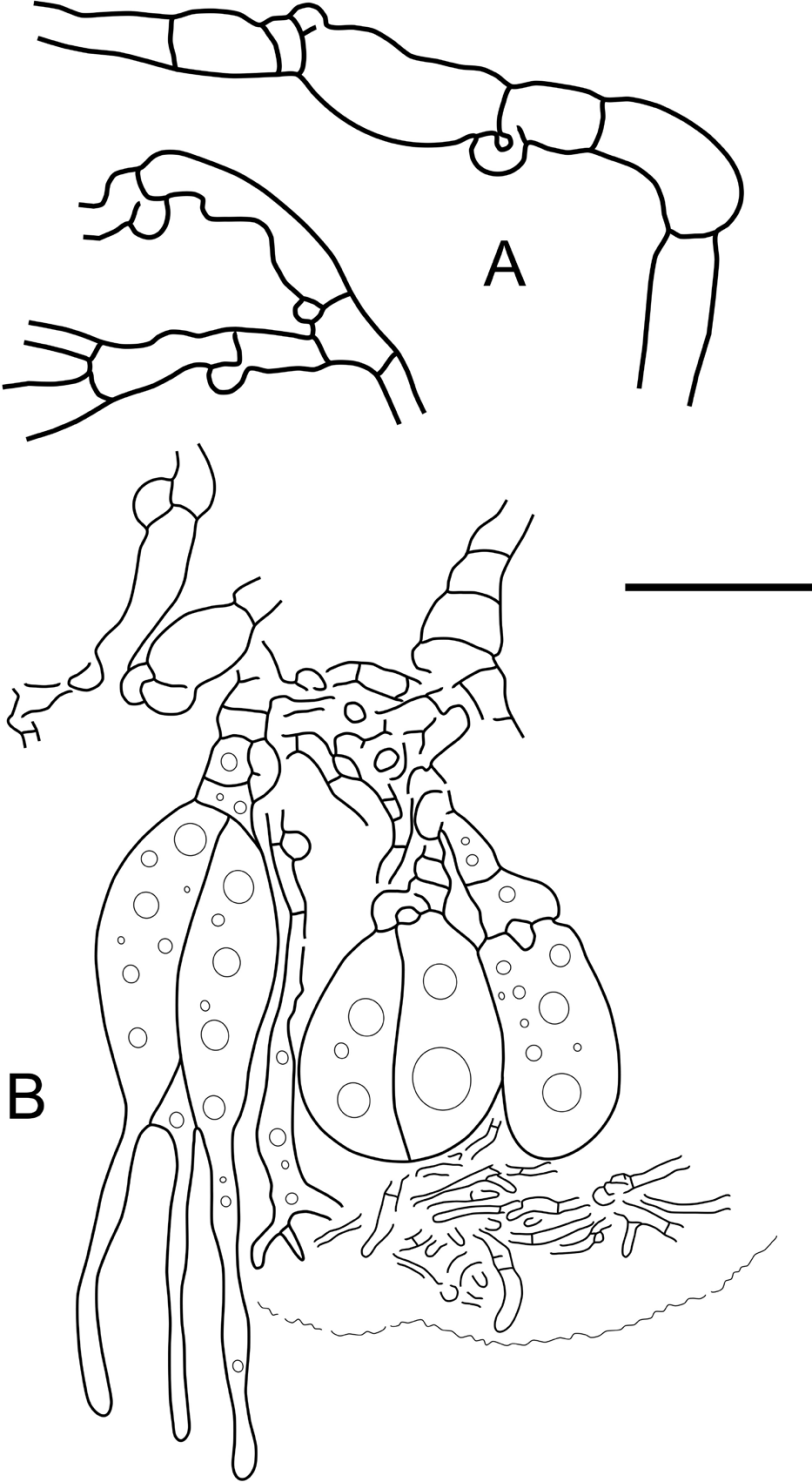
Microstructures of *Exidiopsis effusa* (epitype): **A** subicular hyphae; **B** subhymenial hyphae and hymenial cells. Scale bar: 10 μm.

**Figure 27.**
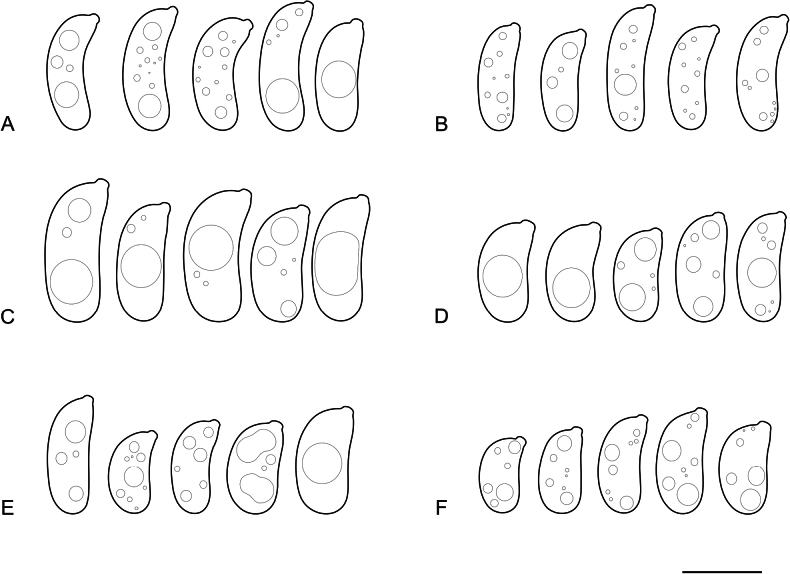
Basidiospores: **A***Exidiopsis effusa* (epitype); **B***E. perflua* (holotype, three spores left) and *E. effusa* (the narrow-spored specimen, Spirin 15511, two spores right); **C***Leiostroma farinellum* (Spirin 12586); **D***L. incanum* (holotype); **E***L. infecundum* (three left – holotype, two right – Viner 2022/1066); **F***L. squalidum* (holotype). Scale bar: 10 μm.

Figs 25A, 26, 27A, 27B

≡ Exidia effusa Bref., Untersuchungen aus dem Gesammtgebiete der Mykologie 7: 94, 1888. Lectotype (selected here, MBT 10031445). Fig. 20, Tafel V in Brefeld, Untersuchungen aus dem Gesammtgebiete der Mykologie 7, 1888. Epitype (selected here, MBT 10031446). Sweden. Västergötland: Göteborg, Guldheden, Salix caprea (fallen log), 14 Mar 2025, Spirin 18205* (GB).
= Exidiopsis quercina Vuillemin, Bulletin des Séances de la Société des Sciences de Nancy 2 (3): 30, 1890. Neotype (selected here, MBT 10031447). France. Lozère: Saint-Etienne-du-Valdonnez, Forêt du Sapet, Fagus sylvatica (fallen branch), 28 Jun 2022, Spirin 15511* (H).

##### Description.

Basidiomata annual, effused, usually up to 10 cm in the longest dimension but occasionally exceeding 1 m long (while growing on dead standing logs), at first waxy-gelatinous and watery-grayish, semitranslucent, then more or less distinctly gelatinized, drying to a hardly visible, vernicose crust, up to 0.1 mm thick. Hymenial surface smooth or more often bearing randomly or rather regularly arranged spine-like projections up to 150 × 100 μm, 2–3 per mm, and at least partly covered by a grayish or pale lilac, easily removable pruina. Margin rather sharply delimited, adnate, concolorous with hymenial surface.

Hyphal structure monomitic, hyphae clamped; subicular hyphae thin- or slightly thick-walled, gelatinized, mostly interwoven, 2–4 μm in diam., occasionally inflated up to 7–8 μm in diam., subhymenial hyphae thin-walled, predominantly ascending, often agglutinated, 1.5–4.5 μm in diam., occasionally inflated up to 5–6 μm in diam.; basidia-bearing hyphae thin-walled, 1.5–4 μm in diam., sometimes showing a zigzag-like branching pattern. Hyphidia thin-walled, with a hyaline, hyphoid or subfusiform stem, 20–40 × 2–6 μm in diam., and dendroid apical branches 1–1.5 μm in diam., forming a loose continuous layer up to 15 μm thick. Probasidia broadly clavate, 10–30 (–40) × 5–9 μm. Basidia narrowly ellipsoid-ovoid, four-celled, (10.5–) 12–21.5 (–23) × (8.0–) 8.1–12.3 (–13.2) μm (n = 80/7), thin-walled, embedded, often with a distinctly tapering stalk-like base, up to 7 × 4 μm, sterigmata up to 25 × 2–3 μm. Basidiospores thin-walled, narrowly cylindrical to cylindrical, often distinctly curved, (9.4–) 9.8–16.8 (–17.2) × (3.6–) 3.7–6.2 (–6.6) μm (n = 210/7), L = 11.40–14.26, W = 4.14–5.26, Q = 2.46–2.91.

##### Remarks.

As mentioned above, this species was first described from Germany by Brefeld (1888) as *Exidia effusa* and later moved to *Exidiopsis* by Vuillemin (1890). No authentic specimens by Brefeld are extant. We therefore select his illustration accompanying the protologue as a lectotype of this species. Brefeld’s description and drawings are very clear, and the identity of his species is certain. One of the most important macroscopic traits mentioned in the protologue is the presence of papillate outgrowths on the hymenial surface. We were able to observe them in all freshly collected specimens of *E. effusa* (Fig. 25A). These projections, as well as (at least partly) gelatinized, semitranslucent basidiomata of *E. effusa* are the easiest traits to separate it from the closely related *E. perflua* (see further remarks under that species).

According to our data, *E. effusa* has a western distribution in Europe. We studied specimens of *E. effusa* from France, Luxembourg and the southern part of Scandinavia. GenBank sequence PV330055 also indicates its presence in Belgium. In turn, *E. perflua* seems to be restricted to more northern and eastern areas of Europe. Based on this distribution pattern, we treat *E. quercina*, originally collected in the north-eastern part of France, as a synonym of *E. effusa*. Maire (1902) stated that he had an opportunity to study authentic material of *E. quercina* stored in alcohol. The current location of this material is unknown. We therefore select a neotype for *E. quercina*.

#### Exidiopsis perflua

Taxon classificationAnimaliaAuricularialesAuriculariaceae

Spirin sp. nov.

0CC5F046-C2BA-5CD2-8DC7-4789F1007F4D

862480

Figs 25B, 27B

##### Holotype.

Russia • Nizhny Novgorod Reg.: Lukoyanov Dist., Razino, 54.92449; 44.28289, *Ulmus glabra* (fallen decorticated branch), 28 Jul 2021, Spirin 14386* (H).

##### Etymology.

Perfluus (Lat., adj.) – spilt, diffluent.

##### Description.

Basidiomata annual, effused, up to 15 cm in the longest dimension, waxy, up to 0.1 mm thick. Hymenial surface smooth, at first watery-grayish, semitranslucent, then often with pinkish or lilac hues, opaque, grayish-white in herbarium. Margin first pruinose, in older basidiomata rather sharply delimited or gradually thinning, adnate, concolourous with hymenial surface.

Hyphal structure monomitic, hyphae clamped; subicular hyphae thin- or slightly thick-walled, interwoven or subparallel, anastomosing, 1.5–5 μm in diam., accidentally inflated up to 6–7 μm in diam., subhymenial hyphae thin-walled, interwoven or ascending, in older basidiomata partly agglutinated, 1.5–3 μm in diam.; basidia-bearing hyphae thin-walled, 2–4 (–5) μm in diam. Hyphidia thin-walled, with a hyaline, hyphoid or nodulose stem, 2–6 μm in diam., and dendroid apical branches 0.5–1.5 μm in diam., scattered among basidia or partly covering hymenial cells and forming a continuous layer up to 20 μm thick. Probasidia broadly clavate, 13–29 × 5.5–14 μm. Basidia narrowly ellipsoid-ovoid to nearly obconical, four-celled, (10–) 10.5–20 (–21) × (6.6–) 6.9–13.6 (–15.8) μm (n = 101/9), thin-walled, exposed or embedded, occasionally with a distinctly tapering stalk-like base, up to 5 × 4 μm, sterigmata up to 45 × 2–3 μm. Basidiospores thin-walled, allantoid to cylindrical, more rarely broadly cylindrical, often distinctly curved, (9.8–) 10.1–16.1 (–17.6) × (3.8–) 3.9–6.2 (–6.4) μm (n = 300/10), L = 11.90–14.00, W = 4.11–5.83, Q = 2.05–3.24.

##### Remarks.

*Exidiopsis perflua* is introduced here as a sister species of *E. effusa*. Macroscopically, it can be differentiated from the latter species due to its smooth basidiomata which become opaque when mature or after drying. In contrast, basidiomata of *E. effusa* remain semitranslucent and they often bear small spine-like projections on the hymenial surface. Microscopically, *E. perflua* differs from *E. effusa* in having slightly narrower subhymenial hyphae and smaller basidia which are often openly arranged. These differences are subtle, however, and in many cases the basidiospore size does not separate the two species either. According to our data, the distribution areas of the two species do not coincide, at least at the large extent: *Exidiopsis perflua* appears to prefer colder areas with a more continental climate and *E. effusa* seems to be restricted to the western, more temperate parts of Europe

*Exidiopsis perflua* and *E. effusa* produce basidiomata from midwinter through the early summer. During colder seasons, they may persist longer, until July or August. Both species inhabit still hard, dead, hanging or just fallen branches or, more rarely, thin dead standing logs of angiosperms. These branches are usually partly covered by bark, and the basidiomata of *Exidiopsis* spp. occur on decorticated parts.

#### Leiostroma

Taxon classificationAnimaliaAuricularialesAuriculariaceae

Fr., Novitiae Florae Suecicae 5: 80, 1819

0170A89F-61B4-5B0F-9C7D-E9D16E17A72C

##### Description.

Basidiomata annual, effused, arid, soft and easily crumbling, nearly white to gray or brown. Hymenial surface smooth or spinulose. Hyphal structure monomitic; hyphae clamped, hyaline or grayish-brownish, normally spaced, basidia-bearing hyphae well-differentiated. Crystals abundant, angular or sand-like. Cystidia absent. Hyphidia present, with a well-differentiated, often widened stem and richly branched, densely encrusted apical outgrowths. Basidia four-celled, ellipsoid-ovoid to obconical, sessile, thin-walled or with a distinct wall, exposed or embedded, occasionally with a reduced stalk-like base; some basidia with a grayish-brownish cytoplasm. Basidiospores thin-walled, narrowly to broadly cylindrical, curved. On dead wood of angiosperms and conifers.

##### Generic type.

Thelephora acerina var. abietis Fr. (= *Corticium abietis* (Fr.) Romell) (see comments below).

**Figure 28.**
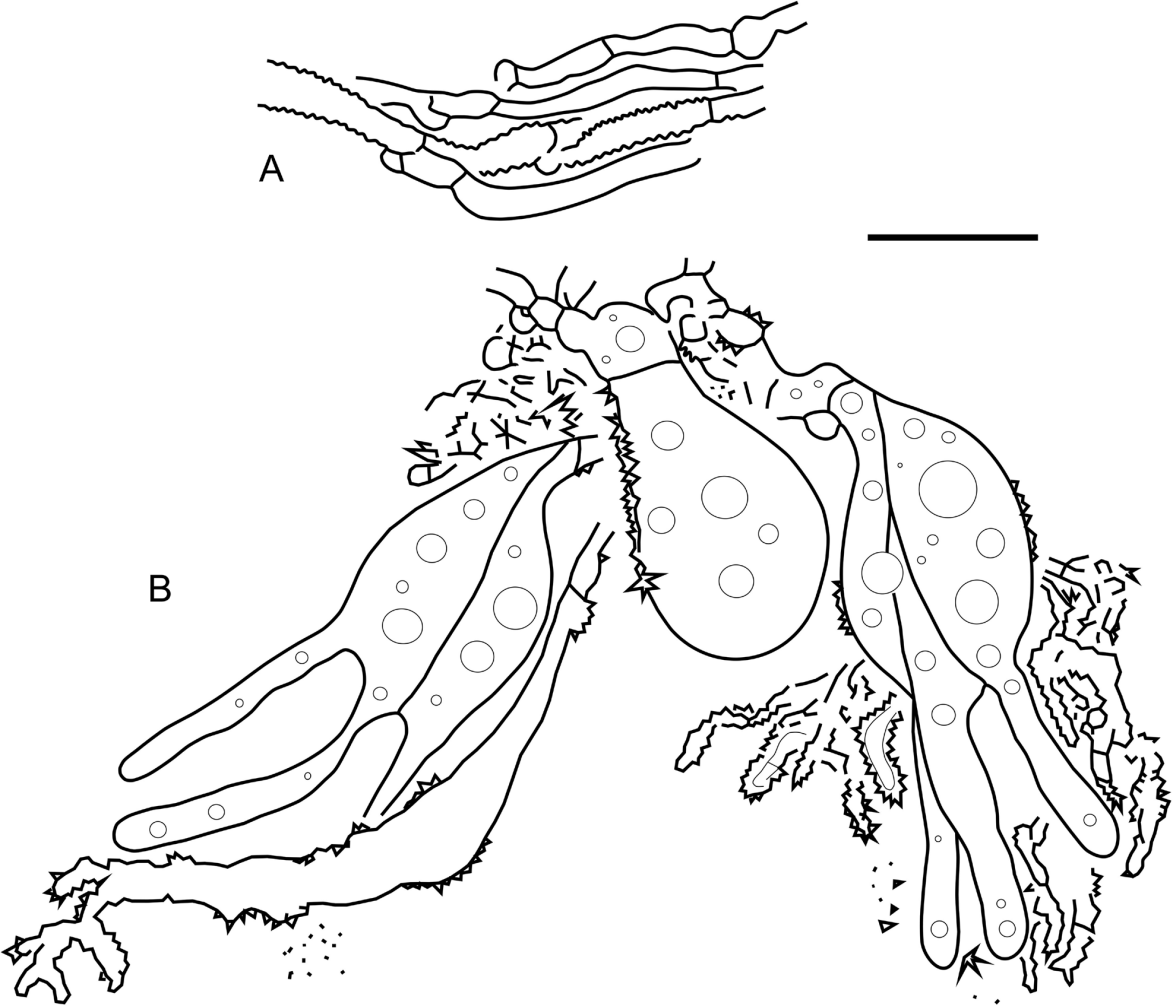
Microstructures of *Leiostroma farinellum* (J. Olsson 13.4.2025): **A** subicular hyphae; **B** subhymenial hyphae and hymenial cells. Scale bar: 10 μm.

As reintroduced here, *Leiostroma* encompasses four species having soft, easily crumbling, effused basidiomata with more or less pronounced tints of gray or brown. The same colouration is also characteristic for the contents of (at least some) hyphae and hymenial cells. All included species lack cystidia and possess very characteristic, abundant, hyaline, sand-like crystalline encrustation. These encrusting bodies are often very densely arranged, which makes microscopic structures difficult to observe. They form continuous shields on subicular hyphae and, in mature basidiomata, also on subhymenial hyphae and occasionally on hymenial cells (Fig. 28). These features, as well as the lack of cystidia, separate *Leiostroma* spp. from the similar-looking smooth *Adustochaete* species. Moreover, basidia-bearing hyphae are well differentiated in *Leiostroma* and usually clearly wider than subhymenial hyphae; in *Adustochaete* spp., no perceptible difference between these hyphal types was observed. Some *Proterochaete* spp. (*P. furfurosa*, *P. letendreana*, and *P. mucedinea*) macroscopically similar to *Leiostroma* can be differentiated by their more densely arranged, partly glued hyphae (vs. spaced in the latter genus), as well as the presence of cystidia (except for *P. letendreana*) and granular, brownish, cyanophilous deposits on hyphae and hymenial cells. Differences between the two genera are discussed in more detail under *Proterochaete*. Mature, faded basidiomata of *Tegmenticium peritrichum* can be mistaken for *Leiostroma* spp.; however, it has cystidia and is devoid of incrustation on hyphae and hymenial cells.

Here we reinstate the generic name *Leiostroma* to encompass, to some extent, a species, formerly known as *Exidiopsis calcea* in North Europe, as well as three closely related species from the southern part of Europe, Macaronesia and Africa. Liu et al. (2022) uncritically took *E. calcea* and, without clarifying its identity, combined it in their newly described genus, *Alloexidiopsis*. The genus was typified with the simultaneously introduced *A. schistacea*, an effused, *Heterochaete*-like species from the southern part of China. The latter species is probably identical with *Heterochaete cretacea* Pat. from Vietnam (Patouillard 1924). Although it was shown by Liu et al. (2022) as forming a strongly supported clade with the so-called *E. calcea* and several other species, the question about their relationships, in our opinion, cannot be solved solely on the basis of the ribosomal gene-based phylogenies. Available ITS sequences of *A. schistacea* reveal a relatively low similarity (less than 94%) with *E. calcea* s. auct. and three other species dealt with below. LSU sequences of this species provide no additional information because they show *A. schistacea* to be very close to and, at the same time, equally distant from *E. calcea* s. auct. with its closest relatives on one hand and *Heteroradulum* spp. on another (98.5–98.7% similarity). Due to the lack of the specimens suitable for obtaining additional genetic markers, we leave the question about the phylogenetic placement of *A. schistacea* open. In the following paragraphs, we argue for resurrecting the genus name *Leiostroma* for *E. calcea* s. auct. and its closest relatives; our arguments in favor of discarding the species name *Thelephora calcea* Pers. (basionym of *E. calcea*) are provided under *Leiostroma farinellum*.

The genus *Leiostroma* was introduced by Fries (1819) without any species assigned to it (“Species plures”). Two years later, Fries (1821) lowered the rank of *Leiostroma* to the section in the genus *Thelephora* and described six species therein, i.e. *Thelephora acerina*, *T. cinerea*, *T. fallax*, *T. maculaeformis*, *T. amphibolia*, and *T. sterilis*. It seems this list reflects his original views on the scope of *Leiostroma* because the description of the section in Systema expands the earlier generic description of *Leiostroma* and does not contradict it. Donk (1957) treated *Leiostroma* sensu Fries 1821 as a subgenus of *Thelephora* and typified it with *T. maculaeformis*. He argued that *T. maculaeformis* was a ‘constant member’ of *Leiostroma*, while two other candidates for typification, *T. cinerea* and *T. acerina*, had been subsequently removed by Fries himself and placed in other sections or genera. Later Donk (1963) detected that *Leiostroma* was published as a genus already in 1819; he, however, maintained his earlier typification of it with *T. maculaeformis*, providing no new arguments. The type material of *T. maculaeformis* was studied by Eriksson (1950) who found it to be conspecific with *Peniophora polygonia* (Pers.) Bourdot & Galzin. The latter species is a member of *Peniophora* in the current sense (Hallenberg et al. 1996).

Before 1982, Donk’s typification looked harmless because *Leiostroma* was considered a devalidated name, i.e. that published prior to Fries’ Systema Mycologicum and therefore having no priority over generic names introduced afterwards. However, after changing the starting point for validly published fungal genera in 1982, *Leiostroma* becomes an older name for *Peniophora*, a large and widely accepted genus of crust fungi. This name change looks very unfortunate, and we therefore argue in favor of abandoning Donk’s typification and conserving *Leiostroma* with another generic type.

Under Code Art. 10.2, any of the six mentioned species mentioned by Fries (1821) is suitable for lectotypification of the genus. However, the first five species are currently classified among well-established genera of corticioid fungi, i.e. *Dendrothele* Höhn. & Litsch. (*T. acerina*), *Peniophora* Cooke (*T. cinerea*, *T. fallax*, and the already mentioned *T. maculaeformis*), and *Phanerochaete* P. Karst. (*T. amphibolia*). Selecting any of these species as a lectotype of *Leiostroma* would result in discarding one of these currently accepted corticioid genera (*Dendrothele*, *Peniophora*, or *Phanerochaete*) from use because it is older than any of them. As far as we know, no authentic specimens of the sixth species, *Thelephora sterilis*, exist.

In the subsequent reintroduction of the section *Leiostroma*, Fries (1828: 224) retained four species from the previous treatise (*T. acerina*, *T. maculaeformis*, *T. amphibolia*, and *T. sterilis*) and added seven more species, i.e. *T. corrugata*, *T. laevigata*, *T. episphaeria*, *T. bufonia*, *T. circinata*, *T. typhae*, and *T. epiphylla*. Again, choosing any of these species as the generic type of *Leiostroma* is problematic: *T. corrugata* and *T. episphaeria* are currently treated under *Hydnoporia*, *T. laevigata* is a species of *Amylostereum*, *T. circinata* is the generic type of *Sarcopodium (Ascomycota)*, *T. typhae* is the generic type of *Epithele*, and *T. epiphylla* is that of *Athelia*. All these currently accepted generic names (except *Athelia* and *Sarcopodium*) are younger than *Leiostroma*. As for *T. bufonia*, its identity remains uncertain. There are five specimens in Persoon’s herbarium in Leiden identified as *T. bufonia*, representing various bark-inhabiting fungi. It seems that Fries was uncertain about identity of this species, and it was certainly not the source of his concept of *Leiostroma*. This leads us to a conclusion that there are no species associated with *Leiostroma* which could be suitable for the typification of this genus without causing problems for the current classification of fungi. Therefore, we have to return to the original description of the genus by Fries in 1819.

The Code Art. 10.2 provides us with a choice: either to select a lectotype of *Leiostroma* among the species associated with its later reintroduction as a section in the genus *Thelephora*, or to choose the type otherwise. The first option looks inconvenient because – as we demonstrated above – it would lead to a disposal of the currently accepted and well-defined genera. So, we follow the second option aligning it, as much as possible, with Fries’ concept of *Leiostroma* as he developed between 1819 and 1828. In both Systema and Elenchus, Fries listed *Thelephora acerina* as the first species in the section *Leiostroma*. He most likely viewed it as the most “typical” representative of the section because the most important features of its introduction (basidiome shape and consistency, hymenium configuration) in Systema repeat those in the section’s description. The species was introduced with four varieties, of which the first, T. acerina var. abietis, is of special interest for the present study. It certainly refers to a taxon macroscopically similar to *Dendrothele acerina* but occurring on *Picea* and clearly implies a widely distributed conifer-dwelling species which was treated as *Sebacina* or *Exidiopsis calcea* after Bresadola (1898) and Wells (1961). In our opinion, the latter name is inapplicable to T. acerina var. abietis (see remarks under *Leiostroma farinellum*) and must be interpreted otherwise. Romell (1895) was the first who clarified the identity of var. abietis, and we see no reasons to reject his interpretation. He raised it to the species level; however, his *Corticium abietis* is preceded by *Xerocarpus farinellus* by Karsten (1882) which we reinstate as a species of *Leiostroma* below. Nevertheless, this fact does not make T. acerina var. abietis unsuitable for the typification of the genus. We therefore propose to conserve it as a type of *Leiostroma*. A conservation proposal has been submitted to the Nomenclatural Committee for Fungi.

#### Leiostroma farinellum

Taxon classificationAnimaliaAuricularialesAuriculariaceae

(P. Karst.) Spirin comb. nov.

2AE2B030-B61C-5255-949F-43361D513DED

862481

Figs 25C, 27C, 28

≡ Xerocarpus farinellus P. Karst., Bidrag till Kännedom av Finlands Natur och Folk 37: 139, 1882. Holotype. Finland. Etelä-Häme: Tammela, Mustiala, Picea abies, 4 Oct 1880, Karsten 1876 (H6054654, studied).
= Thelephora acerina var. abietis Fr., Systema Mycologicum 1: 453, 1821. Neotype (designated here, MBT 10031452). Sweden. Uppland: Stockholm, Picea abies, [no collecting date], Romell (#129 in Fungi Exsiccati Praesertim Scandinavici) (S F350638, studied).
= Corticium abietis (Fr.) Romell, Botaniska Notiser: 72, 1895.
= Thelephora calcea Pers., Synopsis Methodica Fungorum 2: 581, 1801. Lectotype (selected here, MBT 10031453). [No locality or collecting date indicated], herb. Persoon (L 0054598, studied).
= *Sebacina calcea* Bres., FungiTridentini 2: 64, 1892, pr. p.
= Exidiopsis calcea (Pers.) K. Wells, Mycologia 53: 348, 1961, pr. p.

##### Description.

Basidiomata annual, effused, up to 10 cm in the widest dimension, arid, soft, 0.1–0.5 mm thick. Hymenial surface smooth or indistinctly warted, at first nearly white, with grayish or brownish flecks, then ash- to mouse-gray, in older basidiomata brownish-gray, occasionally irregularly cracking. Margin at first arachnoid, then rather sharply delimited, adnate, as a rule remaining white, up to 0.5 mm wide.

Hyphal structure monomitic, hyphae clamped; subicular hyphae hyaline, slightly to distinctly thick-walled, interwoven or subparallel, in older basidiomata partly agglutinated, 2–5 μm in diam., subhymenial hyphae hyaline or grayish-brownish, thin- to slightly thick-walled, ascending or interwoven, rather loosely arranged, occasionally twisted, 1.5–4 μm in diam., basidia-bearing hyphae thin- to slightly thick-walled, 3–5.5 μm in diam. Crystals abundant in all parts of basidiomata, encrusting hyphae and hymenial cells, small, angular to sand-like. Amorphous, brownish, faintly CB (+) substance abundant in senescent hymenium, partly covering hymenial cells. Hyphidia thin- to slightly thick-walled, often in groups of 3–10, with a cystidium-like, narrowly clavate-subfusiform or hyphoid stem, 19–85 × 2–10 μm, occasionally with CB (+), refractive, brownish contents, often bearing several richly branched apical outgrowths, 1–1.5 μm in diam. at the very apex; apical branches encrusted by small, densely arranged crystals. Basidia four-celled, broadly ellipsoid-ovoid to obconical, sessile, (14.5–) 15–24 (–25.5) × (8.8–) 9.1–15.3 (–15.6) μm (n = 60/5), thin-walled or with a distinct wall, embedded or exposed, sometimes with a short stalk-like base, up to 6 × 5 μm, sterigmata up to 35 × 3–3.5 μm; some basidia with a grayish-brownish cytoplasm. Basidiospores thin-walled, broadly to narrowly cylindrical, often distinctly curved, (10.2–) 11.2–18.0 (–18.1) × (4.5–) 4.8–7.7 (–7.8) μm (n = 210/7), L = 13.23–15.75, W = 5.33–6.67, Q = 2.37–2.91.

##### Remarks.

*Leiostroma farinellum* is widely distributed in the northern hemisphere and occurs almost exclusively on dead, often still corticated wood of conifers. We so far have only one verified collection of *L. farinellum* from an angiosperm host. In the central and southern parts of Europe, *L. farinellum* is clearly restricted to the mountain areas. ITS sequences of two Canadian collections show small differences from the sequences of *L. farinellum* from Europe and differences in *TEF1* region are even more substantial. However, we could not find any reliable morphological differences between the Eurasian and the North American specimens. Their identity deserves a closer look and the species name *Sebacina monticola* from Colorado (Burt 1915) should be kept in mind while revising the *L. farinellum* group in North America. Morphological differences between *L. farinellum* and the macroscopically similar *Adustochaete hiemalis*, *Leiostroma incanum*, *L. infecundum*, and *Proterochaete letendreana* are described in the remarks on these species.

Up to now, *Leiostroma farinellum* (at least its specimens from coniferous wood) was treated under the name *Exidiopsis calcea*. The latter combination made by Wells (1961) is based on *Thelephora calcea* Pers. (Persoon 1801). This name is, however, a homonym of the sanctioned *T. calcea* Fr. (Fries 1828), and it is thus unavailable (Code Art. 3.4). The identity of Fries’ *T. calcea* was discussed by Romell (1895), Litschauer (1938) and Rogers and Jackson (1943). They all univocally rejected its application to *T. calcea* Pers. *sensu* Bresadola (1898, as *Sebacina calcea*). We agree with their standpoint; the protologue of *T. calcea* Fr. in Elenchus clearly precludes *T. calcea* sensu Bres. and most likely refers to *Resinicium furfuraceum* (Bres.) Parmasto (= *Skvortzovia furfuracea* (Bres.) Gruhn & Hallenberg, Hymenochaetales). By moving Persoon’s species to *Sebacina*, Bresadola (1898) made this name available for a heterobasidiomycete species discussed here. However, his *Sebacina calcea* is preceded by *Xerocarpus farinellus* P. Karst. (Karsten 1882) and *Corticium abietis* (Fr.) Romell (Romell 1895), and it has never been typified. We therefore choose *X. farinellus* to make a new combination in *Leiostroma*.

The identity of *S. calcea* (= *E. calcea*) was mixed in all older sources, and we see no advantage in saving Persoon’s name via conservation. Even in North Europe, *S. calcea* was in use for at least two species, *L. farinellum* and *Tegmenticium peritrichum* (Romell’s specimens from deciduous hosts cited by Romell 1895; Neuhoff 1936b). Descriptions of the species by Bresadola (1898), Bourdot and Galzin (1927) and Malençon (1954) from the southern part of Europe and northern Africa were based on collections from both angiosperms and coniferous trees. In these geographic areas, they might have covered five morphologically similar species from four different genera, i.e. *Adustochaete hiemalis*, *Leiostroma farinellum*, *L. incanum*, *Proterochaete letendreana*, and *Ulocolla corticioides*. It is therefore difficult to argue in favor of conserving *T. calcea* Pers. as a well-known and widespread species.

#### Leiostroma incanum

Taxon classificationAnimaliaAuricularialesAuriculariaceae

Spirin & Viner sp. nov.

ADE2A655-E605-5883-BD47-DEC8DA24F19A

862482

Figs 25D, 27D

##### Holotype.

France • Alpes-Maritimes: Nice, Mont Boron, 43.69971; 7.30098, dead angiosperm branch, 17 Apr 2019, Viner 2019/280* (H).

##### Etymology.

Incanus (Lat., adj.) – whitened, faded.

##### Description.

Basidiomata annual, effused, up to 15 cm in the longest dimension, arid, soft, 0.1–0.3 mm thick. Hymenial surface smooth or indistinctly warted, grayish to mouse-gray, in older basidiomata brownish-gray or more rarely brown, occasionally minutely cracking (fissures seen with a lens only). Margin at first pruinose, then rather sharply delimited, adnate, white to grayish, up to 1 mm wide.

Hyphal structure monomitic, hyphae clamped; subicular hyphae hyaline, thin- to slightly thick-walled, interwoven or subparallel, occasionally glued in large bundles, 2–4 μm in diam., subhymenial hyphae hyaline or grayish-brownish, thin- to slightly thick-walled, ascending or interwoven, spaced or rather tightly arranged, occasionally twisted, 2–5 μm in diam., basidia-bearing hyphae thin- to slightly thick-walled, 2.5–5 μm in diam. Crystals abundant in all parts of basidiomata, encrusting hyphae and hymenial cells, small, angular to sand-like. Amorphous, brownish, faintly CB (+) substance abundant in senescent hymenium, partly covering hymenial cells. Hyphidia thin- to slightly thick-walled, often in groups of 3–7, with a cystidium-like, clavate-subfusiform or hyphoid stem, 22–44 × 3–11 μm, occasionally with CB (+), refractive, brownish contents, often bearing several richly branched apical outgrowths, 0.8–1.5 μm in diam. at the very apex; apical branches usually encrusted by small, densely arranged crystals. Basidia four-celled, ellipsoid-ovoid to obconical, sessile, (14.5–) 16–22 (–22.5) × (9.2–) 9.4–12.2 (–12.9) μm (n = 40/4), thin-walled or with a distinct wall, embedded or exposed, sometimes with a short stalk-like base, up to 5 × 5 μm, sterigmata up to 35 × 3–5 μm; some basidia with a grayish-brownish cytoplasm. Basidiospores thin-walled, broadly cylindrical to cylindrical, more or less clearly curved, (9.3–) 9.4–15.0 (–15.3) × (4.9–) 5.0–7.1 (–7.3) μm (n = 150/5), L = 11.28–12.83, W = 5.70–6.30, Q = 1.93–2.16.

##### Remarks.

*Leiostroma incanum* is a sister species of *L. farinellum*. It is distributed in South Europe and the Caucasus and restricted to angiosperm hosts. Macroscopically, it differs from *L. farinellum* in having deeper-colored, often densely cracking basidiomata. Microscopically, the two species can be differentiated primarily by the basidiospore length: spores of *L. incanum* are on average shorter than in *L. farinellum*. The species also have different ecological preferences: *L. farinellum* is a boreal – alpine species while *L. incanum* clearly prefers areas with a Mediterranean climate. Another species macroscopically similar to *L. incanum* and often occurring in the same habitats is *Proterochaete letendreana*. Their differences are listed under the latter species. See also notes to *L. infecundum* and *Ulocolla corticioides*.

Our data indicate the presence of yet another *Leiostroma* species on angiosperm hosts in the Mediterranean (see Suppl. material 4, Fig. 5, as *Leiostroma* sp. 2). It is so far known from a single specimen from Toulon, France. Based on one collection, it is difficult to judge about morphological differences between this taxon and *L. incanum*. Therefore, we are unwilling to formally introduce it in the present paper awaiting a denser sampling of *Leiostroma* spp. in the southern part of Europe and northern Africa.

#### Leiostroma infecundum

Taxon classificationAnimaliaAuricularialesAuriculariaceae

Viner & Spirin sp. nov.

69007F3F-3176-55FA-90B5-06D09EDF72F0

862483

Figs 25E, 27E

##### Holotype.

Spain • Canary Ids.: La Palma, San Miguel de La Palma, Puntallana, laurel forest, 28.76032; –17.78034, angiosperm wood, 30 Jul 2022, Viner 2022/1091A* (H).

##### Etymology.

Infecundus (Lat., adj.) – fruitless, infecund.

##### Description.

Basidiomata annual, effused, up to 5 cm in the longest dimension, arid, soft, 0.05–0.2 mm thick. Hymenial surface smooth, grayish to cream-colored, occasionally with poorly visible brownish flecks, in older basidiomata sometimes fading to almost white. Margin at first pruinose, then sharply delimited, adnate, concolourous with or slightly paler than hymenial surface, up to 1 mm wide.

Hyphal structure monomitic, hyphae clamped; subicular hyphae hyaline, with a distinct wall or slightly thick-walled, interwoven or subparallel, anastomosing, 2–5 μm in diam., subhymenial hyphae hyaline or more rarely grayish-brownish, thin- to slightly thick-walled, predominantly interwoven, anastomosing, spaced, occasionally twisted, 1–3.5 μm in diam., basidia-bearing hyphae with a distinct wall, 3–5 μm in diam. Crystals usually abundant in all parts of basidiomata, often encrusting hyphae and hymenial cells, small, angular to sand-like. Hyphidia thin- to slightly thick-walled, with cystidia-like, fusiform or hyphoid stem, 22–48 × 3–7.5 μm, bearing several richly branched apical outgrowths, 0.8–1.5 μm in diam. at the very apex; apical branches usually encrusted by small, densely arranged crystals. Basidia four-celled, ellipsoid-ovoid to obconical, sessile, (12–) 12.5–22.5 (–23) × (8.1–) 8.6–12.1 (–12.2) μm (n = 50/5), often with a distinct wall, usually embedded, sometimes with a short stalk-like base, up to 3 × 3 μm, sterigmata up to 35 × 3–5 μm; some basidia with a grayish-brownish cytoplasm. Basidiospores thin-walled, broadly to narrowly cylindrical, often more or less clearly curved, (9.5–) 9.8–15.8 × (4.7–) 4.8–6.6 (–6.9) μm (n = 115/5), L = 10.58–13.60, W = 5.08–6.02, Q = 1.84–2.27.

##### Remarks.

*Leiostroma infecundum* is a look-alike of *L. incanum* distributed in Macaronesia. The basidiomata of *L. infecundum* are in general paler, devoid of deep gray tints, and as a rule thinner than those of *L. incanum*. Hyphidia of *L. infecundum* are narrower and more regular in shape than in *L. incanum*. Additionally, the two species can be distinguished due to different distribution areas and via DNA sequences. Most collections of *L. infecundum* studied by us are sterile.

#### Leiostroma squalidum

Taxon classificationAnimaliaAuricularialesAuriculariaceae

Spirin & A. Savchenko sp. nov.

A6C28194-4F6F-506C-B396-2F9814D3F784

862484

Fig. 27F

##### Holotype.

Kenya • Taita-Taveta: Taita Hills, Ngangao Forest, –3.37043; 38.34067, corticated angiosperm branch, 24 Nov 2017, Savchenko 171124/1010* (H7008778, isotype – EA).

##### Etymology.

Squalidus (Lat., adj.) – rough, shaggy.

##### Description.

Basidiomata annual, effused, up to 15 cm in the longest dimension, arid, rather soft, 0.1–0.2 mm thick. Hymenial surface spinulose, cream-colored to gray or brownish-gray, spines rather regularly distributed, sharp-pointed, 100–200 × 30–60 μm, 6–9 per mm. Margin at first pruinose, then rather abruptly delimited, adnate, white to cream-colored, up to 1 mm wide.

Hyphal structure monomitic, hyphae clamped; subicular hyphae hyaline or brownish, variably thick-walled, interwoven or subparallel, often agglutinated, 1.5–4 μm in diam., subhymenial hyphae hyaline or brownish, thin- to slightly thick-walled, interwoven, rather densely arranged, in older parts occasionally agglutinated, 1.5–3.5 μm in diam., basidia-bearing hyphae thin- to slightly thick-walled, 3–5 μm in diam. hyphae of hymenial spines hyaline or brownish, slightly to distinctly thick-walled, ascending, often agglutinated, in older parts cemented by amorphous brownish substance, CB (+), 1.5–4 μm in diam. Crystals abundant in all parts of basidiomata, encrusting hyphae and hymenial cells, small, angular to sand-like. Hyphidia thin- to slightly thick-walled, single or in groups of 3–5, with narrowly clavate, subfusiform or hyphoid stem, 19–42 × 3–5 μm, bearing numerous richly branched apical outgrowths, 1–1.5 μm in diam. at the very apex; apical branches encrusted by small, densely arranged crystals. Basidia four-celled, narrowly ellipsoid-ovoid to obconical, sessile, (15–) 15.5–22 (–24) × (8.2–) 9.1–12.6 (–12.8) μm (n = 35/3), thin-walled or with a distinct wall, embedded or exposed, occasionally with reduced stalk-like base, up to 8 × 4.5 μm, sterigmata up to 30 × 3–4 μm, some basidia with a grayish-brownish cytoplasm. Basidiospores thin-walled, broadly cylindrical to cylindrical, clearly curved, (9.0–) 9.2–16.2 (–16.5) × (4.3–) 4.8–6.9 (–7.1) μm (n = 90/3), L = 11.47–14.26, W = 5.21–6.18, Q = 2.16–2.31.

##### Remarks.

*Leiostroma squalidum* is the only representative of the genus having a spinulose (i.e. *Heterochaete*-like) hymenophore. Otherwise, it is morphologically very close to *L. incanum* and *L. infecundum* described above. *Leiostroma squalidum* seems to be widely distributed in East Africa because its occurrence has been currently confirmed in both Kenya and Zimbabwe.

#### Pholiobasidion

Taxon classificationAnimaliaAuricularialesAuriculariaceae

Spirin & Grootmyers gen. nov.

20257706-F16F-50EA-8B9A-63C0F4C5CFF3

862485

##### Etymology.

Derived from ‘pholia’ (Greek, noun) – a nest, and ‘basidium’.

##### Description.

Basidiomata annual, effused, gelatinous, opaque, smooth or tuberculate, watery-grayish, margin gradually thinning, adnate. Hyphal structure monomitic, hyphae clamped, hyaline; basidia-bearing hyphae poorly differentiated. Cystidia abundant, hyaline, thin-walled, tapering-clavate. Hyphidia rare, simple or sparsely branched. Basidia four-celled, broadly ellipsoid to subglobose, sessile, thin-walled, often glued in groups of up to four and covered by shields of hyphidia and remnants of collapsed basidia. Basidiospores hyaline, thin-walled, broadly cylindrical to broadly ellipsoid, repetitive, apiculus small. On dead wood of angiosperms.

##### Generic type.

*Pholiobasidion senex*.

**Figure 29.**
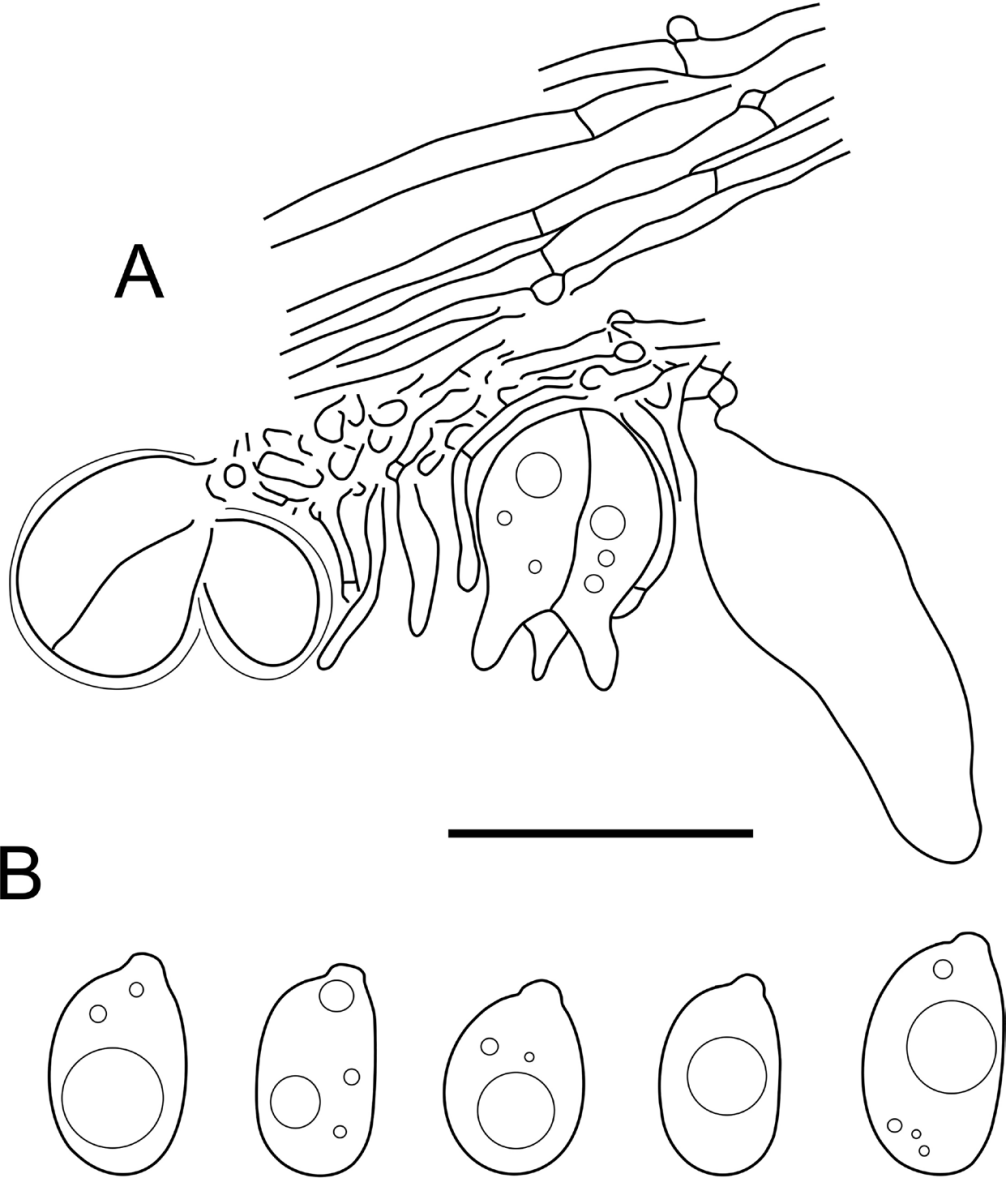
Microstructures of *Pholiobasidion senex* (holotype): **A** hyphae and hymenial cells; **B** basidiospores. Scale bar: 10 μm.

Our data show that *Pholiobasidion* is sister to the rest of the *Auriculariaceae* (Figs 1, 2). Whether it belongs to the family or should be treated separately from the *Auriculariaceae* is a question which cannot be solved without a much denser sampling among the residual *Auriculariales*. Morphologically, *Pholiobasidion* is strikingly similar to *Basidiodendron* spp. due to its small, broadly ellipsoid or subglobose basidia grouped together and covered by remnants of collapsed hymenial cells. However, the remaining morphological traits point elsewhere. In particular, there are no differentiated fishbone-like basidia-bearing hyphae in *Pholiobasidion* which are so characteristic of the core *Basidiodendron* spp. Moreover, cystidia of *P. senex* lack oily, strongly cyanophilous contents and should thus be considered as normal cystidia. In contrast, all species of *Basidiodendron* possess easily detectable gloeocystidia. Basidiospores of *Pholiobasidion* have a conventionally located, rather small and symmetric apiculus (Fig. 29) – they therefore look quite different from the spores of *Basidiodendron* (cf. Spirin et al. 2020, 2021). Phylogenetically, the core *Basidiodendron* spp. are related to *Protohydnum* and form a clade which is very distant from the *Auriculariaceae* (Spirin et al. 2025).

#### Pholiobasidion senex

Taxon classificationAnimaliaAuricularialesAuriculariaceae

Spirin & Grootmyers sp. nov.

C5C69984-6114-5A2E-B3E7-1A97C1B72A96

862486

Figs 25F, 29

##### Holotype.

USA. Ohio: Licking Co., Alexandria, 4235 Morse Rd., 40.0641; 82.5710, strongly decayed angiosperm log, 6 Oct 2019, Grootmyers (H*, isotype – TENN).

##### Etymology.

Senex (Lat., adj.) – ancient, old; in reference to grayish color of basidiomata.

##### Description.

Basidiomata annual, effused, at first appearing as small (up to 0.5 mm in diam.) adpressed-pustular bodies, then fusing together and forming a compound basidiome, up to 2 cm in widest dimension, gelatinous, 0.1–0.2 mm thick, opaque, smooth or tuberculate, watery-grayish, drying to a grayish crust. Margin poorly differentiated, gradually thinning, adnate.

Hyphal structure monomitic, hyphae clamped; subicular hyphae hyaline, with a distinct wall, predominantly subparallel, anastomosing, 1.5–2.5 (3) μm in diam., subhymenial hyphae hyaline, thin-walled, ascending or interwoven, sometimes embedded in gelatinous matrix, 1–2 μm in diam., basidia-bearing hyphae poorly differentiated, hyaline, thin-walled, 1–1.5 μm in diam. Cystidia abundant, hyaline, thin-walled, tapering-clavate, embedded or slightly projecting, 11–25 × 5–7.5 μm. Hyphidia rare, simple or sparsely branched, 1–1.5 μm in diam., scattered among basidia. Basidia four-celled, broadly ellipsoid to subglobose, sessile, (7.3–) 7.8–9.4 (–10.2) × (6.2–) 6.8–8.2 (–8.6) μm (n = 20/1), thin-walled, often glued in groups of up to four and covered by shields of hyphidia and remnants of collapsed basidia, sterigmata up to 5 × 1.5–2 μm. Basidiospores thin-walled, broadly cylindrical to broadly ellipsoid, (5.1–) 5.2–7.1 (–7.2) × (3.3–) 3.5–4.4 (–4.6) μm (n = 30/1), L = 6.18, W = 4.12, Q = 1.51.

##### Remarks.

*Pholiobasidion senex* is the only known species of the genus. It has so far only been found in the type locality in Ohio, USA, where it inhabited a strongly rotten angiosperm log. The differences of *P. senex* from the superficially similar *Basidiodendron* spp. are listed above.

#### Proterochaete

Taxon classificationAnimaliaAuricularialesAuriculariaceae

Spirin & Malysheva, Botany 97: 447, 2019

63614E48-E05B-5320-A99C-F50A8D580ADB

= Heterocorticium S.H. He, T. Nie & Yue Li, Journal of Fungi 9 (3, 318): 7, 2023. Generic type. Heterocorticium bambusicola S.H. He, T. Nie & Yue Li, Journal of Fungi 9 (3, 318): 7, 2023.

##### Description.

Basidiomata annual, effused, waxy and semitranslucent to arid and opaque, nearly white to gray or brownish to brown. Hymenial surface smooth or (in one species) spinulose. Hyphal structure monomitic; hyphae clamped, hyaline or rarely brownish, often densely arranged and at least partly agglutinated, basidia-bearing hyphae variably differentiated. Crystals occasional to abundant, usually angular. Cystidia present or absent. Hyphidia present, with a well-differentiated stem and richly branched, densely encrusted apical outgrowths, in some species bearing amorphous brown cyanophilous substance. Basidia four-celled, ellipsoid-ovoid to obconical, sessile, thin- or slightly thick-walled, exposed or embedded, occasionally with a reduced stalk-like base. Basidiospores thin-walled, cylindrical to nearly allantoid, curved, more rarely ellipsoid. On dead wood of angiosperms.

##### Generic type.

*Sebacina adusta* Burt.

The genus *Proterochaete* was introduced as monotypic, with *Sebacina adusta* as the sole species and generic type (Alvarenga et al. 2019). It was justified via phylogenetic evidence which showed that the species is quite isolated from the rest of the *Auriculariaceae* genera sampled at the time. Morphologically, *Proterochaete* was separated from the rest of the *Exidiopsis*-like taxa because of a highly peculiar combination of macroscopic traits in the type species, i.e. spinulose hymenophore and partly detaching, fimbriate and occasionally rhizomorphic basidiome margin. However, our current data impel us to expand the genus with ten more species, none of which has the aforementioned macroscopic features.

In the current scope, *Proterochaete* can be separated from the other *Exidiopsis / Heterochaete*-like genera of the *Auriculariacae* (*Adustochaete*, *Eichleriella*, *Exidiopsis* s. str., *Heteroradulum*, *Leiostroma*, and *Ulocolla* pr. p.) due to the following characters. First, all but two *Proterochaete* spp. possess abundant, small, as a rule angular crystals on their hyphae and hymenial cells. This feature rules out *Eichleriella*, *Exidiopsis*, *Tegmenticium*, and *Ulocolla* spp. Basidia of many *Proterochaete* spp. vary from ellipsoid-ovoid to obconical; in the latter case, they are reminiscent of the basidial cells in *Heteroradulum*. They are, however, devoid of the well-developed basal stalk characteristic of *Heteroradulum* spp. Moreover, most *Heteroradulum* spp. are dimitic while all species of *Proterochaete* are strictly monomitic. Differences between *Proterochaete* and *Adustochaete* are discussed under the latter genus.

**Figure 30.**
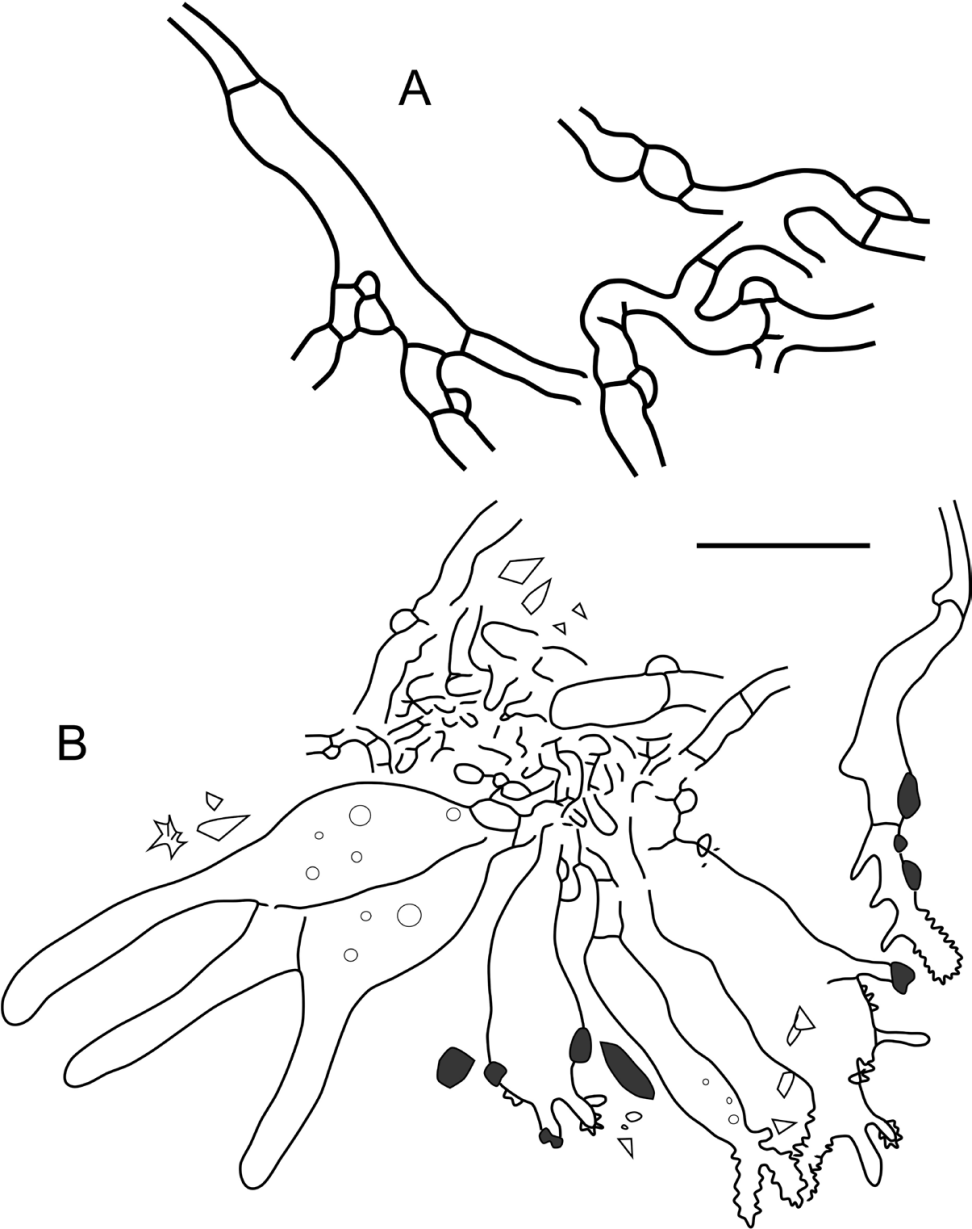
Microstructures of *Proterochaete letendreana* (Spirin 17866): **A** subicular hyphae; **B** subhymenial hyphae and hymenial cells bearing crystals and amorphous brown grains. Scale bar: 10 μm.

As for *Leiostroma*, it differs from the outwardly similar *Proterochaete* spp. (*P. africana*, *P. furfurosa*, *P. letendreana*, and *P. mucedinea*) in having softer basidiomata consisting of rather loosely arranged, not glued hyphae which often bear solid crystalline incrustation. In addition, hyphae and hyphidia of the listed *Proterochaete* spp. bear brownish amorphous granules well visible in CB (Fig. 30); these deposits are especially common in mature and senescent basidiomata where they fuse together and form large, amorphous, distinctly cyanophilous concretions. Moreover, most *Proterochaete* spp. have true cystidia and basidia-bearing hyphae of some species are poorly differentiated from the surrounding subhymenial hyphae. In contrast, all *Leiostroma* spp. described above are acystidiate and their basidia-bearing hyphae are wider than the subhymenial ones and thus easily detectable. See further remarks under *P. letendreana*.

*Proterochaete abstrusa* and *P. adusta* are the only species in the genus with a somewhat deviating morphology. They are totally devoid of a coarse crystalline encrustation, and their mature basidiomata are at least partly gelatinized, not waxy-arid as in other *Proterochaete* species. They are, however, otherwise very similar to other representatives of the genus, especially to *P. macrogyna* and *P. paniculata*. The superficially similar *Eichleriella* spp. can be separated from the mentioned *Proterochaete* species due to a distinctive subicular layer consisting of variably thick-walled, predominantly subparallel, spaced (not glued) and often brownish hyphae.

#### Proterochaete abstrusa

Taxon classificationAnimaliaAuricularialesAuriculariaceae

Spirin sp. nov.

43BF5EAE-F8DB-5A01-A59A-D51ED2CCBDB8

862487

Figs 31, 32A

##### Holotype.

Italy • Liguria: Imperia, Pigna, Buggio, 43.9625; 7.6880, *Carpinus betulus* (fallen, partly corticated branch), 17 Oct 2019, Spirin 13834* (H).

##### Etymology.

Abstrusus (Lat., adj.) – secret, hidden.

##### Description.

Basidiomata annual, effused, a few mm in diam., sometimes fusing together but rarely exceeding 1 cm in the widest dimension, at first waxy, then at least partly gelatinized, 0.05–0.1 mm thick, drying to a whitish or grayish, occasionally cracking shining crust. Hymenial surface at first opalescent, smooth, watery-grayish, then whitish, semitranslucent. Margin gradually thinning, adnate, first whitish, in older basidiomata concolourous with hymenial surface.

**Figure 31.**
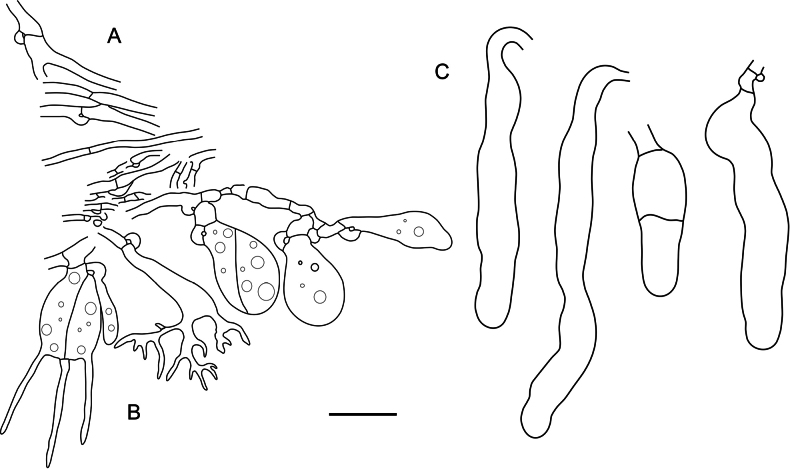
Microstructures of *Proterochaete abstrusa*: **A** subicular hyphae (holotype); **B** subhymenial hyphae and hymenial cells (holotype); **C** cystidia (Spirin 13943). Scale bar: 10 μm.

Hyphal structure monomitic, hyphae clamped, clamps often ansiform; subicular hyphae hyaline, thin-walled, subparallel, anastomosing, 1.5–4 (5) μm in diam., occasionally irregularly inflated (up to 9 μm in diam.), subhymenial hyphae hyaline, thin-walled, predominantly ascending, 1.5–4 μm in diam., rarely inflated and then reaching 5–7 μm in diam., easily collapsing, basidia-bearing hyphae well-differentiated, hyaline, thin-walled, 2–5.5 μm in diam., sometimes showing a zigzag-like branching pattern. Crystals occasionally present as large, rosette-like agglomerations in subhymenium, up to 25 μm in diam. Cystidia usually absent; if present, then thin-walled, narrowly clavate or tapering, embedded or projecting, 21–63 × 5–11.5 μm, occasionally bearing 1–3 inner septa. Hyphidia hyaline, richly branched, with a long hyphoid or narrowly clavate stem, 2–6 μm, and hyaline apical branches, 1–2 μm in diam., covering basidia and occasionally forming a continuous layer up to 15 μm thick. Probasidia narrowly to broadly clavate, more rarely tapering, 11–24 × 4–10 μm. Basidia four-celled, ellipsoid-ovoid or pyriform to subglobose, sometimes obliquely septate, sessile, (13–) 13.5–20.5 (–21.5) × (9.2–) 10.0–15.2 (–15.9) μm (n = 90/9), thin- or slightly thick-walled, predominantly embedded, occasionally with a tapering stalk-like base, up to 10 × 4.5 μm, sterigmata up to 35 × 2.5–4.5 μm. Basidiospores thin-walled, cylindrical to broadly cylindrical, curved, more rarely subfusiform, some spores slightly constricted at the middle and somewhat swollen at the apex, (11.1–) 11.3–23.8 (–24.2) × (4.8–) 4.9–9.1 (–9.2) μm (n = 278/9), L = 13.77–18.99, W = 5.52–7.38, Q = 2.02–2.72.

**Figure 32.**
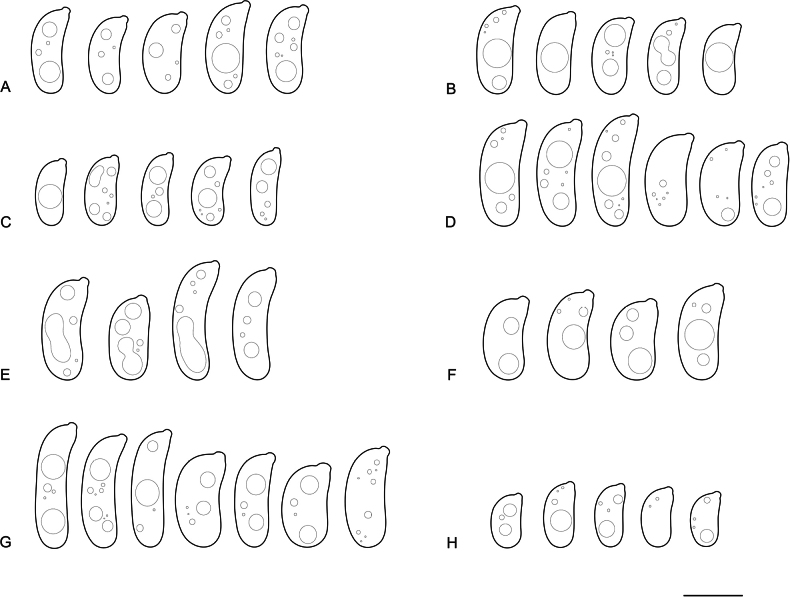
Basidiospores: **A***Proterochaete abstrusa* (holotype); **B***P. africana* (holotype); **C***P. furfurosa* (holotype); **D***P. letendreana* (three left – Spirin 17054, three right – holotype of *Sebacina letendreana*); **E***P. macrogyna* (holotype); **F***P. mucedinea* (Viner 2024/233); **G***P. paniculata* (three left – Spirin 8549, four right – paratype of *Exidiopsis paniculata*); **H***P. stipidosa* (holotype). Scale bar: 10 μm.

##### Remarks.

*Proterochaete abstrusa* is a European species occurring in temperate forests on various plant remnants, normally on fallen but still corticated tree branches. One specimen was collected from a dead grass stem. The species produces thin, hygroscopic basidiomata which become almost invisible when dry. This feature, as well as the absence of regular crystalline encrustation on hyphae and the presence of well-differentiated basidia-bearing hyphae make *P. abstrusa* a somewhat deviating representative of the genus. Morphologically, it is most similar to the North American *P. paniculata* (see description below). However, the latter species produces thicker, tougher, and as a rule darker basidiomata. Moreover, hyphae of *P. paniculata* often bear angular crystals and it has larger basidia. Hyphidia of *P. paniculata* have a sinuous, often irregularly inflated stem; in contrast, hyphidial stems of *P. abstrusa* are often poorly differentiated from the surrounding hyphae (“hyphoid”). Cystidia were totally lacking in all but three specimens of *P. abstrusa* studied by us. In those three specimens, they are abundant and easily observable, varying from narrowly clavate to distinctly tapering.

Senescent, partly gelatinized basidiomata of *P. abstrusa* can be mistaken for the resupinate *Exidia* species, i.e. *E. delita* and *E. plumbescens*. However, these species possess thicker basidiomata than *P. abstrusa*; their hymenial surface is as a rule tuberculate and whitish mineral inclusions are often present. Additionally, *E. plumbescens* can be differentiated from *P. abstrusa* due to visibly swollen, subfusiform stems of the hyphidia. Hyphidia of *E. delita* are rather poorly differentiated, like in *P. abstrusa*, but its basidiospores are slightly shorter and clearly narrower than in the latter species. It should be noted, however, that the size of the basidiospores is exceptionally variable in *P. abstrusa* and cannot be used as the single trait to distinguish between it and similar species.

#### Proterochaete adusta

Taxon classificationAnimaliaAuricularialesAuriculariaceae

(Burt) Spirin & Malysheva, Botany 97: 447, 2019

2035B2F1-083B-509A-A812-CBA771228AF8

≡ Sebacina adusta Burt, Annals of the Missouri Botanical Garden 2: 764, 1915. Lectotype (selected by Alvarenga et al. 2019: 447). USA. Idaho: Coolin, Populus trichocarpa, Sep 1915, Weir 3118 (BPI 723685, studied).
For a description and further synonyms, see Alvarenga et al. (2019). Rödel et al. (2020) published excellent macro- and microphotographs of *P. adusta*.

#### Proterochaete africana

Taxon classificationAnimaliaAuricularialesAuriculariaceae

Spirin & Ryvarden sp. nov.

9C2AD4D7-F6CB-554E-9AA7-CBFB6BD3B710

862489

Fig. 32B

##### Holotype.

Uganda • Kabarole: Kibale National Park, Makere University Field Station, fallen corticated branch, 20 Apr 2002, Ryvarden 44921* (O, isotype – H).

##### Etymology.

Africanus (Lat., adj.) – African.

##### Description.

Basidiomata annual, effused, up to 3 cm in the longest dimension, arid, soft, 0.05–0.1 mm thick. Hymenial surface smooth or indistinctly tuberculate, at first pale ochraceous, then changing to ochraceous-grayish, in older basidiomata irregularly cracking and occasionally covered by dark reddish-brown dots. Margin at first floccose or nearly fibrillose, gradually thinning, whitish, then sharply delimited, compact, adnate, concolourous with hymenial surface, up to 0.5 mm wide.

Hyphal structure monomitic, hyphae clamped; subicular hyphae hyaline, slightly-walled, subparallel, anastomosing, 2–3.5 μm in diam., subhymenial hyphae hyaline, thin- to slightly thick-walled, predominantly interwoven, 1.2–3 μm in diam., basidia-bearing hyphae poorly differentiated, with a distinct wall, 1.8–2.5 μm in diam. Crystals abundant in all parts of basidiomata, encrusting hyphae and hymenial cells, as a rule small, angular; amorphous brownish or rusty-brown, CB (+) concretions present, especially abundant in senescent parts. Needle-like crystals appearing in CB-mounted microscopic slides after 2–3 hours. Cystidia absent. Hyphidia with a thin- to slightly thick-walled, hyaline or rusty-brown, variably shaped (subfusiform, hyphoid or narrowly clavate) stem, 23–48 × 4–10 μm, often with a refractive, CB+ content, bearing several richly branched, hyaline apical outgrowths, 0.8–1.2 μm in diam. at the very apex, partly covering basidia; apical branches usually encrusted by small, densely arranged crystals and occasionally bearing brownish amorphous grains. Basidia four-celled, obconical or more rarely ellipsoid-ovoid, sessile, (16.5–) 17–25.5 (–26) × (9.2–) 9.4–11.8 (–12.0) μm (n = 20/1), thin-walled or with a distinct wall, exposed or partly embedded, rarely with a short stalk, up to 3 × 3 μm, sterigmata up to 20 × 3–3.5 μm. Basidiospores thin-walled, broadly cylindrical to cylindrical, usually distinctly curved, longest spores rarely sigmoid, (11.7–) 12.0–15.2 (–15.3) × (5.2–) 5.3–6.8 (–6.9) μm (n = 30/1), L = 13.55, W = 6.08, Q = 2.23.

##### Remarks.

*Proterochaete africana* is one of two *Proterochaete* species so far detected in Africa. It is morphologically similar to *P. furfurosa*, described below. The two species can be separated by the presence of cystidia (lacking in *P. africana*, present in *P. furfurosa*), as well as the size of basidia and basidiospores (distinctly larger in *P. africana* than in *P. furfurosa*). *Proterochaete africana* is known so far only from the type locality in Uganda.

#### Proterochaete bambusicola

Taxon classificationAnimaliaAuricularialesAuriculariaceae

(S.H. He, T. Nie & Yue Li) Spirin comb. nov.

B8136071-B9B9-5CE0-AE13-7A95F07A2277

862490

≡ Heterocorticium bambusicola S.H. He, T. Nie & Yue Li, Journal of Fungi 9 (3, 318): 7, 2023. Holotype. China. Jiangxi: Yifeng, Guanshan Nat. Reserve, culm of dead bamboo, 10 Aug 2016, He 4236* (BJFC 023678).

##### Note.

This species was introduced by Li et al. (2023) based on specimens from China and Malaysia. It is the generic type of *Heterocorticium*. Morphologically, *H. bambusicola* is most similar to *Proterochaete stipidosa* introduced below. However, it can be distinguished from the latter species by its longer basidiospores and the lack of cystidia. DNA sequences show that *H. bambusicola* and *P. stipidosa* are not closely related (Figs 3, 4) although they both belong to the redefined *Proterochaete*. We therefore combine *H. bambusicola* in *Proterochaete*.

#### Proterochaete furfurosa

Taxon classificationAnimaliaAuricularialesAuriculariaceae

Spirin & Viner sp. nov.

B5A14CA1-7EA1-5683-B28D-C87D067001E8

862491

Fig. 32C

##### Holotype.

Uganda • Kabarole: Kibale National Park, Makere University Field Station, fallen corticated branch, 20 Apr 2002, Ryvarden 44895* (O, isotype – H).

##### Etymology.

Furfurosus (Lat., adj.) – bran-like.

##### Description.

Basidiomata annual, effused, up to 5 cm in the longest dimension, waxy to arid, soft, 0.05–0.1 mm thick. Hymenial surface smooth, at first pale ochraceous, then changing to hazel-colored, in older basidiomata irregularly cracking and dark grayish-brown. Margin at first fibrillose, gradually thinning, whitish, then sharply delimited, cream-colored to grayish-brown, up to 0.5 mm wide.

Hyphal structure monomitic, hyphae clamped; subicular hyphae hyaline or rarely brownish, slightly to distinctly thick-walled, subparallel or interwoven, anastomosing, often covered by crystalline shields, 1.5–4.5 μm in diam., subhymenial hyphae hyaline or rarely brownish, thin- to slightly thick-walled, predominantly interwoven, 1.5–3 μm in diam., basidia-bearing hyphae poorly differentiated, with a distinct wall, 2–4 μm in diam. Crystals abundant in all parts of basidiomata, encrusting hyphae and hymenial cells, as a rule small, angular; amorphous brownish or rusty-brown, CB(+) concretions present, especially abundant in senescent and dark-colored specimens. Needle-like crystals appearing in CB-mounted microscopic slides after 2–3 hours. Cystidia abundant to rare, hyaline, thin-walled, distinctly tapering, strongly swollen at the base, more rarely clavate, sometimes irregularly constricted, 13–44 × 5–9 μm, occasionally with a refractive, CB+ contents. Hyphidia hyaline, thin- to slightly thick-walled, with a hyphoid, sinuous stem, 12–32 × 2–5.5 μm, in older basidiomata with a refractive, CB+ contents, bearing several richly branched, hyaline apical outgrowths, 0.8–1.2 μm in diam. at the very apex, covering basidia and often forming a continuous layer up to 10 μm thick; apical branches usually encrusted by small, densely arranged crystals and occasionally bearing brownish amorphous matter. Basidia four-celled, ellipsoid-ovoid to nearly obconical, sessile, (13.5–) 14–20.5 (–22) × (7.2–) 8.8–11.3 (–12.2) μm (n = 33/3), thin- to slightly thick-walled, normally embedded, sometimes with a short stalk, up to 5 × 4 μm, sterigmata up to 15 × 2–3 μm; basidia in senescent hymenium with a distinctly thickened, gelatinized wall (up to 2 μm thick) and brownish cytoplasm. Basidiospores thin-walled, broadly to narrowly cylindrical, usually distinctly curved, (9.0–) 9.1–13.4 (–13.8) × (3.9–) 4.0–6.1 (–6.2) μm (n = 90/3), L = 10.49–11.46, W = 4.62–5.50, Q = 2.09–2.41, in older hymenium often with a distinct wall and brownish cytoplasm, glued in large groups.

##### Remarks.

*Proterochaete furfurosa* is morphologically similar and phylogenetically close to *P. africana* described above. Their differences are mentioned under the latter species. It is probably widely distributed in East Africa. Our current records confirm its presence in Uganda and Zimbabwe. Faded basidiomata of *P. furfurosa* are superficially similar to *Adustochaete scalena* occurring in the same geographic area. However, basidia and basidiospores of *A. scalena* are distinctly larger than those of *P. furfurosa*. Moreover, it lacks brownish amorphous deposits which are as a rule abundant in *P. furfurosa*.

#### Proterochaete latispora

Taxon classificationAnimaliaAuricularialesAuriculariaceae

(S.H. He, T. Nie & Yue Li) Spirin comb. nov.

49EDCC6F-EF71-5F06-9804-27DA19D852FD

862492

≡ Heterocorticium latisporum S.H. He, T. Nie & Yue Li, Journal of Fungi 9 (3, 318): 10, 2023. Holotype. China. Sichuan: Qionglai, Tiantaishan Forest Park, fallen angiosperm branch, 23 Sep 2012, He 20120923-20* (BJFC 014667).

##### Note.

This species was introduced as a member of *Heterocorticium* by Li et al. (2023) and is so far known from two localities in China. Our data place it in *Proterochaete* (Figs 3, 4). *Proterochaete latispora* differs from all other species of the genus in having ellipsoid basidiospores.

#### Proterochaete letendreana

Taxon classificationAnimaliaAuricularialesAuriculariaceae

(Pat.) Spirin comb. nov.

DB8FAE90-3965-554F-896A-431B723F35A0

862493

**Figure 33.**
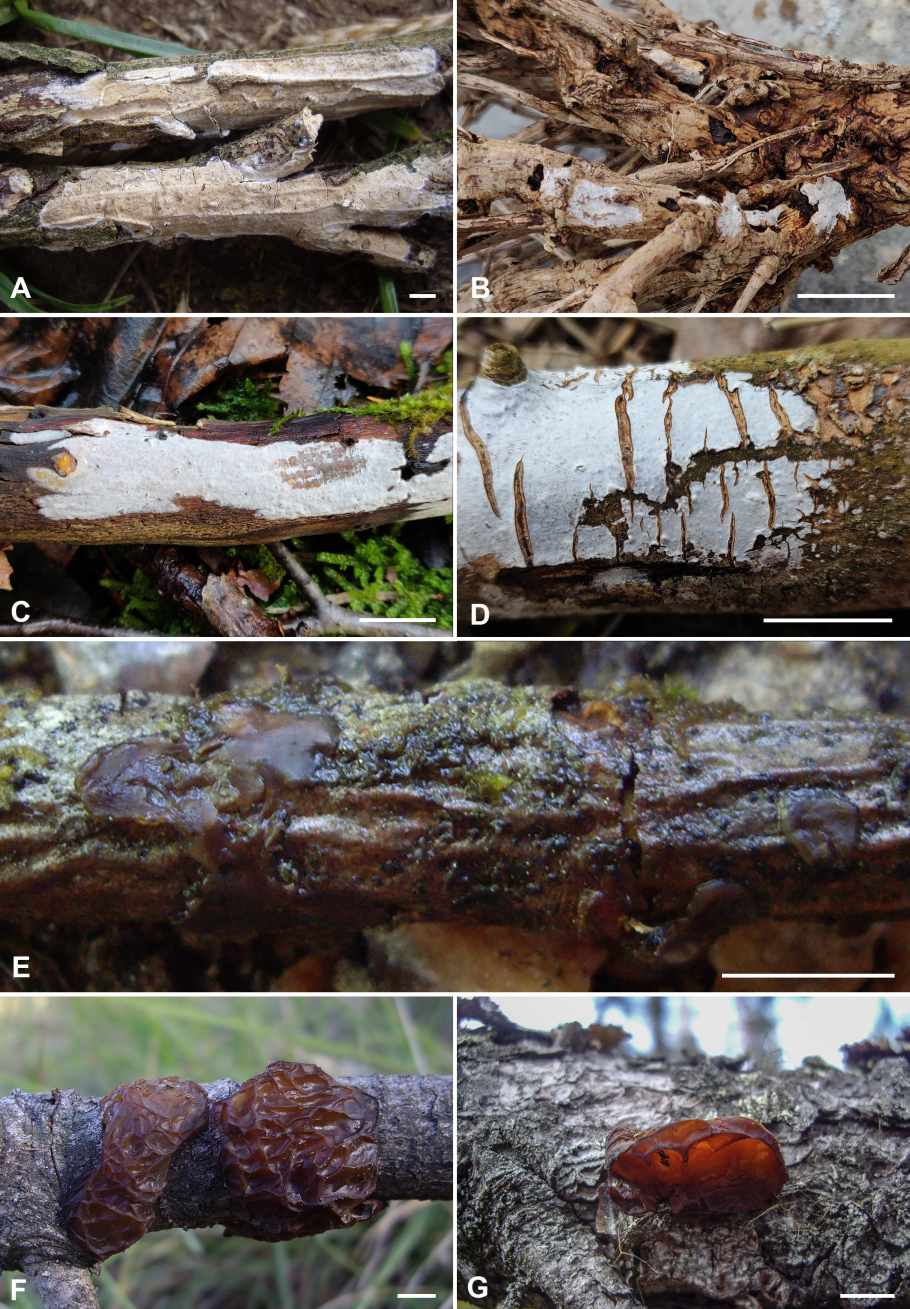
Basidiomata: **A***Proterochaete letendreana* (Spirin 15652); **B***P. letendreana*, pale form (Spirin 18184); **C***Tegmenticium peritrichum* (Spirin 18537); **D***Ulocolla corticioides* (Spirin 18923); **E***Ulocolla orbiculata* (holotype); **F***Ulocolla saccharina* (Spirin 13748); **G***U. subrepanda* (Spirin 6841). Scale bar: 1 cm.

Figs 30, 32D, 33A, B

≡ Sebacina letendreana Pat., Revue Mycologique 7: 152, 1885. Holotype. France. Seine-Maritime: Rouen, Le Grand-Quevilly, Colpoma quercinum and adjacent bark, 1883, Letendre (FH00784209, studied).

##### Description.

Basidiomata annual, effused, up to 10 cm in the longest dimension, waxy to arid, soft, 0.05–0.5 mm thick. Hymenial surface smooth or irregularly warted, at first pure white to pale cream-colored, slowly turning to bluish-gray, then changing to light brownish-gray or chocolate-brown, in older basidiomata irregularly cracking and fading to grayish-white or nearly white. Margin at first pruinose to fibrillose, gradually thinning, white to grayish, then sharply delimited, sometimes slightly elevated, compact, cream-colored, up to 1 mm wide.

Hyphal structure monomitic, hyphae clamped; subicular hyphae hyaline or rarely brownish, thin- to slightly thick-walled, subparallel or interwoven, 2–5 μm in diam., subhymenial hyphae hyaline, thin-walled or with a distinct wall, ascending or interwoven, 1.5–4 μm in diam., basidia-bearing hyphae thin-walled or with a distinct wall, 2.5–5 (6) μm in diam., in some specimens poorly differentiated. Crystals usually abundant in all parts of basidiomata, often encrusting hyphae, in juvenile and vigorously growing basidiomata predominantly small, angular or sand-like, then variably shaped; amorphous brownish or rusty-brown, CB+ concretions present, especially abundant in senescent and dark-colored specimens. Cystidia absent. Hyphidia hyaline or brownish, thin- to slightly thick-walled, with a cystidia-like, fusiform or hyphoid stem, 14–36 × 2–6 μm, bearing several richly branched, hyaline or brownish apical outgrowths, 1–2 μm in diam. at the very apex; apical branches usually encrusted by small, densely arranged crystals and brownish amorphous matter. Basidia four-celled, ellipsoid-ovoid to obconical, sessile, (14–) 14.5–22.5 (–25) × (9.2–) 9.4–15.8 (–17.6) μm (n = 132/13), thin-walled (in senescent hymenium slightly thick-walled and agglutinated), normally embedded, sometimes with a short stalk, up to 5 × 4 μm, sterigmata up to 30 × 3.5–4 μm. Basidiospores thin-walled, broadly to narrowly cylindrical, slightly to distinctly curved, (9.3–) 10.1–21.8 (–24.7) × (4.6–) 4.7–7.2 (–7.6) μm (n = 600/21), L = 12.10–16.23, W = 5.19–6.63, Q = 2.10–2.78.

##### Remarks.

*Proterochaete letendreana* is widely distributed in Europe, from the Mediterranean to the southern part of Finland. It occurs predominantly on still hanging or just fallen, often corticated branches of many angiosperm tree species, as well as on dead shrub and grass stems and lianas, and produces basidiomata throughout the year. *Proterochaete letendreana* is exceptionally variable in both macroscopic and microscopic terms, and it is not surprising that its identity remained obscure prior to the present study.

This species was described as *Sebacina letendreana* by Patouillard (1885) from the north-western part of France. The type specimen, although small, is in a very good condition, fertile, and represents *P. letendreana* in its typical mature state, i.e. showing a brown hymenium and paler margin. Bourdot and Galzin (1927) interpreted it as a form of their broadly defined *Sebacina calcea*. Since then, *S. letendreana* had been treated merely as a synonym of *Exidiopsis calcea* sensu auct. (Pilát 1957; Wells 1961; Donk 1966; Wells and Raitviir 1977). The first sequence of *P. letendreana* was published by Weiß and Oberwinkler (2001) and named *Exidiopsis* sp. (GenBank AF291282). A few other sequences of this species were submitted to GenBank afterwards, some of them mislabelled as *Exidiopsis effusa* (e.g., MZ159758).

Morphologically, *P. letendreana* is similar to three other species occurring in Europe on angiosperm hosts, i.e. *Adustochaete hiemalis*, *Leiostroma farinellum*, and *L. incanum*. The latter three species possess softer basidiomata than *P. letendreana*, consisting of rather loosely arranged, spaced hyphae, and none of them has brownish, amorphous, cyanophilous deposits among their tissues. These deposits can be very rare, however, especially in pale-colored juvenile or faded senescent basidiomata of *P. letendreana*. In these cases, *P. letendreana* can be separated from *A. hiemalis* due to its smaller basidia and the lack of cystidia, as well as partly glued hyphae. In turn, the two *Leiostroma* species have much more extensive crystalline encrustation than *P. letendreana*, often making continuous, solid shields on their hyphae. Moreover, some basidia and subhymenial hyphae of *Leiostroma* spp. occasionally bear grayish-brownish contents. These structures are hyaline in *P. letendreana*. Hyphidia of *L. farinellum* and *L. incanum* often form groups of 3–7, while they are usually single and quite irregularly arranged in *P. letendreana*.

#### Proterochaete macrogyna

Taxon classificationAnimaliaAuricularialesAuriculariaceae

(K. Wells & Raitviir) Spirin & A. Savchenko comb. nov.

A1E6D424-3860-53BB-859E-D1B6B71E28C5

862494

Fig. 32E

≡ Exidiopsis griseobrunnea ssp. macrogyna K. Wells & Raitviir, Mycologia 69: 1002, 1977. Holotype. Russia. Yakutia: Eveno-Bytantaysky Nat. Dist., Batagai-Alyta, Alnus fruticosa (decayed branch), 12 Aug 1972, Parmasto (TAAM 56485*, studied).

##### Description.

Basidiomata annual, effused, orbicular, gregarious, up to 1 cm in diam., soft, up to 0.2 mm thick. Hymenial surface smooth or indistinctly tuberculate, ochraceous-brownish to brown, in older parts partly gelatinized and fading to light grayish-brown, at first continuous, then deeply cracking. Margin at first white, adnate, pubescent, up to 0.3 mm wide, then concolourous with hymenial surface, sharply delimited, sometimes partly detaching.

Hyphal structure monomitic, hyphae clamped, occasionally CB(+); subicular hyphae hyaline, slightly to distinctly thick-walled (walls gelatinized), mostly subparallel, frequently anastomosing, sometimes densely encrusted by angular crystals, variably inflated, 2–7 μm in diam., subhymenial hyphae hyaline or yellowish, thin- to slightly thick-walled, interwoven or ascending, rather loosely arranged, in older hymenium partly agglutinated, 1.5–4.5 μm in diam., often twisted, occasionally irregularly inflated up to 6–7 μm in diam., basidia-bearing hyphae not differentiated. Crystals abundant or rare, distributed in all parts of basidiomata, encrusting hyphae, small, angular. Cystidia rare, thin- to slightly thick-walled, tapering-clavate, often sinuous and sometimes producing sparsely branched side outgrowths, 22–31 × 3.5–8 μm. Hyphidia hyaline, with a distinct wall or slightly thick-walled, with a hyphoid, often sinuous stem, 22–31 × 2–3 μm, bearing several richly branched apical outgrowths, 1–1.5 μm in diam. at the very apex; apical branches rarely encrusted by small, sparsely arranged crystals, usually forming a continuous layer up to 25 μm thick. Basidia four-celled, ellipsoid-ovoid, sessile, (17–) 17.5–25 (–27) × (11.4–) 11.9–15.2 (–15.4) μm (n = 41/3), usually thin-walled (obtaining a distinct wall and partly agglutinated in senescent hymenium), mostly embedded, occasionally with a reduced stalk-like base, up to 5 × 4 μm, sterigmata up to 22 × 2.5–3 μm. Basidiospores thin-walled, cylindrical or cylindrical-subfusiform to nearly allantoid, more rarely broadly cylindrical, slightly or clearly curved, (12.9–) 13.0–21.8 (–23.3) × (5.3–) 5.4–7.1 (–7.3) μm (n = 92/3), L = 16.99–18.25, W = 6.14–6.42, Q = 2.66–2.98.

##### Remarks.

This taxon was described as a subspecies of *Exidiopsis griseobrunnea* (Wells and Raitviir 1977). Our data show that these taxa are not conspecific or even closely related, although they are macroscopically quite similar and occur on the same host tree (*Alnus fruticosa*) in North Asia. *Exidiopsis griseobrunnea ssp. macrogyna* is a sister species of the North American *E. paniculata*, and they both belong to *Proterochaete*, while *E. griseobrunnea* s. str. is a member of *Ulocolla* (Figs 1, 2). Two paratypes of *E. griseobrunnea* ssp. macrogyna (TAAM 45888, TAAM 56815) are not conspecific with the holotype, and therefore they are excluded from the description above.

*Proterochaete macrogyna* is a distinctive species due to its small, orbicular, brown basidiomata with partly elevated and detaching margins. Microscopically, large basidia and basidiospores, as well as the presence of crystals easily separate it from *Ulocolla griseobrunnea* inhabiting the same host. The species is so far known from a few localities with a strongly continental climate in Yakutia, Siberia.

#### Proterochaete mucedinea

Taxon classificationAnimaliaAuricularialesAuriculariaceae

(Pat.) Spirin & Viner comb. nov.

ED3EBB2C-8FA9-5FE0-B1C2-28C15AC42128

862495

Fig. 32F

≡ Sebacina mucedinea Pat., Bulletin de l’Herbier Boissier 3: 60, 1895. Holotype. Ecuador. Pichincha: Quito, Pululahua, twigs on the ground, Feb 1892 Lagerheim (FH00784210, studied).
= Exidiopsis mucedinea (Pat.) K. Wells, Lloydia 20: 46, 1957.

##### Description.

Basidiomata annual, effused, at first small, patch-like, then fusing together and up to 8 cm in the longest dimension, arid, compact, up to 0.1 mm thick. Hymenial surface smooth, cream-colored to grayish, with occasional brownish spots, continuous or finely cracking. Margin at first whitish, pruinose, up to 0.3 mm wide, then concolourous with hymenial surface, rather clearly delimited.

Hyphal structure monomitic, hyphae clamped; subicular hyphae hyaline or brownish, variably thick-walled, subparallel, tightly arranged, 1.5–3 μm in diam., subhymenial hyphae hyaline, thin- to slightly thick-walled, interwoven, rather densely arranged and partly agglutinated, 2–3 μm in diam., basidia-bearing hyphae often well-differentiated, hyaline, slightly thick-walled, 3–5 μm in diam. Crystals abundant in all parts of basidiomata, encrusting hyphae, small, angular. Cystidia rare, thin- to slightly thick-walled, tapering or almost clavate, 18–33 × 4–8 μm. Hyphidia hyaline or brownish, thin- to slightly thick-walled, with hyphoid stem, 18–38 × 2.5–4.5 μm, bearing several richly branched apical outgrowths, 1–2 μm in diam. at the very apex; apical branches encrusted by small, densely arranged crystals or covered by amorphous, brownish or rusty-brown, CB (+) substance. Basidia four-celled, narrowly ovoid to almost obconical, sessile, (16.5–) 17–20.5 (–22) × (8.2–) 9.3–13.2 (–13.4) μm (n = 33/2), thin-walled or with a distinct wall, embedded or exposed, occasionally with a reduced stalk-like base, up to 7 × 5 μm, sterigmata up to 30 × 3–4 μm. Basidiospores thin-walled, broadly cylindrical, mostly clearly curved, (10.1–) 10.2–16.2 (–16.8) × (5.2–) 5.8–7.2 (–8.2) μm (n = 62/2), L = 13.06–13.24, W = 6.48–6.55, Q = 2.00–2.05.

##### Remarks.

Patouillard and Lagerheim (1895) described this species from Ecuador as *Sebacina mucedinea* and mentioned *S. letendreana* from Europe as its look-alike. Here we reinstate *S. mucedinea* as a species of *Proterochaete*. The holotype of *S. mucedinea* is scanty and substerile (only two turgid spores were found) but with a well-developed hymenium. Morphologically, it is nearly identical to two recent specimens from Argentina used for DNA study. Macroscopically, *P. mucedinea* is very similar to another neotropical species, *P. stipidosa* (see description below). These species can be separated from each other primarily by the size of their basidia and basidiospores (clearly smaller in *P. stipidosa* than in *P. mucedinea*).

#### Proterochaete paniculata

Taxon classificationAnimaliaAuricularialesAuriculariaceae

(K. Wells & Bandoni) Spirin & Viner comb. nov.

F740719B-BD58-5ECC-867A-C9EA27CC6276

862496

Fig. 32G

≡ Exidiopsis paniculata K. Wells & Bandoni, Mycologia 79: 283, 1987. Holotype. Canada. British Columbia: Vancouver, University of British Columbia Campus, Robinia pseudoacacia (fallen decorticated branch), 6 Jan 1983, Bandoni 6931 (BPI 871729 – isotype, studied).

##### Description.

Basidiomata annual, effused, often gregarious, up to 2 cm in the longest dimension, waxy, up to 0.15 mm thick. Hymenial surface smooth or indistinctly tuberculate, light ochraceous to brownish, in older parts partly gelatinized and fading to grayish-white, at first continuous, then deeply cracking. Margin at first white, pruinose or pubescent, up to 1 mm wide, then concolourous with hymenial surface, sharply delimited.

Hyphal structure monomitic, hyphae clamped, occasionally CB(+); subicular hyphae hyaline, slightly to distinctly thick-walled (walls gelatinized), subparallel, often in tightly arranged bundles (up to 50 μm in diam.), frequently anastomosing, 2–5 μm in diam., subhymenial hyphae hyaline, thin- to slightly thick-walled, interwoven or ascending, rather loosely arranged, in older hymenium partly agglutinated, 2–6 (7) μm in diam., basidia-bearing hyphae usually well-differentiated, hyaline, thin-walled, 3–5 μm in diam., showing a zigzag-like branching pattern. Crystals abundant or rare, distributed in all parts of basidiomata, encrusting hyphae, small, angular. Needle-like crystals appearing in CB-mounted microscopic slides after 2–3 hours. Cystidia absent. Hyphidia hyaline, thin-walled or with a distinct wall, with a hyphoid or subfusiform, often sinuous stem, 19.5–52 × 4–8.5 μm, bearing several richly branched apical outgrowths, 1–2 μm in diam. at the very apex; apical branches encrusted by small, sparsely arranged crystals or bearing small brownish resinous droplets, occasionally forming a continuous layer up to 10 μm thick. Basidia four-celled, predominantly ellipsoid-ovoid, more rarely nearly obconical, sessile, (14–) 14.5–22 (–24) × (10.7–) 11.1–15.7 (–15.8) μm (n = 53/5), usually thin-walled (obtaining a distinct wall and partly agglutinated in senescent hymenium), mostly embedded, occasionally with a reduced stalk-like base, up to 5 × 3 μm, sterigmata up to 35 × 3–4 μm. Basidiospores thin-walled, cylindrical or cylindrical-subfusiform to nearly allantoid, mostly clearly curved, (11.0–) 12.0–21.0 (–23.2) × (4.3–) 4.6–7.1 (–7.2) μm (n = 110/4), L = 14.58–17.39, W = 5.14–5.82, Q = 2.54–3.39.

##### Remarks.

*Exidiopsis paniculata* was described from British Columbia, Canada, and previously known only from the type locality (Wells 1987). We succeeded in sequencing the paratype of *E. paniculata* from the locus classicus and could connect it to our recent specimens from Washington, USA. The improved species description above is based on type studies and morphological evidence from the US collections. Our data show that *E. paniculata* belongs to *Proterochaete* and that it is the closest relative of the North Asian *P. macrogyna* described above (Figs 1, 2). According to our observations, the spore variation range in *P. paniculata* is wider than mentioned in the protologue.

*Proterochaete paniculata* produces small, dark-colored, resupinate basidiomata on corticated, attached or freshly fallen branches of angiosperms. It is probably widely distributed along the North American Pacific coast and just overlooked due to its small size and dark coloration. Morphologically, it is highly similar to the European *P. letendreana*. The latter species, however, does not show any signs of gelatinization of the hymenium, while the hymenium layer is at least partly gelatinized and hygroscopic in older basidiomata of *P. paniculata*. The sister species of *P. paniculata*, *P. macrogyna*, has orbicular basidiomata with a partly detached margin (vs. corticioid basidiomata with adnate margin in *P. paniculata*). Moreover, it has wider hyphae, as well as wider and more clearly curved basidiospores than *P. paniculata*. *Proterochaete paniculata* is an acystidiate species. *Proterochaete macrogyna* has hymenial cystidia although they are usually quite rare. Differences between *P. paniculata* and *P. abstrusa* are discussed under the latter species.

#### Proterochaete stipidosa

Taxon classificationAnimaliaAuricularialesAuriculariaceae

Viner & Spirin sp. nov.

3C01854D-21E6-5415-86E3-DCCFAEF68BC3

862497

Fig. 32H

##### Holotype.

Argentina • Misiones: Iguazu, Reserva Selva Iryapú, near IMiBio (Instituto Misionero de Biodiversidad), secondary forest, -25.61405, -54.55692, angiosperm branch, 7 May 2024, Viner 2024/266* (H, isotype – SLP).

##### Etymology.

Stipidosus (Lat., adj.) – woody, ligneous.

##### Description.

Basidiomata annual, effused, up to 3 cm in the longest dimension, arid, compact, up to 0.05 mm thick. Hymenial surface smooth, cream-colored to grayish, continuous or finely cracking. Margin pruinose, up to 0.3 mm wide, concolourous with hymenial surface.

Hyphal structure monomitic, hyphae clamped, CB (+); subicular hyphae hyaline, with a distinct wall, subparallel, tightly arranged, 1.5–3 μm in diam., subhymenial hyphae hyaline, with a distinct wall, interwoven or ascending, densely arranged and partly agglutinated, 1.5–4 μm in diam., basidia-bearing hyphae well-differentiated, hyaline, with a distinct wall, 3–5 μm in diam. Brownish deposits abundant in all parts of basidiomata, encrusting hyphae and hymenial cells, small, angular, CB+. Cystidia (gloeocystidia) abundant, hyaline or brownish, thin- to slightly thick-walled, tapering or clavate, 20–34 × 5–9.5 μm, rarely bi- or trifurcate, contents homogeneous, CB (+). Hyphidia hyaline or brownish, thin- to slightly thick-walled, with a hyphoid stem, 17–43 × 2–6 μm, bearing several richly branched apical outgrowths, 1–1.5 μm in diam. at the very apex, some hyphidia with CB+ contents. Basidia four-celled, ellipsoid-ovoid, more rarely nearly obconical, sessile, (12–) 13–15 (–15.5) × (7.3–) 7.8–9.2 (–10.2) μm (n = 23/1), with a distinct wall or slightly thick-walled, embedded or exposed, occasionally with a reduced stalk-like base, up to 3 × 3 μm, sterigmata up to 25 × 2–3 μm. Basidiospores thin-walled, broadly cylindrical to cylindrical, slightly or moderately curved, (8.1–) 8.6–11.3 (–11.8) × (4.3–) 4.4–5.3 (–5.4) μm (n = 21/1), L = 13.06, W = 4.99, Q = 2.00.

##### Remarks.

*Proterochaete stipidosa* is described here as a close relative of *P. mucedinea*. Both species occur in the neotropics and can be distinguished based on the size of basidia and basidiospores (see remarks on *P. mucedinea*). *Proterochaete stipidosa* is so far known from the type locality in Argentina. However, its distribution area could be much wider – it seems that older descriptions of *Exidiopsis mucedinea* (Wells 1961; Lowy 1971) at least partly cover this species. We studied two specimens from Mexico which could be conspecific with *P. stipidosa* (Suppl. material 1). They are, however, more warmly colored (pale buff to pale orange), thicker and deeply cracking, and possess slightly longer basidia and basidiospores than the type specimen of *P. stipidosa*. It is impossible to decide based on morphology if they represent *P. stipidosa* in a more developed condition or belong to yet another species. This problem should be properly addressed after obtaining sequenced material from Mexico.

#### Scrupulispora

Taxon classificationAnimaliaAuricularialesAuriculariaceae

Spirin & Viner gen. nov.

FC0E283D-E01C-5262-8A05-BF42BF1B87EC

862498

##### Etymology.

From ‘scrupulus’ (Lat., noun), a grain of sand, and ‘spora’; in reference to the occasionally encrusted basidiospore wall.

##### Description.

Basidiomata annual, effused, smooth, hardly visible, margin not differentiated. Hyphal structure monomitic, hyphae clamped, hyaline; basidia-bearing hyphae not differentiated. Cystidia abundant, hyaline, thin-walled, clavate or distinctly tapering. Acanthophyses occasionally present, thin-walled, bearing up to five pointed, variably arranged protuberances. Hyphidia abundant, variably branched, scattered among basidia. Basidia four-celled, broadly ellipsoid to subglobose, sessile, thin-walled, often covered by shields of hyphidia and remnants of collapsed basidia. Basidiospores with a distinct wall, smooth or minutely warted, broadly ellipsoid, more rarely subglobose or nearly lacrymoid, repetitive, occasionally germinating with 2–3 tubes, apiculus thick and blunt. On dead wood of angiosperms.

##### Generic type.

*Scrupulispora perparvula*.

This genus is introduced here as monotypic, with *S. perparvula* as the single species. It belongs to *Auriculariaceae* and is probably related to *Oliveonia* (Fig. 2). The two genera share some important morphological traits, such as ephemeral, inconspicuous basidiomata, tapering hymenial cystidia, and small basidia with rather long sterigmata. The presence of acanthophyses in *Scrupulispora* makes it similar to *Heteroacanthella*. However, all known *Heteroacanthella* species are devoid of cystidia and their basidia are one-celled, bearing one or two sterigmata (Oberwinkler et al. 1990; Roberts 1999). Moreover, the basidiospores of *Oliveonia* and *Heteroacanthella* spp. are smooth; in *S. perparvula*, their wall usually bears small, sand-like, cyanophilous, irregularly distributed deposits. We were able to observe this encrustation in both turgid and half-collapsed, empty spores. Therefore, we suppose that the presence of these deposits is more or less permanent, and it may serve as a diagnostic trait of *S. perparvula*. Among the *Auriculariales*, only a few *Basidiodendron* spp. also have ornamented basidiospores. They, however, do not belong to the *Auriculariaceae* (Spirin et al. 2021, 2025).

#### Scrupulispora perparvula

Taxon classificationAnimaliaAuricularialesAuriculariaceae

Spirin & Viner sp. nov.

12063505-2922-5C39-A1B9-2EE061C2368D

862499

Fig. 34

##### Holotype.

France • Aveyron: Millau, Dourbie, 44.09705 3.112, *Acer campestre* (fallen, partly corticated branch), 15 Nov 2022, Spirin 16358* (H, isotype – PC).

##### Etymology.

Perparvulus (Lat., adj.) – extremely tiny.

##### Description.

Basidiomata annual, effused, smooth, seen as a grayish bloom when rehydrated, inconspicuous when dry, up to 1 cm in widest dimension, 0.02–0.03 mm thick. Margin not differentiated.

Hyphal structure monomitic, hyphae clamped; subicular hyphae hyaline, with a distinct wall or slightly thick-walled, interwoven or subparallel, frequently anastomosing, often agglutinated, 1–4 μm in diam., subhymenial hyphae hyaline, thin-walled or with a distinct wall, interwoven, agglutinated, 1–3.5 μm in diam., basidia-bearing hyphae not differentiated. Cystidia abundant, hyaline, thin-walled, clavate or distinctly tapering, occasionally pleural, embedded or slightly projecting, 11–23 × 5–11 μm, with homogeneous, CB (+) contents. Acanthophyses infrequent, hyaline, thin-walled, 11–14 × 2.5–8.5 μm, bearing up to five pointed, variably arranged protuberances, up to 6 × 1–2 μm. Hyphidia abundant, variably branched, 0.8–2 μm in diam., scattered among basidia. Basidia four-celled, broadly ellipsoid to subglobose, sessile, occasionally with a distinctly narrowed, stipe-like base, (10.8–) 11.2–19.2 (–21.6) × (9.0–) 9.2–12.8 (–13.2) μm (n = 40/2), thin-walled, often covered by shields of hyphidia and remnants of collapsed basidia, sterigmata up to 8 × 2–3.5 μm. Basidiospores with a distinct wall (up to 0.3 μm), smooth or more often minutely warted, broadly ellipsoid, rarely subglobose or nearly lacrymoid, (7.1–) 7.2–11.1 (–11.2) × (5.2–) 5.8–9.2 (–10.2) μm (n = 60/2), L = 8.14–9.18, W = 6.73–7.44, Q = 1.21–1.24, apiculus thick and blunt, up to 1.5 × 2 μm, occasionally somewhat eccentric.

**Figure 34.**
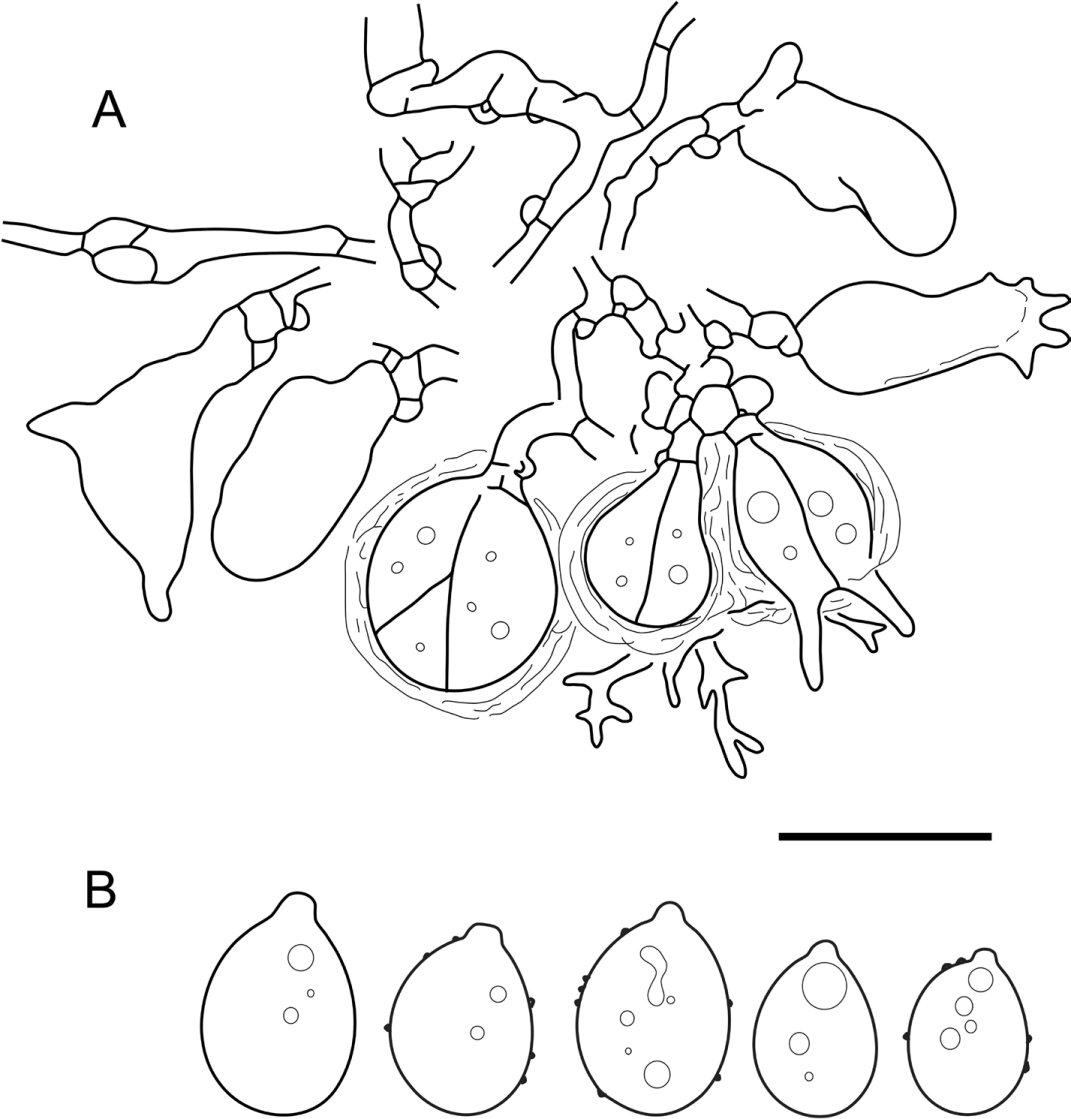
Microstructures of *Scrupulispora perparvula* (holotype): **A** hyphae and hymenial cells; **B** basidiospores. Scale bar: 10 μm.

##### Remarks.

Diagnostic features of *S. perparvula* differentiating it from the similar representatives of the *Auriculariaceae* are discussed above. We introduce this species based on two collections from Austria and France. An unnamed environmental ITS sequence JN858124 (GenBank) belongs to *S. perparvula*. It was obtained from cork samples of *Quercus suber* in Portugal (Barreto et al. 2012). This suggests that *S. perparvula* may be widely distributed in the southern part of Europe.

#### Tegmenticium

Taxon classificationAnimaliaAuricularialesAuriculariaceae

Spirin & Viner gen. nov.

305E479C-B822-5585-B0B9-4FAD2A6B73A5

862500

##### Etymology.

From ‘tegmen’ (short version of ‘tegimen’) (Lat., noun), meaning a cover or a clothing, and *Corticium*, in reference to the crust-like basidiomata.

##### Description.

Basidiomata annual, effused, waxy-gelatinous, opaque, grayish-white. Hymenial surface smooth, covered by a grayish-white, easily removable pruina. Hyphal structure monomitic; hyphae clamped, hyaline or yellowish, agglutinated, basidia-bearing hyphae poorly differentiated. Crystals normally absent. Cystidia present, thin-walled, hyaline, usually clearly tapering. Hyphidia present, with a well-differentiated stem and richly branched, non-encrusted apical outgrowths, forming a continuous layer. Basidia four-celled, ellipsoid-ovoid to subglobose, sessile, thin-walled, embedded, occasionally with a reduced stalk-like base. Basidiospores thin-walled, broadly to narrowly cylindrical, curved. On dead wood of angiosperms.

##### Generic type.

*Sebacina peritricha* Bourdot & Galzin.

This genus is introduced here for a single species, *Exidiopsis (Sebacina) peritricha*, which is currently synonymized with *E. effusa*. Our data (Figs 1, 2) show that these species should be treated separately and that they are not closely related. *Tegmenticium peritrichum* is different from other European species formerly treated under *Exidiopsis* due to its well-developed cystidia, as well as its rather small basidia and basidiospores. Its mature basidiomata are clearly gelatinized and covered by a whitish-grayish, easily removable deposit. Thus, they can be mistaken for *E. effusa*. The latter species, however, lacks cystidia and has larger basidia and, in most specimens, longer basidiospores. Moreover, it possesses spine-like outgrowths on its hymenial surface, although they can be detected only in fresh or rehydrated specimens. Differences of *T. peritrichum* from the outwardly similar *Exidia repleta* and *E. saturata* are discussed above, under *E. repleta*. *Proterochaete abstrusa* occasionally bears cystidia which are similar to those of *T. peritrichum*. However, *P. abstrusa* can be easily recognized due to its slenderer basidiomata, larger basidiospores and basidia, as well as thicker sterigmata. See further notes under *Ulocolla corticioides*.

#### Tegmenticium peritrichum

Taxon classificationAnimaliaAuricularialesAuriculariaceae

(Bourdot & Galzin) Spirin, Viner & Rivoire comb. nov.

E11E6F85-3B71-5E79-883B-F034A6738C55

862501

Figs 33C, 35, 36A

≡ Sebacina peritricha Bourdot & Galzin, Bulletin de la Société Mycologique de France 25: 26, 1909. Lectotype (selected here, MBT 10031461). France. Aveyron: Millau, Massebiau, Pyrus sp., Nov 1907, Galzin 2278 (herb. Bourdot 5159) (PC, studied).
= Exidiopsis peritricha (Bourdot & Galzin) Sacc. & Trotter, Sylloge Fungorum 21: 452, 1912.
= Exidiopsis gypsea K. Wells & Raitv., Mycologia 69: 995, 1977. Holotype. Estonia. Ida-Virumaa: Illuka, Puhatu Nat. Res., Ulmus glabra (fallen twig), 4 Oct 1967, Parmasto (TAAM 016907*, studied).

##### Description.

Basidiomata annual, first waxy-gelatinous, adpressed-orbicular, up to 1 cm diam., then fusing together, crust-like, gelatinous, up to 20 cm in the longest dimension, 0.1–0.2 mm thick. Hymenial surface smooth or indistinctly warted, entirely covered by a grayish-white, easily removable pruina, often fading to almost white in herbarium, occasionally with scattered brownish flecks, in older basidiomata often cracking and showing a darker (amber-colored to reddish-brown) inner layer. Margin compact, sharply delimited, sometimes slightly elevated, in some specimens adnate, pubescent, nearly white or grayish, up to 0.5 mm wide.

**Figure 35.**
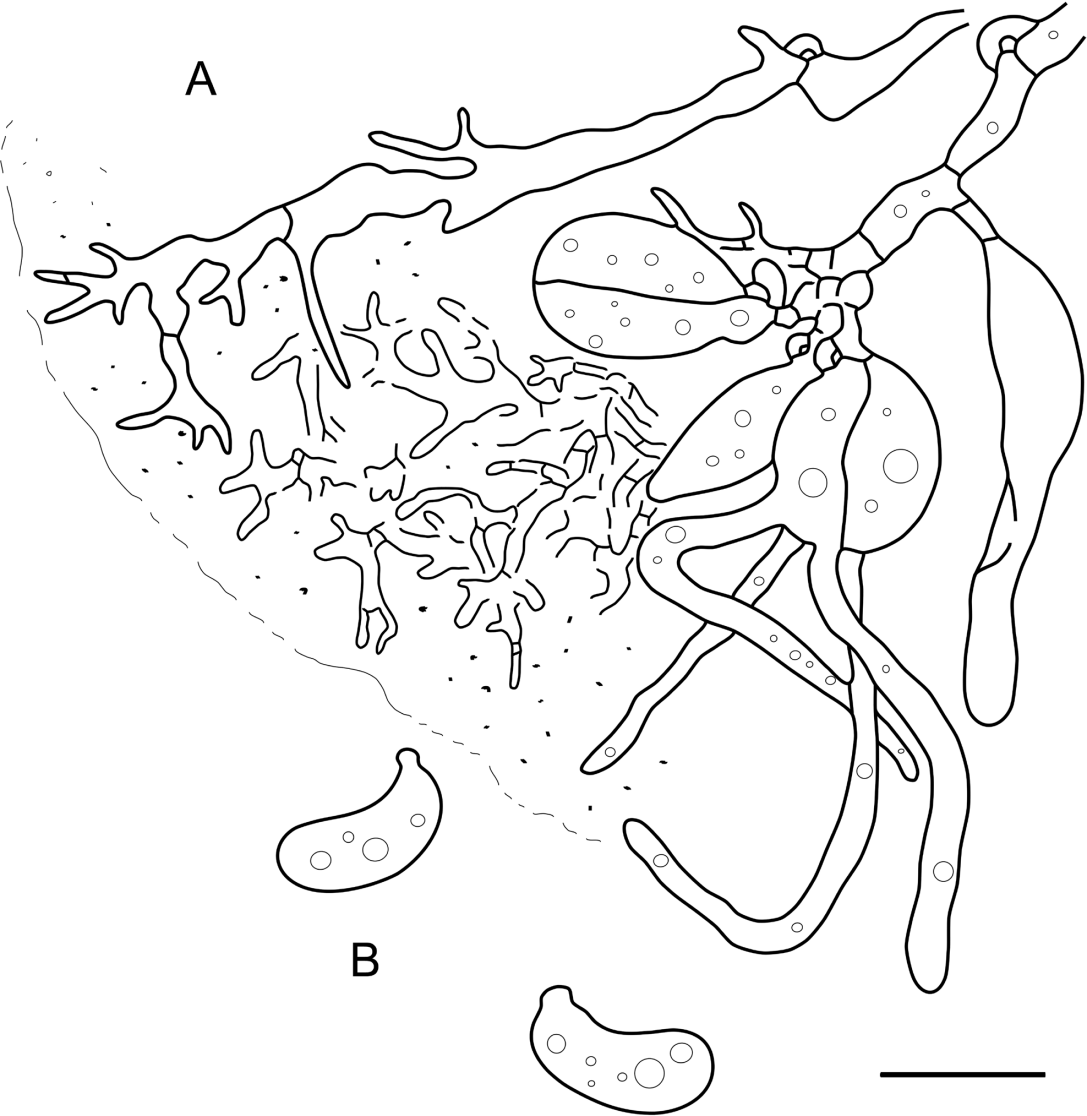
Microstructures of *Tegmenticium peritrichum* (Spirin 18537): **A** hyphae and hymenial cells; **B** basidiospores. Scale bar: 10 μm.

Hyphal structure monomitic, hyphae clamped; subicular hyphae hyaline, thin- to slightly thick-walled, interwoven or subparallel, 2–4 μm in diam., occasionally irregularly inflated (up to 5 μm in diam.), subhymenial hyphae hyaline or yellowish, thin-walled or with a distinct wall, ascending or interwoven, some twisted, agglutinated, 1.5–3.5 μm in diam., rarely irregularly inflated (up to 5 μm in diam.), basidia-bearing hyphae as a rule poorly differentiated, thin-walled, 2–3 μm in diam., often showing a zigzag-like branching pattern. Crystals as a rule absent; if present, then very rare, scattered, varying from small angular to large rhomboid, up to 15 μm in the longest dimension. Cystidia hyaline, thin-walled, abundant or infrequent, usually clearly tapering, some sinuous, rarely bifurcate, 16–70 × 3.5–13 μm, in senescent hymenium occasionally with yellowish-brownish contents. Hyphidia hyaline, more rarely with a yellowish-brownish, CB (+) contents, richly branched, with a thin-walled, subfusiform or hyphoid, often sinuous stem, up to 50 × 3–9 μm, apical branches not encrusted, 1–2 (2.5) μm in diam., often agglutinated, covering basidia and forming a continuous layer up to 30 μm thick. Probasidia clavate or tapering, 11–16 × 6–9 μm. Basidia (two-) four-celled, ellipsoid-ovoid to subglobose, sessile, (11.5–) 12–18 (–18.5) × (8.1–) 8.3–12.6 (–12.7) μm (n = 127/12), thin-walled, sometimes obliquely septate, partly agglutinated, embedded, occasionally with a short stalk-like base, up to 5 × 3 μm, sterigmata up to 45 × 2–2.5 μm. Basidiospores thin-walled, broadly to narrowly cylindrical, often distinctly curved, (8.3–) 8.9–15.1 (–16.1) × (3.6–) 3.8–6.5 (–7.2) μm (n = 457/15), L = 10.44–12.88, W = 4.31–5.74, Q = 2.08–2.76.

**Figure 36.**
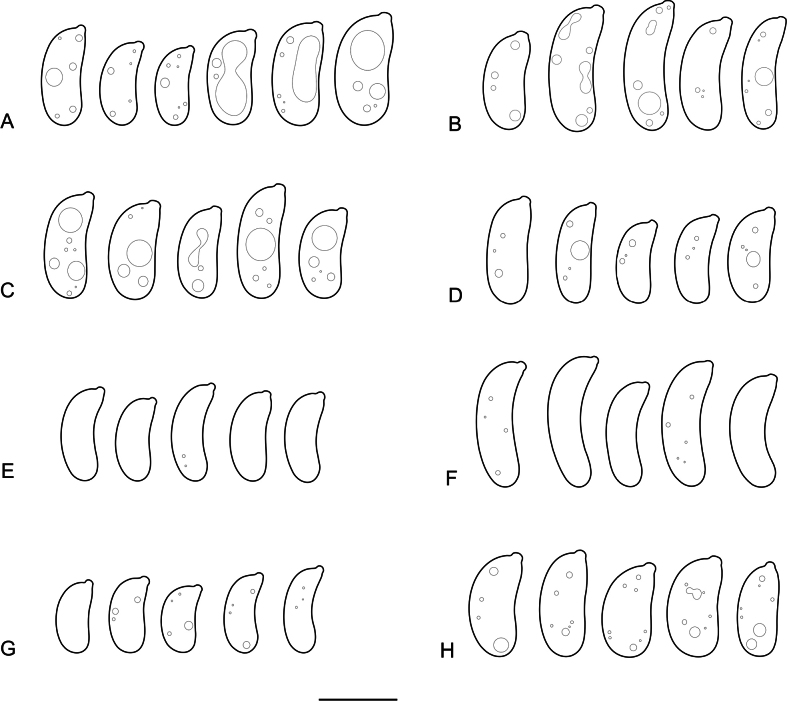
Basidiospores: **A***Tegmenticium peritrichum* (three left – lectotype, three right – holotype of *Exidiopsis gypsea*); **B***Ulocolla corticioides* (lectotype); **C***U. grisea* (Spirin 15604); **D***U. orbiculata* (holotype); **E***U. pinicola* (Spirin 8916); **F***U. saccharina* (Spirin 13748); **G***U. subrepanda* (Spirin 16132); **H***Exidia benieri* (holotype). Scale bar: 10 μm.

##### Remarks.

This species was introduced by Bourdot and Galzin (1909) as *Sebacina peritricha*. In their later work (Bourdot and Galzin 1927), they treated it as a subspecies of *Sebacina uvida* (Fr.) Bourdot & Galzin *sensu* auct. (= *Exidiopsis effusa* in the current sense; see Larsson et al. (2025) for further comments on the identity of *Thelephora uvida* Fr.). Wells (1961) listed *S. peritricha* among the synonyms of his widely defined *E. grisea*. Subsequently, it was included in the synonymy of *E. effusa* (Wells and Raitviir 1977). Fourteen specimens labelled as *S. peritricha* exist in herbarium of Bourdot (PC). Of them, eleven specimens are evidently conspecific, two (*Maire 729* and *1202*) are in poor condition, and one more (*Galzin 6987*) is a homobasidiomycete, most likely a species of *Dendrothele* s. lato. Four specimens were collected prior to the publication of *S. peritricha* and we select the best-preserved collection (*Galzin 2278*) as the lectotype of this species. *Exidiopsis gypsea* described from Estonia (Wells and Raitviir 1977) is conspecific with *S. peritricha*. ITS sequences obtained from the type specimen of *E. gypsea* are available in UNITE (UDB07674230 and UDB07674281).

*Tegmenticium peritrichum* is widely distributed in temperate – hemiboreal forests of Europe although seemingly rare everywhere. It inhabits dead, hanging or just fallen, often still corticated branches of many species of deciduous trees and shrubs.

#### Ulocolla

Taxon classificationAnimaliaTremellalesExidiaceae

Bref., Untersuchungen aus dem Gesammtgebiete der Mykologie 7: 95, 1888

A568E1E5-4F8E-5853-9A14-FB97E39BDC5A

= Sclerotrema Spirin & Malysheva, Fungal Biology 121: 712, 2017. Generic type. Exidiopsis griseobrunnea K. Wells & Raitviir, Eesti NSV Teaduste Akadeemia Toimetised 15: 206, 1966.

##### Description.

Basidiomata annual, gelatinous, adpressed-orbicular or cerebriform, or resupinate, corticioid or stereoid, arid or only slightly gelatinized, grayish or brownish to dark brown. Abhymenial surface smooth; hymenial surface smooth or variably folded, spine-like projections absent in most species. Crystals occasionally present, angular, sometimes in large concretions. Hyphal structure monomitic; hyphae clamped, hyaline or yellowish, not encrusted, basidia-bearing hyphae variably differentiated. Cystidia absent in all but one stereoid species; hyphidia present, with thickened walls, forming a continuous layer, in mature basidiomata of most species often cemented by yellowish-brownish amorphous substance. Basidia four-celled, longitudinally, sometimes obliquely septate, ellipsoid-ovoid, sterigmata slender. Basidiospores hyaline, thin-walled, broadly cylindrical to allantoid, usually distinctly curved. On dead wood of angiosperms or conifers.

##### Generic type.

*Exidia saccharina* Fr. (selected by Clements and Shear 1931: 342).

The genus *Ulocolla* was described by Brefeld (1888) for two species, *U. saccharina* and *U. foliacea*. The first species was explicitly based on *E. saccharina* by Fries, while the second one was often considered as a recombination of *Tremella foliacea* Pers. (currently *Phaeotremella foliacea* (Pers.) Wedin et al., *Tremellales*). Brefeld’s description of *U. foliacea* clearly refers to *U. saccharina* with more distinctly lobate, foliaceous basidiomata and not to *P. foliacea*. However, his reference to Persoon’s *T. foliacea* in the beginning of the *Ulocolla* chapter of his book (Brefeld 1888: 95) makes it, formally, a new combination based on Persoon’s species. Clements and Shear (1931) selected *E. saccharina* as the generic type of *Ulocolla*.

As reintroduced here, *Ulocolla* encompasses seven species with rather divergent macroscopic traits. Three of them (*U. pinicola*, *U. saccharina*, and *U. subrepanda*) were classified in *Exidia*, two (*U. grisea* and *U. griseobrunnea*) in *Exidiopsis*, and one more (*U. corticioides*) was considered merely a variety of the broadly defined *Sebacina calcea* (as var. corticioides). These species groups have never been discussed in connection to each other before or recognized as belonging to a joint monophyletic lineage. Our data firmly place them together (Figs 1, 2).

Despite their various macroscopic habits, *Ulocolla* spp. share several important anatomical traits which allow morphological recognition of this genus. First, basidiomata of most species show more or less pronounced brown pigmentation caused by yellowish or brownish cytoplasm of hyphae and hymenial cells and, in most cases, by the presence of amorphous yellowish-brownish exudates cementing apical branches of hyphidia and sometimes spreading among hymenial cells. Second, *Ulocolla* spp. have much more irregularly inflated hyphae than any other taxa treated in this study (except for *Exidia repleta*, *E. saturata*, and *Exidiopsis* s. str. spp.), which often show rather sharp transitions between normally looking, narrow and strongly widened, inflated hyphal segments. All *Ulocolla* spp. have ellipsoid-ovoid, often obliquely septate basidia sometimes having a tapering, stalk-like base and bearing slender sterigmata.

Four exidioid *Ulocolla* species (*U. orbiculata*, *U. pinicola*, *U. saccharina*, and *U. subrepanda*) differ from the similar species of *Exidia* s. str. by the lack of granular encrustation on their hyphae and hyphidia. Moreover, they have hyphidia with variably thick-walled, brownish and often strongly inflated stems. No such structures have been observed in the brown-colored *Exidia* spp. (*E. candida* and the *E. recisa* complex). In turn, hyphidia of the superficially similar *Descidia repanda* have narrow, hyphoid, rather evenly outlined stems.

#### Ulocolla corticioides

Taxon classificationAnimaliaTremellalesExidiaceae

(Malençon) Spirin & Viner comb. nov.

641E65D5-5E51-5C6B-A560-6B53AFF4C93A

864368

Figs 33D, 36B

≡ Sebacina calcea var. corticioides Malençon, Bulletin Trimestriel de la Société Mycologique de France 70: 156, 1954. Lectotype (selected here, MBT10034108). Morocco. Fez-Meknès: Ifrane, Michlifen, Quercus ilex (decorticated wood), 11 May 1953 Malençon 2419 (MPU898044, studied).

##### Description.

Basidiomata annual, initially patch-like, small, gregarious, waxy-arid, later fusing together and becoming harder (nearly leathery), crust-like, sometimes slightly gelatinized, opaque, 0.05–0.2 mm thick, up to 10 cm in the longest dimension. Hymenial surface smooth or indistinctly warted, first grayish-white or cream-colored, smooth, with scattered, brownish flecks, then indistinctly or clearly tuberculate, sometimes cracking, wood-colored to pale ochraceous, occasionally with distinct, reddish-brown dots or flecks, in senescent basidiocarps yellowish-brown. Margin first pruinose or pubescent, adnate, white, then compact, sharply delimited, in some specimens partly detaching, concolorous with hymenial surface, up to 0.5 mm wide.

Hyphal structure monomitic, hyphae clamped; subicular hyphae hyaline, thin- to slightly thick-walled, interwoven or subparallel, anastomosing, occasionally in bundles, in older basidiocarps agglutinated, 2–4 μm in diam., occasionally distinctly inflated (up to 5–7 μm in diam.), subhymenial hyphae hyaline, grayish or yellowish, thin-walled or with a distinct wall, predominantly interwoven, tightly agglutinated, 1.5–3 μm in diam., occasionally irregularly inflated (up to 5 μm in diam.), basidia-bearing hyphae as a rule poorly differentiated, thin-walled or with a distinct wall, 2.5–4 μm in diam., sometimes showing a zigzag-like branching pattern. Crystals absent or accidental, angular, up to 10 μm in the longest dimension., rather abundant in senescent, substerile basidiocarps (and then in large groups up to 20 μm in diam.). Cystidia absent. Hyphidia hyaline, grayish or yellowish, richly branched, with a thin- or slightly thick-walled, subfusiform, nodulose or hyphoid, often sinuous stem, up to 70 × 2–11 μm, apical branches not encrusted, 0.8–1.5 μm in diam., often agglutinated, covering basidia and forming a continuous layer up to 30 μm thick. Probasidia clavate or tapering, 11–19 × 5.5–10 μm. Basidia four-celled, ellipsoid-ovoid to broadly ellipsoid, sessile, (11–) 11.5–20.5 (–21) × (8.3–) 8.8–14.0 (–15.3) μm (n = 116/7), thin-walled or with a distinct wall, occasionally with a tapering stalk-like base, up to 6 × 4 μm, embedded, sterigmata up to 35 × 2–4 μm. Basidiospores thin-walled, narrowly to broadly cylindrical, often distinctly curved, (8.9–) 9.0–17.7 (–18.3) × (3.9–) 4.0–6.8 (–6.9) μm (n = 215/7), L = 11.64–15.23, W = 4.31–5.93, Q = 2.39–3.02.

##### Remarks.

*Ulocolla corticioides* was originally described by Malençon (1954) from Morocco as a variety of the widely interpreted *Sebacina calcea*. We studied five authentic specimens of this taxon stored in MPU and selected one of them as a lectotype. One specimen (Uotila 40874) from the locus classicus was used for DNA study. The ITS sequence of this collection is identical to those of three our specimens of which one (Viner 2025/94A) was included in the multigene phylogenies (Figs 1, 2). DNA data firmly place S. calcea var. corticioides close to *Exidia saccharina*, the generic type of *Ulocolla*. We therefore raise this variety to the species rank and combine it in *Ulocolla*.

*Ulocolla corticioides* is a southern species occurring mainly in the Mediterranean. Two historical collections from Öland, Sweden, are assigned to this species based on morphological study. We are not aware of other specimens of *U. corticioides* from temperate Europe. The species typically occurs on still standing and at least partly corticated, dead logs or dead, still attached branches of angiosperm trees (*Arbutus*, *Crataegus*, *Quercus*). In the protologue, Malençon (1954) mentioned that some basidiomata of *U. corticioides* on fallen logs of *Quercus* in Morocco reached up to 1 m long. The ITS sequence of *S. calcea*AJ427408 from Morocco (GenBank, obtained from CBS strain 463.62) belongs to *U. corticioides*.

Macroscopically, *U. corticioides* is most similar to *Tegmenticium peritrichum* described above. Senescent basidiomata of these species with brownish stains and flecks on the hymenial surface are especially similar. However, they are covered by a whitish pruina in *T. peritrichum*, which can be easily removed by the slightest touch, thus exposing a darker, gelatinous inner layer (Fig. 33C). The basidiocarp structure of *U. corticioides* is different: a somewhat gelatinized layer, if present, is adjacent to the substrate and it shows not a striking difference but a gradual transition to non-gelatinous hymenial and epihymenial layers. Moreover, *U. corticioides* has on average longer basidiospores than *T. peritrichum* (although their dimensions partly overlap) and it lacks cystidia.

In the Mediterranean, *U. corticioides* may occur in the same habitats with *Leiostroma incanum*. The species show a certain morphological similarity to each other, especially in the younger stages. *Ulocolla corticioides* can be separated from *L. incanum* due to its tightly arranged, partly agglutinated, not encrusted hyphae and hyphidia, as well as poorly differentiated basidia-bearing hyphae.

#### Ulocolla grisea

Taxon classificationAnimaliaTremellalesExidiaceae

(Bres.) Spirin comb. nov.

DD2F0ACF-D4C1-59B9-996C-93C5E507B3D6

862506

Figs 36C, 37

≡ *Sebacina grisea* Bres., Annales Mycologici 6: 45, 1908.
= Thelephora grisea Pers., Mycologia Europaea 1: 149, 1822. Neotype (selected by Wells 1961: 343). France. [no locality indicated], Abies alba, 24 Apr 1900, Strasser (S F350416, studied).
= Exidiopsis grisea (Bres.) Bourdot & Maire, Bulletin de la Société Mycologique de France 36: 71, 1920.

##### Description.

Basidiomata annual, effused, up to 10 cm in the longest dimension, rarely larger, gelatinous, semitranslucent, up to 0.5 mm thick. Hymenial surface grayish, smooth or indistinctly tuberculate in fresh condition, bluish-gray and shining in herbarium. Margin sharply delimited, adnate.

Hyphal structure monomitic, hyphae clamped; subicular hyphae hyaline or yellowish, thin- or slightly thick-walled, subparallel or interwoven, anastomosing, agglutinated, 1.5–4 μm in diam., subhymenial hyphae hyaline or yellowish, thin-walled, predominantly ascending, 1.5–4 μm in diam., occasionally inflated and then up to 5 μm in diam., basidia-bearing hyphae not differentiated. Crystals occasionally present, angular, scattered among hyphae, sometimes producing large concretions up to 25 μm in diam. Cystidia absent. Hyphidia thin-walled, with a thin-walled, hyaline, hyphoid or nodulose stem, 17–35 × 3–5 μm, and abundant, richly branched, hyaline apical outgrowths, 1–1.5 μm in diam. at the very apex, in older basidiomata partly embedded in gelatinous matrix and producing a continuous layer up to 30 μm thick. Probasidia usually tapering, more rarely narrowly clavate, 10–20 × 5–6 μm. Basidia four-celled, ellipsoid-ovoid to subglobose, occasionally with a distinctly narrowed, stalk-like base, (10–) 10.5–15.5 (–16) × (8.1–) 8.3–12.2 (–12.8) μm (n = 50/4), thin-walled, embedded, sterigmata up to 20 × 1.5–2 μm. Basidiospores thin-walled, broadly to narrowly cylindrical, distinctly curved, (9.0–) 9.1–14.3 (–15.1) × (3.9–) 4.0–5.7 (–5.8) μm (n = 180/6), L = 10.69–12.94, W = 4.60–5.06, Q = 2.23–2.62.

**Figure 37.**
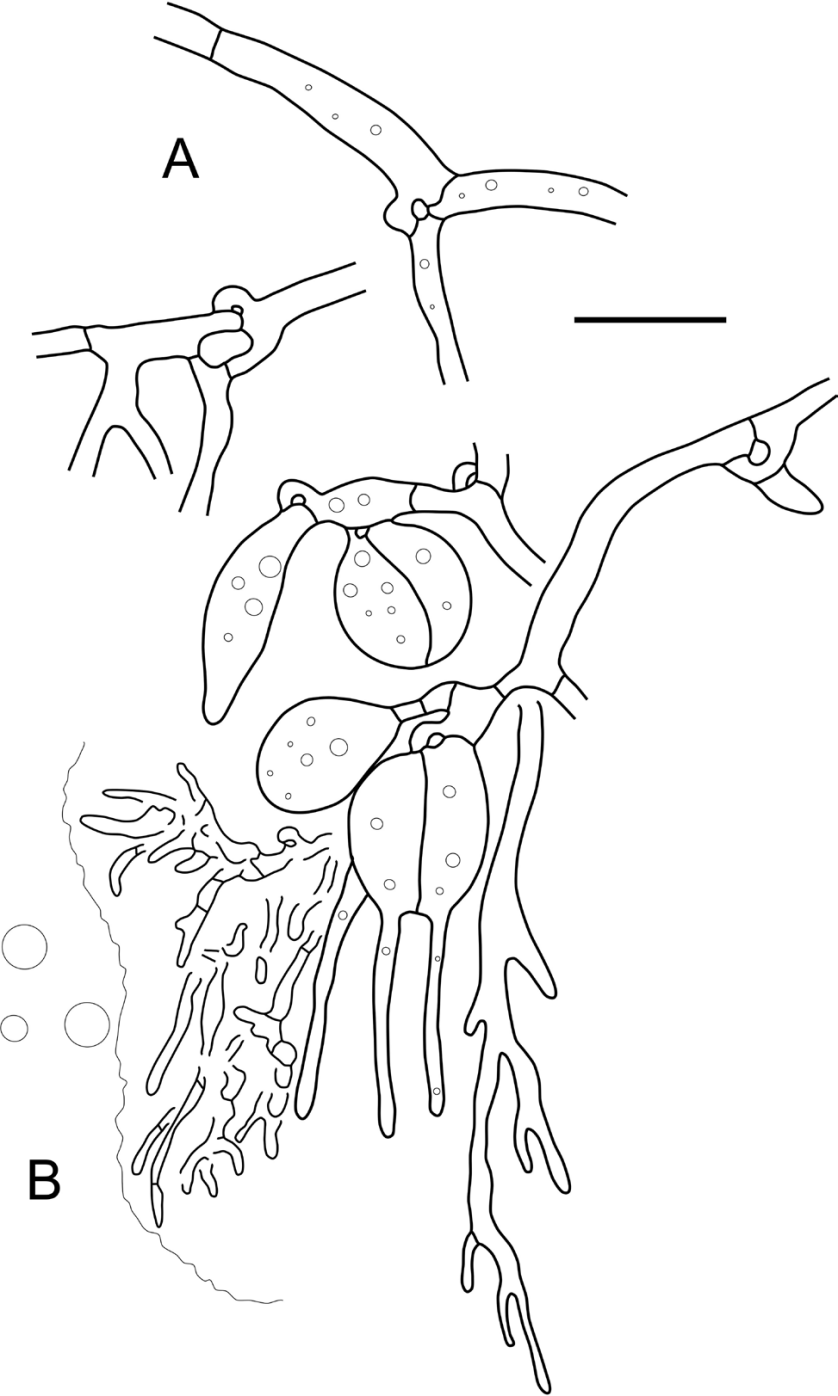
Microstructures of *Ulocolla grisea* (Spirin 15604): **A** subicular hyphae; **B** subhymenial hyphae and hymenial cells. Scale bar: 10 μm.

##### Remarks.

This species was first introduced by Persoon (1822) as *Thelephora grisea*. Although only macroscopic traits were mentioned in the protologue, there is a little doubt about the species Persoon dealt with – there are no other effused, semitranslucent, bluish-grayish, rather thick jelly fungi on coniferous wood in Europe. Unfortunately, Persoon’s *T. grisea* turned out to be a homonym of *T. grisea* Schwein.; the latter name was sanctioned by Fries (1828) and this made Persoon’s species name unavailable. It was resurrected by Bresadola (1908) who transferred *T. grisea* Pers. to *Sebacina* and thus made it available for future recombinations in other genera. Wells (1961) redescribed *Exidiopsis grisea* with a long list of synonyms which included, inter alia, *E. effusa* and *Sebacina plumbescens*. His view on the identity of *E. grisea* was contested by Donk (1966) and Reid (1970). Christiansen (1959), Oberwinkler (1963) and Wells and Raitviir (1977) reintroduced the species in the sense accepted here. No authentic material from Persoon is extant; Wells (1961) selected one of Bresadola’s specimens from Stockholm as a neotype of *T. grisea*.

*Ulocolla grisea* is closely related to *U. orbiculata* from Far East Asia. Their differences are listed under the latter species. *Exidiopsis effusa* is macroscopically somewhat similar to *U. grisea*. However, *U. grisea* is restricted to wood remnants of conifers (especially *Abies* and *Pinus*) while *E. effusa* occurs exclusively on angiosperm hosts. The hymenophore of *E. effusa* often bears sharp projections and covered by whitish, easily removable pruina. In contrast, basidiomata of *U. grisea* are completely smooth or indistinctly tuberculate and possess no deposits on hymenial surface. Moreover, *E. effusa* has wider hyphae than *U. grisea*, which are often quite irregularly inflated, and its basidia are larger and basidiospores are on average longer than in the latter species. *Protohydnum livescens* is another effused conifer-dwelling species with rather thick gelationous basidiomata occurring in Europe. It differs from *U. grisea* by the pronounced reddish or brownish tints of dried specimens, as well as in having pedunculate basidia and wider basidiospores (see description in Spirin et al. 2025).

#### Ulocolla griseobrunnea

Taxon classificationAnimaliaTremellalesExidiaceae

(K. Wells & Raitviir) Spirin & Malysheva comb. nov.

8699BED1-430C-53ED-9391-4D76A9126576

862502

≡ Exidiopsis griseobrunnea K. Wells & Raitviir, Eesti NSV Teaduste Akadeemia Toimetised 15: 206, 1966. Holotype. Russia. Tyumen Reg.: Yamal-Nenets Autonomous Dist., Krasnoselkup, Alnus fruticosa, 31 Jul 1964 Parmasto (TAAM 17048*, studied).
= Sclerotrema griseobrunneum (K. Wells & Raitviir) Spirin & Malysheva, Fungal Biology 121: 713, 2017.

##### Note.

For a description and photographs, see Malysheva and Spirin (2017). *Ulocolla griseobrunnea* differs from other *Ulocolla* spp. due to its effused, stereoid basidiomata and the presence of hymenial cystidia. The species was reported by Torkelsen (1972) from Norway. However, her specimens belong to *Exidia plumbescens*. So far, there have been no verified records of *U. griseobrunnea* from Europe.

#### Ulocolla orbiculata

Taxon classificationAnimaliaTremellalesExidiaceae

Spirin & Viner sp. nov.

E5BB709C-CCDA-5CE1-AAF1-97E2D05FB308

862507

Figs 33E, 36D

##### Holotype.

Russia • Khabarovsk Reg.: Verkhnebureinskii Dist., Hegdy, 50.49582 133.30898, *Abies nephrolepis* (hanging dead branch), 18 Aug 2014, Spirin 7487* (H).

##### Etymology.

Orbiculatus (Lat., adj.) – orbiculate.

##### Description.

Basidiomata annual, at first frustulate, later appressed-orbicular, up to 0.5 cm in diam., gradually fusing together and forming compound effused fructifications, up to 3 cm in the longest dimension, gelatinous, semitranslucent, up to 0.5 mm thick. Hymenial surface smooth, grayish-brown in fresh condition, brown or dark-brown and vernicose when dry. Margin sharply delimited and often slightly elevated, in mature basidiomata partly detaching.

Hyphal structure monomitic, hyphae clamped; subicular hyphae hyaline, variably thick-walled, subparallel, anastomosing, tightly agglutinated, hardly discernible, 1.5–3 μm in diam., subhymenial hyphae hyaline or yellowish, thin-walled or with a distinct wall, ascending or interwoven, frequently anastomosing, 1–3.5 μm in diam., occasionally inflated and then up to 5 μm in diam., basidia-bearing hyphae not differentiated. Crystals occasionally present, angular, scattered among hyphae, sometimes producing large concretions up to 25 μm in diam. Cystidia absent. Hyphidia thin-walled, with a thin-walled, hyaline, hyphoid stem, 1.5–3.5 μm in diam., and abundant, richly branched, hyaline or brownish apical outgrowths, 0.5–1.5 μm in diam. at the very apex, often forming a continuous layer up to 40 μm thick. Probasidia slightly tapering to nearly bullet-shaped, 9–19 × 3–8 μm. Basidia four-celled, ellipsoid-ovoid to subglobose, occasionally with a distinctly narrowed, stalk-like base, (10–) 11–15 (–15.5) × (7.8–) 7.9–10.1 (–11.2) μm (n = 20/1), thin-walled, embedded, sterigmata up to 20 × 2 μm. Basidiospores thin-walled, broadly to narrowly cylindrical, often distinctly curved, (9.8–) 10.0–14.3 (–15.7) × (3.9–) 4.0–5.2 (–5.3) μm (n = 21/1), L = 11.97, W = 4.45, Q = 2.69.

##### Remarks.

*Ulocolla orbiculata* is described here as the sister species of *U. grisea*. These species are phylogenetically closely related and anatomically very similar. However, their basidiomata differ considerably: *U. grisea* has widely effused (corticioid), adnate basidiomata while *U. orbiculata* possesses button-shaped basidiomata with an elevated margin reminiscent of many *Exidia* spp. which later fuse together in compound, partly detaching fruitbodies. *Ulocolla orbiculata* is so far known from two localities in Khabarovsk Region, Far East Russia. It was found on dead but still attached, corticated branches of *Abies*.

#### Ulocolla pinicola

Taxon classificationAnimaliaTremellalesExidiaceae

(Peck) Spirin comb. nov.

ED3282CA-E3D0-5C3A-A7E4-A05C445AE0F1

862503

Fig. 36E

≡ Tremella pinicola Peck, Annual Report on the New York State Museum of Natural History 39: 44, 1886. Holotype. USA. New York: Saratoga Co., Day, coniferous branches, ‘July’, Peck (NYS).
= Exidia pinicola (Peck) Coker, Journal of the Elisha Mitchell Scientific Society 36: 150, 1920.

##### Description.

Basidiomata annual, at first cushion-shaped or adpressed-orbicular, narrowly or broadly attached, later cerebriform or almost foliaceous, up to 5 cm in diam. and 2 cm thick, gelatinous. Hymenial surface at first nearly smooth or uneven, then distinctly folded, amber-yellow to yellowish-brown, later reddish-brown, in dry condition brownish-black. Abhymenial surface smooth, concolourous with hymenial surface. Margin free, concolourous with hymenial surface, entire, even or indistinctly lobate.

Hyphal structure monomitic, hyphae clamped; context hyphae hyaline, thin- to distinctly thick-walled, interwoven or more rarely subparallel, anastomosing, 1–6.5 μm in diam., subhymenial hyphae hyaline to yellowish, thin- to distinctly thick-walled, predominantly ascending, 1.5–4 μm in diam., occasionally inflated up to 6–7 μm in diam., basidia-bearing hyphae 2–4 μm in diam. Cystidia absent. Hyphidia with a slightly thick-walled, hyaline or brownish, hyphoid or nodulose stem, 2–5 μm in diam., and hyaline or yellowish-brownish apical branches, 1–1.5 μm in diam., forming a continuous layer up to 40 μm thick. Probasidia clavate or tapering, 13–19 × 4–8 μm. Basidia thin-walled, four-celled, ellipsoid-ovoid, occasionally with a distinctly narrowed, stalk-like base, (13–) 13.5–19 (–21.5) × (8.4–) 8.5–10.3 (–11.0) μm (n = 20/2), sterigmata up to 30 × 1.2–1.5 μm. Basidiospores narrowly cylindrical to cylindrical, distinctly curved, (8.7–) 9.2–13.2 (–14.2) × (3.3–) 3.7–5.1 (–5.2) μm (n = 90/3), L = 10.46–11.67, W = 4.00–4.27, Q = 2.62–2.82.

##### Remarks.

*Ulocolla pinicola* is the North American relative of *U. saccharina* and *U. subrepanda* (Fig. 12). It inhabits various coniferous trees, mainly in the cold temperate and boreal regions. Morphologically, it is indiscernible from *U. subrepanda* from Eurasia and can be separated from the latter species based on DNA sequences and geographic distribution. In turn, *U. saccharina* from Europe possesses larger basidia and basidiospores than *U. pinicola*, and it is so far found only on *Pinus* spp.

This species was originally described from New York as *Tremella pinicola* (Peck 1886). Coker (1920) restudied the type and combined it in *Exidia*. He further concluded that *E. pinicola* is an older name for *E. umbrinella* from Europe (see description above). However, the spore measurements given by Coker (3.7–4 × 9.5–11.1 μm) rule out *E. umbrinella* and, in combination with macroscopic features, clearly point towards a member of the *U. saccharina* group.

#### Ulocolla saccharina

Taxon classificationAnimaliaTremellalesExidiaceae

(Fr.) Bref., Untersuchungen aus dem Gesammtgebiete der Mykologie 7: 95, 1888

36520E82-0652-59A6-8431-793084DD23ED

Figs 33F, 36F, 38

≡ Exidia saccharina Fr., Systema Mycologicum 2: 225, 1822. Neotype (selected here, MBT 10031462). Finland. Uusimaa: Helsinki, Koskela, Pinus sylvestris, 14 Jun 2014, Miettinen 18284.1* (H).
= Tremella spiculosa var. saccharina Alb. & Schwein., Conspectus Fungorum in Lusatiae Superioris: 302, 1805.
= Exidia subsaccharina F. Wu, Rivoire, Tohtirjap & Y.C. Dai, Frontiers in Microbiology 13 (1080290): 7, 2023. Holotype. France. Rhône: Chaussan, Pinus sylvestris, 10 Aug 2008, Dai 22187* (BJFC 36778).

##### Description.

Basidiomata annual, at first cushion-shaped or adpressed-orbicular, narrowly or broadly attached, later cerebriform, up to 5 cm in diam., finally often fusing together and reaching 20 cm in the longest dimension, up to 2 cm thick, gelatinous. Hymenial surface at first nearly smooth or uneven, then distinctly folded, occasionally covered by rather regularly arranged spine-like projections up to 0.2 mm long, amber-yellow to yellowish-brown, later reddish-brown, in dry condition brownish-black. Abhymenial surface smooth, concolourous with hymenial surface. Margin free, concolourous with hymenial surface, entire, even or indistinctly lobate.

**Figure 38.**
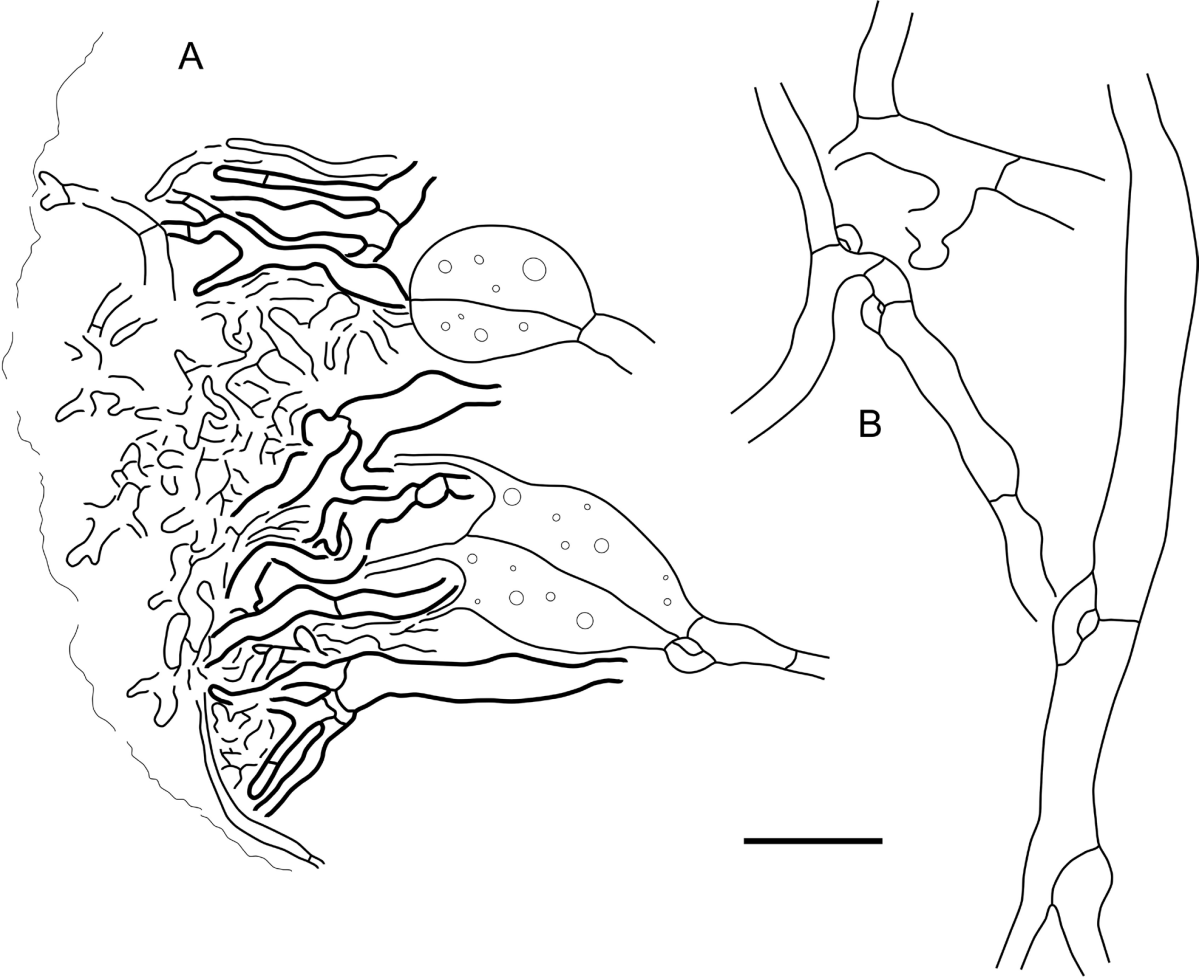
Microstructures of *Ulocolla saccharina* (Spirin 13748): **A** subhymenial hyphae and hymenial cells; **B** context hyphae. Scale bar: 10 μm.

Hyphal structure monomitic, hyphae clamped; context hyphae hyaline, thin- to slightly or rarely distinctly thick-walled, predominantly interwoven, spaced, anastomosing, 1–6.5 μm in diam., subhymenial hyphae hyaline to yellowish, thin- to slightly thick-walled, ascending or interwoven, 2–5 (7) μm in diam., basidia-bearing hyphae 2–6 μm in diam. Cystidia absent. Hyphidia with a thin- or slightly thick-walled, yellowish or brownish, hyphoid or nodulose stem, 2–9 μm in diam., and hyaline or yellowish-brownish apical branches, 1–2 μm in diam., forming a continuous layer up to 50 μm thick. Probasidia broadly to narrowly clavate, 13–20 × 4–8.5 μm. Basidia thin-walled, four-celled, ellipsoid-ovoid, some with a distinctly narrowed, stalk-like base, (14.5–) 15–22.5 (–23) × (9.1–) 9.2–13.0 (–13.2) μm (n = 60/6), sterigmata up to 30 × 2–2.5 μm. Basidiospores narrowly cylindrical to allantoid, more rarely cylindrical, distinctly curved, (10.1–) 10.2–17.7 (–18.1) × (3.7–) 3.8–5.8 (–6.1) μm (n = 180/6), L = 13.49–15.25, W = 4.24–5.00, Q = 2.98–3.24.

##### Remarks.

*Ulocolla saccharina* is distributed in temperate and hemiboreal forests of Europe and seemingly restricted to wood remains of *Pinus* spp. Substantial basidiomata with distinctly a plicate or reticulate hymenophore often bearing papillose projections, as well as large basidia and long basidiospores help to separate it from the closely related *U. subrepanda*. The latter species has a more northern distribution and occurs on a larger set of coniferous hosts (see below).

This species was introduced by Fries (1822) as *E. saccharina* although his name and, at some important parts, description referred to that of ‘*Tremella spiculosa* γγ *saccharina*’ by Albertini and Schweinitz (1805). No authentic material is extant from the latter authors or Fries. Albertini and Schweinitz (ibid.) described T. spiculosa var. saccharina as producing large, brown, plicate-undulate basidiomata on freshly cut *Pinus* logs and having a papillose hymenial surface. The latter feature was also emphasized by Fries (1822). He observed *E. saccharina* inhabiting logs of *Pinus* and *Picea* (given as *Abies*) in Sweden. From his indication of the host trees, we can suggest that Fries’ concept of *E. saccharina* was mixed, i.e. it also covered the second European species from the *U. saccharina* complex reintroduced below as *U. subrepanda*. However, the hymenial surface of *U. subrepanda* can hardly be described as papillose. Poorly developed sharp warts are present only occasionally in senescent basidiomata of the latter species and they are visible only under magnification. On the contrary, hymenial projections of *U. saccharina*, if present, are usually detectable with the naked eye. This trait allows us to suggest that the pine-dwelling collections (i.e. those ones belonging to *U. saccharina* in the present sense) were the main source of the species introduction by Fries. The color photo of the type specimen of *E. subsaccharina* published by Gruhn et al. (2024) shows typical specimen of *U. saccharina* with a papillose hymenial surface. Based on morphological traits and sequences of *E. subsaccharina*, we place it as a synonym of *U. saccharina*. See further remarks under *U. pinicola* and *U. subrepanda*.

#### Ulocolla subrepanda

Taxon classificationAnimaliaTremellalesExidiaceae

(P. Karst.) Spirin comb. nov.

6EBF40BD-0529-5109-9496-856489992C47

862504

Figs 33G, 36G

≡ Exidia albida ssp. subrepanda P. Karst., Meddelanden af Societas pro Fauna et Flora Fennica 18: 73, 1891. Lectotype (selected by Spirin et al. 2018: 7). Finland. Etelä-Häme: Tammela, Mustiala, Picea abies, 18 Nov 1890, Karsten 2143 (H6061344, studied).
= Exidia subrepanda (P. Karst.) Oudemans, Enumeratio Systematica Fungorum 1: 398, 1919.

##### Description.

Basidiomata annual, at first cushion-shaped or adpressed-orbicular, narrowly or broadly attached, later cerebriform or almost foliaceous, up to 6 cm in diam. and 2 cm thick, gelatinous. Hymenial surface at first nearly smooth or uneven, then distinctly folded, occasionally covered by poorly visible densely arranged warts, amber-yellow to yellowish-brown, later reddish-brown, in older specimens fading to pale yellow, in dry condition brownish-black. Abhymenial surface smooth, concolourous with hymenial surface. Margin free, concolourous with hymenial surface, entire, even or indistinctly lobate.

Hyphal structure monomitic, hyphae clamped; context hyphae hyaline, thin- to distinctly thick-walled (walls often strongly gelatinized), interwoven, anastomosing, 1–5 μm in diam., subhymenial hyphae hyaline to yellowish, thin- to distinctly thick-walled, ascending or interwoven, 1–5 μm in diam., basidia-bearing hyphae 2–4 μm in diam. Cystidia absent. Hyphidia with a distinctly thick-walled, brownish or brown, hyphoid or nodulose (occasionally almost moniliform) stem, 2–6 μm in diam., and yellowish-brownish apical branches, 1–2 μm in diam., forming a continuous layer up to 40 μm thick. Probasidia clavate or tapering, 14–18 × 5–7 μm. Basidia thin- or slightly thick-walled, four-celled, ellipsoid-ovoid to narrowly ovoid, some with a distinctly narrowed, stalk-like base, occasionally obliquely septate, (13–) 13.5–20 (–20.5) × (7.2–) 7.3–10.4 (–10.9) μm (n = 70/7), sterigmata up to 15 × 1.2–1.5 μm. Basidiospores narrowly cylindrical to cylindrical, distinctly curved, (8.1–) 8.2–13.3 (–14.2) × (3.2–) 3.3–5.4 (–5.7) μm (n = 240/8), L = 9.96–11.33, W = 3.91–4.85, Q = 2.35–2.81.

##### Remarks.

*Ulocolla subrepanda* is widely distributed in the cold temperate and boreal ecosystems of Eurasia where it inhabits wood (mainly freshly fallen logs and thick branches) of various coniferous trees. Morphologically and phylogenetically, it is closely related to *U. saccharina* from Europe and *U. pinicola* from North America. It differs from *U. saccharina* in having smaller basidia and basidiospores. The basidiomata of *U. subrepanda* are also usually smaller than those of *U. saccharina*, and they are devoid of easily detectable hymenial projections. We could not find any reliable morphological traits separating *U. subrepanda* and *U. pinicola*. However, these species show clear differences in ITS sequences and they are distributed in different geographic areas.

This species was first introduced as a variety of *Exidia albida* sensu auct. (Karsten 1891) and then raised to the species rank in the genus *Exidia* (Oudemans 1919). The type specimen was collected from *Picea abies*. It is completely sterile; however, the host tree and rather small basidia preclude *U. saccharina* s. str. (see above).

### Key for identification of *Exidia* s. lato species in Europe

**Table:** 

1	Basidiomata pinkish- to brick-red. Hyphae clampless	***Ditangium* spp. (*Sebacinales*) (for further information, see Malysheva et al. 2019)**
–	Basidiomata colored otherwise. Hyphae clamped or clampless	**2**
2	Basidiomata nearly black or dark gray	**3**
–	Basidiomata hyaline, whitish, yellowish, reddish-brown or brown	**11**
3	Abhymenial surface rough, hirsute or scabrose	**4**
–	Abhymenial surface smooth or minutely dotted	**5**
4	Abhymenial surface hairy. Average basidiospore length less than 16 μm	***Exidia glandulosa***
–	Abhymenial surface scabrose. Average basidiospore length over 17 μm	***Exidia truncata***
5	On conifers	**6**
–	On angiosperms	**7**
6	Hymenial surface as a rule with spine-like outgrowths. Subhymenial hyphae predominantly interwoven	***Exidia pithya***
–	Hymenial surface without spine-like projections. Subhymenial hyphae clearly ascending	***Exidia friesiana***
7	Basidia regularly reaching 25 μm long. Basidiospores 13–22 × 4–7 μm, L > 16	***Exidia zelleri***
–	All basidia less than 21 μm long. Basidiospores up to 16.5 μm long, L < 14.5	**8**
8	Basidiomata cupulate-orbicular, with a narrowed base, remaining discrete	***Exidia impressa***
–	Basidiomata first effused-orbicular, then often fusing together	**9**
9	Basidiospores narrowly cylindrical to allantoid, 10–15.5 × 3–4 μm, aguttulate. Hymenial surface always without spine-like projections	***Exidia spiculata***
–	Basidiospores narrowly to broadly cylindrical, with oil-rich contents. Hymenial projections present or absent	**10**
10	Average basidiospore width less than 4.7 μm. Hymenial projections often (but not always!) present. Widespread species on numerous hosts	***Exidia pithya***
–	Average basidiospore width over 5.4 μm. Hymenial projections always absent. On *Quercus*, rare southern species	***Exidia inornata***
11	Basidiomata hyaline or very pale colored, usually small (less than 1 cm in diam.). Basidia pedunculate	***Myxarium* spp. (for further information, see Spirin et al. 2019 and a note on *Exidia cinnamomescens* below)**
–	Basidiomata variably colored. Basidia sessile	**12**
12	Basidiomata brownish. Hyphae constantly clampess	***Globulisebacina badioumbrina* (*Sebacinales* ) (for further information, see Friebes and Spirin 2025)**
–	Hyphae clamped	**13**
13	Basidiomata cupulate-orbicular, with a narrowed base, yellowish- or reddish-brown. Abhymenial surface minutely dotted	**14**
–	Basidiomata cushion-shaped, adpressed-orbicular, turbinate or nearly foliaceous, broadly attached, variably colored. Abhymenial surface smooth	**16**
14	On conifers	***Exidia umbrinella***
–	On angiosperms	**15**
15	Basidiomata reddish-brown to dark brown. Northern or mountain species, predominantly on *Salix* spp.	***Exidia recisa***
–	Basidiomata yellowish- to ochraceous-brown. Southern species, on many angiosperm hosts but not on *Salix*	***Exidia straminea***
16	On conifers	**17**
–	On angiosperms	**18**
17	Average basidiospore length over 13 μm. Exclusively on *Pinus*. Southern species	***Ulocolla saccharina***
–	Average basidiospore length less than 11.5 μm. On *Pinus*, *Picea* and *Larix*, mostly in northern areas	***Ulocolla subrepanda***
18	Basidiomata white to cream-colored or very pale brown. Hymenial spine-like projections absent. Average basidiospore length over 14.5 μm	***Descidia thuretiana***
–	Basidiomata amber-yellow to light-brown, if whitish then with spine-like projections on hymenial surface. Average basidiospore length less than 13.5 μm	**19**
19	Exclusively on *Betula*. Hymenial spine-like projections always absent. Basidiospores narrowly cylindrical to allantoid, 10–15 × 2.8–4 μm	***Descidia repanda***
–	On various hosts. Hymenial spine-like projections occasionally present. Basidiospores narrowly to broadly cylindrical, 9–15 × 3–5 μm	***Exidia candida***

### Key for identification of *Exidiopsis* s. lato species in Europe

**Table:** 

1	Basidiomata hygroscopic, at least partly gelatinized	**2**
–	Basidiomata non-hygroscopic, arid or waxy, soft	**13**
2	On conifers	**3**
–	On angiosperms	**4**
3	Basidia pedunculate. Basidiospores 9–16 × 5–7 μm	***Protohydnum livescens***
–	Basidia sessile. Basidiospores 9–14.5 × 4–5.5 μm	***Ulocolla grisea***
4	Margin clearly elevated and partly detaching	***Descidia thuretiana* (effused forms)**
–	Margin adnate and usually gradually thinning	**5**
5	Spine-like hymenial projections present at least at some parts of the basidiome	**6**
–	Spine-like projections permanently lacking	**7**
6	Margin as a rule fimbriate, occasionally with short rhizomorphs	***Proterochaete adusta***
–	Margin sharply delimited, adnate, rhizomorphs absent	***Exidiopsis effusa***
7	Hymenial surface covered by grayish-white, easily removable pruina. Cystidia present	**8**
–	Hymenial surface without visible deposits. Cystidia absent	**10**
8	Basidiocarps corticioid. Hyphae predominantly thin-walled, rather evenly outlined	***Tegmenticium peritrichum***
–	Basidiocarps initially frustulate or adpressed- orbicular, later fusing together but at least partly remaining discernible. Hyphae thin- to slightly thick-walled, some strongly inflated	**9**
9	Basidiocarps grayish-white. Basidiospores 11–15 × 5–6 μm	***Exidia repleta***
–	Basidiocarps with bluish or violet hues. Basidiospores 10–15 × 4–5 μm	***Exidia saturata***
10	Basidiocarps opaque. Only the innermost layer of basidiocarps slightly gelatinized	***Ulocolla corticioides***
–	Basidiocarps semitranslucent or opalescent, entirely gelatinous	**11**
11	Hyphidia with a poorly differentiated, hyphoid stem, 2–4 μm in diam	***Exidia delita***
–	Hyphidia with a hyphoid or subfusiform, well-visible stem, 2–8 μm in diam.	**12**
12	Basidiomata substantial, usually several cm long and up to 1.5 mm thick. Mineral inclusions present (visible under lens). Cystidia absent	***Exidia plumbescens***
–	Basidiomata normally small, rarely exceeding 1 cm in the longest dimension, 0.05–0.1 mm thick. Mineral inclusions absent. Cystidia rarely present	***Proterochaete abstrusa***
13	Spine-like hymenial projections present. Gloeocystidia present. Crystals accidental or absent	***Eichleriella macrochaeta***
–	Hymenial surface smooth. Gloeocystidia absent	**14**
14	Basidiomata waxy, delicate. Cystidia absent. Crystals accidental or absent. Usually occurring in spring	***Exidiopsis perflua* (cf. also *E. effusa* above)**
–	Basidiomata arid. Cystidia present or absent. Crystals abundant. Throughout a year	**15**
15	Brown, cyanophilous amorphous substance present on hyphae and hymenial cells	***Proterochaete letendreana***
–	Only crystalline encrustation present	**16**
16	Hymenial surface with pinkish or reddish tints. Basidia with a distinct stalk-like base	***Heteroradulum deglubens* (effused forms with adnate margin and smooth hymenial surface)**
–	Without pinkish or reddish tints. Basidia occasionally with a reduced stalk-like base	**17**
17	Basidiomata small, not exceeding 1 cm in length, white or pale cream-colored. Cystidia present. Basidia up to 30 μm long. On tiny twigs and dead liana stems	***Adustochaete hiemalis***
–	Basidiomata larger, at least a few cm in widest dimension, pale gray to grayish-brown. Basidia up to 25 μm long	**18**
18	Average basidiospore length less than 13 μm. Exclusively on angiosperms in the Mediterranean	***Leiostroma incanum* (cf. also *U. corticioides* above)**
–	Average basidiospore length over 13 μm. Almost exclusively on conifers in temperate and boreal forests	***Leiostroma farinellum***

### Excluded and insufficiently known taxa

#### Exidia benieri

Taxon classificationAnimaliaAuricularialesAuriculariaceae

Pat., Journal de Botanique 8: 221, 1894

E5F049CE-585E-561B-8EF7-27966D5687BB

Fig. 36H

##### Holotype.

Tunisia • El Fedja, *Laurus nobilis*, Oct 1893, Bénier (FH00784211, studied).

*Exidia benieri* was described from Tunisia (Patouillard 1894). No modern records of this species are known to us. Morphologically, it is a member of the *E. pithya* complex. Due to its broadly cylindrical basidiospores, *E. benieri* is strongly reminiscent of *E. inornata* (see description above). The spores are, however, shorter in *E. benieri* than in *E. inornata*, (9.1–) 10.5–12.8 (–12.9) × (4.8–) 4.9–6.5 (–6.6) μm (n = 30/1), L = 11.64, W = 5.53, Q = 2.12. Moreover, it occasionally possesses blunt or spine-like projections on the hymenial surface, similar to those of *E. pithya* and most other *Exidia* spp. which are lacking in *E. inornata*. We therefore prefer to keep *E. benieri* and *E. inornata* separate, awaiting newly collected and sequenced material from North Africa.

#### Exidia cinnamomescens

Taxon classificationAnimaliaAuricularialesAuriculariaceae

Raitv., Tartu Riikliku Ülikooli Toimetised 136: 208, 1963

0A984B4F-7195-52E9-81A3-78F8EE2D6D79

= Myxarium cinnamomescens (Raitviir) Raitviir, Plants and Animals of the Far East: 113, 1971.

##### Holotype.

Russia • Komi Rep.: Syktyvdinsky Dist., Graddor, *Populus tremula* (fallen log), 8 Aug 1957, Põldmaa (TAAM 5995*, studied).

##### Note.

This species was first described as a member of *Exidia* (Raitviir 1963) and later combined in *Myxarium* (Raitviir 1971). It was accepted as *M. cinnamomescens* in our recent publications (Spirin et al. 2018, 2019, 2025). The species name was associated with a sister species of *M. populinum* (P. Karst.) Spirin & Malysheva, inhabiting various angiosperm hosts in temperate and boreal forests of Eurasia. The two species have been separated almost exclusively based on their spore size (mainly the spore width). The basidiospore dimensions from the type specimen of *E. cinnamomescens* fall within the same range with other specimens so labelled, (9.3–) 10.2–13.4 (–13.7) × (4.0–) 4.1–5.3 (–5.8) μm (n = 30/1), L = 11.88, W = 4.75, Q = 2.52. Those latter specimens were used by us for DNA studies. However, the ITS sequence newly obtained from the type specimen of *E. cinnamomescens* shows that it is conspecific with *M. populinum*. It means that the species named as *M. cinnamomescens* in our previous publications needs a new species epithet.

There are four names for the European exidioid *Myxarium* spp. which had remained unaddressed in our earlier studies, i.e. *Tremella gemmata* Lév., *Exidia corrugativa* Bref., *Tremella ilicis* Boud., and *Exidia alboglobosa* Lloyd. The type material of *T. gemmata* (in PC, studied) belongs to *M. hyalinum* as typified and redescribed in Spirin et al. (2018). As far as we know, no extant material exists for *E. corrugativa* and *T. ilicis*. In our opinion, their original illustrations (published by Brefeld 1888; Boudier 1911, correspondingly) again represent *M. hyalinum*. Finally, the type of *E. alboglobosa* (BPI 701610, studied) most likely belongs to a sister species of *M. nucleatum* Wallr., devoid of mineral inclusions (see comments in Spirin et al. 2019: 6–7). This necessitates introducing *M. cinnamomescens* sensu auct. as a new species:

#### Myxarium corticale

Taxon classificationAnimaliaAuricularialesHyaloriaceae

Savchenko, Spirin & Malysheva sp. nov.

E3F159EA-E3BE-50C4-BBE6-B730821F636E

862508

##### Holotype.

Norway • Akershus: Ås, Ved Høyskolen, *Populus tremula* (fallen corticated branch), 10 Dec 1972, Hansen & Gulden 595/72* (O F160494).

##### Etymology.

Corticalis (Lat., adj.) – bark-inhabiting, in reference to the occurrence on corticated tree branches.

##### Description.

Basidiomata annual, gelatinous, first semitranslucent, pustulate, about 1 mm in diam., then fusing together, cerebriform or adpressed-orbicular, finally opalescent or opaque, up to 8 mm in the widest dimension and 1–5 mm thick. Hymenial surface hyaline, often with light yellowish or brownish tints, then almost white, indistinctly furrowed to more or less smooth, in herbarium specimens pale ochraceous or brownish. Mineral inclusions usually absent or, if present, very small, embedded in hymenial layer and seen only under magnification. Margin detaching, later adnate, compact, more or less concolourous with hymenial surface.

Hyphal structure monomitic, hyphae clamped (clamps often open); context hyphae hyaline, predominantly interwoven, thin-walled or with a distinct wall, 3–6 μm in diam., often clearly swollen at septa and then up to 9 μm in diam., subhymenial hyphae hyaline, thin-walled, mostly ascending, 1–2.5 μm in diam. Cystidia absent. Hyphidia richly branched, thin-walled, hyaline, apical branches 1–2 μm in diam., covering hymenial surface. Basidia four-celled, ellipsoid-ovoid to subglobose, 11–17.5 × 7–13 μm, with a well-pronounced basal enucleate stalk up to 65 × 3.5 μm. Basidiospores thin-walled, narrowly to broadly cylindrical, slightly to distinctly curved, (8.6–) 8.9–14.0 (–14.5) × (3.4–) 3.5–5.7 (–5.8) μm (n = 180/6), L = 10.20–12.21, W = 4.15–5.01, Q = 2.38–2.64.

##### Remarks.

*Myxarium corticale* was illustrated as *M. cinnamomescens* in Spirin et al. (2018, 2019). All specimens named as *M. cinnamomescens* in the latter two papers (except for the type of *E. cinnamomescens*), as well as in Põldmaa et al. (2019) and Spirin et al. (2025), belong to *M. corticale*. Phylogenetically, *M. corticale* is a sister species of *M. populinum*. In most cases, they can be differentiated due to their host preference: *M. populinum* seems to be restricted to *Populus tremula*, with a few records on *Salix* spp., while *M. corticale* occurs on different angiosperm hosts, most often on *Betula*, *Fraxinus*, *Padus*, and *Tilia*. However, it may inhabit *P. tremula*, too. Only the aspen-dwelling specimens with an average spore width exceeding 4.8 μm can be safely identified as *M. corticale* and those ones with an average spore width less than 4.1 μm as *M. populinum*. With our current knowledge, morphological recognition is more or less impossible for the specimens on aspen with the average spore width falling between 4.1 and 4.8 μm. In these cases, *M. corticale* and *M. populinum* can be differentiated exclusively via ITS sequencing.

#### Exidia fuliginea

Taxon classificationAnimaliaAuricularialesAuriculariaceae

Mont., Historia física y política de Chile, Botánica 7: 393, 1850

5F547508-5E3D-5695-9EE8-720A787CE986

##### Holotype.

Chile • O’Higgins: Rancagua, rotten log, [no collecting date], Bertero 701 (Montagne’s herbarium #1104) (PC, studied).

*Exidia fuliginea* was described from Chile (Gay 1850) and is so far known only from the type specimen. Macroscopic traits, i.e. dark-brown resupinate basidiomata with a somewhat lobed margin and spinose hymenial surface (1–2 projections per mm), as well as encrusted, blackish hyphidia and cylindrical, curved basidiospores, (9.8–) 10.0–14.2 (–16.8) × (3.6–) 3.9–5.2 (–5.3) μm (n = 20/1), L = 12.21, W = 4.46, Q = 2.76, point to *Exidia* as an appropriate genus for this species. However, sequenced material of *E. fuliginosa* is necessary to infer its phylogenetic relationships with other *Exidia* spp.

#### Exidia novozealandica

Taxon classificationAnimaliaAuricularialesAuriculariaceae

Lloyd, Mycological Writings 7 (75): 1356, 1925

D7F92FEC-D83E-5963-8DF9-1DD16338F168

##### Note.

In the protologue, Lloyd (1925) compared *E. novozealandica* with *E. japonica* and differentiated it from the latter species by its rougher, black hymenial projections. Cooper (2023) combined *E. novozealandica* in *Tremellochaete* although no justification was provided for this recombination. The identity of *E. novozealandica* deserves further study. See also the notes on *E. tenax* below.

#### Exidia punctata

Taxon classificationAnimaliaAuricularialesAuriculariaceae

L. Wang & C.L. Zhao, Phytotaxa 689: 33, 2025

B2EB4239-6890-5E55-8000-50FA3178F3A8

##### Note.

Phylogenetic data place *E. punctata*, recently described from China, in the genus *Adustochaete* (Figs 13, 14). The original description, although very sparse, points to this genus as well (Jiang et al. 2025). However, *E. punctata* cannot be combined in *Adustochaete*, due to the existence of another *Adustochaete punctata* described a year before (Dong et al. 2024). Therefore, a new name is needed. We are, however, unwilling to introduce it without studying authentic material of *E. punctata*. The environmental sequence GU054135 in GenBank belongs to *E. punctata* or its close relative (Fig. 14).

#### Exidia purpureocinerea

Taxon classificationAnimaliaAuricularialesAuriculariaceae

MacOwan, Grevillea 10 (55): 106, 1882

E8826507-A4EB-5074-8D1F-E57D4AB847B7

##### Holotype.

South Africa. Eastern Cape: Somerset East, Boschberg, [no collecting date], MacOwan (K(M), isotype – S F20629, studied).

The type material of *E. purpureocinerea* is nearly sterile and badly infected, and only two turgid basidiospores were found, measuring 8.2–9.7 × 3.4–3.8 μm. These spore measurements, combined with the rather light-colored basidiomata and the presence of obtuse, regularly arranged (3–4 per mm, up to 70 × 60 μm) projections indicate that *E. purpureocinerea* is a member of the *E. japonica* complex. Morphologically, it is very similar to or probably even conspecific with *Tremellochaete atlantica* R.L.M. Alvarenga. The latter species was originally described from Brazil (Phookamsak et al. 2019) and later reported from the southern part of China (Tohtirjap et al. 2023). However, we refrain from synonymizing these species awaiting sequenced material of *E. purpureocinerea* from South Africa. *Exidia tanzanica* is another *Tremellochaete*-like species from Africa (see description above). It differs from *E. purpureocinerea* in having darker basidiomata with more densely arranged hymenial projections, as well larger basidia and basidiospores.

#### Exidia saccharina f. carnea

Taxon classificationAnimaliaAuricularialesAuriculariaceae

Raitv., Plants and Animals of the Far East: 116, 1971

87E22DCF-733F-5EED-B6D2-D37986359F14

##### Holotype.

Russia • Krasnoyarsk Reg.: Mana Dist., Oreshnoye, *Picea obovata*, 6 Sep 1958, Parmasto (TAAM 9819*, studied).

The ITS sequence obtained from the type of material of E. saccharina f. carnea confirms that it should be treated as a synonym of *Ditangium cerasi* (*Sebacinales*).

#### Exidia tenax

Taxon classificationAnimaliaAuricularialesAuriculariaceae

Cooke, Grevillea 8 (46): 57, 1879

3BE132DB-A1FB-5996-8DD2-33C0BA0161DF

##### Holotype.

New Zealand. Southland: Winton, on branches, [no collecting date], Berggren 111 (K(M) 1435722, studied).

After studying the type material of *E. tenax*, we concluded that it is yet another species in the *Exidia japonica* complex. The darker basidiomata and narrower basidiospores of *E. tenax* (measured as (10.1–) 10.5–15.4 (–16.3) × (3.4–) 4.1–5.2 (–5.4) μm (n = 23/1), L = 12.51, W = 4.59, Q = 2.74) preclude its conspecificity with *Tremellochaete australiensis* F. Wu et al. recently described from Australia (Tohtirjap et al. 2023). It is possible that *E. novozealandica* introduced from the same geographic area is a younger synonym of *E. tenax*. Alternatively, *E. novozealandica* may turn out to be an older name for *T. australiensis*. Solving these questions requires a thorough study of the *E. japonica* group in Oceania, as well as the type study of *E. novozealandica*.

#### Exidiopsis citrina

Taxon classificationAnimaliaAuricularialesAuriculariaceae

Hauerslev, Mycotaxon 49: 221, 1993

6EECB587-75EA-5B77-B57F-7CC5B18626E7

##### Holotype.

Denmark • Hovedstaden: Lyngby-Taarbæk, Ørholm, *Salix* sp. (?), 19 Nov 1976, Hauerslev 5257 (C 19751, studied).

*Exidiopsis citrina* was introduced based on a single specimen from Denmark (Hauerslev 1993). Rather small, predominantly pyriform basidiospores, as well as constantly two-celled basidia with relatively thick sterigmata are atypical for the *Auriculariaceae*. This species is definitely not a member of *Exidiopsis* as reintroduced above but its taxonomic position cannot be defined without DNA data.

#### Exidiopsis diversa

Taxon classificationAnimaliaAuricularialesAuriculariaceae

K. Wells, Mycologia 79: 280, 1987

90625C14-1998-51ED-A029-92EB4A5E47CF

##### Holotype.

Canada • British Columbia: Princeton, Asp Creek, *Populus* sp., 25 May 1979, Wells 2769 (BPI871728 – isotype, studied).

Morphological and cultural descriptions of *E. diversa* were provided by Wells (1987). Microscopic traits (shape and size of basidia and basidiospores) put it close to *Tegmenticium peritrichum* although the basidiomata of *E. diversa* are much thinner than in the latter species, ca. 0.05 mm thick, and they lack the differentiated cystidia characteristic of *T. peritrichum*. This species deserves further study with the use of DNA methods.

#### Exidiopsis invasa

Taxon classificationAnimaliaAuricularialesAuriculariaceae

Hauerslev, Mycotaxon 49: 221, 1993

8370AF5C-70F9-5202-B7F9-2DAEF220BF46

##### Note.

*Exidiopsis invasa* was described as an intrahymenial parasite of *Trechispora microspora* (P. Karst.) Liberta (*Trechisporales*) (Hauerslev 1993). Morphological traits (small basidia, subglobose basidiospores and the presence of haustorial cells) suggest that *E. invasa* is most likely a member of the *Tremellomycetes*. No modern collections of this species are known to us.

#### Exidiopsis pulverea

Taxon classificationAnimaliaAuricularialesAuriculariaceae

Hauerslev, Mycotaxon 49: 225, 1993

A615A07C-3C64-507C-8128-79A894679E8F

##### Holotype.

Denmark • Midtjylland: Barrit, Stagsrode Skov, *Cryptomeria japonica*, 12 Oct 1963, Hauerslev 2209 (C19750, studied).

The species possesses gloeocystidia and basidia covered by involucres and should be therefore treated as a member of *Basidiodendron* s. lato. Its phylogenetic position is the subject of a forthcoming publication.

#### Exidiopsis tenuis

Taxon classificationAnimaliaAuricularialesAuriculariaceae

Hauerslev, Mycotaxon 49: 227, 1993

682F7D58-326D-501C-88CF-81551AEDF4CF

##### Holotype.

Denmark • Hovedstaden: Holte, on wood, 20 May 1984, Hauerslev 6323 (C 19749, studied).

Morphological traits place *E. tenuis* in the vicinity of *Basidiodendron radians* (Rick) P. Roberts s. lato. The identity of *E. tenuis* will be clarified on another occasion.

#### Heterochaete mussooriensis

Taxon classificationAnimaliaAuricularialesAuriculariaceae

Bodman, Lloydia 15: 221, 1952

69224ECD-7839-54D3-B0BE-081208793E51

##### Holotype.

India • Uttarakhand: Mussoorie, dead wood, 20 Jul 1940, Ahmad 375 (NY00738345, studied).

Li et al. (2023) combined *H. mussooriensis* from India in *Heteroradulum* and placed *Heteroradulum semis* Spirin & Malysheva, recently described from East Asia (Malysheva and Spirin 2017), among its synonyms. However, they did not study the type specimen of *H. mussooriensis* and based their synonymy merely on reading the protologue. We did study the type of *H. mussooriensis* in 2016, before *H. semis* was introduced, and our study showed that these taxa are not conspecific. *Heterochaete mussooriensis* has pale, thin (up to 200 μm thick) basidiomata with adnate and rather poorly differentiated margin which are strongly different from the bright-colored, considerably thicker, partly detaching basidiomata of *H. semis*. Moreover, hymenial spines of *H. mussooriensis* are more sparsely arranged (4–5 per mm) than in *H. semis* (5–7 per mm). We could not find any ripe spores in the type specimen of *H. moossoriensis* although measurements given in the protologue (Bodman 1952), 12–15 × 9–10 μm, definitely preclude *H. semis* (basidiospores measured 11–14 × 6–7.5 μm). Nevertheless, morphological traits of *H. mussooriensis* point to *Heteroradulum* as the correct genus for it but its position therein should be re-assured with the use of DNA data.

#### Heterochaete nigerrima

Taxon classificationAnimaliaBragantiaAuriculariaceae

Viégas, Bragantia 5: 240, 1945

BF3697B3-AD6C-530E-8182-A6DE6A5CFC26

= Tremellochaete nigerrima (Viégas) Spirin & Malysheva, Fungal Biology 121: 714, 2017.

##### Holotype.

Brazil • São Paulo: Campinas, Arraial dos Souzas, 22 Sep 1935, Krug 1157 (NY00738346 – isotype, studied).

This species shows a great similarity to *Exidia japonica*, the generic type of *Tremellochaete*, and it was therefore combined in that genus (Malysheva and Spirin 2017). No sequences of *H. nigerrima* have been available so far. Considering the existence of several competing names for *Tremellochaete* spp. from the tropics, we refrain from recombining *H. nigerrima* in *Exidia* until the species is connected to newly collected material and DNA data.

#### Sebacina candida

Taxon classificationAnimaliaAplousobranchiaPolycitoridae

L.S. Olive, Bulletin of the Torrey Botanical Club 85: 21, 1958

729CE834-1707-5AFD-95AC-1198388BDEDD

##### Holotype.

Tahiti • Faa Tautia Valley, corticated dead limbs, 9 Aug 1956, Olive T442 (NY00738349, studied).

After studying the type specimen of *S. candida*, we concluded that it is most likely a member of *Proterochaete* and it probably belongs in the vicinity of *P. latispora*, another representative of the genus with distinctly ellipsoid basidiospores. However, the basidiospores of *S. candida* are clearly smaller than in *P. latispora*, 6.8–9.3 × 5.1–6.0 μm (n = 8). The identity of *S. candida* should be re-established based on sequenced material from Polynesia. Wells (1961) combined *S. candida* in *Exidiopsis* and reintroduced it based on a wider set of specimens from the neotropics. However, the conspecificity of this material with *S. candida* s. typi is questionable. Furthermore, Wells (ibid.) mentioned *Tremella sordida* Speg. originally described from Tierra del Fuego as an older name for *S. candida* which eventually could not have been combined in *Exidiopsis*, due to the existence of *Exidiopsis sordida* (S.L. Olive) K. Wells (= *Sebacina sordida* S.L. Olive). The latter species was compared by Wells with the European *E. grisea* (= *Ulocolla grisea* in the present study). No modern, sequenced material is known to us for either *T. sordida* or *S. sordida*.

#### Sebacina glauca

Taxon classificationAnimaliaAuricularialesAuriculariaceae

Pat., Bulletin de la Société Mycologique de France 9: 140, 1893

3122F253-EE73-5EAE-A359-6AC1944A543D

##### Holotype.

Ecuador • Pichincha, dead branches, Mar 1892, Lagerheim (FH00060387, studied).

*Sebacina glauca* was described from Ecuador (Patouillard and Lagerheim 1893) and it remains known from the type material only. The type specimen is in good condition and fertile. Morphological traits point to *Proterochaete* (especially to *P. macrogyna* and *P. paniculata*) as the most appropriate genus for *S. glauca*. However, we refrain from making a formal recombination of it in *Proterochaete* before obtaining more material and DNA sequences. Wells (1961) placed *S. glauca* in the synonyms of the widely defined *Exidiopsis grisea* (= *Ulocolla grisea* in the present study). However, this synonymy should be abandoned.

#### Sebacina livescens

Taxon classificationAnimaliaPinalesPinaceae

Bres., Fungi Tridentini 2 (11–13): 64, 1898

14BFD2AB-3D60-505F-A160-FE7654887098

##### Type materials.

Lectotype (selected by Spirin et al. 2025: 370). Italy • Trentino-Alto Adige: Trento, Andalo, *Abies alba* (rotten log), Aug 1896, Bresadola (S F29132, studied).

The species was combined in *Exidiopsis* by Bourdot and Maire (1920). It was recently shown to be a member of *Protohydnum (Auriculariales)* (Spirin et al. 2025).

#### Sebacina opalea

Taxon classificationAnimaliaAuricularialesAuriculariaceae

Bourdot & Galzin, Bulletin de la Société Mycologique de France 39: 262, 1924

D7F769DE-EA4D-5639-8B81-555378C1B2FE

##### Note.

*Sebacina opalea* was described from France (Bourdot and Galzin 1924, 1927) and later reintroduced as a member of *Exidiopsis* by Reid (1970) and Roberts (1993). The species has not been formally typified yet. However, our study of the authentic material, as well as newly collected specimens confirm that *S. opalea* should be removed from the *Auriculariales*. Its reintroduction and taxonomic replacement is the subject of a forthcoming publication.

#### Sebacina umbrina

Taxon classificationAnimaliaAplousobranchiaPolycitoridae

D.P. Rogers, University of Iowa Studies in Natural History 17: 38, 1935

ADDF6B5F-6ED5-5997-A195-B562A3347183

##### Note.

This species was originally described from Iowa, USA (Rogers 1935) and later reported from Denmark (Christiansen 1959) and Germany (Oberwinkler 1963). Descriptions of *S. umbrina* by the latter authors certainly refer to dark-colored individuals of *Proterochaete letendreana*, which is widely distributed in temperate Europe. We had no opportunity to study the type material of *S. umbrina* and its identity therefore remains unclear. Wojewoda (1981) combined *S. umbrina* in *Exidiopsis*. However, its taxonomic position should be reassessed based on DNA data.

#### Ulocolla badioumbrina

Taxon classificationAnimaliaTremellalesExidiaceae

Bres., Annales Mycologici 1: 115, 1903

E42B97EC-8F8E-5B0A-9E42-1FC79FBEDA68

##### Syntypes.

Poland. [no exact locality indicated], *Alnus glutinosa* and *Salix cinerea*, Eichler (S F20292, 20289, studied).

*Ulocolla badioumbrina* was combined in *Exidia* by Killermann (1928). It was recently moved to the genus *Globulisebacina (Sebacinales)* (Friebes and Spirin 2025).

## Discussion

In this paper, 64 accepted species of the *Auriculariaceae* were investigated, primarily those which were earlier considered as members of *Exidia* and *Exidiopsis*. Additionally, twenty-one species are treated under excluded taxa, all but three of which await further taxonomic revisions. Our study focused mainly on European species; in total, 34 species from the continent, including all previously described *Exidia* and *Exidiopsis* spp. in the *Auriculariaceae*, are reintroduced or described as new and reclassified according to the newly justified generic division of the family. The identity of *Exidia pithya* and its sister species clearly requires further study with the use of additional genetic markers.

Evidently, the genus-level reclassification of the *Auriculariaceae* proposed here is a product of our contemporary, still quite limited knowledge of the target group. In particular, species sampling from tropical areas is fragmentary. This means that newly recorded taxa added to the multigene phylogeny of the family may challenge our genus concepts. Nevertheless, we believe that the main outline of the redefined or newly introduced genera will remain unchanged. Of all species dealt with in the present study, only six could be regarded as representing difficult cases blurring, to a certain extent, the morphological concepts of the accepted genera. These are *Adustochaete hiemalis* versus *Leiostroma* spp. versus *Proterochaete letendreana*, and *Descidia thuretiana* versus effused *Exidia* spp. (*E. delita* and *E. plumbescens*). In both cases, a meticulous morphological study is required to distinguish between them, meaning that, in many cases, the generic affiliation of a specimen at hand cannot be grasped at first sight.

Due to the lack of non-ribosomal genetic markers, the phylogenetic position of seven existing genera, *Alloexidiopsis*, *Crystallodon*, *Heterochaete* s. str., *Hirneolina*, *Metulochaete*, *Nodulochaete*, and *Punctochaete*, remains uncertain. Moreover, two lineages represented in our multigene trees, viz. the *Elmerina* and *Exidiopsis* s. str. – *Heteroradulum* clades, are awaiting their reclassification based on a much denser species sampling. Another problem that we could not satisfactorily solve with our current data is determining the proper limits of the family *Auriculariaceae*. In our phylogenetic reconstructions, *Pholiobasidion senex* was recovered as sister to the rest of the *Auriculariaceae*. We considered it as belonging to this family mainly because of its higher genetic proximity to the *Auriculariaceae* than to the known genera of the residual *Auriculariales*. It is possible, however, that a future all-encompassing multigene phylogeny of the *Auriculariales* will place it outside of the family.

## Supplementary Material

XML Treatment for Adustochaete

XML Treatment for Adustochaete fumida

XML Treatment for Adustochaete gloeoapicata

XML Treatment for Adustochaete hiemalis

XML Treatment for Adustochaete minuta

XML Treatment for Adustochaete nivea

XML Treatment for Adustochaete rava

XML Treatment for Adustochaete scalena

XML Treatment for Adustochaete tribulata

XML Treatment for Descidia

XML Treatment for Descidia repanda

XML Treatment for Descidia thuretiana

XML Treatment for Eichleriella

XML Treatment for Eichleriella macrochaeta

XML Treatment for Elmericium

XML Treatment for Elmericium spinosum

XML Treatment for Exidia

XML Treatment for Exidia candida

XML Treatment for Exidia ciliata

XML Treatment for Exidia crenata

XML Treatment for Exidia delita

XML Treatment for Exidia friesiana

XML Treatment for Exidia glandulosa

XML Treatment for Exidia impressa

XML Treatment for Exidia inornata

XML Treatment for Exidia japonica

XML Treatment for Exidia pithya

XML Treatment for Exidia plumbescens

XML Treatment for Exidia recisa

XML Treatment for Exidia reflexa

XML Treatment for Exidia repleta

XML Treatment for Exidia saturata

XML Treatment for Exidia semiorbis

XML Treatment for Exidia spiculata

XML Treatment for Exidia straminea

XML Treatment for Exidia subglandulosa

XML Treatment for Exidia tanzanica

XML Treatment for Exidia truncata

XML Treatment for Exidia umbrinella

XML Treatment for Exidia uvapassa

XML Treatment for Exidia yadongensis

XML Treatment for Exidia zelleri

XML Treatment for Exidiopsis

XML Treatment for Exidiopsis effusa

XML Treatment for Exidiopsis perflua

XML Treatment for Leiostroma

XML Treatment for Leiostroma farinellum

XML Treatment for Leiostroma incanum

XML Treatment for Leiostroma infecundum

XML Treatment for Leiostroma squalidum

XML Treatment for Pholiobasidion

XML Treatment for Pholiobasidion senex

XML Treatment for Proterochaete

XML Treatment for Proterochaete abstrusa

XML Treatment for Proterochaete adusta

XML Treatment for Proterochaete africana

XML Treatment for Proterochaete bambusicola

XML Treatment for Proterochaete furfurosa

XML Treatment for Proterochaete latispora

XML Treatment for Proterochaete letendreana

XML Treatment for Proterochaete macrogyna

XML Treatment for Proterochaete mucedinea

XML Treatment for Proterochaete paniculata

XML Treatment for Proterochaete stipidosa

XML Treatment for Scrupulispora

XML Treatment for Scrupulispora perparvula

XML Treatment for Tegmenticium

XML Treatment for Tegmenticium peritrichum

XML Treatment for Ulocolla

XML Treatment for Ulocolla corticioides

XML Treatment for Ulocolla grisea

XML Treatment for Ulocolla griseobrunnea

XML Treatment for Ulocolla orbiculata

XML Treatment for Ulocolla pinicola

XML Treatment for Ulocolla saccharina

XML Treatment for Ulocolla subrepanda

XML Treatment for Exidia benieri

XML Treatment for Exidia cinnamomescens

XML Treatment for Myxarium corticale

XML Treatment for Exidia fuliginea

XML Treatment for Exidia novozealandica

XML Treatment for Exidia punctata

XML Treatment for Exidia purpureocinerea

XML Treatment for Exidia saccharina f. carnea

XML Treatment for Exidia tenax

XML Treatment for Exidiopsis citrina

XML Treatment for Exidiopsis diversa

XML Treatment for Exidiopsis invasa

XML Treatment for Exidiopsis pulverea

XML Treatment for Exidiopsis tenuis

XML Treatment for Heterochaete mussooriensis

XML Treatment for Heterochaete nigerrima

XML Treatment for Sebacina candida

XML Treatment for Sebacina glauca

XML Treatment for Sebacina livescens

XML Treatment for Sebacina opalea

XML Treatment for Sebacina umbrina

XML Treatment for Ulocolla badioumbrina

## Additional information

### Artificial Intelligence (AI) use

The authors accept full responsibility for the content of the manuscript, including the disclosure of any use of AI.

No AI tools were used in the preparation of this manuscript.

### Adherence to national and international regulations

All the fungal strains used in this study have been legally obtained, respecting the Convention on Biological Diversity (Rio Convention).

### Author ORCIDs

V. Spirin https://orcid.org/0000-0001-5436-6997

V. Malysheva https://orcid.org/0000-0002-7403-9616

N. Schoutteten https://orcid.org/0000-0002-5746-7344

A. Savchenko https://orcid.org/0000-0001-6377-6349

T. Læssøe https://orcid.org/0000-0003-4710-2648

L. Ryvarden https://orcid.org/0000-0002-4670-7306

D. Grootmyers https://orcid.org/0000-0002-1042-4902

J. Vlasák https://orcid.org/0000-0002-5363-4459

M.V. Vignale https://orcid.org/0000-0001-9253-199X

I. Viner https://orcid.org/0000-0002-5193-7720

### Data availability

All of the data that support the findings of this study are available in the main text or Supplementary Information.

## Supplementary materials

Supplementary material 1 Specimens examined Data type doc https://binary.pensoft.net/file/1730457This dataset is made available under the Open Database License (http://opendatacommons.org/licenses/odbl/1.0/). The Open Database License (ODbL) is a license agreement intended to allow users to freely share, modify, and use this Dataset while maintaining this same freedom for others, provided that the original source and author(s) are credited. Viacheslav Spirin, Vera Malysheva, Nathan Schoutteten, Anton Savchenko, Bernard Rivoire, Jan Olsson, Thomas Læssøe, Leif Ryvarden, Django Grootmyers, Matti Kulju, Josef Vlasák, María Victoria Vignale, Ilya Viner

Supplementary material 2 DNA sequences used in the present study Data type doc https://binary.pensoft.net/file/1730458This dataset is made available under the Open Database License (http://opendatacommons.org/licenses/odbl/1.0/). The Open Database License (ODbL) is a license agreement intended to allow users to freely share, modify, and use this Dataset while maintaining this same freedom for others, provided that the original source and author(s) are credited. Viacheslav Spirin, Vera Malysheva, Nathan Schoutteten, Anton Savchenko, Bernard Rivoire, Jan Olsson, Thomas Læssøe, Leif Ryvarden, Django Grootmyers, Matti Kulju, Josef Vlasák, María Victoria Vignale, Ilya Viner

Supplementary material 3 DNA sequence alignments used in the analyses Data type doc https://binary.pensoft.net/file/1730460This dataset is made available under the Open Database License (http://opendatacommons.org/licenses/odbl/1.0/). The Open Database License (ODbL) is a license agreement intended to allow users to freely share, modify, and use this Dataset while maintaining this same freedom for others, provided that the original source and author(s) are credited. Viacheslav Spirin, Vera Malysheva, Nathan Schoutteten, Anton Savchenko, Bernard Rivoire, Jan Olsson, Thomas Læssøe, Leif Ryvarden, Django Grootmyers, Matti Kulju, Josef Vlasák, María Victoria Vignale, Ilya Viner

Supplementary material 4 Phylogeny of *Leiostroma* inferred from the ITS dataset using BI analysis Data type pdf Explanation note Bayesian posterior probabilities followed by ML bootstrap values are shown at nodes; branch lengths reflect estimated number of changes per site. https://binary.pensoft.net/file/1730461This dataset is made available under the Open Database License (http://opendatacommons.org/licenses/odbl/1.0/). The Open Database License (ODbL) is a license agreement intended to allow users to freely share, modify, and use this Dataset while maintaining this same freedom for others, provided that the original source and author(s) are credited. Viacheslav Spirin, Vera Malysheva, Nathan Schoutteten, Anton Savchenko, Bernard Rivoire, Jan Olsson, Thomas Læssøe, Leif Ryvarden, Django Grootmyers, Matti Kulju, Josef Vlasák, María Victoria Vignale, Ilya Viner

Supplementary material 5 Phylogeny of the *Exidia* clade in the *Auriculariaceae* inferred from the ITS + LSU dataset using BI analysis Data type pdf Explanation note Bayesian posterior probabilities followed by ML bootstrap values are shown at nodes; branch lengths reflect estimated number of changes per site. https://binary.pensoft.net/file/1730462This dataset is made available under the Open Database License (http://opendatacommons.org/licenses/odbl/1.0/). The Open Database License (ODbL) is a license agreement intended to allow users to freely share, modify, and use this Dataset while maintaining this same freedom for others, provided that the original source and author(s) are credited. Viacheslav Spirin, Vera Malysheva, Nathan Schoutteten, Anton Savchenko, Bernard Rivoire, Jan Olsson, Thomas Læssøe, Leif Ryvarden, Django Grootmyers, Matti Kulju, Josef Vlasák, María Victoria Vignale, Ilya Viner
