# Supplementary material for: Exidia and Exidiopsis (Auriculariales, Basidiomycota): a reconsideration
Source: IMA Fungus. 2026 Jul 29;17:e189042. doi: 10.3897/imafungus.17.189042 (PMC13444229; doi:10.3897/imafungus.17.189042)
Supplement: Supplementary file 1 [file imafungus-17-e189042-s004.pdf]

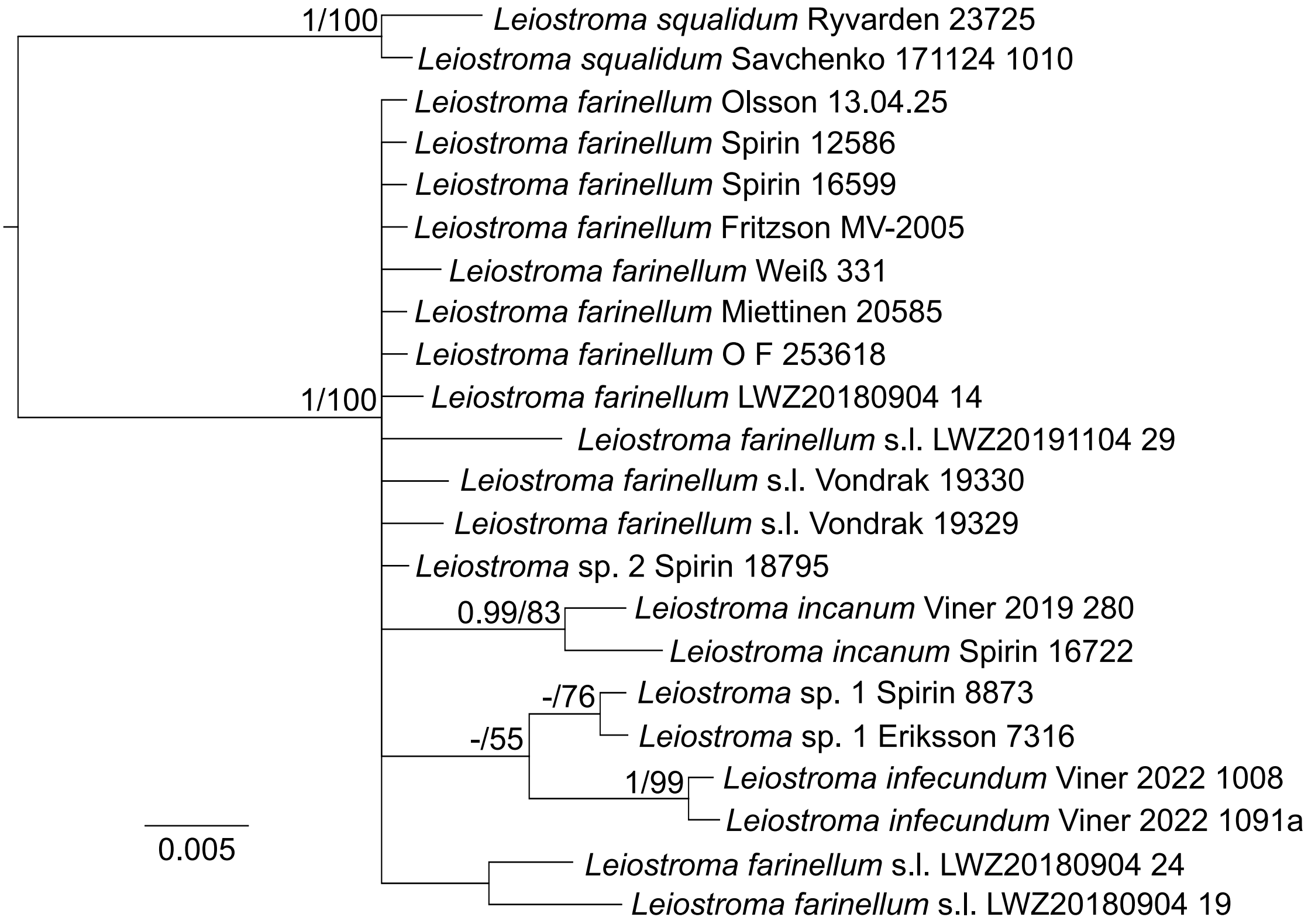
1/100
Leiostroma squalidum Ryvarden 23725
Leiostroma squalidum Savchenko 171124 1010
Leiostroma farinellum Olsson 13.04.25
Leiostroma farinellum Spirin 12586
Leiostroma farinellum Spirin 16599
Leiostroma farinellum Fritzson MV-2005
Leiostroma farinellum Weiß 331
Leiostroma farinellum Miettinen 20585
Leiostroma farinellum O F 253618
1/100
Leiostroma farinellum LWZ20180904 14
Leiostroma farinellum s.l. LWZ20191104 29
Leiostroma farinellum s.l. Vondrak 19330
Leiostroma farinellum s.l. Vondrak 19329
Leiostroma sp. 2 Spirin 18795
0.99/83
Leiostroma incanum Viner 2019 280
Leiostroma incanum Spirin 16722
-/76
Leiostroma sp. 1 Spirin 8873
-/55
Leiostroma sp. 1 Eriksson 7316
1/99
Leiostroma infecundum Viner 2022 1008
Leiostroma infecundum Viner 2022 1091a
0.005
Leiostroma farinellum s.l. LWZ20180904 24
Leiostroma farinellum s.l. LWZ20180904 19
