# Supplementary material for: Exidia and Exidiopsis (Auriculariales, Basidiomycota): a reconsideration
Source: IMA Fungus. 2026 Jul 29;17:e189042. doi: 10.3897/imafungus.17.189042 (PMC13444229; doi:10.3897/imafungus.17.189042)
Supplement: Supplementary file 2 [file imafungus-17-e189042-s005.pdf]

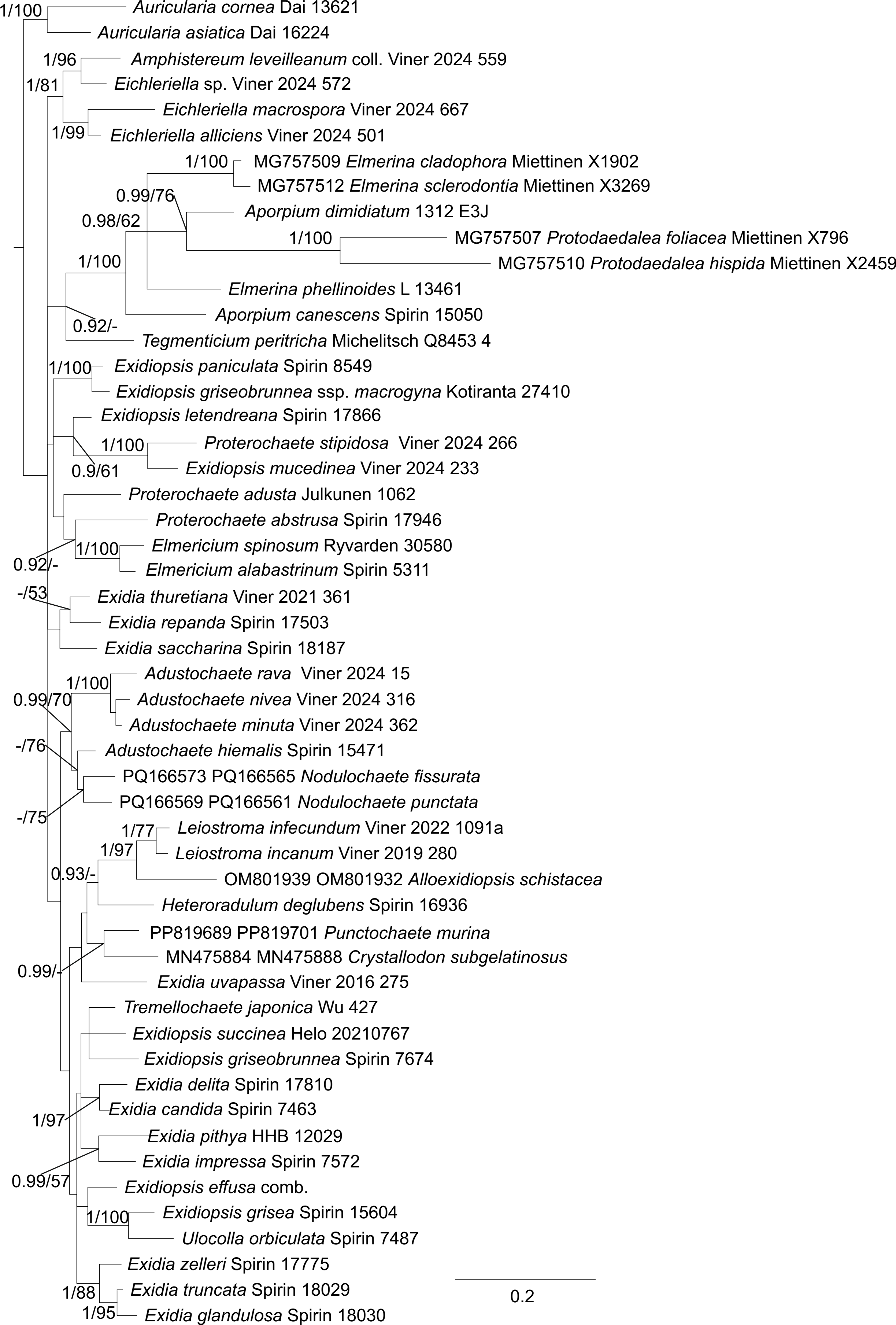

1/100
Auricularia cornea Dai 13621
Auricularia asiatica Dai 16224
1/96
Amphistereum leveilleanum coll. Viner 2024 559
1/81
Eichleriella sp. Viner 2024 572
Eichleriella macrospora Viner 2024 667
1/99
Eichleriella alliciens Viner 2024 501
1/100
MG757509 Elmerina cladophora Miettinen X1902
MG757512 Elmerina sclerodontia Miettinen X3269
0.99/76
Aporpium dimidiatum 1312 E3J
0.98/62
1/100
MG757507 Protodaedalea foliacea Miettinen X796
MG757510 Protodaedalea hispida Miettinen X2459
1/100
Elmerina phellinoides L 13461
Aporpium canescens Spirin 15050
0.92/-
Tegmenticium peritricha Michelitsch Q8453 4
Exidiopsis paniculata Spirin 8549
1/100
Exidiopsis griseobrunnea ssp. macrogyna Kotiranta 27410
Exidiopsis letendreana Spirin 17866
1/100
Proterochaete stipidosa Viner 2024 266
0.9/61
Exidiopsis mucedinea Viner 2024 233
Proterochaete adusta Julkunen 1062
Proterochaete abstrusa Spirin 17946
1/100
Elmericium spinosum Ryvarden 30580
0.92/-
Elmericium alabastrinum Spirin 5311
-/53
Exidia thuretiana Viner 2021 361
Exidia repanda Spirin 17503
Exidia saccharina Spirin 18187
Adustochaete rava Viner 2024 15
1/100
0.99/70
Adustochaete nivea Viner 2024 316
Adustochaete minuta Viner 2024 362
-/76
Adustochaete hiemalis Spirin 15471
PQ166573 PQ166565 Nodulochaete fissurata
PQ166569 PQ166561 Nodulochaete punctata
-/75
1/77
Leiostroma infecundum Viner 2022 1091a
1/97
Leiostroma incanum Viner 2019 280
0.93/-
OM801939 OM801932 Alloexidiopsis schistacea
Heteroradulum deglubens Spirin 16936
PP819689 PP819701 Punctochaete murina
MN475884 MN475888 Crystallodon subgelatinosus
0.99/-
Exidia uvapassa Viner 2016 275
Tremellochaete japonica Wu 427
Exidiopsis succinea Helo 20210767
Exidiopsis griseobrunnea Spirin 7674
Exidia delita Spirin 17810
Exidia candida Spirin 7463
1/97
Exidia pithya HHB 12029
Exidia impressa Spirin 7572
0.99/57
Exidiopsis effusa comb.
Exidiopsis grisea Spirin 15604
1/100
Ulocolla orbiculata Spirin 7487
Exidia zelleri Spirin 17775
Exidia truncata Spirin 18029
1/88
1/95
Exidia glandulosa Spirin 18030
0.2
