# Supplementary material for: Exidia and Exidiopsis (Auriculariales, Basidiomycota): a reconsideration
Source: IMA Fungus. 2026 Jul 29;17:e189042. doi: 10.3897/imafungus.17.189042 (PMC13444229; doi:10.3897/imafungus.17.189042)
Supplement: Supplementary file 3 [file imafungus-17-e189042-s001.doc]

**Supplementary material 1. Specimens examined** (type specimens are cited in the main text and not repeated here)

*Adustochaete gloeoapicata*. Mexico. Veracruz: Xalapa, Botanical Garden, dead corticated branches, 22 Sep 1985, Ryvarden 23268 (O, H).

*Adustochaete hiemalis*. France. Aveyron: Millau, Dourbie, *Clematis vitalba*, 10 May 2022 Spirin 15471*** (H). Lozère: Saint-Germain-du-Teil, *Lavandula* sp., 30 Jun 2022, Spirin 15766*** (H).

*Adustochaete minuta*. Argentina. Misiones: Iguazu, Reserva Selva Iryapú, near IMiBio (Instituto Misionero de Biodiversidad), angiosperm wood, 10 May 2024, Viner 2024/362* (H, SLP). Mexico. Veracruz: Actopan, La Mancha, dead corticated branches, 23 Sep 1985, Ryvarden 23395, 23430 (O); Xalapa, Botanical Garden, dead corticated branch, 22 Sep 1985, Ryvarden 23233 (O).

*Adustochaete nivea*. Argentina. Misiones: General Manuel Belgrano, Reserva Natural Yate-I, angiosperm wood, 8 May 2024, Viner 2024/316* (H, SLP). Mexico. Veracruz: Xalapa, Botanical Garden, dead shrub stem, 22 Sep 1985, Ryvarden 23295 (O).

*Adustochaete rava*. Argentina. Cordoba: San Pedro, Parque Provincial Piñalito, angiosperm branch, 27 Apr 2024, Viner 2024/15* (H, SLP).

*Amphistereum leveilleanum* s. lato. Argentina. Cordoba: Arguello, Reserva Natural Urbana General San Martín, *Acacia praecox*, 12 May 2024, *Viner 2024/559** (H).

*Aporpium canescens*. Romania. Arad Co.: Bârzava, Runcu-Groşi, *Quercus petraea*, 15 Sep 2021, *Spirin 15050** (H).

*Aporpium dimidiatum*. Costa Rica. Monteverde: Santa Helena, hardwood, 29 Dec 2013, *Vlasák 1312/E3-J** (H).

*Descidia repanda*. Finland. Inari Lappi: Utsjoki, Kistuskaidi, *Betula* sp., 18 Sep 2005, Pippola 380 (H). Sompion Lappi: Sodankylä, Luosto, *Betula pubescens*, 6 Sep 2020, Spirin 14193* (H). Iceland. East Iceland: Múlasýsla, Egilsstaðir, *B. pubescens*, 8 Aug 1997, Ahti 54799 (H). Norway. Finnmark: Kárášjohka, *B. pubescens*, 31 Aug 2008, Miettinen 13385 (H). Russia. Krasnoyarsk Reg.: Ermakovskoe Dist., Sayano-Shushensky Nature Reserve, Bolshaya Golaya River, *Betula* sp., 16 Aug 2015, Malysheva (LE F-312770*). Sweden. Dalarna: Malung, Bötåberget, *B. pubescens*, 25 Aug 2024, Spirin 17503* (GB).

*Descidia thuretiana*. Finland. Uusimaa: Espoo, Kauklahti, Lasilaakso, *Ribes alpinum*, 9 Nov 2025, Spirin 18501 (H). France. Aveyron: Millau, Le Causse Noir, *Quercus pubescens*, 21 Feb 2025, Spirin 18046 (H), Ravin de Potensac, *Epilobium fleischeri*, 23 Feb 2025, Spirin 18181 (H), *Q. pubescens*, 23 Feb 2025, Spirin 18188 (H). Lozère: St.-Etienne-du-Valdonnez, Forêt du Sapet, Ruisseau de la Besse, *Fagus sylvatica*, 28 Jun 2022, Spirin 15516* (H). Russia. Krasnodar Reg.: Sochi, Krasnaya Polyana, *Quercus petraea*, 3 May 2021, Viner 2021/361* (H). Slovenia. Kranj: Njivica, *F. sylvatica*, 25 Sep 2019, Spirin 13139 (H). Spain. Basque Autonomous Community: Vizcaya, Camino de la Vaguada de Arrano, *F. sylvatica*, 19 Apr 1976, Telleria (H). Sweden. Västergötland: Mölndal, Solängen, *Ribes* sp., 21 Feb 2024, Spirin 17048 (GB); Göteborg, Botanical Garden, *Berberis amurensis*, 1 Apr 2024, Spirin 17223*, 17224 (GB), Guldheden, *Rosa* sp., 3 Mar 2024, Spirin 17055* (GB), 6 Apr 2024, Spirin 17228 (GB), *Salix caprea*, 3 Mar 2024, Spirin 17060*, 17062 (GB), Änggården, *Ulmus* sp., 7 Jul 2024, Spirin 17254 (GB). For other specimens examined, see Spirin et al. (2018).

*Eichleriella alliciens*. Argentina. Cordoba: Cordoba, Gral. Pueyrredón, near Bajada de Piedra, *Marus* sp., 13 May 2024 *Viner 2024/501** (H), Arguello, Reserva Natural Urbana General San Martín, angiosperm branch, 16 May 2024 *Viner* *2024/563** (H).

*Eichleriella macrochaeta*. Argentina. Cordoba: Arguello, Reserva Natural Urbana General San Martín, *Acacia greggii*, 12 May 2024, Viner 2024/414* (H). Misiones: Iguazu, Reserva Selva Iryapú, angiosperm branch, 5 May 2024, Viner 2024/204 (H, SLP). Switzerland. Ticino: Sementina, Boschetti, *Fraxinus excelsior*, 13 Oct 2019, Spirin 13625 (H). Zimbabwe. Manicaland: Rhodes Inyanga Nat. Park, Mtarzi Waterfall, dead wood, 17 Jan 1989, Ryvarden 26214 (O), 26258* (O, H). Mashonaland: Mt. Hampden, dead wood, 15 Jan 1989, Ryvarden 26034* (O, H). For other specimens examined, see Malysheva & Spirin (2017).

*Eichleriella macrospora*. Argentina. Cordoba: Cordoba, Arguello, Reserva Natural Urbana General San Martín, *Acacia praecox*, 12 May 2024 *Viner* *2024/667** (H).

*Eichleriella* sp. Argentina. Cordoba: La Calera Colón Department, Cam a la Laguna Azul, *Ailanthus* sp., 17 May 2024 *Viner 2024/634** (H).

*Endoperplexa septocystidiata.* Romania. Braşov Co.: Braşov, Tampa, *Fagus sylvatica*, 13 Sep 2021, *Spirin 14897** (H).

*Exidia candida*. Canada. British Columbia: McLeod Lake Dist., McKenzie Road, *Alnus tenuifolia*, 2 Jul 1969, Eriksson 12510 (GB-0162044). Nova Scotia: Cumberland Co., Parrsboro, dead angiosperm branch, 15 May 2004, Ahti 62508 (H). Yukon: Coal River Springs, *A. tenuifolia*, 19 Jul 1977, Ahti & Scotter (H). Finland. Uusimaa: Espoo, Kauklahti, Lasilaakso, *Tilia cordata*, 9 Nov 2025, Spirin 18494 (H). Russia. Khabarovsk Reg.: Solnechnyi Dist., Suluk-Makit, *Salix* sp., 17 Aug 2011, Spirin 4159* (H); Verkhnebureinskii Dist., Kyvyty, *Salix udensis*, 17 Aug 2014, Spirin 7463* (H), Sidorka, *Alnus hirsuta*, 24 Aug 2014, Spirin 7959* (H). Krasnoyarsk Reg.: Mirnoye, *Alnus sibirica*, 19 Aug 2013, Kotiranta 26315* (H). Leningrad Reg.: Podporozhie Dist., Oksozero, *Sorbus aucuparia*, 27 May 2017, Spirin 11221 (H). Sweden. Dalarna: Venjan, Lybergsgnupen, *Betula pubescens*, 23 Aug 2024, Spirin 17471 (GB). For other specimens examined, see Spirin et al. (2018).

*Exidia crenata*. Canada. Nova Scotia: Cumberland Co., Parrsboro, dead angiosperm branch, 15 May 2004, Ahti 62509 (H). USA. Massachusetts: Worcester, Broad Meadow Brook, *Quercus* sp., 28 Apr 2013, Miettinen 16073 (H). Tennessee: Sevier Co., Schoolhouse Gap, *Quercus* sp., 28 Sep 2015, Miettinen 19510* (H).

*Exidia delita*. Netherlands. Groningen: Loppensum, Dorpsbosje, deciduous wood, 30 Dec 2018, Enzlin 18-002* (GENT).

*Exidia friesiana*. Canada. Newfoundland and Labrador: Newfoundland, Gros Morne National Park, *Abies balsamea*, 11 Sep 2014, Ahti 74629* (H). Finland. Helsinki: Haltiala, *Picea abies*, 1 Nov 2020, Miettinen 24358 (H). France: Var, Toulon, Mont Faron, *Pinus halepensis*, 20 Jan 2026, Spirin 18538, 18545* (H), 21 Jan 2026, Spirin 18615 (H). Norway. Oppland: Sel, Sagåa Nat. Res., *P. abies*, 13 Sep 2016, Spirin 11067 (O). Russia. Chelyabinsk Reg.: Taganai National Park, *Abies sibirica*, 7 Aug 2002, Kotiranta 19332*** (H). Khabarovsk Reg.: Solnechnyi Dist., Igdomi, *Picea ajanensis*, 6 Aug 2014, Spirin 3895 (H), 4 Sep 2016, Spirin 10947* (H); Verkhnebureinskii Dist., Dublikan, *P. ajanensis*, 20 Aug 2014, Spirin 7660* (H). Leningrad Reg.: Boksitogorsk Dist., Vozhani, *Picea abies*, 1 Oct 2016, Spirin 11164* (H). Sweden. Västergötland: Medelplana, Hjälmsäter, Såten, *Abies alba*, 19 Dec 2024, Spirin 18006* (GB); Trollhättan, Slätthult, *P. abies*, 29 Apr 2007, J. Jeppson (GB-0107545). Öland: Böda, *A. alba*, 1 Oct 1998, Lundqvist (UPS F-015886). Dalarna: Idre, Töfsingdalen, *P. abies*, 31 Aug – 3 Sep 1980, Hallenberg 3367 (GB-0185200).

*Exidia glandulosa*. Finland. Varsinais-Suomi: Turku, Muhkuri, 26 Apr 1946, Kukkonen (H). Uusimaa: Espoo, Kauklahti, Lasilaakso, *Tilia cordata*, 9 Nov 2025, Spirin 18495 (H); Helsinki, Östersundom, *Quercus robur*, 28 Oct 2022, Spirin 16064* (H). Etelä-Häme: Hämeenlinna, Kaupunginpuisto, *Populus tremula* (?), 22 May 2021, Pisilä* (H). France. Aveyron: Millau, Le Causse Noir, *Quercus pubescens*, 21 Feb 2025, Spirin 18039*** (H), Le Pas Destrech, *Q. pubescens*, 25 Apr 2026, Spirin 18918 (H). Lozère: Mende, Forêt domaniale de Mende, Ruisseau de Rieucros, *Corylus avellana*, 27 Jun 2022, Spirin 15500* (H). Russia. Mordovia: Saransk, Dobrovolnyi, *Q. robur*, 29 Sep 2013, Ivoilov (LE F-314277*). Nizhny Novgorod Reg.: Lukoyanov Dist., Sanki, *C. avellana*, 9 Sep 2011, Spirin 4527 (H), 7 Aug 2014, Spirin 7203* (H). Novgorod Reg.: Chudovo Dist., Krasnofarfornyi, *Q. robur*, 11 Oct 2018, Bolshakov (LE F-321630*). Sweden. Småland: Nybro, Kråksmåla, Mjösgölemo, *Q. robur*, 27 Sep 2024, Elleström*** (GB). Västergötland: Gärdhem, Trollhättan, *Q. robur*, 21 Jan 2002, J. Jepsson (GB-0107550); Göteborg, Änggårdsbergen, *Q. robur*, 5 Mar 2024, Spirin 17067, 17068* (GB), Guldheden, *Q. robur*, 16 Dec 2024, Spirin 18001 (GB), 28 Dec 2024, Spirin 18030*** (GB); Medelplana, Munkängarna, *Q. robur*, 10 Mar 2025, Ahlefelt (GB), deciduous wood, 10 Mar 2025, Svensson (GB); Skepplanda, Skepplanda Kyrka, *T. cordata*, 17 May 2025, Ahlefelt* (GB). Bohuslän: Lycke, Älgön, *Q. robur*, 6 May 1972, Bohlin (GB-0185257); Resteröd, Ulvesund, *Q. robur*, 21 Dec 2024, Spirin 18028*** (GB); Ytterby, Guddehjälm, *Q. robur*, 27 Jan 1975, Eriksson (GB-0185256).

*Exidia impressa*. Russia. Khabarovsk Reg.: Verkhnebureinskii Dist., Dublikan, *Alnus fruticosa*, 19 Aug 2014, Spirin 7572* (H). Krasnoyarsk Reg.: Ermakovskoe Dist., Sayano-Shushensky Nature Reserve, Bol’shaya Golaya River, *Alnus* sp., 15 Aug 2015, Malysheva (LE F-312781*).

*Exidia japonica*. Russia. Primorie Reg.: Ussuriysk Dist., Ussuri Nat. Park, Peishula, *Fraxinus mandshurica*, 1 Aug 2018 *Kotiranta 28786** (H).

*Exidia pithya*. Austria. Niederösterreich: Rosenau am Sonntagberg, *Abies alba*, ‘m. Febr.’, Strasser (Kryptogamae Exsiccatae #941, H). Czech Republic. Prague: Petřín, *Tilia cordata*, 6 Aug 2025, Spirin 18295 (H). Finland. Helsinki: Uutela, *Picea abies*, 5 Nov 2022, Spirin 16131 (H). Etelä-Häme: Asikkala, Kirkonkylä, *Populus tremula*, 31 Oct 2010, Haikonen 28197 (H). Oulun Pohjanmaa: Muhos, Kylmälä, *Salix phylicifolia*, 11 Jun 1987, Ohenoja 6 (H). France. Aveyron: Creissels, Cascade de Creissels, *Rubus* sp., 9 May 2022, Spirin 15354* (H); Millau, Ravin de Potensac, *Amelanchier ovalis*, 24 Apr 2026, Spirin 18910 (H). Italy. Lombardy: Varese, San Gemolo, *Corylus avellana*, 14 Oct 2019, Spirin 13639* (H). Mongolia. Ulan Bator: Bogd Uul, Dzaysan, *Betula platyphylla*, 7 Jul 1972, Ahti 29081 (H). Russia. Dagestan: Tlyaratinsky Dist., Dagestansky Nat. Res., *Betula* bark, 28 May 2018, Viner 2018/25* (H). Khabarovsk Reg.: Khabarovsk Dist., Ulun, *Rhododendron dauricum*, 26 Aug 2012, Spirin 5651* (H); Solnechnyi Dist., Igdomi, *B. platyphylla*, 7 Aug 2011, Spirin 3938* (H), 2 Sep 2016, Spirin 10755* (H), Sohakh, *R. dauricum*, 15 Aug 2014, Spirin 7299* (H); Verkhnebureinskii Dist., Dublikan, *Alnus fruticosa*, 20 Aug 2014, Spirin 7673* (H), 21 Aug 2014, Spirin 7781* (H), *Sorbus sibirica*, 21 Aug 2014, Spirin 7725* (H), Hegdy, *A. fruticosa*, 22 Aug 2014, Spirin 7860* (H). Krasnodar Reg.: Adygoy, *Carpinus betulus* (?), May 2017, Stepanchikova (H). Leningrad Reg.: Podporozhie Dist., Oksozero, *Sorbus aucuparia*, with *E. candida* var. *cartilaginea*, 27 May 2017, Spirin 11222 (H). Nizhny Novgorod Reg.: Lukoyanov Dist., Razino, *P. tremula*, 5 Aug 2014, Spirin 7092* (H), *Betula pubescens*, 8 Aug 2014, Spirin 7220 (H), 22 Jul 2018, Spirin 11953* (H), *T. cordata*, 3 Aug 2017, Spirin 11350b* (H). Primorie Reg.: Khanka Dist., Komissarovo, *Exochorda serratifolia*, 13 Jun 2024, Marchuk (LE F-347690*). Slovenia. Gorenjska: Kranj, Njivica, *Fagus sylvatica*, 25 Sep 2019, Spirin 13129* (H). Sweden. Skåne: Maglehem, Blåherremölla, *Fagus sylvatica*, 1 Oct 2025, Spirin 18389 (GB). Västergötland: Göteborg, Botanical Garden, *Enkianthus campanulatus*, 1 Jun 2024, Spirin 17233* (GB), Guldheden, *B. pubescens*, 6 Apr 2024, Spirin 17227 (GB), 4 Jul 2024, Spirin 17249 (GB), *P. tremula*, 3 Mar 2024, Spirin 17057* (GB), *Salix caprea*, 3 Mar 2024, Spirin 17065* (GB), *Rosa* sp., 29 May 2024, Spirin 17232 (GB); Medelplana, Hjälmsäter, Såten, *A. alba*, 19 Dec 2024, Olsson* (GB); Mölndal, Änggårdsbergen, *C. avellana*, 1 Apr 2024, Spirin 17218* (GB), 1 Jun 2024, Spirin 17235* (GB), Snöflingegatan, *C. avellana*, 27 May 2024, Spirin 17231* (GB); Otterstad, Skalludden, *P. tremula*, 10 Nov 1996, M. Jeppson (GB-0182271); Partille, Tultered, *C. avellana*, Mar 2023, Pielach (GB). USA. Arkansas: Marion Co., Buffalo National River, dead angiosperm branch, 26 Oct 2013, Birkebak* (H).

*Exidia plumbescens*. Belgium. Oost-Vlaanderen: Zegelsem, Sighelsempad, deciduous wood, 15 Dec 2004, J. Schoutteten 04-001*** (GENT). Canada. British Columbia: Mc Leod Dist., road 44, *Populus* sp., 25 Jun 1969, Eriksson 12078 (GB-0185809). Finland. Etelä-Häme: Tammela, Mustiala, *Salix* sp., 2–4 Apr 1887, Karsten 4939, 4940, 4941 (H), *Populus tremula* (?), 2 Oct 1895, Karsten 4943 (H). Kainuu: Sotkamo, Lontanniemi, deciduous wood, 18 Oct 2021, Helo 20210767* (OULU, H). Oulun Pohjanmaa: Tyrnävä, Ängeslevänjoki, *Salix* sp., 30 Sep 2024, Kulju 68/30.9.2024 (OULU, H). Netherlands. Groningen: Bourtange, TWV forest, deciduous wood, 18 Feb 2019, Enzlin 19-013* (GENT). Norway. Troms: Storfjord, Skibotndalen, Lullesletta, 19 Aug 1992, Torkelsen (O F-295622). Russia. Kamchatka: Bystrinky Dist., Uksichan, *Salix* sp., 3 Aug 1997, Kotiranta 12843 (H). Khabarovsk Reg.: Verkhnebureinskii Dist., Sidorka, *Salix schwerinii*, 24 Aug 2014, Spirin 7958*** (H). Leningrad Reg.: Boksitogorsk Dist., Radogoshch, *Salix cinerea*, 26 Sep 2012, Spirin 5836*** (H). Primorie Reg.: Khasan Dist., Kedrovaya Pad’ Nat. Res., *Populus* sp., 25 Jul 2016, Viner 2016/356* (H). Tuva: Ush-Bel’dyr, angiosperm, 11 Aug 1972, Raitviir & Kullman (TAAM 62650). Sweden. Västergötland: Medelplana, Munkängarna, *Ulmus glabra*, 19 Dec 2024, Spirin 18015* (GB, H). Torne Lappmark: Abisko Nat. Park, *Salix* sp., 8 Aug 1981, Hallenberg 3734 (GB-0185320).

*Exidia recisa*. Finland. Uusimaa: Espoo, Kauklahti, Lasilaakso, *Populus tremula*, 9 Nov 2025, Spirin 18517 (H), *Salix caprea*, 9 Nov 2025, Spirin 18519*** (H), *Ulmus glabra*, 9 Nov 2025, Spirin 18518* (H), Leppävaara, Vermonpuisto, *S. caprea*, 1 Sep 2025, Spirin 18346* (H); Helsinki, Pihlajisto, *S. caprea*, 1 Feb 2025, Miettinen 27519* (H), Veräjämäki, *S. caprea*, 19 Oct 2011, Miettinen 14913* (H), fallen branch (*Acer* or *Salix*), 7 Feb 2025, Miettinen 27521 (H); Sipoo, Hindsby, *P. tremula*, 3 Nov 2025, Spirin 18484* (H), *Padus avium*, 3 Nov 2025, Spirin 18488* (H), *S. caprea*, 3 Nov 2025, Spirin 18489 (H); Vantaa, Tammisto, *Tilia cordata*, 30 Oct 2025, Viner 2025/36* (H). France. Isère: Séchilienne, Lac Luitel, *Salix aurita*, 24 Sep 2014, Rivoire 5695 (LY). Russia. Altai Republic: Altai Nat. Reserve, Kamga Field Station, *Salix* sp., 18 Aug 2008, Malysheva (LE F-231785, 231765). Khakassia: Abakan, Erkagi, *Salix viminalis*, 15 Aug 2011, Kotiranta 25191 (H). Nizhny Novgorod Reg.: Lukoyanov Dist., Razino, *S. caprea*, 8 Sep 2011, Spirin 4477 (H). Sweden. Småland: Värnamo, Rusarebo, *S. caprea*, 17 Oct 2024, Spirin 17725 (GB). Dalarna: Transtrand, Bredvallen, *S. caprea*, 22 Aug 2024, Spirin 17465 (GB); Malung, Lybergsgnupen, *S. caprea*, 24 Aug 2024, Spirin 17497 (GB).

*Exidia reflexa*. Russia. Primorie Reg.: Terney Dist., Sikhote-Alin Nature Reserve, Maisa, angiosperm branch, 13 Aug 2012, Malysheva (LE F-303425*).

*Exidia repleta*. France. Var: Mont Faron, *Quercus coccifera*, 20 Jan 2026, Spirin 18598*, 18608 (H).

*Exidia saturata*. France. Var: Mont Faron, *Rosmarinus officinalis*, 23 Jan 2026, Spirin 18750* (H).

*Exidia semiorbis*. Russia. Primorie Reg.: Shkotovo Dist., Ussuri Nat. Res., Peishula, *Salix* sp. (fallen branch), 16 Aug 2011, Malysheva (LE F-262977*); Terney Dist., Sikhote-Alin Nature Reserve, Suvorovka, angiosperm branch, 13 Aug 2012, Malysheva (LE F-303433*).

*Exidia spiculata*. Canada. Alberta: Yellowhead Co., William A. Switzer Provincial Park, *Salix* sp., 24 Jul 2015, Spirin 8839* (H). British Columbia: McLeod Lake Dist., Road 49, *Alnus* sp., 26 Jun 1969, Eriksson 12178 (GB-0185189). North-West Territory: Mackenzie Dist., Crossley Lakes, *Salix* sp. (?), 13 Jul 1966, Scotter 8147a (H). Quebec: Outaouais, Gatineau Nat. Park, Church Hill, *Alnus rugosa*, 21 Oct 1967, Eriksson 9186 (GB-0185188). Finland. Uusimaa: Espoo, Leppävaara, Perkkaa, *Populus tremula*, 2 Sep 2025, Spirin 18347 (H). Etelä-Häme: Hämeenlinna, *Salix caprea*, 18 Mar 2007, Alanko 132668 (H); Padasjoki, Kellosalmi, *P. tremula*, 5 May 1984, Haikonen 4295 (H); Pirkkala, Haikka, *Sorbus aucuparia*, 23 Oct 2006, Salo 10914 (H). Satakunta: Kankaanpää, Venesjärvi, *P. tremula*, 7 Jun 1935, Laurila (H6103191). Russia. Leningrad Reg.: Lodeinoye Pole Dist., Nizhnesvirsky Nat. Res., *P. tremula*, 18 Sep 1996, Zmitrovich (LE F-201999*). Zabaikalie Reg.: Verkhnii Bukukun, *Salix* sp., 9 Sep 2010, Kotiranta 29955 (H).

*Exidia straminea*. France. Aveyron: Creissels, Chemin de Brunas, *Amelanchier ovalis*, 25 Apr 2026, Spirin 18925 (H); Millau, Dourbie, *A. ovalis*, 23 Feb 2025, Spirin 18158 (H), Le Causse Noir, *A. ovalis*, 19 Jun 2025, Spirin 18228 (H), 23 Apr 2026, Spirin 18862 (H), Le Monna, *Lonicera* sp., 23 Feb 2025, Spirin 18144 (H), *Laburnum* sp., 23 Feb 2025, Spirin 18157 (H), *A. ovalis*, 24 Apr 2026, Spirin 18895, 18899 (H), Ravin de Potensac, *A. ovalis*, 23 Feb 2025, Spirin 18174*** (H), 24 Apr 2026, Spirin 18907 (H). Rhône: Chaussan, Bois de Pinloup, *Padus avium*, 8 Dec 2017, Rivoire 7046* (LY); Rontalon, Bois de la Sebri, *P. avium*, 2 Jul 2012, Rivoire 4495 (LY). Italy. Piedmont: Novara, Riva Valdobbia, *Padus avium*, 1899, Carestia (as *Exidia recisa* f. *cerasi* Bres., #610 in Saccardo’s Mycotheca Italica) (S F282950).

*Exidia truncata*. Sweden. Västergötland: Göteborg, Guldheden, *Q. robur*, 3 Mar 2024, Spirin 17059* (GB, H). Bohuslän: Resteröd, Ulvesund, *Q. robur*, 21 Dec 2024, Spirin 18022*, 18023 (GB).

*Exidia umbrinella*. Russia. Karachay-Cherkessia: Karachayevsk Dist., Teberda Natural Reserve, Khadzhibey Gorge, *Picea* sp., 8 Aug 2009, Malysheva (LE F-254078*).

*Exidia uvapassa*. Russia. Primorie Reg.: Khasan Dist., Kedrovaya Pad’ Nat. Reserve, *Quercus mongolica*, 2 Sep 2011, Malysheva (LE F-262970), old-growth mixed forest, *Betula* bark, 25 Jul 2016, Viner 2016/275* (H).

*Exidia yadongensis*. Russia. Khabarovsk Reg.: Solnechnyi Dist., Igdomi, *Padus avium*, 3 Sep 2016, Spirin 10874* (H), *Alnus hirsuta*, 3 Sep 2016, Spirin 10904* (H); Verkhnebureinskii Dist., Dublikan, *Alnus fruticosa*, 23 Aug 2014, Spirin 7953 (H), Kyvyty, *A. fruticosa*, 17 Aug 2014, Spirin 7462* (H). Primorie Reg.: Terney Dist., Sikhote-Alin Nat. Res., *A. hirsuta*, 29 Aug 2012, Malysheva (LE F-303418*).

*Exidia zelleri*. Canada. Alberta: Banff National Park, Mt. Norquay, *Salix* sp., 28 Aug 1967, Eriksson 7466 (GB-01852222). Finland. Uusimaa: Sipoo, Hindsby, *Populus tremula*, 3 Nov 2025, Spirin 18484a, 18487 (H). Etelä-Häme: Hollola, Paimela, *P. tremula*, 25 Nov 2015, Haikonen 30419 (H). Russia. Khabarovsk Reg.: Solnechnyi Dist., Igdomi, *Salix* sp., 3 Sep 2016, Spirin 10927* (H); Verkhnebureinskii Dist., Hegdy, *Salix schwerinii*, 22 Aug 2014, Spirin 7868* (H). Leningrad Reg.: Boksitogorsk Dist., Vozhani, *P. tremula*, 30 Jul 2016, Spirin 10456* (H). Murmansk Reg.: Kola, 1861, Fellman (herb. Karsten 5420, 5421) (H). Primorie Reg.: Terney Dist., Sikhote-Alin Nature Reserve, Malaya Tenevaya, *Populus* sp., 30 Aug 2012, Pimenova (LE F-303406*). Tyumen Reg.: Khanty-Mansi Autonomous Dist., *P. tremula*, 16 Jun 2011, Bulyonkova (LE F-347689*). Zabaikalie Reg.: Duldurga Dist., Alkhanay, *Salix* sp., 30 Aug 2010, Kotiranta 29841 (H). Sweden. Västergötland: Partille, Tultered, *P. tremula*, 3 Nov 2024, Spirin 17775*, 17780* (GB). Torne Lappmark: Abisko, *Alnus incana*, 18 Aug 1960, Eriksson 8937 (GB-0185237).

*Exidiopsis effusa*. France. Aveyron: Millau, Ravin de Potensac, *Quercus pubescens*, 23 Feb 2025, Spirin 18204 (H). Luxembourg. Junglinster: Eisenborn, deciduous wood, 14 Apr 1992, J. Schoutteten 92-001* (GENT). Norway. Akershus: Baerum, Kjaglidalen, *C. avellana*, 4 May 2016, Spirin 10026* (O), *Sorbus aucuparia*, 4 May 2016, Spirin 10024* (O); Asker, Stokkerelva at Åstaddammen, *Ulmus glabra*, 5 May 2016, Spirin 10032 (O). Oslo: Bygdøy, *C. avellana*, 3 May 2016, Spirin 10007 (O). Sweden. Västergötland: Göteborg, Guldheden, *Salix caprea*, 3 Mar 2024, Spirin 17061 (GB); Österplana, *Populus tremula*, 3 Mar 2025, Olsson*** (GB), Stora Salen, angiosperm wood, 21 Apr 2025, Olsson*** (GB).

*Exidiopsis griseobrunnea* ssp. *macrogyna* (paratypes, not conspecific with the type!). Russia. Yakutia: Oymyakon Dist., Tomtor, *A. fruticosa*, 3 Sep 1972, Parmasto (TAAM 56815); Verkhnekolymskii Dist., Verkhnekolymsk, *A. fruticosa*, 23 Aug 1972, Järva (TAAM 45888).

*Exidiopsis perflua*. Finland. Uusimaa: Helsinki, Pornaistenniemi, *Padus avium*, 1 May 2015, Miettinen 19136*** (H). Norway. Finnmark: Kárášjohka, *Salix* sp. (dry branch), 5 Sep 2008, Miettinen 13505.1 (H). Russia. Leningrad Reg.: Boksitogorsk Dist., Chagoda, *Alnus incana*, 9 May 2018, Spirin 11869 (H); Podporozhie Dist., Grishino, *A. incana*, 27 May 2017, Spirin 11233 (H), Vazhinka, *S. aucuparia*, 21 May 2016, Spirin 10126 (H), 21 Jun 2016, Spirin 10220 (H), 1 Jun 2018, Spirin 11913 (H), *Populus tremula*, 1 Jun 2018, Spirin 11920 (H). Slovenia. Radovljica: Krnica, Lipanca, *Alnus viridis*, 6 Jun 2019, Spirin 12632, 12635, 12638* (H), 29 Jul 2020, Spirin 13956 (H). Sweden. Gästrikland: Gävle, Lövudden, *Ribes nigrum*, 23 Apr 1973, Nannfeldt (Flora Suecica #22952) (H). Torne Lappmark: Abisko, Torneträsk, angiosperm branch, 14 Aug 1960, Eriksson 8798 (GB-0185787).

*Heteroradulum deglubens.* Sweden. Västergötland: Medelplana, Munkängarna, *Ulmus glabra*, 15 Oct 2023 *Spirin 16936** (GB).

*Leiostroma farinellum*. Finland. Uusimaa: Helsinki, Veräjämäki, *P. abies*, 25 Mar 2017, Miettinen 20585* (H). Romania. Suceava: Suceava, *Picea abies*, 13 Oct 1983, Hallenberg 7970 (GB-0185343), Poiana Stampei, *P. abies*, 17 Oct 1983, Hallenberg 8093 (GB-0185344). Russia. Khabarovsk Reg.: Khabarovsk Dist., Bolshoi Khekhtsir, *Pinus koraiensis*, 26 Aug 2014, Spirin 8083 (H); Solnechnyi Dist., Igdomi, *Picea ajanensis*, 7 Aug 2011, Spirin 3990 (H). Leningrad Reg.: Boksitogorsk Dist., Kolp’, *P. abies*, 25 Jul 2011, Spirin 3696 (H). Nizhny Novgorod Reg.: Lukoyanov Dist., Panzelka, *P. abies*, 12 Sep 2010, Spirin 3459 (H), Razino, *P. abies*, 8 Sep 2011, Spirin 4498 (H), 18 Aug 2015, Spirin 9496 (H). Slovenia. Radovljica: Krnica, Šijec, *P. abies*, 6 Jun 2019, Spirin 12597 (H). Tolmin: Vršič, *Pinus mugo*, 5 Jun 2019, Spirin 12586* (H). Sweden. Västergötland: Lidköping, Otterstad, *Pinus sylvestris*, 20 Feb 2005, Carlsson 05-153 (GB-0233824); Medelplana, Hjälmsäter, Såten, fallen corticated angiosperm branch, 13 Apr 2025, Olsson*** (GB). Åsele Lappmark: Åsele, Rödberget Nat. Reserve, *P. abies*, 28 Aug 2023, Spirin 16599* (GB).

*Leiostroma incanum*. Armenia. Syunik: Tsav, *Rhamnus cathartica*, 2 Oct 1962, Parmasto (TAAM 15314, 16216). Azerbaijan. Lerik, *Albizia julibrissin*, 11 Oct 1962, Parmasto (TAAM 15993). Portugal. Algarve: Loumé, Fonte da Benémola, *Ceratonia siliqua*, 2 Jan 2022, Miettinen 25155 (H). Russia. Krasnodar Reg.: Goryachii Klyuch, Mirnyi, *Acer tataricum*, 1 Oct 1966, Parmasto (TAAM 19829), *Carpinus betulus*, 3 Oct 1966, Parmasto (TAAM 19924). Slovenia. Sežana: Kačiče-Pared, *Laburnum* sp., 27 Sep 2023, Spirin 16722* (H, GB). Turkey. Muǧla: Marmaris, *Platanus* sp., 12 Apr 1972, Hallenberg (GB-0185796).

*Leiostroma infecundum*. Spain. Canary Ids.: Tenerife, Las Mercedes, *Persea indica*, 19 Dec 1973, Hallingbäck (GB-0185798), Las Montanas de Anaga, angiosperm wood, 10 Mar 1989, Hallenberg 11025 (GB-0185283), 13 Mar 1989, Hallenberg 11059 (GB-0185282), *Laurus azorica*, 1 Apr 1997, E. Larsson 39-97 (GB-0150240); La Palma, San Miguel de La Palma, Puntallana, angiosperm wood, 22 Jul 2022, Viner 2022/1008*, 2022/1021 (H), 26 Jul 2022 Viner 2022/1047 (H), San Andrés y Sauces, angiosperm wood, 26 Jul 2022 Viner 2022/1066 (H).

*Leiostroma squalidum*. Zimbabwe. Eastern Prov.: Mutare, Vumba Mts., White Horse Inn, decorticated angiosperm wood, 20 Feb 1986, Ryvarden 23725* (O, H), decorticated angiosperm branch, 20 Feb 1986, Ryvarden 23753 (O).

*Leiostroma* sp. 1. Canada. Alberta: Yellowhead Co., Hinton, *Picea mariana*, 24 Jul 2015, Spirin 8870, 8873* (H). North-West Territory: Mackenzie River, *Populus balsamifera*, 18 Aug 1967, Eriksson 7316* (GB).

*Leiostroma* sp. 2. France. Var: Toulon, Sente des Douaniers, *Quercus petraea*, 24 Jan 2026, Spirin 18795* (H).

*Proterochaete abstrusa*. Denmark. Sjælland: Hovedstaden, Helsinge, Ellemose, *Salix* sp., 6 Nov 2014, Læssøe DMS-695676* (C). Syddanmark: Åbenrå, Tinglev, Ulvemose, dead corticated branch, 20 Aug 2012, Heilmann-Clausen DMS-456745* (C). France. Var: Toulon, Mont Faron, *Quercus coccifera*, 21 Jan 2026, Spirin 18652 (H). Aveyron: Millau, Le Pas Destrech, *Quercus pubescens*, 25 Apr 2026, Spirin 18917 (H), Ravin de Potensac, *Helichrysum* sp., 8 Nov 2024, Spirin 17946* (H). Isère: Le Cheylas, La Rolande et Maupas, *Cornus sanguinea* (?), 8 Oct 2018, Rivoire 7250 (LY, H). Lozère: Mende, Ruisseau de Rieucros, *Fraxinus excelsior*, 27 Jun 2022, Spirin 15484 (H). Italy. Liguria: Imperia, Pigna, Buggio, *Carpinus betulus*, 17 Oct 2019, Spirin 13822 (H), Schoutteten 19-316* (GENT). Norway. Hedmark: Ringsaker, Kløvstadhøvda, *Salix caprea*, 7 Oct 2025, Spirin 18430 (O, H). Slovenia. Radovljica: Ravne v Bohinju, Orožnova Koča, *Salix* sp. (fallen corticated branch), 28 Jul 2020, Spirin 13943*** (H).

*Proterochaete adusta*. Belgium. Antwerpen: Mechelen, Kauwdaalbos, *Populus* sp., 26 Feb 2020, Van Autgaerden 20-048* (GENT); St. Kat. Waver, Goorbosbeekvallei, *Populus* sp., 12 Mar 2020, Van Autgaerden 20-067* (GENT). Limburg: Boekhout, domain Den Turck, *Populus* sp., 6 Feb 2020, Van Autgaerden 20-041* (GENT). Finland. Kainuu: Kajaani, Patjamäki, *Populus tremula*, 31 Oct 2021, Helo 20211019, 20211022 (OULU); Kuhmo, Riihivaara, *P. tremula*, 26 Aug 2017, Julkunen 1062* (OULU, H). Netherlands. Groningen: Wehe-den Hoorn, Starkenborgh, deciduous wood, 21 Mar 2020, Enzlin 20-015* (GENT), 18 Aug 2021, Enzlin 21-039*, 21-043* (GENT). Utrecht: Utrecht, Nieuw Wulven bij Houten, Speelbos, *Populus* sp., 26 Jan 2020, Bruggeman-Nannenga 7148* (GENT). For other specimens examined, see Alvarenga et al. (2019).

*Proterochaete furfurosa*. Uganda. Kabarole: Kibale National Park, Makere University Field Station, fallen corticated branch, 20 Apr 2002, Ryvarden 44939* (O). Zimbabwe. Manicaland: J. Meikele Research Station, fallen decorticated branch, 24 Jan 1994, Ryvarden 33459* (O).

*Proterochaete letendreana*. Armenia. Syunik: Tsav, *Juglans regia* and *Malus orientalis*, 2 Oct 1962, Parmasto (TAAM 16217, 16220). Tavush: Getashen, *Acer campestre*, 26 Sep 1962, Parmasto (TAAM 15405). Austria. Niederösterreich: Perchtoldsdorf, Föhrenberge Nat. Park, *Fagus sylvatica*, 10 Sep 2022, Spirin 15776 (H, W), *Fraxinus excelsior*, 10 Sep 2022, Spirin 15798 (H, W), *Lonicera* sp., 10 Sep 2022, Spirin 15808*** (H, W). Azerbaijan. Zagatala: Zaqatala, *Tilia platyphyllos*, 12 Aug 1974, Puusepp (TAAM 90800). Belgium. Antwerpen: Duffel, Neteland, *Salix* sp., 20 Feb 2020, Van Autgaerden 20-052* (GENT); Putte, Peultenbossen, deciduous wood, 17 Jan 2019, Van Autgaerden 19-005* (GENT); Mechelen, Kauwendaalbos, deciduous wood, X.2020, Van Autgaerden 19-008*, 19-009* (GENT). Namur: Philippeville, Viroinval, *Corylus avellana*, 11 Oct 2019, Spirin 13495 (H), *F. excelsior*, 11 Oct 2019, Spirin 13516 (H), *Salix caprea*, 11 Oct 2019, Spirin 13512, 13519 (H). Oost-Vlaanderen: Ronse, deciduous wood, 28 Dec 1997, J. Schoutteten 97-164* (GENT); Steenhuize, deciduous wood, 17 Feb 2007, J. Schoutteten 07-004* (GENT); Zegelsem, Burreken, deciduous wood, 1 Feb 2018, J. Schoutteten 18-003* (GENT). Vlaams Braband: Zemst, Vriezenbroek, deciduous wood, 13 Jan 2019, Van Autgaerden 19-011* (GENT). West-Vlaanderen: Wingene, deciduous wood, 24 Oct 2016, Schoutteten 16-027* (GENT). Czech Republic. Prague: Šárka – Lysolaje Nat. Park, *A. campestre*, 7 Aug 2025, Spirin 18304 (H); Košíře-Motol Nat. Park, *F. excelsior*, 8 Aug 2025, Spirin 18332 (H), *Robinia pseudoacacia*, 8 Aug 2025, Spirin 18334 (H). Denmark. Jylland: Syddanmark, Tinglev, Ulvemose, *Salix cinerea*, 20 Aug 2012, Heilmann-Clausen DMS-456746 (C). Sjælland: Hovedstaden, Hillerød, Strødam, *Tilia cordata*, 4 Nov 2014, Læssøe DMS-695051 (C); Sjælland, Lejre, Hejede Overdrev, Smuldmosen, *Salix* sp., 2 Nov 2014, Læssøe DMS-694321 & Smidth (C). Finland. Uusimaa: Espoo, Vermonpuisto, *Populus tremula*, 1 Sep 2025, Spirin 18345 (H); Helsinki, Östersundom, Stora Dammen, *S. caprea*, 31 Oct 2022, Spirin 16079 (H), Vuosaari, Fallbacka, *P. tremula*, 4 Nov 2022, Spirin 16116 (H). France. Var: Toulon, Mont Faron, *Quercus coccifera*, 20–21 Jan 2026, Spirin 18599, 18674 (H). Aveyron: Creissels, Cascade de Creissels, *Acer pseudoplatanus*, 16 Nov 2022, Spirin 16476 (H), *Clematis vitalba*, 21 Jun 2025, Spirin 18269, 18283 (H), *F. excelsior*, 16 Nov 2022, Spirin 16470* (H), 21 Jun 2025, Spirin 18271 (H), *Populus nigra*, 7 Nov 2024, Spirin 17866* (H), *Rubus* sp., 21 Jun 2025, Spirin 18265 (H); Millau, Larzac, Le Rajal del Gorp, *Amelanchier ovalis*, 22 Feb 2025, Spirin 18104 (H), Le Causse Noir, *Quercus pubescens*, 8 May 2022, Spirin 15313 (H), 13 Nov 2022, Spirin 16172*** (H), *Thymus* sp., 22 Mar 2024, Spirin 17157* (H), Dourbie, *F. excelsior*, 10 May 2022, Spirin 15402 (H), 20 Jun 2025, Spirin 18245, 18252 (H), *Lavandula* sp., 15 Nov 2022, Spirin 16434 (H), *Populus* sp., 15 Nov 2022, Spirin 16370 (H), *Salix alba*, 20 Jun 2025, Spirin 18237 (H), *Sambucus nigra*, 20 Jun 2025, Spirin 18238, 18240 (H), *Ulmus* sp., 20 Jun 2025, Spirin 18234, 18241 (H), Ravin de Potensac, *Helichrysum* sp., 15 Nov 2022, Spirin 16439 (H), 23 Feb 2025 Spirin 18184, 18186 (H), *Epilobium fleischeri*, 23 Feb 2025, Spirin 18182 (H), Le Monna, *Lavandula* sp., 23 Feb 2025, Spirin 18146 (H). Lozère: Mende, Ruisseau de Rieucros, *F. excelsior*, 27 Jun 2022, Spirin 15493 (H); Aubrac, Les Salces, *Cytisus oromediterraneus*, 30 Jun 2022, Spirin 15652*, 15661, 15667, 15671 (H); Saint-Germain-du-Teil, *Lavandula* sp., 30 Jun 2022, Spirin 15764*, 15765, 15768 (H). Vendée: Saint-Jean-de-Monts, angiosperm wood, 26 Jan 2007, Jeppson (GB-0107644). Germany. Nordrhein-Westfalen: Bielefeld, Ochsenburg, *F. sylvatica* (?), 25 Nov 2015, Miettinen 19843.1 (H), *Quercus robur* (?), 25 Nov 2015, Miettinen 19846 (H). Greece. Athens: Athens National Garden, *Citrus reticulata*, 18 Dec 2025, Viner 2025/363 (H), Kaisariani, *Casuarina* sp., 10 Jan 2022, Viner 2202/17 (H). Italy. Lazio: Latina, Circeo Nat. Park, *Rubus* sp., 22 Oct 1984, Ryvarden 22467 (O). Liguria: Imperia, Pigna, Buggio, *Arbutus unedo*, 17 Oct 2019, Spirin 13826 (H). Veneto: Venice, Eraclea, *Robinia* sp., 13 Nov 1982, Bernicchia 1147 (GB). Netherlands. Gelderland: Vorten, Hackfort, *Populus* sp., 13 Jan 2019, Enzlin 19-001* (GENT). Groningen: Finsterswolde, *T. cordata*, 18 Nov 2018, Enzlin 18-059* (GENT); Kropswolde, deciduous wood, 27 Sep 2018, Enzlin 18-102* (GENT). Overijssel: Heino, Ansepad, fallen angiosperm branch, 23 Aug 2019, Bruggeman-Nannenga 6137* (GENT). Utrecht: Houten, Nieuw Wulven, fallen branch, 6 Aug 2019, Bruggeman-Nannenga 6091* (GENT). Norway. Rogaland: Hjelmeland, Fisterfjellet Nat. Res., decorticated angiosperm branch, 16 Oct 1998, Hjortstam 17987 (GB). Vest-Agder: Lyngdal, Fladstad, *T. cordata*, 1 Nov 2017, Spirin 11749, 11762 (O). Akershus: Asker, Esvika, *T. cordata*, 30 Sep 2017, Spirin 11676 (O), 28 Sep 2018, Spirin 12475 (O), *Ulmus* sp., 30 Sep 2017, Spirin 11668 (O), Stokkerelva at Åstaddammen, *Ulmus* sp., 28 Sep 2018, Spirin 12436 (O). Oslo: Bygdøy, *T. cordata*, 30 Sep 2017, Spirin 11682, 11684, 11685, 11687 (O). Sør-Trøndelag: Oppdal, Kongsvoll, *Salix lanata*, 19 Aug 1980, Hjortstam 11545 (GB-0185694). Romania. Bistrița-Năsăud: Bistrița, *R. pseudoacacia*, 12 Oct 1983, Hallenberg 7936 (GB-0185342). Russia. Dagestan: Magaramkent, Samur Nat. Res., *Crataegus kyrtostyla*, 17 Sep 1973, Parmasto (TAAM 58123), 18 Sep 1977 (TAAM 58273). Krasnodar Reg.: Gelendzhik, Arkhipo-Osipovka, *Quercus petraea*, 24 Sep 1966, Parmasto (TAAM 18803); Goryachiy Klyuch, Pyatigorskaya, *Pyrus caucasica*, 1 Oct 1966, Parmasto (TAAM 19854); Slavyansk Dist., Krasnyi Les, *Crataegus* sp. and *R. pseudoacacia*, 27 Sep 1966, Parmasto (TAAM 19723, 19741), dry branch, 28 Sep 1966, Parmasto (TAAM 19791). Nizhny Novgorod Reg.: Lukoyanov Dist., Sanki, *Betula pubescens*, 21 Jul 2018, Spirin 11948*** (H). Slovakia. Banská Bystrica: Murán, dead wood, 5 Oct 2008 E. Larsson 202-08 (GB). Slovenia. Cerknica: Goričice, *C. avellana*, 30 Sep 2023, Spirin 16856 (H), *A. campestre*, 30 Sep 2023, Spirin 16860* (H). Postojna: Grobišče, *S. nigra*, 29 Sep 2023, Spirin 16793 (H), *R. pseudoacacia*, 29 Sep 2023, Spirin 16809 (H); Slovenska Vas, *Prunus spinosa*, 26 Sep 2023, Spirin 16675 (H). Sežana: Divača, *Ostrya carpinifolia*, 27 Sep 2023, Spirin 16716 (H). Sweden. Skåne: Kiaby, Kjugekull, *Ulmus glabra*, 3 Oct 2025, Spirin 18407 (GB), *F. excelsior*, 3 Oct 2025, Spirin 18409 (GB); Maglehem, Blåherremölla, *F. sylvatica*, 1 Oct 2025, Spirin 18392 (GB); Simrishamn, Södra Mellby, *Carpinus betulus*, 22 Oct 1975, Jakobsson & Eriksson (GB-0185738). Västergötland: Alingsås, Fagerlid, *Ribes uva-crispa*, 16 Dec 1972, Hjortstam 6190 (GB); Göteborg, Botanical Garden, *Lonicera tatarica*, 7 Dec 1940, Nathorst-Windahl 2442 (GB-0185616), *Philadelphus coronarius*, 1 Apr 2024, Spirin 17219, 17222 (GB); Götene, Törnsäter, angiosperm wood, 19 Dec 2023, Olsson (GB); Kinnekulle, Råbäck, deciduous branches, 30 Oct 1976, Hjortstam 7441 (GB-0185774), 30 Oct 2006, Hjortstam 19346, 19375 (GB), Österplana, *Laburnum* sp., 25 Mar 2024, Olsson (GB), *Prunus* sp., 25 Mar 2024, Olsson (GB), *Salix* sp., 22 Feb 2025, Olsson (GB); Mölndal, Solängen, *A. campestre*, 26 May 2024, Spirin 17230 (GB), *Ribes* sp., 21 Feb 2024, Spirin 17050, 17051*** (GB), *Syringa* sp., 28 Feb 2024, Spirin 17054*** (GB); Skepplanda, Frövet, *U. glabra*, 11 Oct 2025, Spirin 18456 (GB, H); Västra Tunhem, Hunneberg, *Q. robur*, 16 Oct 1982, Larsson 3511 (GB), *Ulmus* sp., 16 Oct 1982, Larsson 3518 (GB). Bohuslän: Hönö, Ersdalsvägen, *Cytisus scoparius*, 12 Nov 2023, Spirin 17041, 17045* (GB), 15 Jul 2024, Spirin 17312, 17324 (GB), *P. tremula*, 15 Jul 2024, Spirin 17309 (GB), 12 Aug 2024, Spirin 17361 (GB). Öland: Borgholm, deciduous branch, 22 Oct 1967, Hjortstam (GB-0185685). Turkey. İzmir: Davutlar, dry twigs, 17 May 1983, Murto 1348 (H). Ukraine. Chernihiv Reg.: Korop Dist., Vyshenky, *Acer negundo*, 5 Aug 2020, Akulov (CWU (MYC) AB694*).

*Proterochaete macrogyna*. Russia. Yakutia: Ĥaŋalas Dist., Buotama, *Alnus fruticosa*, 18 Aug 2016, Kotiranta 27629 (H); Verkhoyanskii Dist., Verkhoyansk, *A. fruticosa*, 10 Aug 2016, Kotiranta 27405, 27406, 27410* (H).

*Proterochaete mucedinea*. Argentina. Misiones: Iguazu, Reserva Selva Iryapú, angiosperm branch, 5 May 2024, Viner 2024/205* (H, SLP), Hipólito Yrigoyen, angiosperm stump, 5 May 2024, Viner 2024/233* (H, SLP).

*Proterochaete paniculata*. Canada. British Columbia: Vancouver, University of British Columbia Campus, *Robinia pseudoacacia*, 8 Apr 1984, Bandoni 7310A*** (BPI 871730, paratype). USA. Washington: Pend Oreille Co., Slate Creek, *Ceanothus sanguinea*, 15 Oct 2014, Spirin 8572 (H), *Philadelphus lewisii*, 15 Oct 2014, Spirin 8545, Spirin 8549*, 8560a (H).

*Proterochaete* aff. *stipidosa*. Mexico. Veracruz: Barranca de Psecado, dead corticated branch, 26 Sep 1985, Ryvarden 23471 (O). Tamaulipas: Cuidad Victoria, corticated branch, 22 Aug 1961, Krug (TAAM 192049 ex TRTC 37230).

*Scrupulispora perparvula*. Austria. Niederösterreich: Perchtoldsdorf, Perchtoldsdorfer Heide, *Crataegus* sp., 10 Sep 2022, Spirin 15819a* (H, W).

*Tegmenticium peritrichum*. Austria. Steiermark: Hieflau, Unteres Schwabeltal, *Corylus avellana*, 17–20 Sep 2020, Michelitsch*** (GJO); Admont, Johnsbachtal, *Fraxinus excelsior*, 17–20 Sep 2020, Michelitsch*** (GJO). Finland. Varsinais-Suomi: Lohja, Maila, *Ulmus glabra*, 2 Oct 1937, Laurila (H6049691), Torhola, *C. avellana*, 14 Nov 2025, Spirin 18537*** (H), *U. glabra*, 17 Oct 2003, Kotiranta 20048 (H). France. Aveyron: Boutes, *Pyrus* sp., 27 Jun 1910, Galzin 6434 (herb. Bourdot 8344) (PC); Brusque, Bois de Saint-Thomas, *Crataegus* and *Rosa* sp., May 1907, Galzin 2107 (herb. Bourdot 4628), 3 Jun 1907, Galzin 2106 (herb. Bourdot 4628), *Sorbus aria*, Sep 1910, Galzin 6973 (herb. Bourdot 8349), Galzin 6985 (herb. Bourdot 8346) (PC); Combret, Bétirac, *Acer campestre*, 9 Oct 1913, Galzin 13973 (herb. Bourdot 12440) (PC); La Bastide-Pradines, *S. aria*, 5 May 1913, Galzin 13190 (herb. Bourdot 20139); Millau, Massebiau, *Pyrus* sp., 18 Apr 1907, Galzin 2011 (herb. Bourdot 4627), 17 Nov 1909 Galzin 5073 (herb. Bourdot 8343) (PC). Isère: Le Bourg d’Oisans, *Ribes* sp., 9 Jul 2018, Rivoire 7162*** (LY, H). Meuse: Void-Vacon, Void, *Salix caprea*, 14 Aug 1916, Maire 35 (herb. Bourdot 17975) (PC). Russia. Karachay-Cherkessia: Zelenchuk Dist., Teberda, *Ribes* sp., 24 Sep 1968, Parmasto (TAAM 53237). Sweden. Västergötland: Falköping, Slöta, Ålleberg, *Fraxinus excelsior*, 24 Sep 1969, Hjortstam 2516 (GB-0185673). Uppland: Solna, Ulriksdal, 5 Jun 1910, Romell 4501 (S F350684); Stockholm, Skuggan, *Syringa* sp., 3 May 1893, Romell 2459 (S F350675).

*Ulocolla corticioides*. France. Aveyron: Creissels, Cascade de Creissels, *Crataegus* sp., 25 Apr 2026, Spirin 18928* (H); Millau, Le Pas Destrech, *Crataegus* sp., 25 Apr 2026, Spirin 18923* (H). Greece. Attica: Acharnes, Mt. Parnitha, *Arbutus andrachne*, 15 Dec 2025, Viner 2025/94A* (H). Morocco. Fez-Meknès: Azrou, Azrou Forest, *Quercus ilex*, 3 Nov 1951 Malençon 1955 (MPU898047), Jaaba Forest, *Quercus* sp., 4 Oct 1995 Uotila 40874* (H); Ifrane, Michlifen, *Q. ilex*, 1 Nov 1953 Malençon 2585 (MPU898048), 8 May 1954 Malençon 2673, 2674 (MPU898046, MPU898045). Sweden. Öland: Mörbylånga, Bårby borg, angiosperm wood, 15 Apr 1967, Hjortstam (GB-0185682); Resmo, Landborgen, *Crataegus* sp., 14 May 1972 J. Jeppson 273 (GB-0185684).

*Ulocolla grisea*. Austria. Steiermark: Grazer Bergland, Schöckelkreuz, *Abies alba*, 1 Nov 1981, Hallenberg (GB-0185322). France. Lozère: Saint-Etienne-du-Valdonnez, Forêt du Sapet, *Pinus sylvestris*, 29 Jun 2022, Spirin 15604*** (H). Haut-Rhin: Ligsdorf, Glaserberg, *A. alba*, 29 Jan 1983, Rastetter (GB-0185323). Russia. Krasnodar Reg.: Mostovskoy Dist., Umpyr, *Abies nordmanniana*, 11 Sep 1991, Hallenberg 12173 (GB-0185202). Spain. Andalusia: Cádiz, Grazalema, *Abies pinsapo*, 26 Apr 2026, Viner 2026/58* (H). Sweden. Östergötland: Väversunda, Omberg, *Abies* sp., 30 Oct 1978, Hallingbäck (GB-0185778). Turkey. Antalya: Giglikara Ormani, *Cedrus libani*, 11 Apr 1972, Hallenberg (GB-0185797).

*Ulocolla griseobrunnea*. Canada. Nova Scotia: Queens Co., Kejimkujik Nat. Park, *Alnus crispa*, 9 May 1999, Ahti 57260 (H). Russia. Khabarovsk Reg.: Verkhnebureinskii Dist., Dublikan, *Alnus fruticosa*, 20 Aug 2014, Spirin 7674* (H). Magadan Reg.: Magadan, Arman, *A. fruticosa*, 11 Aug 1995, Kotiranta 12162 (H). Tyumen Reg.: Yamal-Nenets Autonomous Dist., Jangana, *Alnus* sp., 26 Aug 1996, Kotiranta 12741 (H). For other specimens, see Malysheva and Spirin (2017).

*Ulocolla orbiculata*. Russia. Khabarovsk Reg.: Verkhnebureinskii Dist., Kyvyty, *Abies nephrolepis*, 17 Aug 2014, Spirin 7353 (H).

*Ulocolla pinicola*. Canada. Alberta: Yellowhead Co., Whitehorse Wildland National Park, *Picea glauca*, 26 Jul 2015, Spirin 8942 (H). British Columbia: Fraser – Fort George Reg. Dist., Mt. Robson Provincial Park, *Pinus contorta*, 25 Jul 2015, Spirin 8906 (H), *Picea* sp., 25 Jul 2015, Spirin 8916* (H); McLeod Lake Dist., McKenzie Road, *Pinus* sp., 2 Jul 1969, Eriksson 12503 (GB-0185247). North-West Territory: Mackenzie Dist., Nahanni Nat. Park, *P. contorta*, 12 Jul 1977, Ahti 32804 (H).

*Ulocolla saccharina*. Denmark. Bornholm: Dueodde, *Pinus sylvestris*, 17 Oct 1960, Bjørnekær (H ex C). Finland. Uusimaa: Helsinki, Käärmeluoto, *P. sylvestris*, 9 Aug 2008, Miettinen 13223.1, 13225 (H). France. Hérault: Saint-Guilhem-le-Désert, *Pinus nigra*, 20 Jun 2011, Miettinen 14475.3 (H). Aveyron: Millau, Le Causse Noir, *P. nigra*, 21 Feb 2025, Spirin 18052 (H), Ravin de Potensac, *P. sylvestris*, 23 Feb 2025, Spirin 18187* (H). Greece. Athens: Hymettus, *Pinus* sp., 19 Dec 2025, Viner 2025/129 (H). Italy. Piedmont: Alessandria, Voltaggio, Capanne di Marcarolo, *Pinus cembra*, 16 Oct 2019, Spirin 13748*** (H). Sweden. Halland: Harplinge, Harvedal, *P. sylvestris*, 28 Sep 2025, Spirin 18374 (GB). Uppland: Uppsala, Kungsparken, *P. sylvestris*, Aug 1853, E. Fries (ex herb. E. Hisinger, H).

*Ulocolla subrepanda*. Finland. Uusimaa: Kirkkonummi, Sundsberg, *Pinus sylvestris*, 20 Oct 2012, Miettinen 15787* (H); Helsinki, Uutela, *Picea abies*, 5 Nov 2022, Spirin 16132* (H). Norway. Finnmark: Kárášjohka, *P. sylvestris*, 3 Sep 2008, Miettinen 13486.1* (H), 5 Sep 2008, Miettinen 13509 (H). Russia. Khabarovsk Reg.: Solnechnyi Dist., Gorin, *Picea ajanensis*, 13 Aug 2011, Spirin 4134 (H), Suluk-Makit, *Larix gmelinii*, 17 Aug 2011, Spirin 4185 (H). Leningrad Reg.: Podporozhie Dist., Vazhinka, *P. abies*, 5 Oct 2013, Spirin 6841* (H). Sweden. Småland: Rumskulla, Norra Kvill Nat. Park, *Pinus sylvestris*, 14 Oct 1970, Eriksson (GB-0185238). Västergötland: Daretorp, Blåhult, *P. sylvestris*, 29 Sep 1974, M. & J. Jeppson 920 (GB-0185240); Odensåker, Bosgården, *Pinus sylvestris*, 2 May 1970, Neuendorf (GB-0185242). Dalarna: Malung, Bötåberget, *Picea abies*, 25 Aug 2024, Spirin 17504 (GB). Ångermanland: Junsele, Kortinghöjden, *P. sylvestris*, 22 Sep 1970, Hjortstam 4382 (GB-0185245); Nordmaling, Långrumpskogen, *P. abies*, 30 Aug 2023, Spirin 16629* (GB).
