# Supplementary material for: Exidia and Exidiopsis (Auriculariales, Basidiomycota): a reconsideration
Source: IMA Fungus. 2026 Jul 29;17:e189042. doi: 10.3897/imafungus.17.189042 (PMC13444229; doi:10.3897/imafungus.17.189042)
Supplement: Supplementary file 4 [file imafungus-17-e189042-s002.doc]

Supplementary material 2

DNA sequences used in the present study

| Species | Specimen | Origin  (ISO 3166) | GenBank / UNITE accession number | | | | | Source |
| --- | --- | --- | --- | --- | --- | --- | --- | --- |
|  |  |  | ITS | LSU | *RPB1* | *RPB2* | *TEF1* |  |
| *Adustochaete albomarginata* | CLZhao 22774 | CN | PP852049 | PP849033 |  |  |  | Dong et al. (2024) |
| *Adustochaete fumida* | Ryvarden 23591 (O) | ZW | PZ630694 |  |  |  |  | this study |
| *Adustochaete gloeoapicata* | Ryvarden 23269 (O) | MX-VC | PZ630603 | PZ630603 |  |  |  | this study |
| *Adustochaete hiemalis* | Spirin 15471 (H) | FR | PZ630597 | PZ630597 | PZ599898 | PZ621135 |  | this study |
| *A. hiemalis* | Spirin 15766 (H) | FR | PZ630598 |  |  |  |  | this study |
| *A. hiemalis* | Spirin 17997 (GB) | SE | PZ630599 |  |  |  |  | this study |
| *Adustochaete interrupta* | Ryvarden 23435 (O) | MX | NR_169690 | MK391527 |  |  |  | Alvarenga et al. (2019); GenBank |
| *Adustochaete minuta* | Viner 2024/362 (H) | AR | PZ630600 | PZ630600 | PZ599899 | PZ621116 |  | this study |
| *Adustochaete nivea* | RLMA 531 | BR | MN165954 | MN165989 |  |  |  | Hyde et al. (2020) |
| *A. nivea* | Viner 2024/316 (H) | AR | PZ630601 | PZ630601 | PZ599900 | PZ621117 |  | this study |
| *Adustochaete punctata* | CLZhao 29710 | CN | PP852056 | PP849038 |  |  |  | Dong et al. (2024) |
| *A. punctata* | CLZhao 29711 | CN | PP852057 | PP849039 |  |  |  | Dong et al. (2024) |
| *A. punctata* | CLZhao 29685 | CN | PP852053 | PP849036 |  |  |  | Dong et al. (2024) |
| *A. punctata* | CLZhao 29671 | CN | PP852051 | PP849034 |  |  |  | Dong et al. (2024) |
| *A. punctata* | CLZhao 29675 | CN | PP852052 | PP849035 |  |  |  | Dong et al. (2024) |
| *A. punctata* | CLZhao 29686 | CN | PP852054 | PP849037 |  |  |  | Dong et al. (2024) |
| *A. punctata* | CLZhao 29706 | CN | PP852055 |  |  |  |  | Dong et al. (2024) |
| *A. punctata* | CLZhao 29669 | CN | PP852050 |  |  |  |  | Dong et al. (2024) |
| *Adustochaete rava* | URM 92997 | BR | NR_177482 | MK391526 |  |  |  | Alvarenga et al. (2019); GenBank |
| *A. rava* | Viner 2024/15 (H) | AR | PZ630602 | PZ630602 | PZ599901 | PZ621130 |  | this study |
| *Adustochaete scalena* | Ryvarden 26006 (O) | ZW | PZ630738 |  |  |  |  | this study |
| *Adustochaete tribulata* | Ryvarden 44803 (O) | UG | PZ630628 | PZ630628 |  |  |  | this study |
| *Adustochaete yunnanensis* | CLZhao 4401 | CN | MZ911966 | MZ950630 |  |  |  | Li & Zhao (2022) |
| *A. yunnanensis* | CLZhao 8212 | CN | MZ911964 | MZ950629 |  |  |  | Li & Zhao (2022) |
| *A. yunnanensis* | CLZhao 4671 | CN | MZ911965 |  |  |  |  | Li & Zhao (2022) |
| *A. yunnanensis* | CLZhao 35784 | CN | PQ197728 |  |  |  |  | GenBank |
| *Alloexidiopsis schistacea* | LWZ20200819-21a | CN | OM801939 | OM801932 |  |  |  | Liu et al. (2022) |
| *Amphistereum leveilleanum* s.l. | Viner 2024/559 (H) | AR | PZ630613 | PZ630613 | PZ599902 | PZ621118 |  | this study |
| *Aporpium canescens* | Spirin 15050 (H) | RO | PZ630691 | PZ630691 | PZ599903 | PZ621139 |  | this study |
| *Aporpium dimidiatum* | Vlasák 1312/E3J (H) | US-VI | KT156683 | PZ516000 | PZ525026 | PZ538047 |  | Vlasák et al. (2016); this study |
| *Auricularia asiatica* | Dai 16224 | CN | KX022011 | KX022042 | MZ753953 | MZ740045 |  | Wu et al. (2021) |
| *Auricularia cornea* | Dai 13621 | CN | MZ618936 | MZ669905 | MZ753960 | MZ740052 |  | Wu et al. (2021) |
| *Auricularia sinodelicata* | Dai 15083 | CN | MZ618940 | MZ669910 | MZ753976 | MZ740057 |  | Wu et al. (2021) |
| *Crystallodon subgelatinosum* | RC1609 | BR | MN475884 | MN475888 |  |  |  | Alvarenga & Gibertoni (2021) |
| *Descidia repanda* | Spirin 14193 (H) | FI | PZ630565 |  |  |  |  | this study |
| *D. repanda* | LE F-312770 | RU-KRA | PZ013381 | PZ013395 |  |  |  | this study |
| *D. repanda* | Spirin 17503 (GB) | SE | PZ630566 | PZ630566 | PZ599904 | PZ621136 |  | this study |
| *Descidia thuretiana* | Spirin 15516 (H) | FR | PZ630562 |  |  |  |  | this study |
| *D. thuretiana* | Viner 2021/361 (H) | RU-KDR | PZ630567 | PZ630567 | PZ599905 | PZ621142 |  | this study |
| *D. thuretiana* | Spirin 17055 (GB) | SE | PZ630568 |  |  |  |  | this study |
| *D. thuretiana* | Spirin 17060 (GB) | SE | PZ630563 |  |  |  |  | this study |
| *D. thuretiana* | Spirin 17223 (GB) | SE | PZ630564 |  |  |  |  | this study |
| *Eichleriella alliciens* | Viner 2024/501 (H) | AR | PZ630595 | PZ630595 |  | PZ621127 |  | this study |
| *E. alliciens* | Viner 2024/563 (H) | AR | PZ630596 |  |  |  |  | this study |
| *Eichleriella macrochaeta* | Ryvarden 26034 (O) | ZW | PZ630736 |  |  |  |  | this study |
| *E. macrochaeta* | Ryvarden 26258 (O) | ZW | PZ630737 |  |  |  |  | this study |
| *E. macrochaeta* | Viner 2024/414 (H) | AR | PZ630605 |  |  | PZ621128 |  | this study |
| *Eichleriella macrospora* | Viner 2024/667 (H) | AR | PZ630604 | PZ630604 | PZ599906 | PZ621129 |  | this study |
| *Eichleriella* sp. | Viner 2024/572 (H) | AR | PZ630614 | PZ630614 | PZ599907 | PZ621110 |  | this study |
| *Elmericium spinosum* | Ryvarden 30580 (O) | CM | PZ630692 | PZ630692 |  |  |  | this study |
| *Elmerina cladophora* | Miettinen 14314 (H) | ID | MG757509 | MG757509 |  |  |  | Malysheva et al. (2018) |
| *Elmerina sclerodontia* | Miettinen 16431 (H) | MY | MG757512 | MG757512 |  |  |  | Malysheva et al. (2018) |
| *Endoperplexa septocystidiata* | Spirin 14897 (H) | RO | PZ100084 | PZ100084 |  | PZ240080 |  | Spirin et al. (2026b) |
| *Exidia candida* | Spirin 4159 (H) | RU-KHA | PZ630622 |  |  |  |  | this study |
| *E. candida* | Spirin 7463 (H) | RU-KHA | PZ630621 | PZ630621 | PZ599908 | PZ621107 |  | this study |
| *E. candida* | Spirin 7959 (H) | RU-KHA | PZ630623 |  |  |  |  | this study |
| *E. candida* | Kotiranta 26315 (H) | RU-KRA | PZ630624 |  |  |  |  | this study |
| *Exidia ciliata* | SP467241 | BR | MK391523 | MK391529 |  |  |  | Alvarenga et al. (2019) |
| *Exidia cinnamomescens* | TAAM 5995 | RU-KO | UDB07686184 |  |  |  |  | this study |
| *Exidia crenata* | Dai 19156 | CA-ON | MT663358 |  |  |  |  | Wu et al. (2020) |
| *E. crenata* | Matheny 2527 | US | DQ241774 | AY700191 |  |  |  | GenBank |
| *E. crenata* | iNAT37567171 | US-IN | ON561747 |  |  |  |  | GenBank |
| *E. crenata* | Knapp WCC-20201113-05 | US-GA | OL629082 |  |  |  |  | GenBank |
| *E. crenata* | UBR16-A11-ITS1_A21 | US-AR | MN752653 |  |  |  |  | Alshammari & Stephenson (2018) |
| *E. crenata* | UBR16-A12-ITS4_A23 | US-AR | MN752654 |  |  |  |  | Alshammari & Stephenson (2018) |
| *E. crenata* | iNAT107426341 | US-AL | ON102035 |  |  |  |  | GenBank |
| *E. crenata* | Dirks ACD0002 | US-MA | MT913602 |  |  |  |  | GenBank |
| *E. crenata* | iNAT187125930 | US-OH | PV708685 |  |  |  |  | GenBank |
| *E. crenata* | Dai 19467 | US-MI | MT663360 |  |  |  |  | Wu et al. (2020) |
| *E. crenata* | FLAS-F-60866 | US-FL | MH016854 |  |  |  |  | GenBank |
| *E. crenata* | PBM 4146 (TENN) | US-TN | MH558303 |  |  |  |  | GenBank |
| *E. crenata* | Knapp MUOB355435 | US-GA | OL142178 |  |  |  |  | GenBank |
| *E. crenata* | PUL F25993 | US-WI | MW448585 |  |  |  |  | GenBank |
| *E. crenata* | rcw_51 | US | KM103987 |  |  |  |  | GenBank |
| *E. crenata* | iNAT188320919 | US-OH | PQ287882 |  |  |  |  | GenBank |
| *E. crenata* | iNAT183514734 | US-CO | PQ316730 |  |  |  |  | GenBank |
| *E. crenata* | iNAT178068903 | US-AL | PP467902 |  |  |  |  | GenBank |
| *E. crenata* | CMH450 | US-MO | KF800541 |  |  |  |  | Rittenour et al. (2013) |
| *E. crenata* | FLAS-F-69047 | US-FL | OP163244 |  |  |  |  | GenBank |
| *E. crenata* | iNAT205683750 | US-OH | PV263438 |  |  |  |  | GenBank |
| *E. crenata* | FH BHI-F585 | US-MA | MF161299 |  |  |  |  | Haelewaters et al. (2018) |
| *E. crenata* | iNAT157781088 | US-OH | OR987163 |  |  |  |  | Canan et al. (2025) |
| *E. crenata* | Dai 15415 | US-CT | MT663357 |  |  |  |  | Wu et al. (2020) |
| *E. crenata* | Miettinen 19510 (H) | US-TN | PZ630658 |  |  |  |  | this study |
| *Exidia delita* | Enzlin 18-002 (GENT) | NL | PZ014177 |  |  |  |  | this study |
| *E. delita* | Spirin 17810 (H) | FR | PZ630625 | PZ630625 | PZ599909 | PZ621122 |  | this study |
| *Exidia friesiana* | Ahti 74629 (H) | CA-NL | PZ630701 |  |  |  |  | this study |
| *E. friesiana* | Dai 20925 | CN | PP051395 | PP051474 |  |  | PP707034 | GenBank |
| *E. friesiana* | Dai 22832 | CN | PP051324 |  |  |  | PP591739 | GenBank |
| *E. friesiana* | Dai 22863 | CN | PP051321 |  |  |  | PP591736 | GenBank |
| *E. friesiana* | Dai 22952 | CN | PP051323 |  |  |  | PP591738 | GenBank |
| *E. friesiana* | Dai 22923 | CN | PP051322 |  |  |  | PP591737 | GenBank |
| *E. friesiana* | Weiß 313 | DE | AF291275 |  |  |  |  | Weiß & Oberwinkler (2001) |
| *E. friesiana* | Spirin 18545 (H) | FR | PZ630698 |  |  |  | PZ621154 | this study |
| *E. friesiana* | O F-248080 | NO | UDB038121 |  |  |  |  | UNITE |
| *E. friesiana* | Kotiranta 19332 (H) | RU-CHE | PZ630696 |  |  |  |  | this study |
| *E. friesiana* | Spirin 7660 (H) | RU-KHA | PZ630699 |  |  |  |  | this study |
| *E. friesiana* | Spirin 10947 (H) | RU-KHA | PZ630700 |  |  |  |  | this study |
| *E. friesiana* | Spirin 11164 (H) | RU-LEN | PZ630697 |  |  |  |  | this study |
| *E. friesiana* | Spirin 18006 (GB) | SE | PZ630722 |  |  |  | PZ621153 | this study |
| *Exidia glandulosa* | Weiß 365 | DE | AF291279 |  |  |  |  | Weiß & Oberwinkler (2001) |
| *E. glandulosa* | S96 | DE | FJ820584 |  |  |  |  | GenBank |
| *E. glandulosa* | Dai 21231 (BJFC) | FI | MT663369 |  |  |  |  | Wu et al. (2020) |
| *E. glandulosa* | Pisilä 22.5.2021 (H) | FI | PZ630635 |  |  |  |  | this study |
| *E. glandulosa* | Spirin 16064 (H) | FI | PZ630644 |  |  |  |  | this study |
| *E. glandulosa* | Spirin 15500 (H) | FR | PZ630636 |  |  |  |  | this study |
| *E. glandulosa* | Spirin 18039 (H) | FR | PZ630641 |  |  |  |  | this study |
| *E. glandulosa* | LE F-314277 | RU-MO | PZ013382 | PZ013397 |  |  |  | this study |
| *E. glandulosa* | Spirin 7203 (H) | RU-NIZ | PZ630638 |  |  |  |  | this study |
| *E. glandulosa* | LE F-321630 | RU-NGR | PZ013383 | PZ013396 |  |  |  | this study |
| *E. glandulosa* | Ahlefelt 17.5.2025 (GB) | SE | PZ630642 |  |  |  |  | this study |
| *E. glandulosa* | Elleström 27.9.2024 (GB) | SE | PZ630637 |  |  |  |  | this study |
| *E. glandulosa* | Spirin 17068 (GB) | SE | PZ630643 |  |  |  |  | this study |
| *E. glandulosa* | Spirin 18028 (GB) | SE | PZ630639 |  |  |  |  | this study |
| *E. glandulosa* | Spirin 18030 (GB) | SE | PZ630640 | PZ630640 | PZ599910 | PZ621125 |  | this study |
| *Exidia impressa* | Spirin 18143 (H) | FR | PZ630728 |  |  |  |  | this study |
| *E. impressa* | Spirin 7572 (H) | RU-KHA | PZ630626 | PZ630626 | PZ599911 | PZ621123 |  | this study |
| *E. impressa* | LE F-312781 | RU-KRA | PZ013384 | PZ013398 |  |  |  | this study |
| *Exidia inornata* | LIP 240916-002 | FR | PQ662641 | PQ662659 |  |  | PQ658684 | Gruhn et al. (2024) |
| *E. inornata* | AMB68895 | FR | PQ662642 |  |  |  | PQ658685 | Gruhn et al. (2024) |
| *E. inornata* | AMB74354 | FR | PQ662643 |  |  |  |  | Gruhn et al. (2024) |
| *E. inornata* | AMB74393 | FR | PQ662644 |  |  |  |  | Gruhn et al. (2024) |
| *Exidia japonica* | Kotiranta 28786 (H) | RU-PRI | PZ630693 | PZ630693 |  |  |  | this study |
| *E. japonica* | Wu 427 | CN | PP051334 | PP051414 | PP734278 | PP776439 |  | GenBank |
| *Exidia pithya* | MHHNU 8450 | CN | MK172826 |  |  |  |  | GenBank |
| *E. pithya* | SICAUCC 23-0084 | CN | PP060700 |  |  |  |  | GenBank |
| *E. pithya* | LXL01 | CN | MZ645962 |  |  |  |  | GenBank |
| *E. pithya* | SWFU 006051 | CN | MK809452 |  |  |  |  | GenBank |
| *E. pithya* | CLZhao 6051 | CN | MK795133 |  |  |  |  | GenBank |
| *E. pithya* | Dai 18024 (BJFC) | CN | MH213394 |  |  |  |  | Wu et al. (2021) |
| *E. pithya* | GLMC 374 | DE | MT156125 |  |  |  |  | Bien & Damm (2020) |
| *E. pithya* | Dai 21232 (BJFC) | FI | MT663362 |  |  |  |  | Wu et al. (2020) |
| *E. pithya* | Dai 21233 (BJFC) | FI | MT663363 |  |  |  |  | Wu et al. (2020) |
| *E. pithya* | Spirin 15354 (H) | FR | PZ630707 |  |  |  |  | this study |
| *E. pithya* | AMB74802 | FR | PQ662652 |  |  |  |  | Gruhn et al. (2024) |
| *E. pithya* | AMB74799 | FR | PQ662651 |  |  |  |  | Gruhn et al. (2024) |
| *E. pithya* | AMB74138 | FR | PQ662649 |  |  |  |  | Gruhn et al. (2024) |
| *E. pithya* | AMB68364 | FR | PQ662648 |  |  |  |  | Gruhn et al. (2024) |
| *E. pithya* | AMB68079 | FR | PQ662647 |  |  |  |  | Gruhn et al. (2024) |
| *E. pithya* | AMB68048 | FR | PQ662646 |  |  |  |  | Gruhn et al. (2024) |
| *E. pithya* | AMB64679 | FR | PQ662645 |  |  |  |  | Gruhn et al. (2024) |
| *E. pithya* | Cazenave 65230-2 | FR | PQ662650 |  |  |  |  | Gruhn et al. (2024) |
| *E. pithya* | Spirin 13639 (H) | IT | PZ630712 |  |  |  |  | this study |
| *E. pithya* | Viner 2018/25 (H) | RU-DA | PZ630706 |  |  |  |  | this study |
| *E. pithya* | Spirin 3938 (H) | RU-KHA | PZ630705 |  |  |  |  | this study |
| *E. pithya* | Spirin 5651 (H) | RU-KHA | PZ630724 |  |  |  |  | this study |
| *E. pithya* | Spirin 7299 (H) | RU-KHA | PZ630716 |  |  |  |  | this study |
| *E. pithya* | Spirin 7673 (H) | RU-KHA | PZ630720 |  |  |  |  | this study |
| *E. pithya* | Spirin 7725 (H) | RU-KHA | PZ630717 |  |  |  |  | this study |
| *E. pithya* | Spirin 7781 (H) | RU-KHA | PZ630721 |  |  |  |  | this study |
| *E. pithya* | Spirin 7860 (H) | RU-KHA | PZ630719 |  |  |  | PZ621152 | this study |
| *E. pithya* | Spirin 10755 (H) | RU-KHA | PZ630713 |  |  |  |  | this study |
| *E. pithya* | Stepanchikova 5.2017 (H) | RU-KDA | PZ630718 |  |  |  |  | this study |
| *E. pithya* | Spirin 7092 (H) | RU-NIZ | PZ630704 |  |  |  |  | this study |
| *E. pithya* | Spirin 11350b (H) | RU-NIZ | PZ630708 |  |  |  |  | this study |
| *E. pithya* | Spirin 11953 (H) | RU-NIZ | PZ630715 |  |  |  |  | this study |
| *E. pithya* | LE F-347690 | RU-PRI | PZ013385 |  |  |  |  | this study |
| *E. pithya* | Spirin 13129 (H) | SI | PZ630711 |  |  |  |  | this study |
| *E. pithya* | Olsson 19.12.2024 (GB) | SE | PZ630702 |  |  |  |  | this study |
| *E. pithya* | Spirin 17065 (GB) | SE | PZ630725 |  |  |  |  | this study |
| *E. pithya* | Spirin 17218 (GB) | SE | PZ630709 |  |  |  |  | this study |
| *E. pithya* | Spirin 17231 (GB) | SE | PZ630714 |  |  |  |  | this study |
| *E. pithya* | Spirin 17233 (GB) | SE | PZ630723 |  |  |  |  | this study |
| *E. pithya* | Spirin 17235 (GB) | SE | PZ630710 |  |  |  |  | this study |
| *E. pithya* | Spirin 17359 (GB) | SE | PZ630703 |  |  |  | PZ621151 | this study |
| *E. pithya* | Birkebak 26.9.2013 (H) | US-AR | PZ630726 |  |  |  |  | this study |
| *E. pithya* | iNAT197585922 | US-CA | PQ619198 |  |  |  |  | GenBank |
| *E. pithya* | iNAT148043918 | US-CA | OR881192 |  |  |  |  | GenBank |
| *E. pithya* | Russell MycoMap 4415 | US-IN | ON561703 |  |  |  |  | GenBank |
| *E. pithya* | CA FUNDIS iNat146772307 | US-IN | OR081335 |  |  |  |  | GenBank |
| *E. pithya* | BHI-F210b (FH) | US-MA | MF161201 |  |  |  |  | Haelewaters et al. (2018) |
| *E. pithya* | CMH508 | US-MO | KF800599 |  |  |  |  | Rittenour et al. (2013) |
| *E. pithya* | HHB 12209-sp (CFMR) | US-WI | MW353407 |  |  |  |  | Wang & Thorn (2021) |
| *Exidia plumbescens* | J. Schoutteten 04-001 (GENT) | BE | PZ014188 |  |  |  |  | this study |
| *E. plumbescens* | Helo 20210767 (OULU) | FI | PZ630607 | PZ630607 | PZ599912 | PZ621113 |  | this study |
| *E. plumbescens* | Enzlin 19-013 (GENT) | NL | PZ014176 |  |  |  |  | this study |
| *E. plumbescens* | TAAM 7008 | RU-KRA | UDB07686188 | UDB07686189 |  |  |  | this study |
| *E. plumbescens* | Viner 2016/356 (H) | RU-PRI | PZ630695 |  |  |  |  | this study |
| *E. plumbescens* | Spirin 18015 (GB) | SE | PZ630608 |  |  |  |  | this study |
| *‘Exidia’ punctata* | CL Zhao 33767 | CN | PQ783836 | PQ783846 |  |  |  | Jiang et al. (2025) |
| *‘E.’ punctata* | CL Zhao 25823 | CN | PQ783835 |  |  |  |  | Jiang et al. (2025) |
| *‘E.’ punctata* | clone LX041829-122-052-F12 |  | GU054135 |  |  |  |  | GenBank |
| *Exidia recisa* | MW 315 | DE | AF291276 |  |  |  |  | Weiß & Oberwinkler (2001) |
| *E. recisa* | Spirin 12333 (TUF 114806) | EE | UDB0754268 |  |  |  |  | UNITE |
| *E. recisa* | Lindberg 180317 (H) | FI | MT663365 | MT664783 |  |  |  | Wu et al. (2020) |
| *E. recisa* | Miettinen 14913 (H) | FI | MT663366 |  |  |  |  | Wu et al. (2020) |
| *E. recisa* | Miettinen 27519 (H) | FI | PZ630665 |  |  |  |  | this study |
| *E. recisa* | Spirin 18346 (H) | FI | PZ630659 |  |  |  |  | this study |
| *E. recisa* | Spirin 18484 (H) | FI | PZ630660 |  |  |  |  | this study |
| *E. recisa* | Spirin 18488 (H) | FI | PZ630663 |  |  |  |  | this study |
| *E. recisa* | Spirin 18518 (H) | FI | PZ630662 |  |  |  |  | this study |
| *E. recisa* | Spirin 18519 (H) | FI | PZ630661 |  |  |  |  | this study |
| *E. recisa* | Viner 2025/36 (H) | FI | PZ630664 |  |  |  |  | this study |
| *E. recisa* | LY BR-5695 | FR | MT663364 |  |  |  |  | Wu et al. (2020) |
| *E. recisa* | clone 4248_681 | LT | MT236624 |  |  |  |  | Marciulynas et al. (2020) |
| *E. recisa* | O F-248317 | NO | UDB037979 |  |  |  |  | UNITE |
| *Exidia reflexa* | Wu 454 | CN | PP051320 | PP051400 |  |  | PP591735 | GenBank |
| *E. reflexa* | Wu 473 | CN | PP051319 |  |  |  | PP591734 | GenBank |
| *E. reflexa* | Dai 20842 (BJFC) | CN | MN850387 |  |  |  | MN819841 | Ye et al. (2020) |
| *E. reflexa* | Dai 20861 (BJFC) | CN | MN850388 |  |  |  | MN819842 | Ye et al. (2020) |
| *E. reflexa* | Dai 20874 (BJFC) | CN | MN850389 |  |  |  | MN819843 | Ye et al. (2020) |
| *E. reflexa* | Dai 20833 (BJFC) | CN | MN850386 |  |  |  | MN819840 | Ye et al. (2020) |
| *E. reflexa* | LE F-303425 | RU-PRI | PZ013386 | PZ013399 |  |  |  | this study |
| *E. repleta* | Spirin 18598 (H) | FR | PZ630582 |  |  |  |  | this study |
| *E. repleta* | Spirin 18748 (H) | FR | PZ630583 | PZ630583 | PZ599913 | PZ621108 |  | this study |
| *Exidia saccharina* f. *carnea* | TAAM 9819 | RU-KRA | UDB07686185 |  |  |  |  | this study |
| *E. saturata* | Spirin 18746 (H) | FR | PZ630581 | PZ630581 | PZ599914 | PZ621140 |  | this study |
| *E. saturata* | Spirin 18750 (H) | FR | PZ630584 | PZ630584 |  |  |  | this study |
| *Exidia semiorbis* | LE F-262976 | RU-PRI | PZ013387 | PZ013400 |  |  |  | this study |
| *E. semiorbis* | LE F-262977 | RU-PRI | PZ013388 |  |  |  |  | this study |
| *E. semiorbis* | LE F-303433 | RU-PRI | PZ013389 | PZ013401 |  |  |  | this study |
| *Exidia spiculata* | Spirin 8839 (H) | CA-AB | PZ630727 |  |  |  |  | this study |
| *E. spiculata* | iNAT188199522 | CA-AB | PQ847842 |  |  |  |  | GenBank |
| *E. spiculata* | Dai 21677 | CN | PP051328 |  |  |  |  | GenBank |
| *E. spiculata* | Dai 22532 | CN | PP051329 |  |  |  |  | GenBank |
| *E. spiculata* | Yuan 20 | CN | PP051327 |  |  |  | PP591741 | GenBank |
| *E. spiculata* | AMB64822 | FR | PQ662638 |  |  |  |  | Gruhn et al. (2024) |
| *E. spiculata* | AMB74137 | FR | PQ662640 |  |  |  |  | Gruhn et al. (2024) |
| *E. spiculata* | AMB60487 | FR | PQ662636 |  |  |  | PQ658682 | Gruhn et al. (2024) |
| *E. spiculata* | AMB65411 | FR | PQ662639 |  |  |  | PQ658683 | Gruhn et al. (2024) |
| *E. spiculata* | LE F-201999 | RU-LEN | PZ013390 | PZ013402 |  |  |  | this study |
| *Exidia straminea* | Rivoire 7046 | FR | MT663367 | MT664784 |  |  |  | Wu et al. (2020) |
| *E. straminea* | Spirin 18148 (H) | FR | PZ630666 |  |  |  |  | this study |
| *E. straminea* | Spirin 18174 (H) | FR | PZ630667 |  |  |  |  | this study |
| *Exidia subglandulosa* | Dai 22885 | CN | PP051325 | PP051405 |  |  | PP591740 | GenBank |
| *E. subglandulosa* | Wu 270 (BJFC) | CN | MN850381 |  |  |  | MN819838 | Ye et al. (2020) |
| *E. subglandulosa* | Wu 271 (BJFC) | CN | MN850382 |  |  |  | MN819839 | Ye et al. (2020) |
| *E. subglandulosa* | Wu 272 (BJFC) | CN | MN850383 |  |  |  | MN819837 | Ye et al. (2020) |
| *E. subglandulosa* | Wu 273 (BJFC) | CN | MN850384 |  |  |  | MN819836 | Ye et al. (2020) |
| *E. subglandulosa* | Wu 278 (BJFC) | CN | MN850385 |  |  |  |  | Ye et al. (2020) |
| *Exidia tanzanica* | Ryvarden 34395 (O) | TZ | PZ630734 |  |  |  |  | this study |
| *Exidia truncata* | Spirin 17059 (GB) | SE | PZ630650 |  |  |  |  | this study |
| *E. truncata* | Spirin 18022 (GB) | SE | PZ630646 |  |  |  |  | this study |
| *E. truncata* | Spirin 18029 (GB) | SE | PZ630645 | PZ630645 | PZ599915 | PZ621112 |  | this study |
| *E. truncata* | Ben Ali H21586 | TN | KU973844 |  |  |  |  | GenBank |
| *Exidia umbrinella* | LE F-254078 | RU-KC | PZ013391 |  |  |  |  | this study |
| *Exidia uvapassa* | Viner 2016/275 (H) | RU-PRI | PZ630627 | PZ630627 | PZ599916 | PZ621141 |  | this study |
| *Exidia yadongensis* | Wu 305 (BJFC) | CN | MT663376 | MT664792 |  |  |  | Wu et al. (2020) |
| *E. yadongensis* | MHHNU8392 | CN | MK172825 |  |  |  |  | GenBank |
| *E. yadongensis* | HMIGD 27135 | CN | MT276338 |  |  |  |  | GenBank |
| *E. yadongensis* | HMAS 263385 | CN | MT276337 |  |  |  |  | GenBank |
| *E. yadongensis* | HMIGD 41367 | CN | MT276339 |  |  |  |  | GenBank |
| *E. yadongensis* | 2010ydher003 | CN | JN400351 |  |  |  |  | GenBank |
| *E. yadongensis* | Dai 17210 (BJFC) | CN | MT663371 |  |  |  |  | Wu et al. (2020) |
| *E. yadongensis* | Dai 17212 (BJFC) | CN | MT663373 |  |  |  |  | Wu et al. (2020) |
| *E. yadongensis* | Dai 22869 | CN | PP051351 |  |  |  |  | GenBank |
| *E. yadongensis* | 2010ydher004 | CN | JN400352 |  |  |  |  | GenBank |
| *E. yadongensis* | Dai 23318 | CN | PP051352 |  |  |  |  | GenBank |
| *E. yadongensis* | Q Zhao 2358 | CN | MT663377 |  |  |  |  | Wu et al. (2020) |
| *E. yadongensis* | 2010ydher001 | CN | JN400349 |  |  |  |  | GenBank |
| *E. yadongensis* | FLF91 | CN | OR020899 |  |  |  |  | GenBank |
| *E. yadongensis* | HKAS 34367 | CN | MT276336 |  |  |  |  | GenBank |
| *E. yadongensis* | MHHNU 8392 | CN | MT276340 |  |  |  |  | GenBank |
| *E. yadongensis* | STDS-2-44 | JP | LC098751 |  |  |  |  | GenBank |
| *E. yadongensis* | Spirin 7462 (H) | RU-KHA | PZ630669 |  |  |  |  | this study |
| *E. yadongensis* | Spirin 10874 (H) | RU-KHA | PZ630668 |  |  |  |  | this study |
| *E. yadongensis* | Spirin 10904 (H) | RU-KHA | PZ630670 |  |  |  |  | this study |
| *E. yadongensis* | LE F-303418 | RU-PRI | PZ013392 | PZ013403 |  |  |  | this study |
| *Exidia zelleri* | Lebeuf MQ18R011 QFB30094 | CA-QC | MN992149 |  |  |  |  | GenBank |
| *E. zelleri* | Dai 18111 | CN | PP051364 |  |  |  |  | GenBank |
| *E. zelleri* | Dai 21706 | CN | PP051365 |  |  |  |  | GenBank |
| *E. zelleri* | Dai 22533 | CN | PP051367 |  |  |  |  | GenBank |
| *E. zelleri* | Dai 23155 | CN | PP051370 |  |  |  |  | GenBank |
| *E. zelleri* | Dai 23156 | CN | PP051369 |  |  |  |  | GenBank |
| *E. zelleri* | Dai 25827 | CN | PP051363 |  |  |  |  | GenBank |
| *E. zelleri* | Dai 25828 | CN | PP051362 |  |  |  |  | GenBank |
| *E. zelleri* | Wu 277 | CN | PP051368 |  |  |  |  | GenBank |
| *E. zelleri* | Wu 400 | CN | PP051366 |  |  |  |  | GenBank |
| *E. zelleri* | Spirin 7868 (H) | RU-KHA | PZ630652 |  |  |  |  | this study |
| *E. zelleri* | Spirin 10927 (H) | RU-KHA | PZ630648 |  |  |  |  | this study |
| *E. zelleri* | LE F-347689 | RU-KHM | PZ013393 |  |  |  |  | this study |
| *E. zelleri* | Spirin 10456 (H) | RU-LEN | PZ630647 |  |  |  |  | this study |
| *E. zelleri* | LE F-303406 | RU-PRI | PZ013394 | PZ013404 |  |  |  | this study |
| *E. zelleri* | Spirin 17775 (GB) | SE | PZ630651 | PZ630651 | PZ599917 | PZ621111 |  | this study |
| *E. zelleri* | Spirin 17780 (GB) | SE | PZ630649 |  |  |  |  | this study |
| *Exidia* sp. 1 | iNAT236454698 | US-CA | PV455405 |  |  |  |  | GenBank |
| *Exidia* sp. 1 | iNAT206157816 | US-CA | PQ146076 |  |  |  |  | GenBank |
| *Exidia* sp. 2 | Dai 22961 | CN | PP051348 |  |  |  |  | GenBank |
| *Exidia* sp. 2 | Dai 23238 | CN | PP051347 |  |  |  |  | GenBank |
| *Exidia* sp. 2 | Dai 23242 | CN | PP051349 |  |  |  |  | GenBank |
| *Exidia* sp. 2 | Dai 23332 | CN | PP051350 |  |  |  |  | GenBank |
| *Exidiopsis effusa* | Bleys 19012025 | BE | PV330055 |  |  |  |  | GenBank |
| *E. effusa* | Spirin 15511 (H) | FR | PZ630618 |  |  |  |  | this study |
| *E. effusa* | J. Schoutteten 92-001 (GENT) | LU | PZ014187 |  |  |  |  | this study |
| *E. effusa* | Spirin 10024 (O) | NO | PZ630729 |  |  |  |  | this study |
| *E. effusa* | Spirin 10026 (O) | NO | PZ630730 |  |  |  |  | this study |
| *E. effusa* | Olson 3.3.2025 (GB) | SE | PZ630615 | PZ630615 | PZ599918 |  |  | this study |
| *E. effusa* | Olsson 21.4.2025 (GB) | SE | PZ630617 |  |  | PZ621119 |  | this study |
| *E. effusa* | Spirin 18205 (GB) | SE | PZ630616 | PZ630616 |  | PZ621115 |  | this study |
| *Exidiopsis perflua* | BF-OTU139 | FI | AM901783 |  |  |  |  | Pitkäranta et al. (2008) |
| *E. perflua* | Miettinen 19136 (H) | FI | KX262145 |  |  |  |  | Malysheva & Spirin (2017) |
| *E. perflua* | Spirin 14386 (H) | RU-NIZ | PZ630619 |  |  | PZ621121 |  | this study |
| *E. perflua* | Spirin 12638 (H) | SI | PZ630620 |  |  |  |  | this study |
| *Heteroradulum deglubens* | Spirin 16936 (GB) | SE | PZ630612 | PZ630612 | PZ599919 | PZ621132 |  | this study |
| *L. farinellum* | LWZ20180904-14 | CN | OM801935 |  |  |  |  | Liu et al. (2022) |
| *L. farinellum* s.l. | LWZ20191104-29 | CN | OM801938 |  |  |  |  | Liu et al. (2022) |
| *L. farinellum* s.l. | LWZ20180904-19 | CN | OM801936 |  |  |  |  | Liu et al. (2022( |
| *L. farinellum* s.l. | LWZ20180904-24 | CN | OM801937 |  |  |  |  | Liu et al. (2022) |
| *L. farinellum* s.l. | Vondrak 19330 | CZ | OM104979 |  |  |  |  | GenBank |
| *L. farinellum* s.l. | Vondrak 19329 | CZ | OM104980 |  |  |  |  | GenBank |
| *L. farinellum* | Weiß 331 | DE | AF291280 |  |  |  |  | Weiß & Oberwinkler (2001) |
| *L. farinellum* | Miettinen 20585 (H) | FI | PZ630671 | PZ630671 |  |  |  | this study |
| *L. farinellum* | O F-253618 | SE | PlutoF |  |  |  |  | PlutoF |
| *L. farinellum* | Spirin 12586 (H) | SI | PZ630674 |  |  |  | PZ621150 | this study |
| *L. farinellum* | Fritzson MV-2005 | SE | PQ653258 |  |  |  |  | GenBank |
| *L. farinellum* | Olsson 13.4.2025 (GB) | SE | PZ630676 |  |  |  |  | this study |
| *L. farinellum* | Spirin 16599 (GB) | SE | PZ630673 |  |  |  | PZ621148 | this study |
| *Leiostroma incanum* | Viner 2019/280 (H) | FR | PZ630678 | PZ630678 | PZ599920 | PZ621105 | PZ621143 | this study |
| *L. incanum* | Spirin 16722 (H) | SI | PZ630677 |  |  |  | PZ621147 | this study |
| *Leiostroma infecundum* | Viner 2022/1008 (H) | ES | PZ630672 |  |  |  | PZ621144 | this study |
| *L. infecundum* | Viner 2022/1091a (H) | ES | PZ630681 | PZ630681 | PZ599921 | PZ621124 | PZ621145 | this study |
| *Leiostroma squalidum* | Savchenko 171124/1010 (H) | KE | PZ630683 | PZ630683 |  |  |  | this study |
| *L. squalidum* | Ryvarden 23725 (O) | ZW | PZ630682 |  |  |  |  | this study |
| *Leiostroma* sp. 1. | Spirin 8873 (H) | CA-AB | PZ630679 |  |  |  | PZ621149 | this study |
| *Leiostroma* sp. 1 | Eriksson 7316 (GB) | CA-NWT | PZ630680 |  |  |  |  | this study |
| *Leiostroma* sp. 2 | Spirin 18795 (H) | FR | PZ630675 |  |  |  | PZ621146 | this study |
| *Nodulochaete fissurata* | CLZhao 27533 | CN | PQ166573 | PQ166565 |  |  |  | Dong et al. (2025a) |
| *N. fissurata* | CLZhao 22803 | CN | PQ166569 | PQ166561 |  |  |  | Dong et al. (2025a) |
| *Pholiobasidion senex* | Grootmyers 6.10.2019 (H) | US-OH | PZ630741 | PZ630741 | PZ599922 | PZ621126 |  | this study |
| *Proterochaete abstrusa* | Læssøe 2014-695676 (C) | DK | PZ630656 |  |  |  |  | this study |
| *P. abstrusa* | Heilmann-Clausen DMS-456745 (C) | DK | PZ630653 |  |  |  |  | this study |
| *P. abstrusa* | Spirin 17946 (H) | FR | PZ630655 | PZ630655 | PZ599923 | PZ621138 |  | this study |
| *P. abstrusa* | Schoutteten 19-316 (GENT) | IT | PZ014184 |  |  |  |  | this study |
| *P. abstrusa* | Spirin 13834 (H) | IT | PZ630657 | PZ630657 |  |  |  | this study |
| *P. abstrusa* | Spirin 13943 (H) | SI | PZ630654 | PZ630654 |  |  |  | this study |
| *Proterochaete adusta* | Van Autgaerden 20-048 (GENT) | BE | PZ014196 |  |  |  |  | this study |
| *P. adusta* | Van Autgaerden 20-067 (GENT) | BE | PZ014197 |  |  |  |  | this study |
| *P. adusta* | Van Autgaerden 20-041 (GENT) | BE | PZ014195 |  |  |  |  | this study |
| *P. adusta* | Julkunen 1062 (OULU) | FI | PZ630606 | PZ630606 | PZ599924 | PZ621103 |  | this study |
| *P. adusta* | Spirin 9021 (H) | CA-AB | MK391520 |  |  |  |  | Alvarenga et al. (2019) |
| *P. adusta* | Miettinen 10519 (H) | CN | MK391519 |  |  |  |  | Alvarenga et al. (2019) |
| *P. adusta* | He 6076 | CN | OM100763 |  |  |  |  | GenBank |
| *P. adusta* | Enzlin 20-015 (GENT) | NL | PZ014201 |  |  |  |  | this study |
| *P. adusta* | Enzlin 21-039 (GENT) | NL | PZ014199 |  |  |  |  | this study |
| *P. adusta* | Enzlin 21-043 (GENT) | NL | PZ014200 |  |  |  |  | this study |
| *P. adusta* | Bruggeman-Nannenga 7148 (GENT) | NL | PZ014198 |  |  |  |  | this study |
| *P. adusta* | Larsson 17537 (O) | NO | MK391522 |  |  |  |  | Alvarenga et al. (2019) |
| *P. adusta* | Spirin 4910 (H) | RU-KHA | MK391521 |  |  |  |  | Alvarenga et al. (2019) |
| *Proterochaete africana* | Ryvarden 44921 (O) | UG | PZ630687 | PZ630687 |  |  |  | this study |
| *Proterochaete bambusicola* | He 4236 (BJFC) | CN | MH178259 | MH178283 |  |  |  | Li et al. (2023) |
| *P. bambusicola* | He 4604 (BJFC) | CN | MH178260 | MH178284 |  |  |  | Li et al. (2023) |
| *P. bambusicola* | He 3344 | CN | MH178258 |  |  |  |  | GenBank |
| *P. bambusicola* | Dai 26239 | CN | PQ167128 |  |  |  |  | GenBank |
| *P. bambusicola* | Zhao 16100 | CN | PP392888 |  |  |  |  | GenBank |
| *Proterochaete furfurosa* | Ryvarden 44895 (O) | UG | PZ630685 | PZ630685 |  |  |  | this study |
| *P. furfurosa* | Ryvarden 44939 (O) | UG | PZ630686 |  |  |  |  | this study |
| *P. furfurosa* | Ryvarden 33459 (O) | ZW | PZ630684 |  |  |  |  | this study |
| *P. latispora* | He 20120923-20 (BJFC) | CN | MH178261 | MH178285 |  |  |  | Li et al. (2023) |
| *Proterochaete letendreana* | Spirin 15808 (H) | AT | PZ630585 |  |  |  |  | this study |
| *P. letendreana* | Van Autgaerden 19-005 (GENT) | BE | PZ014191 |  |  |  |  | this study |
| *P. letendreana* | Van Autgaerden 19-008 (GENT) | BE | PZ014192 |  |  |  |  | this study |
| *P. letendreana* | Van Autgaerden 19-009 (GENT) | BE | PZ014193 |  |  |  |  | this study |
| *P. letendreana* | Van Autgaerden 19-011 (GENT) | BE | PZ014194 |  |  |  |  | this study |
| *P. letendreana* | Van Autgaerden 20-052 (GENT) | BE | PZ014183 |  |  |  |  | this study |
| *P. letendreana* | J. Schoutteten 97-164 (GENT) | BE | PZ014190 |  |  |  |  | this study |
| *P. letendreana* | J. Schoutteten 07-004 (GENT) | BE | PZ014189 |  |  |  |  | this study |
| *P. letendreana* | J. Schoutteten 18-003 (GENT) | BE | PZ014186 |  |  |  |  | this study |
| *P. letendreana* | Schoutteten 16-027 (GENT) | BE | PZ014185 |  |  |  |  | this study |
| *P. letendreana* | Montri-24 | CH | MK028373 |  |  |  |  | Hofstetter et al. (2019) |
| *P. letendreana* | FO 46291 | DE | AF291282 |  |  |  |  | Weiß & Oberwinkler (2001) |
| *P. letendreana* | Spirin 15652 (H) | FR | PZ630588 | PZ630588 |  |  |  | this study |
| *P. letendreana* | Spirin 15764 (H) | FR | PZ630594 |  |  |  |  | this study |
| *P. letendreana* | Spirin 16172 (H) | FR | PZ630731 |  |  |  |  | this study |
| *P. letendreana* | Spirin 16470 (H) | FR | PZ630589 |  |  |  |  | this study |
| *P. letendreana* | Spirin 17157 (H) | FR | PZ630586 | PZ630586 |  |  |  | this study |
| *P. letendreana* | Spirin 17866 (H) | FR | PZ630591 | PZ630591 | PZ599926 | PZ621109 |  | this study |
| *P. letendreana* | K(M) 257342 | GB | MZ159758 |  |  |  |  | GenBank |
| *P. letendreana* | Bruggeman 6091 (GENT) | NL | PZ014181 |  |  |  |  | this study |
| *P. letendreana* | Bruggeman 6137 (GENT) | NL | PZ014182 |  |  |  |  | this study |
| *P. letendreana* | Enzlin 18-059 (GENT) | NL | PZ014179 |  |  |  |  | this study |
| *P. letendreana* | Enzlin 18-102 (GENT) | NL | PZ014178 |  |  |  |  | this study |
| *P. letendreana* | Enzlin 19-001 (GENT) | NL | PZ014180 |  |  |  |  | this study |
| *P. letendreana* | Spirin 11948 (H) | RU-NIZ | PZ630592 |  |  |  |  | this study |
| *P. letendreana* | Spirin 16860 (H) | SI | PZ630587 |  |  |  |  | this study |
| *P. letendreana* | Spirin 17045 (GB) | SE | PZ630590 |  |  |  |  | this study |
| *P. letendreana* | Spirin 17051 (GB) | SE | PZ630593 |  |  |  |  | this study |
| *P. letendreana* | Spirin 17054 (GB) | SE | PZ630732 |  |  |  |  | this study |
| *P. letendreana* | CWU (Myc) Ab694 | UA | OP809643 |  |  |  |  | GenBank |
| *Proterochaete macrogyna* | Kotiranta 27410 (H) | RU-SA | PZ630610 | PZ630610 | PZ599927 | PZ621133 |  | this study |
| *P. macrogyna* | TAAM 56485 | RU-SA | UDB07686186 |  |  |  |  | this study |
| *Proterochaete mucedinea* | Viner 2024/205 (H) | AR | PZ630689 |  |  |  |  | this study |
| *P. mucedinea* | Viner 2024/233 (H) | AR | PZ630690 | PZ630690 | PZ599929 | PZ621131 |  | this study |
| *Proterochaete paniculata* | Bandoni 7310A (BPI) | CA-BC | PZ630609 |  |  |  |  | this study |
| *P. paniculata* | Spirin 8549 (H) | US-WA | PZ630611 | PZ630611 | PZ599928 | PZ621137 |  | this study |
| *Proterochaete stipidosa* | Viner 2024/266 (H) | AR | PZ630688 | PZ630688 | PZ599925 | PZ621114 |  | this study |
| *Proterochaete* sp. | CL Zhao 35782 | CN | PV441134 |  |  |  |  | GenBank |
| *Protoacia delicata* | Spirin 13010 (H) | RU-NIZ | ON117837 | ON109165 | ON217542 | PZ240092 |  | Spirin et al. (2023, 2026b) |
| *Protodaedaelea foliacea* | Miettinen 13054 (H) | ID | MG757507 | MG757507 |  |  |  | Malysheva et al. (2018) |
| *Protodaedalea hispida* | Spirin 5139 (H) | RU-KHA | MG757510 | MG757510 |  |  |  | Malysheva et al. (2018) |
| *Pseudohydnum gelatinosum* | Miettinen 21866 (H) | FI | ON117838 | OM451461 | ON217543 | PZ240088 |  | Spirin et al. (2023, 2026b) |
| *Punctochaete murina* | CLZhao 32118 | CN | PP819689 | PP819701 |  |  |  | Dong et al. (2025b) |
| *Scrupulispora perparvula* | Spirin 15819a (H) | AT | PZ630740 | PZ630740 |  |  |  | this study |
| *S. perparvula* | Spirin 16358 (H) | FR | PZ630739 | PZ630739 |  | PZ621120 |  | this study |
| *Tegmenicium peritrichum* | Michelitsch Q8453/4 (GJO) | AT | PZ630632 | PZ630632 |  |  |  | this study |
| *T. peritrichum* | Michelitsch Q8354/4 (GJO) | AT | PZ630633 |  |  |  |  | this study |
| *T. peritrichum* | TAAM 016907 | EE | UDB07674230,  UDB0767428 |  |  |  |  | UNITE |
| *T. peritrichum* | Spirin 18537 (H) | FI | PZ630631 | PZ630631 | PZ599930 | PZ621106 |  | this study |
| *T. peritrichum* | Rivoire 7162 (LY) | FR | PZ630634 |  |  |  |  | this study |
| *Tremellochaete atlantica* | Dai 22375 (BJFC) | CN | OP605375 | OP605359 |  |  |  | Tohtirjap et al. (2023) |
| *T. australiensis* | Dai 18758 (BJFC) | AU | OP605380 | OP605364 |  |  |  | Tohtirjap et al. (2023) |
| *Tremellochaete cerradensis* | SP467241 | BR | MK391524 | MK391530 |  |  |  | Alvarenga et al. (2019) |
| *Ulocolla corticioides* | Spirin 18923 (H) | FR | PZ630579 |  |  |  |  | this study |
| *U. corticioides* | Spirin 18928 (H) | FR | PZ630580 |  |  |  |  | this study |
| *U. corticioides* | Viner 2025/94A (H) | GR | PZ630578 | PZ630578 | PZ599931 | PZ621134 |  | this study |
| *U. corticioides* | Uotila 40874 (H) | MA | PZ630733 |  |  |  |  | this study |
| *Ulocolla grisea* | Viner 2026/150 (H) | ES | PZ630735 |  |  |  |  | this study |
| *U. grisea* | Spirin 15604 (H) | FR | PZ630630 | PZ630630 | PZ599933 | PZ621102 |  | this study |
| *Ulocolla griseobrunnea* | Spirin 7674 (H) | RU-KHA | PZ099310 | PZ099310 | PZ599934 | PZ240096 |  | Spirin et al. (2026b); this study |
| *U. griseobrunnea* | TAAM 017048 | RU-YAN | UDB07686187 |  |  |  |  | this study |
| *Ulocolla orbiculata* | Spirin 7487 (H) | RU-KHA | PZ630629 | PZ630629 |  |  |  | this study |
| *Ulocolla pinicola* | Spirin 8916 (H) | CA-BC | PZ630574 | PZ630574 |  |  |  | this study |
| *U. pinicola* | clone L049479E09 |  | JX136056 |  |  |  |  | Huffman et al. (2013) |
| *Ulocolla saccharina* | Miettinen 18284.1 (H) | FI | PZ630577 |  |  |  |  | this study |
| *U. saccharina* | Dai 22187 | FR | OP605371 |  |  |  |  | Tohtirjap et al. (2023) |
| *U. saccharina* | Dai 22195 | FR | OP605370 |  |  |  |  | Tohtirjap et al. (2023) |
| *U. saccharina* | Spirin 18187 (H) | FR | PZ630575 | PZ630575 | PZ599932 | PZ621104 |  | this study |
| *U. saccharina* | HFRG_JW230325_1 | GB | OR907167 |  |  |  |  | GenBank |
| *U. saccharina* | HFRG_PN250322_1 | GB | PV690307 |  |  |  |  | GenBank |
| *U. saccharina* | Spirin 13748 (H) | IT | PZ630576 |  |  |  |  | this study |
| *Ulocolla subrepanda* | Dai 21719 (BJFC) | CN | OP605368 |  |  |  |  | Tohtirjap et al. (2023) |
| *U. subrepanda* | Dai 21720 (BJFC) | CN | OP605369 |  |  |  |  | Tohtirjap et al. (2023) |
| *U. subrepanda* | Dai 15848 (BJFC) | CN | OP605366 |  |  |  |  | Tohtirjap et al. (2023) |
| *U. subrepanda* | Dai 15890 (BJFC) | CN | OP605367 |  |  |  |  | Tohtirjap et al. (2023) |
| *U. subrepanda* | Wu 761 | CN | PP051379 |  |  |  |  | GenBank |
| *U. subrepanda* | RoKi 88 | DE | AF291277 |  |  |  |  | Weiß & Oberwinkler (2001) |
| *U. subrepanda* | TUF102956 | EE | UDB019410 |  |  |  |  | UNITE |
| *U. subrepanda* | Miettinen 15787 (H) | FI | PZ630572 |  |  |  |  | this study |
| *U. subrepanda* | Spirin 16132 (H) | FI | PZ630571 |  |  |  |  | this study |
| *U. subrepanda* | O-F-75950 | NO | UDB035291 |  |  |  |  | UNITE |
| *U. subrepanda* | Miettinen 13486.1 (H) | NO | PZ630570 |  |  |  |  | this study |
| *U. subrepanda* | Spirin 6841 (H) | RU-LEN | PZ630573 |  |  |  |  | this study |
| *U. subrepanda* | Spirin 16629 (GB) | SE | PZ630569 |  |  |  |  | this study |

**Additional references** (for other references, see the main text)

Alshammari NI, Stephenson SL (2018) A preliminary study of wood-decay fungi in forests of northwest Arkansas. Current Research in Environmental and Applied Mycology 8: 556–563. <https://doi.org/10.5943/cream/8/5/4>

Alvarenga RLM, Gibertoni TB (2021) *Crystallodon* Alvarenga gen. nov., a new genus of the *Auriculariales* from the Neotropics. Cryptogamie Mycologie 42(2): 17–24. <https://doi.org/10.5252/cryptogamie-mycologie2021v42a2>

Bien S, Damm U (2020) *Prunus* trees in Germany – a hideout of unknown fungi? Mycological Progress 19: 667–690. <https://doi.org/10.1007/s11557-020-01586-4>

Haelewaters D, Dirks AC, Kappler LA et al. (2018) A preliminary checklist of fungi at the Boston Harbor Islands. Northeastern Naturalist 25(9): 45–76. <https://doi.org/10.1656/045.025.s904Canan> K, Quark M, Coulon A et al. (2025) Ohio Mushroom DNA Lab: Nanopore strikes back, one year of achievements and challenges. McIlvainea 34: 1–28.

Hofstetter V, Buyck B, Eyssartier G et al. (2019) The unbearable lightness of sequenced-based identification. Fungal Diversity 96: 243–284. <https://doi.org/10.1007/s13225-019-00428-3>

Huffman JA, Prenni AJ, DeMott PJ et al. (2013) High concentrations of biological aerosol particles and ice nuclei during and after rain. Atmospheric Chemistry and Physics 13: 6151–6164. <https://doi.org/10.5194/acp-13-6151-2013>

Marciulynas A, Marciulyniene D, Lynikiene J et al. (2020) Fungi and oomycetes in the irrigation water of forest nurseries. Forests 11(4): 459. <https://doi.org/10.3390/f11040459>

Pitkäranta M, Meklin T, Hyvärinen A et al. (2008) Analysis of fungal flora in indoor dust by ribosomal DNA sequence analysis, quantitative PCR, and culture. Applied and Environmental Microbiology 74: 233–244. <https://doi.org/10.1128/AEM.00692-07>

Rittenour WR, Ciaccio CE, Barnes CS et al. (2013) Internal transcribed spacer rRNA gene sequencing analysis of fungal diversity in Kansas City indoor environments. Environmental Science: Processes and Impacts 16: 33–43. <https://doi.org/10.1039/C3EM00441D>

Spirin V, Malysheva V, Savchenko A et al. (2026b) *Oliveonia* (*Auriculariales*, *Basidiomycota*), a potentially ectomycorrhizal genus with high species diversity. Fungal Diversity (submitted).

Vlasák J, Vlasák Jr J, Ryvarden L (2016) Studies in Neotropical polypores 42. Synopsis Fungorum 35: 9–33.

Wang SR, Thorn RG (2021) *Exidia qinghaiensis*, a new species from China. Mycoscience 62: 212–216. <https://doi.org/10.47371/mycosci.2021.03.002>
