# Supplementary material for: Exidia and Exidiopsis (Auriculariales, Basidiomycota): a reconsideration
Source: IMA Fungus. 2026 Jul 29;17:e189042. doi: 10.3897/imafungus.17.189042 (PMC13444229; doi:10.3897/imafungus.17.189042)
Supplement: Supplementary file 5 [file imafungus-17-e189042-s003.doc]

Supplementary material 3

DNA sequence alignments used in the analyses

| Dataset | Figure # | Specimens included | Original alignment length | Edited alignment length | Parsimony informative characters | intron model of evolution | *RPB1* exon position 1–2 model of evolution | *RPB1* exon position 3 model of evolution | *RPB2* exon position 1–2 model of evolution | *RPB2* exon position 3 model of evolution | ITS model of evolution | LSU model of evolution | *TEF1* exon position 1–2 model of evolution | *TEF1* exon position 3 model of evolution |
| --- | --- | --- | --- | --- | --- | --- | --- | --- | --- | --- | --- | --- | --- | --- |
| *Auriculariaceae* ITS + LSU + *RPB1 + RPB2* | 1 | 48 | 5240 | 3304 | 1326 | K80+G | GTR+G | HKY+I+G | GTR+I+G | GTR+G | SYM+I+G | SYM+I+G |  |  |
| *Auriculariaceae* ITS + LSU + *RPB2* | 2 | 51 | 3417 | 2202 | 723 |  |  |  | HKY+I+G | GTR+G | GTR+I+G | SYM+I+G |  |  |
| *Proterochaete* ITS + LSU | 3 | 17 | 2177 | 1335 | 191 |  |  |  |  |  | SYM+G | K80+I+G |  |  |
| *Proterochaete* ITS | 4 | 65 | 2177 | 407 | 129 |  |  |  |  |  | SYM+G |  |  |  |
| *Leiostroma* ITS + *TEF1* | 5 | 8 | 3150 | 906 | 31 | JC |  |  |  |  | K80 |  | F81+I | JC+I |
| *Exidiopsis* s. str. ITS | 6 | 12 | 1714 | 430 | 5 |  |  |  |  |  | K80 |  |  |  |
| *Exidia* s. str. ITS + LSU | 7 | 28 | 2156 | 1172 | 121 |  |  |  |  |  | SYM+I+G | SYM+I+G |  |  |
| *Exidia glandulosa* ITS | 8 | 38 | 2015 | 508 | 59 |  |  |  |  |  | SYM+I+G |  |  |  |
| *Exidia recisa* ITS | 9 | 69 | 2011 | 464 | 40 |  |  |  |  |  | SYM+G |  |  |  |
| *Exidia pithya* ITS | 10 | 80 | 2017 | 437 | 31 |  |  |  |  |  | HKY + I |  |  |  |
| *Exidia pithya* ITS + *TEF1* | 11 | 39 | 1158 | 855 | 96 | K80+I+G4 |  |  |  |  | K80+G4 |  | HKY | K80+I+G4 |
| *Ulocolla saccharina* ITS | 12 | 20 | 2201 | 467 | 11 |  |  |  |  |  | K80+I |  |  |  |
| *Adustochaete* ITS + LSU | 13 | 20 | 2437 | 1357 | 153 |  |  |  |  |  | SYM+G | K80+I+G |  |  |
| *Adustochaete* ITS | 14 | 30 | 2437 | 495 | 131 |  |  |  |  |  | SYM+I+G |  |  |  |
| *Leiostroma* ITS | Suppl 4 | 5 | 22 | 2163 | 364 | 42 |  |  |  |  | K80+I+G |  |  |  |
| *Auriculariaceae* ITS + LSU | Suppl 5 | 52 | 2162 | 1481 | 528 |  |  |  |  |  | SYM+I+G | SYM+I+G |  |  |
